# Luminescent zinc(ii) and copper(i) complexes for high-performance solution-processed monochromic and white organic light-emitting devices†
†Electronic supplementary information (ESI) available: Experimental procedures, device performances, and computational details. CCDC 1054456, 1400003 and 1400004. For ESI and crystallographic data in CIF or other electronic format see DOI: 10.1039/c4sc03161j



**DOI:** 10.1039/c4sc03161j

**Published:** 2015-06-02

**Authors:** Gang Cheng, Gary Kwok-Ming So, Wai-Pong To, Yong Chen, Chi-Chung Kwok, Chensheng Ma, Xiangguo Guan, Xiaoyong Chang, Wai-Ming Kwok, Chi-Ming Che

**Affiliations:** a State Key Laboratory of Synthetic Chemistry , HKU-CAS Joint Laboratory on New Materials, and Department of Chemistry , The University of Hong Kong , Pokfulam Road , Hong Kong SAR , China . Email: cmche@hku.hk; b HKU Shenzhen Institute of Research and Innovation , Shenzhen 518053 , China; c Key Laboratory of Photochemical Conversion and Optoelectronic Materials , Technical Institute of Physics and Chemistry , Chinese Academy of Sciences , Beijing 100190 , China; d State Key Laboratory on Integrated Optoelectronics , College of Electronic Science and Engineering , Jilin University , Changchun 130012 , China; e Department of Applied Biology and Chemical Technology , The Hong Kong Polytechnic University , Hung Hom, Kowloon , Hong Kong SAR , China; f School of Chemistry and Chemical Engineering , Shenzhen University , Shenzhen 518060 , China

## Abstract

The synthesis and spectroscopic properties of luminescent tetranuclear zinc(ii) complexes of substituted 7-azaindoles and a series of luminescent copper(i) complexes containing 7,8-bis(diphenylphosphino)-7,8-dicarba-*nido*-undecaborate ligand are described. These complexes are stable towards air and moisture. Thin film samples of the luminescent copper(i) complexes in 2,6-dicarbazolo-1,5-pyridine and zinc(ii) complexes in poly(methyl methacrylate) showed emission quantum yields of up to 0.60 (for **Cu-3**) and 0.96 (for **Zn-1**), respectively. Their photophysical properties were examined by ultrafast time-resolved emission spectroscopy, temperature dependent emission lifetime measurements and density functional theory calculations. Monochromic blue and orange solution-processed OLEDs with these Zn(ii) and Cu(i) complexes as light-emitting dopants have been fabricated, respectively. Maximum external quantum efficiency (EQE) of 5.55% and Commission Internationale de l'Eclairage (CIE) coordinates of (0.16, 0.19) were accomplished with the optimized **Zn-1**-OLED while these values were, respectively 15.64% and (0.48, 0.51) for the optimized **Cu-3**-OLED. Solution-processed white OLEDs having maximum EQE of 6.88%, CIE coordinates of (0.42, 0.44), and colour rendering index of 81 were fabricated by using these luminescent Zn(ii) and Cu(i) complexes as blue and orange light-emitting dopant materials, respectively.

## Abstract 2

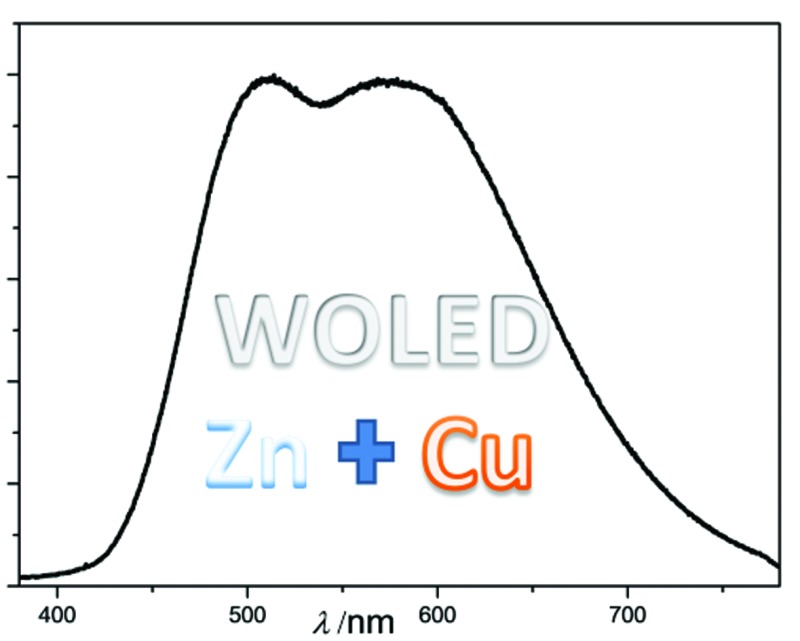
High performance orange (EQE up to 15.64%) and white (EQE up to 6.88%) solution processed OLEDs fabricated solely with emitters of non-platinum group metals were reported. The white device has CIE coordinates of (0.42, 0.44) and CRI of 81.

## Introduction

**Scheme 1:**
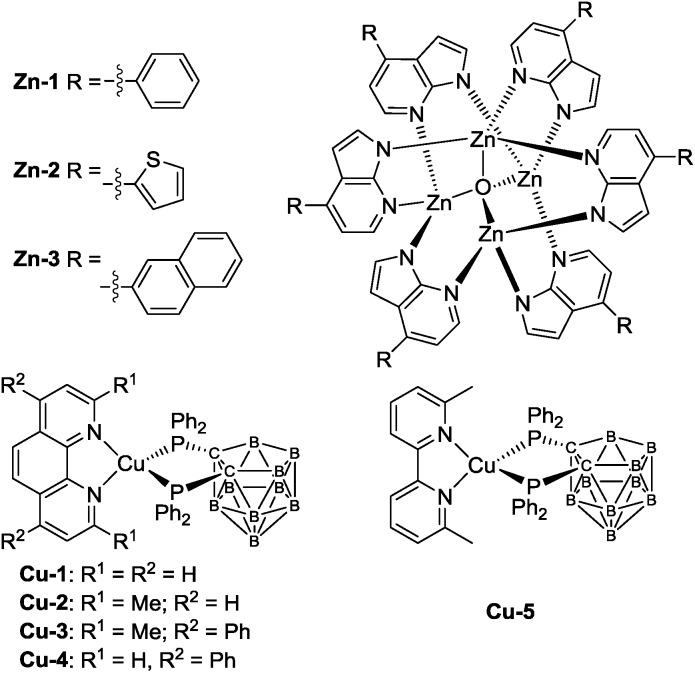
Structures of **Zn-1–Zn-3** and **Cu-1–Cu-5**.

Organic light-emitting diodes (OLEDs), particularly the solution processed ones, are appealing new technology for display and lighting purposes.^1–4^ For high performance OLEDs, phosphorescent dopant materials based on complexes of platinum group metals such as iridium(iii), platinum(ii), osmium(ii) and gold(iii) are commonly used, some of which have already demonstrated industrial applications.^5–8^ On the other hand, the applications of luminescent complexes of earth abundant metals such as that of copper(i) and zinc(ii) described in this work in OLED science and technology are relatively sparse. In the literature, the applications of luminescent Zn(ii) complexes in materials science are well documented. As examples, Zn(ii)-2-(2-hydroxyphenyl)benzothiazolates have been used as white-light emitting, blue-light emitting, electron transporter, and/or host materials in OLEDs.^9,10a–g^ However, electroluminescent (EL) efficiencies of white^10*d*^ and blue^9*d*^ OLEDs with most reported luminescent Zn(ii) complexes are not high with maximum current efficiencies (CEs) of 1.39 and 0.54 cd A^–1^, respectively. In recent work, by using the principle of thermally activated delayed fluorescence (TADF), Adachi and co-workers reported high performance green OLEDs with luminescent Zn(ii) complexes.^10*h*^ Besides Zn(ii) complexes, there has been a spurred interest to develop luminescent Cu(i) materials for OLEDs. With luminescent Cu(i) dopant material, high performance OLEDs with EQE of up to 21.3% have been reported in the literature.^11–13^ Nonetheless, the reported luminescent Cu(i) complexes that gave high EQEs in OLEDs show green emission and/or not stable towards air and moisture.^12b,13c^ A charged Cu(i) complex had been reported as light-emitting material in solution-processed OLED with EQE of up to 15.0%.^13*c*^ In the present work, we report the spectroscopic and photophysical properties of a panel of high efficiency, blue fluorescent Zn(ii) complexes **Zn-1**, **Zn-2** and **Zn-3** and a series of air-stable, charge-neutral luminescent Cu(i) complexes containing 7,8-bis(diphenylphosphino)-7,8-dicarba-*nido*-undecaborate ligand (Scheme 1). The emissions of **Cu-2**, **Cu-3** and **Cu-5** have been confirmed to be TADF by nanosecond time-resolved emission spectroscopy, temperature dependent emission lifetime measurements and density functional theory (DFT) calculations. Solution-processed monochromic OLEDs with these luminescent Zn(ii) and Cu(i) complexes have been fabricated and characterized. With **Zn-1**, **Zn-2** and **Zn-3** as blue emitters for solution-processed OLEDs, high CE of up to 8.12 cd A^–1^, corresponding EQE of 5.55%, and Commission Internationale de l'Eclairage (CIE) coordinates of (0.16, 0.19) have been achieved with **Zn-1** as emitter. For orange **Cu-2** OLED, the EQE was up to 16.57%. Finally, by combining the blue-light emitting Zn(ii) complex **Zn-1** and the orange Cu(i) complex **Cu-3**, we have demonstrated a white solution-processed OLED with maximum EQE of 6.88%, CIE coordinates of (0.42, 0.44) and colour rendering index of 81. To our best knowledge, this is the first example of high performance white solution-processed OLED fabricated solely with metal emitters of non-platinum group metals.

## Results and discussion

Chemical structures of **Zn-1–Zn-3** and **Cu-1–Cu-5** are depicted in Scheme 1. Details of their synthesis and characterization data are given in the ESI.† The Zn(ii) complexes were prepared by refluxing 4-substituted-1*H*-pyrrolo[2,3-*b*]pyridines with triethylamine in methanol, followed by the addition of a methanolic solution of zinc(ii) salt. The crude product was a white/yellow solid. It was purified by dissolving it in CH_2_Cl_2_ which was filtered through Celite and pre-concentrated. A white/yellow solid was obtained by precipitation with methanol followed by centrifugation and dried under vacuum. Attempts to grow crystals of **Zn-1–Zn-3** with suitable quality for single-crystal X-ray diffraction analysis were not successful. In view of the resemblance of the coordination mode of substituted 7-azaindolate with the parent 7-azaindolate, it is reasonable to propose an isostructural relationship among the Zn_4_O(AID)_6_ and **Zn-1–Zn-3** complexes. A previous study had disclosed the crystal structure of Zn_4_O(AID)_6_.^9*b*^ To cautiously confirm the reported structural description, as depicted in Fig. S60 (ESI†), we re-examined the crystal structure of Zn_4_O(AID)_6_ which was prepared following the same synthetic procedure as **Zn-1–Zn-3**. Crystals of Zn_4_O(AID)_6_ with quality suitable for structure determination by X-ray diffraction analysis were obtained by slow diffusion of diethyl ether into a CH_2_Cl_2_ solution of Zn_4_O(AID)_6_. The [Zn_4_O] core has a central oxygen atom surrounded by four Zn atoms in tetrahedral geometry. Each 7-azaindolate ion serves as a bridging ligand connecting two Zn(ii) ions *via* coordination to the nitrogen atoms of the pyrrole and pyridine moieties, respectively. The current structural solution (CCDC deposition number ; 1054456) provides a more chemically rational model than the previously reported one. In the previously reported structure, the lattice solvent molecules were modelled as disordered CH_2_Cl_2_ and water. In this work, a disordered diethyl ether was found to be more sensible. Although crystals of **Zn-1–Zn-3** with quality suitable for single crystal X-ray diffraction analysis have not been obtained, the Zn_4_O(L)_6_ (L = substituted 7-azaindolate ligand) core structures of **Zn-1–Zn-3** could be inferred by mass spectrometry and NMR experiments. The ^13^C NMR and ^1^H–^13^C HSQC NMR spectra of **Zn-1–Zn-3** show multiplet signals corresponding to the seven carbon atoms of the 7-azaindole core of each 4-substituted-1*H*-pyrrolo[2,3-*b*]pyridine at *δ* ∼100, ∼112, ∼123, ∼138, ∼143 and ∼156 ppm, respectively, and three of these multiplet signals could be assigned to three quaternary carbons on the 7-azaindole core. The multiplet signals between *δ* ∼126 and ∼137 ppm correspond to the carbon atoms of the 4-substitution of the 4-substituted-1*H*-pyrrolo[2,3-*b*]pyridine ligand. Complexes **Zn-1–Zn-3** are thermally stable with high decomposition temperature (*T*_d_ corresponds to 5% weight loss in thermogravimetric analysis (TGA) measurement) of 369–440 °C (Fig. S1 in ESI†).

**Fig. 1:**
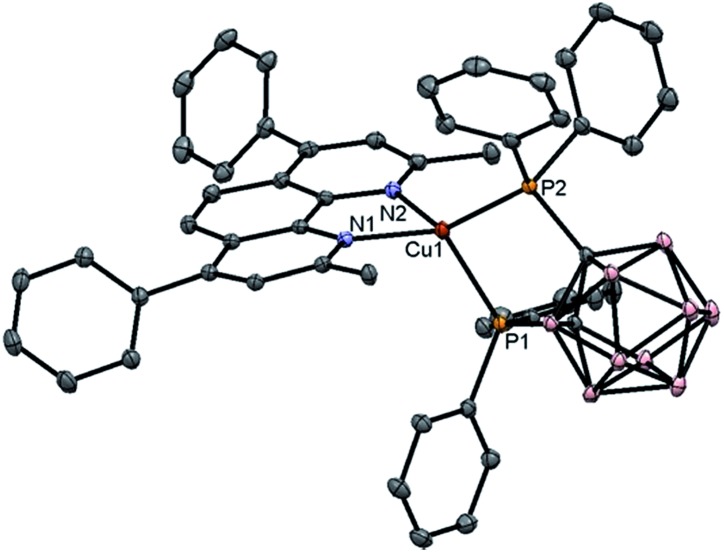
X-Ray crystal structure of **Cu-3**. Hydrogen atoms and solvent molecules are omitted for clarity.

The Cu(i) complexes were prepared by reacting [Cu(MeCN)_4_]PF_6_ with tetramethylammonium 7,8-bis(diphenylphosphino)-7,8-dicarba-*nido*-undecaborate ([NMe_4_][(PPh_2_)_2_C_2_B_9_H_10_]) in ethanol followed by addition of phenanthroline or bipyridine ligand.^14^ The crude product appeared as a yellow/orange solid and was purified by column chromatography on silica gel column with CH_2_Cl_2_ as eluent. Both ^1^H NMR spectra of **Cu1** and **Cu4** show doublet signals at ∼6.4 and ∼9.9 ppm that can be assigned to the protons at 2- and 9-position of the phenanthroline ligand. X-Ray crystal structures of **Cu-1** and **Cu-3** are determined in this work (Fig. S61 in ESI† and 1) while that of **Cu-2** and **Cu-4** have been reported.^14^ The crystals of **Cu-1** and **Cu-3** were obtained by diffusing diethyl ether into CH_2_Cl_2_ solutions of the corresponding copper complex. The crystal structure of **Cu-3** is shown in Fig. 1. **Cu-3** adopts a distorted tetrahedral geometry with N–Cu–N and P–Cu–P angles of 80.85 and 91.47°, respectively. The lengths of N–Cu and P–Cu bonds are 2.074–2.078 and 2.249–2.264 Å, respectively, similar to those found in Cu(i) complexes bearing diimine and diphosphine ligands.^11e,14^

### Photophysical properties and TRE measurements

**Fig. 2:**
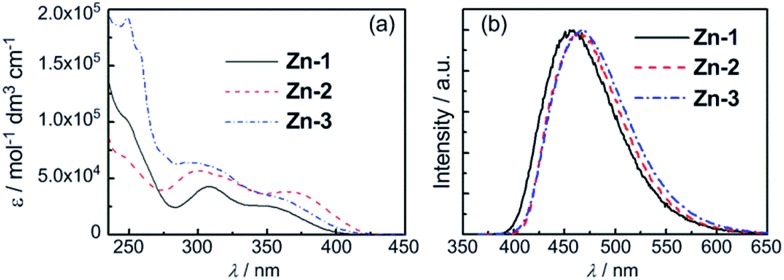
(a) UV/Vis absorption spectra of **Zn-1–Zn-3** in degassed CH_2_Cl_2_ (1 × 10^–5^ mol dm^–3^). (b) Emission spectra of **Zn-1–Zn-3** in degassed CH_2_Cl_2_ (2 × 10^–5^ mol dm^–3^) at room temperature.

Photophysical properties of **Zn-1–Zn-3** in solutions, thin film, and the solid state are summarized in Table 1. Complexes **Zn-1–Zn-3** show an intense absorption band at 299–308 nm (*ε* = 4.3–6.4 × 10^4^ dm^3^ mol^–1^ cm^–1^) and a broad shoulder at 350–368 nm (*ε* = 2.6–3.8 × 10^4^ dm^3^ mol^–1^ cm^–1^) in CH_2_Cl_2_ as depicted in Fig. 2a. The band at 299–308 nm is similarly present in the free ligand but not in [Zn_4_O(O_2_CMe)_6_],^9*b*^ hence is assigned to intraligand (IL) ^1^π → π* transition. The shoulder at 350–368 nm is attributed to a metal perturbed IL ^1^π → π* transition. No significant shift (5–11 nm) in absorption peak maxima is observed for **Zn-1** in MeCN, CH_2_Cl_2_, DMF, THF and toluene (Fig. S2, ESI†).

**Table 1:** Photophysical data of Zn(ii) and Cu(i) complexes

Complex	Medium	UV/Vis absorption, *λ*_abs_/nm (*ε*/dm^3^ mol^–1^ cm^–1^)	Emission		
			*λ*_max_/nm	*τ* ^*a*^/μs	*Φ* ^*b*^
**Zn-1**	CH_2_Cl_2_	248 (102 600), 308 (42 900), 350 (25 500)	455	0.015	0.66
	Toluene	303 (58 800), 351 (16 200)	455	0.015	0.17
	THF	303 (63 300), 356 (7700)	457	0.016	0.26
	MeCN	307 (47 700), 345 (29 600)	461	0.015	0.48
	DMF	306 (54 200), 356 (16 000)	465	0.015	0.55
	Solid (RT)		469		0.24
	Solid (77 K)		470		0.26
	Glassy (2-MeTHF, 77 K)		385, 468, 502		
	Thin film (5% in PMMA)		450		0.96

**Zn-2**	CH_2_Cl_2_	244 (71 000), 302 (56 900), 368 (37 900)	466	0.014	0.59
	Solid (RT)		499		
	Solid (77 K)		501		
	Glassy (2-MeTHF, 77 K)		390		
	Thin film (5% in PMMA)		475		0.71

**Zn-3**	CH_2_Cl_2_	249 (192 800), 299 (63 800), 355 (34 100)	467	0.008	0.45
	Solid (298 K)		491		
	Solid (77 K)		483		
	Glassy (77 K)		386, 498		
	Thin film (5% in PMMA)		460		0.78

**Cu-1**	CH_2_Cl_2_	271 (32 090), 322 (9430), 465 (5360)	Non-emissive	—	—
	Solid (298 K)		600	1.8	0.018
	Solid (77 K)		633	37.3	
	Glassy (77 K)		610	170	

**Cu-2**	CH_2_Cl_2_	274 (28 340), 438 (3030)	592	1.2	0.05
	Solid (298 K)		537	8.7	0.34
	Solid (77 K)		580	569	0.109
	Glassy (77 K)		550	1509	
	Thin film (5% in PMMA)		559		0.20
	Thin film (5% in PYD2)		368^*c*^, 558		0.30

**Cu-3**	CH_2_Cl_2_	285 (33 360), 448 (4210)	602	1.3	0.049
	Benzene	291 (19 270), 461 (3640)	550	<0.2	0.095
	THF	288 (42 460), 458 (3040)	605	1.6	<0.01
	Chloroform	287 (31 910), 457 (2150)	607	2.0	0.017
	Ethyl acetate	284 (34 730), 458 (3060)	610	<0.2	<0.01
	DMF	285 (38 110), 446 (4580)	Non-emissive	—	—
	Solid (298 K )		570	5.3	0.155
	Solid (77 K)		591	859	0.337
	Glassy (2-MeTHF, 77 K)		588	1271	0.62
	Thin film (5% in PMMA)		570	9.9	0.22
	Thin film (5% in PYD2)		562	14.6	0.60

**Cu-4**	CH_2_Cl_2_	285 (42 560), 473 (6900)	Non-emissive	—	—
	Solid (298 K)		632	1.5	<0.01
	Solid (77 K)		670	29.7	
	Glassy (77 K)		620	265	

**Cu-5**	CH_2_Cl_2_	305 (20 070), 434 (3250)	Non-emissive	—	—
	Solid (298 K)		552	6.2	0.266
	Solid (77 K)		598	208	0.105
	Thin film (5% in PMMA)		573		0.27
	Thin film (5% in PYD2)^*c*^		368^*c*^		0.042

^*a*^Emission lifetime.

^*b*^Emission quantum efficiency; no distinct variation in emission quantum yields of different batch of samples of the Zn(ii) complexes from independent preparation was observed.

^*c*^Incomplete energy transfer.

Emission spectra of **Zn-1–Zn-3** with concentrations at 10^–5^ M in degassed CH_2_Cl_2_ solutions are depicted in Fig. 2b. An intense emission with *λ*_max_ at 455–467 nm (*Φ* = 0.45–0.66; *τ* = 8–15 ns) is observed upon excitation of the Zn(ii) complexes at ∼350 nm. No distinct variation in emission quantum yields of different batches of samples of the Zn(ii) complexes from independent preparation was observed. Femtosecond time-resolved fluorescence (fs-TRF) measurements with excitation wavelength at 350 nm were undertaken. The TRF spectra and related fluorescence decay profiles of **Zn-1–Zn-3** are shown in Fig. S10 (ESI†). Comparison of TRF and steady-state emission spectra recorded in the same solvent system revealed that TRF spectra of **Zn-1–Zn-3** with decay lifetimes of 8–15 ns closely resemble the corresponding steady-state emission spectra. Steady-state emissions of **Zn-1–Zn-3** are attributed to fluorescence from the lowest singlet excited state (S_1_). Photophysical properties of Cu(i) complexes **Cu-1–Cu-5** are given in Table 1. **Cu-1–Cu-5** show strong absorption bands at 271–305 nm (*ε* = 2.0–4.3 × 10^4^ dm^3^ mol^–1^ cm^–1^) and 438–473 nm (*ε* = 0.3–0.69 × 10^4^ dm^3^ mol^–1^ cm^–1^) in CH_2_Cl_2_ as depicted in Fig. S6.† The low energy absorption band of **Cu-3** at *λ*_max_ = 446 nm in DMF is red-shifted to *λ*_max_ = 461 nm in the less polar solvent, benzene (Fig. S8a†). Complexes **Cu-1–Cu-5** are non-emissive or weakly emissive with quantum yields of lower than 0.1 in dilute solutions. However, high emission quantum efficiency of up to 0.62 has been observed with **Cu-3** in PYD2 thin film at room temperature and in 2-MeTHF glassy solution at 77 K. The solid-state emission spectra of **Cu-1–Cu-5** are depicted in Fig. S7.†

**Fig. 3:**
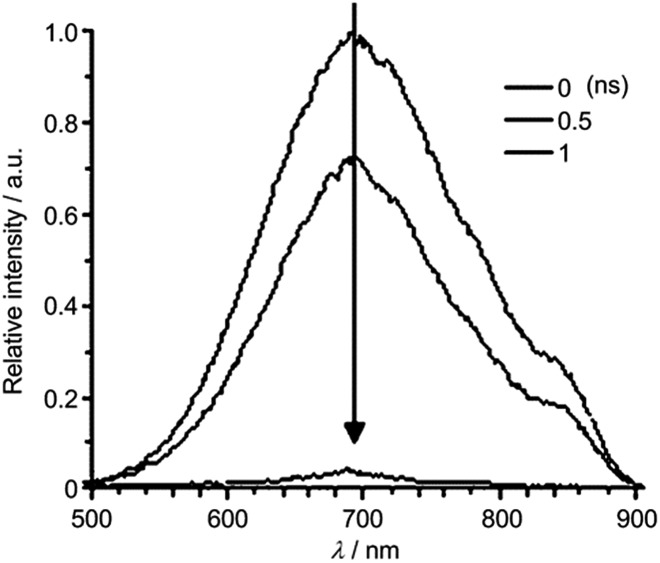
ns-TRE of **Cu-1** in CH_2_Cl_2_ recorded at indicated time intervals after excitation at 350 nm.

**Fig. 4:**
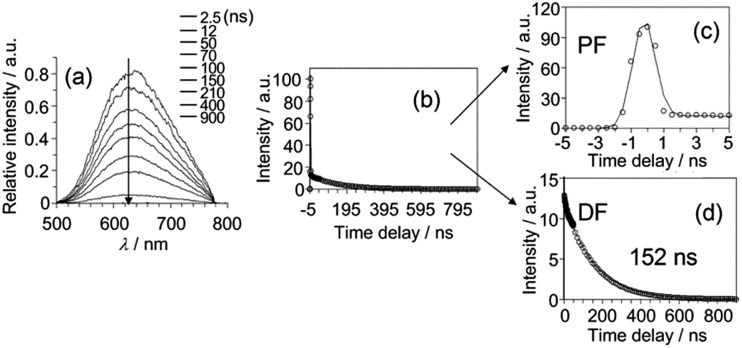
(a) ns-TRE of **Cu-2** in CH_2_Cl_2_ recorded at indicated time intervals after excitation at 350 nm. (b)–(d) ns-TRE decay profile of **Cu-2** in CH_2_Cl_2_ recorded at 350 nm.

To examine the origin of the emission from these Cu(i) complexes, nanosecond TRE (ns-TRE) measurements on **Cu-1**, **Cu-2** and **Cu-3** in CH_2_Cl_2_ were performed. The results are, respectively, depicted in Fig. 3, 4 and S11.† An emission with *λ*_max_ ∼ 690 nm is observed for **Cu-1**. This emission is short-lived and decays rapidly in time scale faster than the time resolution of TRE measurement (∼2 ns). Therefore, this emission is attributable to prompt fluorescence from the S_1_ singlet excited state. Of note, there is a massive red-shift in energy (∼7000 cm^–1^) of the fluorescence from the absorption (*λ*_max_ ∼ 465 nm) spectrum, suggesting large structural distortion of the emissive excited state from the ground state.^15^ The massive excited state structural distortion facilitates non-radiative decay, which may account for the weakly emissive nature of this complex in solutions at room temperature.

The emission *λ*_max_ of **Cu-2** and **Cu-3** (Fig. 4a and S11a†) are at ∼620 and ∼640 nm, respectively. Unlike that of **Cu-1**, these emissions decay in time scales of hundreds of nanoseconds to tens of microseconds. Analysis of the decay kinetics of emission intensity (measured by the integrated area of transient emission spectra) revealed a bi-exponential dynamics (Fig. 4b and S11b†). The first decay component (Fig. 4c and S11c†) has time constant faster than the time resolution of the TRE measurement and the second component (Fig. 4d and S11d†) has a lifetime of ∼152 ns and ∼210 ns for **Cu-2** and **Cu-3** in the open air condition, respectively. A decay lifetime of ∼1.27 μs is observed for the emission of **Cu-3** in the deoxygenated condition by purging the sample solution with nitrogen. For **Cu-2** or **Cu-3**, the time-resolved emission spectra recorded at different time intervals are similar. The first component, owing to its very fast decay time, can be attributed to prompt fluorescence from the S_1_ singlet excited state; the second component with lifetime of hundreds of nanoseconds for **Cu-2** and **Cu-3** in air and ∼1.27 μs for **Cu-3** in the deoxygenated condition is tentatively attributed to delayed fluorescence (DF) also from the S_1_ state produced through thermally activated conversion from closely lying emissive triplet state. The much longer lifetime of DF under the deoxygenated condition is because the lifetime of DF is defined by the lifetime of the triplet state which is known to be much longer-lived in deoxygenated condition due to elimination of the oxygen quenching.

**Fig. 5:**
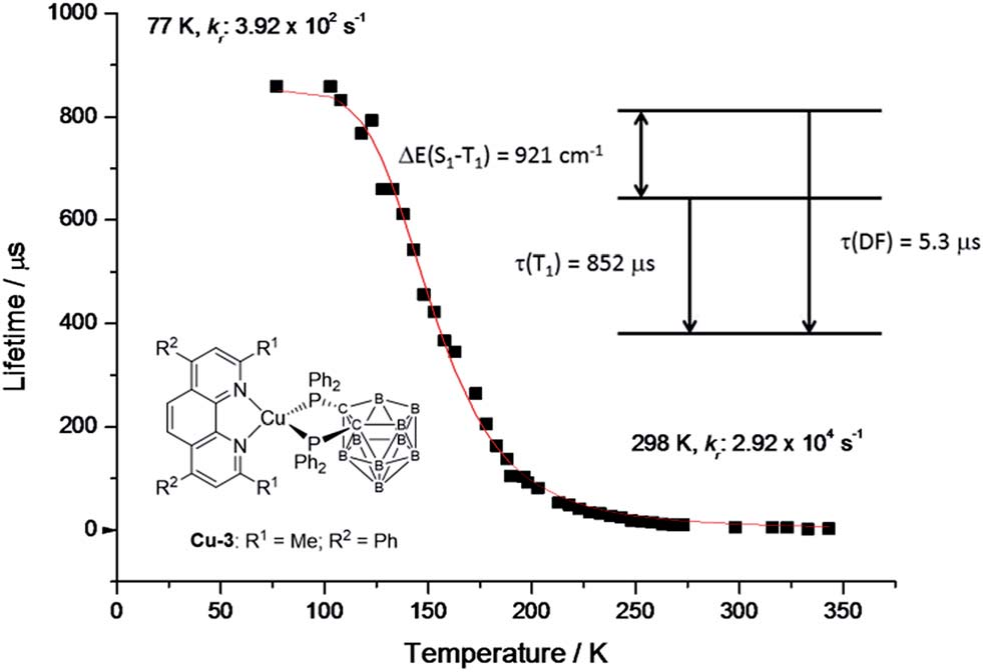
Emission decay time of **Cu-3** powder *vs*. temperature. The red line represents a fit of eqn (1) to the experimental data with phosphorescence decay time *τ*(T_1_) = 852 μs measured at 77 K. The resulting fit parameters are Δ*E*(S_1_ – T_1_) = 921 cm^–1^ and *τ*(S_1_) = 0.05 μs. The spontaneous fluorescence is not observed directly due to much faster intersystem crossing. *τ*(DF) = 5.3 μs is the decay time of the delayed fluorescence at ambient temperature.

To support the delayed fluorescence assignment, emission lifetimes at various temperatures have been recorded for **Cu-2**, **Cu-3** and **Cu-5**; the results are shown in Fig. S13,† 5 and S14,† respectively. With decreasing temperature, the decay time of **Cu-3** steadily increases before 200 K; there is a sharp increase in decay time at around 200 K to around 100 K, and then the decay time remains relatively the same (Fig. 5). This finding could be accounted for by the drastic decrease of radiative decay rate (*k*_r_) from 2.92 × 10^4^ s^–1^ at 298 K to 3.92 × 10^2^ s^–1^ at 77 K. Similar finding was obtained for **Cu-2** and **Cu-5**. The present emission spectral and decay dynamics data are compatible with the mechanism proposed by Yersin and co-workers in that DF is formed due to thermally activated conversion from the triplet state lying close in energy to the S_1_ singlet.^11*f*^ With reference to literature, the relationship between the change of emission decay time and temperature can be expressed as eqn (1):^11*e*^
1
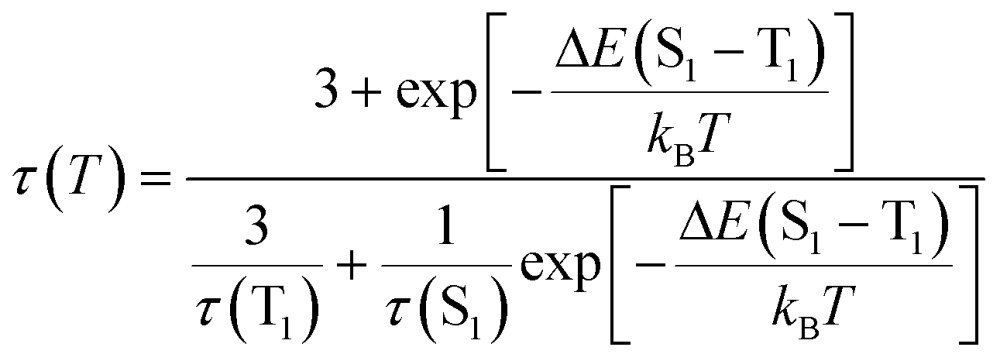



By fitting the experimental data, Δ*E*(S_1_ – T_1_), *τ*(S_1_) and *τ*(T_1_) of **Cu-3** have been found to be 921 cm^–1^, 0.04 μs and 852 μs, respectively (the corresponding values for **Cu-2** and **Cu-5** are depicted in Fig. S13 and S14†).

### Electrochemical properties of **Zn-1–Zn-3** and **Cu-1–Cu-5**

Electrochemical properties of **Zn-1–Zn-3** and **Cu-1–Cu-5** were studied by cyclic voltammetry. For **Zn-1–Zn-3**, in the anodic scan, there is one irreversible wave at 0.87–0.89 V *vs.* FeCp_2_^0/+^. This wave is attributed to the ligand oxidation. No reduction peak is observed in the cathodic scan up to –2.2 V. The HOMO levels for **Zn-1–Zn-3** were estimated to be –5.52 to –5.56 eV (Fig. S15–S17, ESI†). All Cu(i) complexes show an irreversible reduction wave at –2.15 to –2.48 V *vs.* FeCp_2_^0/+^ assigned to the reduction at phenanthroline/bipyridine ligand. For **Cu-1** and **Cu-4**, they show three quasi-reversible/irreversible oxidation waves at 0.48–0.49, 0.74–0.77 and 1.14–1.17 V whereas **Cu-2**, **Cu-3** and **Cu-5**, which have methyl groups on the 2-, 9-position of phenanthroline/6-, 6′-position of bipyridine ligand, show two irreversible oxidation waves at 0.50–0.55 and 0.95–0.97 V *vs.* FeCp_2_^0/+^. For all Cu(i) complexes, the first oxidation wave is assigned as oxidation of Cu(i) and the second to oxidation localized on the carborane ligand. The significantly different oxidation behaviour between **Cu-1**, **Cu-4** and **Cu-2**, **Cu-3**, **Cu-5** may be due to the absence of methyl groups at 2-, 9-position of phenanthroline ligand in the cases of **Cu-1** and **Cu-4** which allows the complex to undergo structural distortion upon oxidation, whereas the steric hindrance offered by methyl groups on the diimine ligand of **Cu-2**, **Cu-3** and **Cu-5** inhibits such conformation change and may lead to ligand dissociation during oxidation.

### DFT calculations

**Fig. 6:**
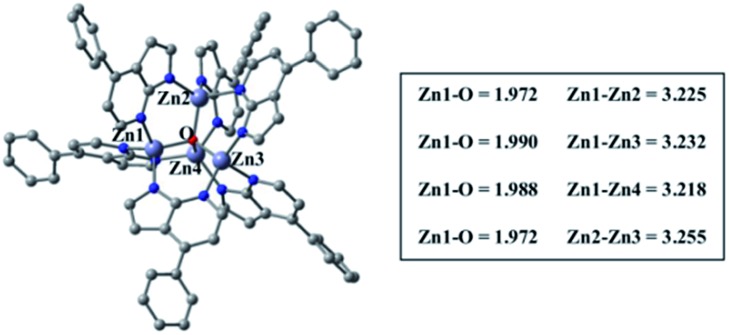
Optimized ground-state (S_0_) geometry of **Zn-1** by using PBE0/6-31G*(LANL2DZ).

**Fig. 7:**
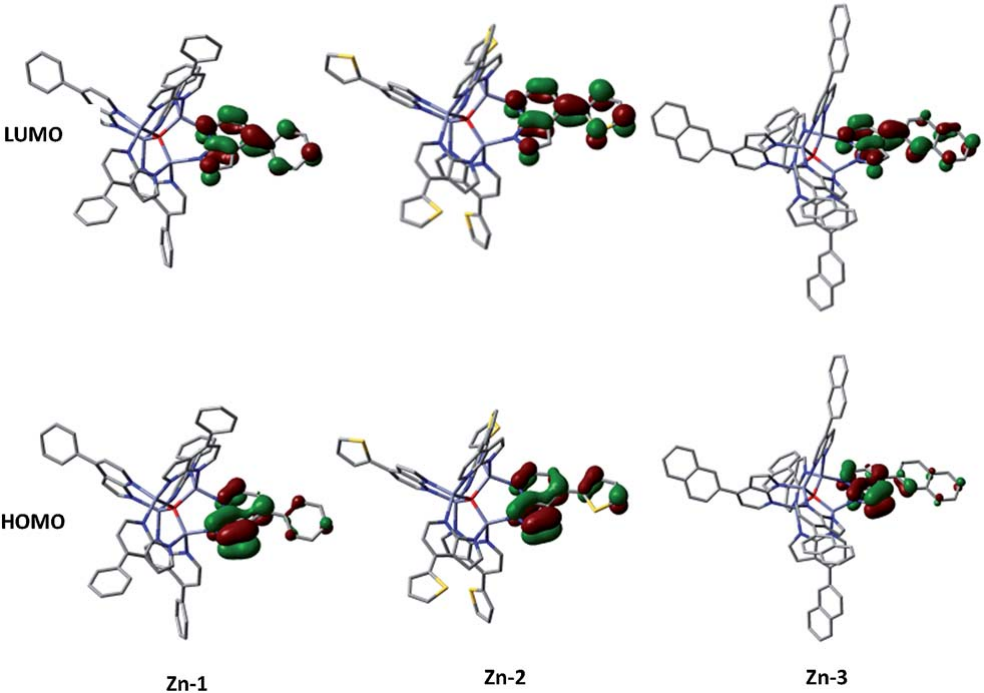
Computed surfaces of HOMO and LUMO of **Zn-1–Zn-3** (isovalue = 0.05).

Density functional theory (DFT) and time-dependent DFT (TDDFT) calculations were performed to understand the electronic structures of **Zn-1–Zn-3** and **Cu-1–Cu-5** using Gaussian 09 package^16^ (for details, see the ESI†). Fig. 6 depicts the optimized structure of **Zn-1**. The calculated Zn···Zn distances of 3.218–3.255 Å and Zn–O distances of 1.972–1.990 Å are similar to related values reported for [Zn_4_O(AID)_6_] (Zn–O 1.903–1.975 Å, Zn···Zn 3.147–3.209 Å).^9*b*^ The emission data and the HOMO/LUMO surfaces of **Zn-1–Zn-3** are listed in Table 2 and Fig. 7. The emission of each complex originates from HOMO → LUMO (96%) with oscillator strength of 0.3103, 0.4513 and 0.4377, respectively. As shown in Fig. 7, each emission is intraligand charge transfer (ILCT) in nature because the HOMO and LUMO of each complex come from the same ligand. The calculated emission wavelength is 430, 441 and 448 nm for **Zn-1**, **Zn-2** and **Zn-3**, respectively, which is in good agreement with corresponding experimental emission *λ*_max_ values.

**Table 2:** Emission data of **Zn-1–Zn-3**

	Emission/nm		Oscillator strength (*f*)	Transition nature
	Calc.	Expt.		
**Zn-1**	430	455	0.3103	HOMO → LUMO (96%) ILCT
**Zn-2**	441	466	0.4513	HOMO → LUMO (96%) ILCT
**Zn-3**	448	467	0.4377	HOMO → LUMO (96%) ILCT

Table 3 depicts the calculated structures of **Cu-1–Cu-5**. The averaged bond distances of Cu–P and Cu–N are 2.33 and 2.13 Å, respectively, which are slightly longer than the corresponding experimental parameters of **Cu-1** and **Cu-3** (averaged Cu–P = 2.25 Å and Cu–N = 2.08 Å). The TDDFT calculated transition of HOMO → LUMO for **Cu-1–Cu-5** is at 469, 447, 458, 481 and 445 nm, respectively, which is in good agreement with the corresponding experimental absorption *λ*_max_ values of 465, 438, 448, 473 and 434 nm. The electronic transition is metal-to-ligand charge transfer (MLCT) in nature because the HOMO is mainly localized at the Cu atom and LUMO is mainly localized on the ligand.

**Table 3:** The calculated structural parameters (Å) of the copper(i) complexes (**Cu-1–Cu-5**) in ground state (S_0_)

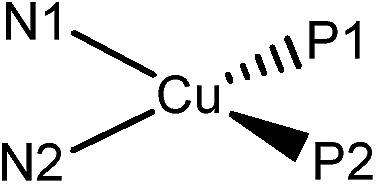	Cu–P1	Cu–P2	Cu–N1	Cu–N2
**Cu-1**	2.315	2.306	2.125	2.104
**Cu-2**	2.355	2.347	2.154	2.148
**Cu-3**	2.351	2.349	2.140	2.146
**Cu-4**	2.307	2.319	2.101	2.114
**Cu-5**	2.362	2.353	2.150	2.147

As shown in Table 4, the calculated emission (fluorescence) *λ*_max_ values of **Cu-2** and **Cu-3** are 599 and 611 nm, respectively, which match very well with the experimental emission peaks of **Cu-2** (592 nm) and **Cu-3** (602 nm). The calculated energy gaps (Δ*E*(S_1_ – T_1_)) between singlet excited state (S_1_) and triplet excited (T_1_) are, respectively 1855 and 1129 cm^–1^ for **Cu-2** and **Cu-3**, which are comparable to the Δ*E*(S_1_ – T_1_) = 1300 cm^–1^ similarly calculated for Cu(POP)(pz_2_BH_2_)^11*d*^ (Δ*E*(S_1_ – T_1_) = 1300 cm^–1^) which displays a thermally activated delayed fluorescence (TADF) from the lowest excited singlet state (S_1_) at room temperature. The analysis of the emission decay lifetime *versus* temperature reveals the gap (Δ*E*(S_1_ – T_1_)) for **Cu-3** to be 921 cm^–1^ (see Fig. 5) which is 208 cm^–1^ (∼0.6 kcal mol^–1^) lower than the TDDFT calculated value (1129 cm^–1^). To test the validity of the calculated value, we did similar TDDFT calculation on the **Cu(POP)-2** having a well-defined TADF property.^11*e*^ The calculated emission of **Cu(POP)-2** is 561 nm at S_1_ excited state, which is in good agreement with the experimental emission data (555 nm); the calculated Δ*E*(S_1_ – T_1_) value is 1432 cm^–1^, which is 712 cm^–1^ (2.0 kcal mol^–1^) larger than the experimental value of 720 cm^–1^.^11*e*^ For **Cu-2** and **Cu-5**, the calculated Δ*E*(S_1_ – T_1_) value is 660 and 687 cm^–1^ larger than the experimental values, respectively. Since both the emissions of S_1_ and T_1_ were based on the optimized geometries in TDDFT calculation, the discrepancy between the experimental and calculated Δ*E*(S_1_ – T_1_) values might be due to the lack of the zero point energy correction of the excited states (S_1_/T_1_). To obtain the zero point energy, one needs to perform the frequency calculation in the excited state (S_1_/T_1_), which is highly demanding in the context of computational resources and beyond the scope of this work.

**Table 4:** Emission data of **Cu-1–Cu-5**

	S_1_	T_1_	Δ*E*(S_1_ – T_1_)	Expt^*a*^
**Cu-1**	934 nm, H → L (98.0%), *f* = 0.03	989 nm, H → L (96.4%)	N.A.	Non-emissive
**Cu-2**	599 nm, H → L (97.3%), *f* = 0.10	673 nm, H → L (95.2%)	1855 cm^–1^	592 nm
**Cu-3**	611 nm, H → L (97.0%), *f* = 0.13	655 nm, H → L (94.7%)	1129 cm^–1^	602 nm
**Cu-4**	968 nm, H → L (98.0%), *f* = 0.03	1031 nm, H → L (96.5%)	N.A.	Non-emissive
**Cu-5**	643 nm, H → L (96.9%), *f* = 0.05	707 nm, H → L (95.2%)	1428 cm^–1^	Non-emissive

^*a*^Emissive spectra are measured in CH_2_Cl_2_ solution at 298 K (conc. 2 × 10^–5^ mol dm^–3^).

It is informative to compare the calculated geometrical changes between the ground (S_0_) and the excited states (S_1_/T_1_) as depicted in Table 5. The P2–N2–N1–P1 dihedral angles of all the complexes in the ground state (S_0_) are quite similar (52.3–52.8°). In excited states, however, the geometrical changes are quite different. For **Cu-1** and **Cu-4**, the dihedral angle of P2–N2–N1–P1 changes dramatically in excited state (Δ(S_0_ – S_1_) = 20.2 and 19.8; Δ(S_0_ – T_1_) = 12.8 and 12.7). While for **Cu-2**, **Cu-3** and **Cu-5**, their geometries are quite rigid in all states (Δ(S_0_ – S_1_) = 3.4, 3.3 and 2.8; Δ(S_0_ – T_1_) = 2.6, 1.1 and –0.2). The smaller geometrical changes are related to smaller Huang–Rhys factor (*S*_M_), which leads to smaller non-radiative decay constant and thus higher emission quantum yield. This is consistent with the experimental findings that, in the solid state at room temperature, **Cu-1** and **Cu-4** are weakly emissive with quantum yield of 0.01–0.018 while **Cu-2**, **Cu-3** and **Cu-5** are strongly emissive with quantum yield of 0.155–0.34 (see Table 1).

**Table 5:** Comparison of the geometries in ground state (S_0_) and excited states (S_1_/T_1_)

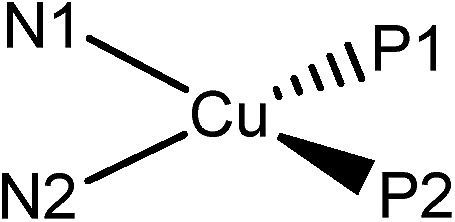	Dihedral angle P2–N2–N1–P1/°		
	S_0_	S_1_	T_1_
Cu-1	52.8	32.6	40.0
Cu-2	52.4	49.0	49.8
Cu-3	52.3	49.0	51.2
Cu-4	52.4	32.6	39.7
Cu-5	52.3	49.5	52.5

The geometrical changes in excited state may also correlate to the emission energy. **Cu-1** and **Cu-2** were selected for comparison. Emissions of both **Cu-1** and **Cu-2** originate from HOMO → LUMO (over 97%). The frontier molecular orbitals (FMOs) of **Cu-1** and **Cu-2** in the ground state and singlet excited state are shown in Fig. S62 and S63.† In the ground state (S_0_), both **Cu-1** and **Cu-2** have similar HOMO–LUMO gap, which is in line with the similar UV-vis absorption spectra of the complexes. The excited states (S_1_) of **Cu-1** and **Cu-2**, because of their MLCT in nature, have the Cu atom in a formal d^9^ electronic configuration and then experience a noticeable Jahn–Teller effect, and the latter is the driving force for a more planar geometry in the excited state (smaller dihedral angle of P2–N2–N1–P1, see Table 5). The more planar geometry (smaller dihedral angle of P2–N2–N1–P1) in the excited state leads to a higher energy level of HOMO (stronger σ* antibonding between metal and the ligand). For **Cu-1**, its HOMO energy increases substantially due to the considerable geometrical distortion. As for **Cu-2**, the steric effect of the methyl group confines the geometrical distortion thus the energy level of HOMO does not increase too much. Thus, **Cu-2** has a higher energy emission than **Cu-1** due to the smaller geometrical distortion in the excited state. This is also in line with the experimental findings described in previous sections.

In summary of this section, the low energy intense absorption bands of the Cu(i) complexes studied herein are metal to ligand charge transfer (MLCT) in nature. Geometrical distortion in the excited state is balanced by both the Jahn–Teller effect and steric effect of the substituent methyl group in the cases of complexes **Cu-2**, **Cu-3** and **Cu-5**. Such geometrical distortion not only affects the non-radiative decay constant but also the emission energy. TADF is possible for **Cu-2**, **Cu-3** and **Cu-5** due to the small energy gap between first singlet and triplet excited state (Δ*E*(S_1_ – T_1_)).^11^

### Electroluminescent properties of **Zn-1–Zn-3**

**Fig. 8:**
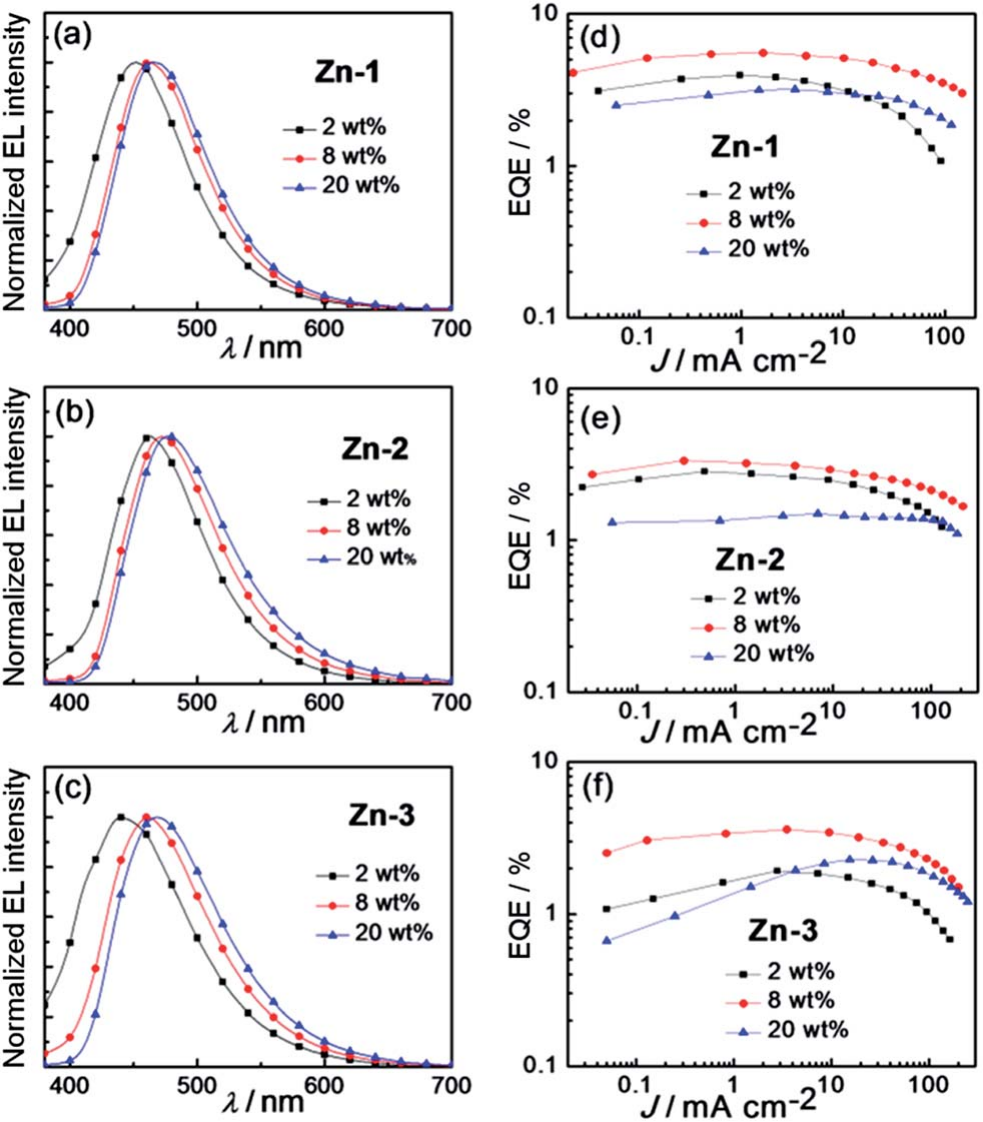
Normalized EL spectra of solution-processed OLEDs based on (a) **Zn-1**, (b) **Zn-2** and (c) **Zn-3** at different doping concentration. EQE–current density characteristics of devices based on (d) **Zn-1**, (e) **Zn-2** and (f) **Zn-3** at different doping concentrations.

Blue solution-processed OLEDs were fabricated with a simple architecture of ITO/PEDOT:PSS/PVK:OXD-7:Zn(ii) complex/TmPyPb/TPBi/LiF/Al. A mixture of PVK (polyvinylcarbazole) and OXD-7 [(1,3-bis[(4-*tert*-butylphenyl)-1,3,4-oxadiazolyl]phenylene)] with weight ratio of 90 : 5 was used as the host, TmPyPb [1,3,5-tri[(3-pyridyl)-phen-3-yl]benzene] as hole-blocking layer, and TPBi [2,2′,2′′-(1,3,5-benzinetriyl)tris(1-phenyl-1-*H*-benzimidazole)] as electron-transporting layer.^17^ Fig. 8a–c depict EL spectra of OLEDs fabricated with **Zn-1–Zn-3** at dopant concentrations of 2–20 wt%. Compared with photoluminescent (PL) spectra of thin film samples of **Zn-1–Zn-3** depicted in Fig. S5 (ESI†), EL spectra of corresponding devices at low dopant concentration of 2 wt% were slightly blue-shifted in energy. This could be explained by the overlapping of emissions originated from the PVK host and the Zn(ii)-dopant due to incomplete energy transfer from PVK to Zn(ii)-emitter at such low dopant concentration.^8g,17^ Consequently, as depicted in Fig. 8d–f, EQEs of devices with 2 wt% Zn(ii)-dopant were relatively low because of the low efficiency of PVK.^8*g*^ Upon increasing the doping concentration of Zn(ii)-emitter to 8 wt%, all EL spectra matched well to corresponding PL ones, suggesting efficient energy transfer from PVK to Zn(ii)-emitter. Thus, EQEs of all Zn-devices were improved in the cases of 8 wt% dopant concentration. Further increase in dopant concentration to 20 wt% led to a red shift in EL spectra of all devices; this could be the result of intermolecular interactions between the Zn(ii) complexes. At such high dopant concentration, self-quenching of the Zn(ii)-emitters became notable and therefore EQEs of all Zn(ii)-OLEDs at 20 wt% dopant concentration were low. Among the blue OLEDs studied in this work, the one with 8 wt% **Zn-1** showed the highest EQE of 5.55% and CE of 8.12 cd A^–1^ (Table 6) attributed to the high emission quantum yield (0.96) of **Zn-1** in the thin film (Table 1). These efficiency values are in general much higher than those of the devices fabricated with other blue luminescent Zn(ii) complexes.^9b–d,10a–f^ In addition, the EL maximum of the OLED with 8 wt% **Zn-1** was located at 462 nm with CIE coordinates of (0.16, 0.19), suggesting **Zn-1** could be a suitable blue emitter for white OLEDs.

**Table 6:** Key performance parameters of OLEDs with **Zn-1–Zn-3**

Complex (wt%)	L^*a*^/cd m^–2^	CE^*b*^/cd A^–1^	PE^*c*^/lm W^–1^	EQE^*d*^ (%)	CIE^*e*^ (*x*, *y*)
**Zn-1** (2%)	1080	4.29	2.31	3.94	(0.16, 0.15)
**Zn-1** (8%)	6503	8.12	4.28	5.55	(0.16, 0.19)
**Zn-1** (20%)	3480	5.12	2.45	3.18	(0.16, 0.21)
**Zn-2** (2%)	2290	4.14	2.37	2.83	(0.17, 0.20)
**Zn-2** (8%)	6300	6.00	3.14	3.33	(0.18, 0.26)
**Zn-2** (20%)	4095	3.00	1.50	1.49	(0.19, 0.30)
**Zn-3** (2%)	1240	2.14	1.12	1.92	(0.17, 0.16)
**Zn-3** (8%)	4650	5.57	3.00	3.61	(0.17, 0.21)
**Zn-3** (20%)	5430	4.12	1.88	2.29	(0.18, 0.25)

^*a*^Max. luminance.

^*b*^Max. current efficiency.

^*c*^Max. power efficiency.

^*d*^Max. external quantum efficiency.

^*e*^CIE coordinates at 100 cd m^–2^.

### Electroluminescent properties of **Cu-2**, **Cu-3** and **Cu-5**

**Fig. 9:**
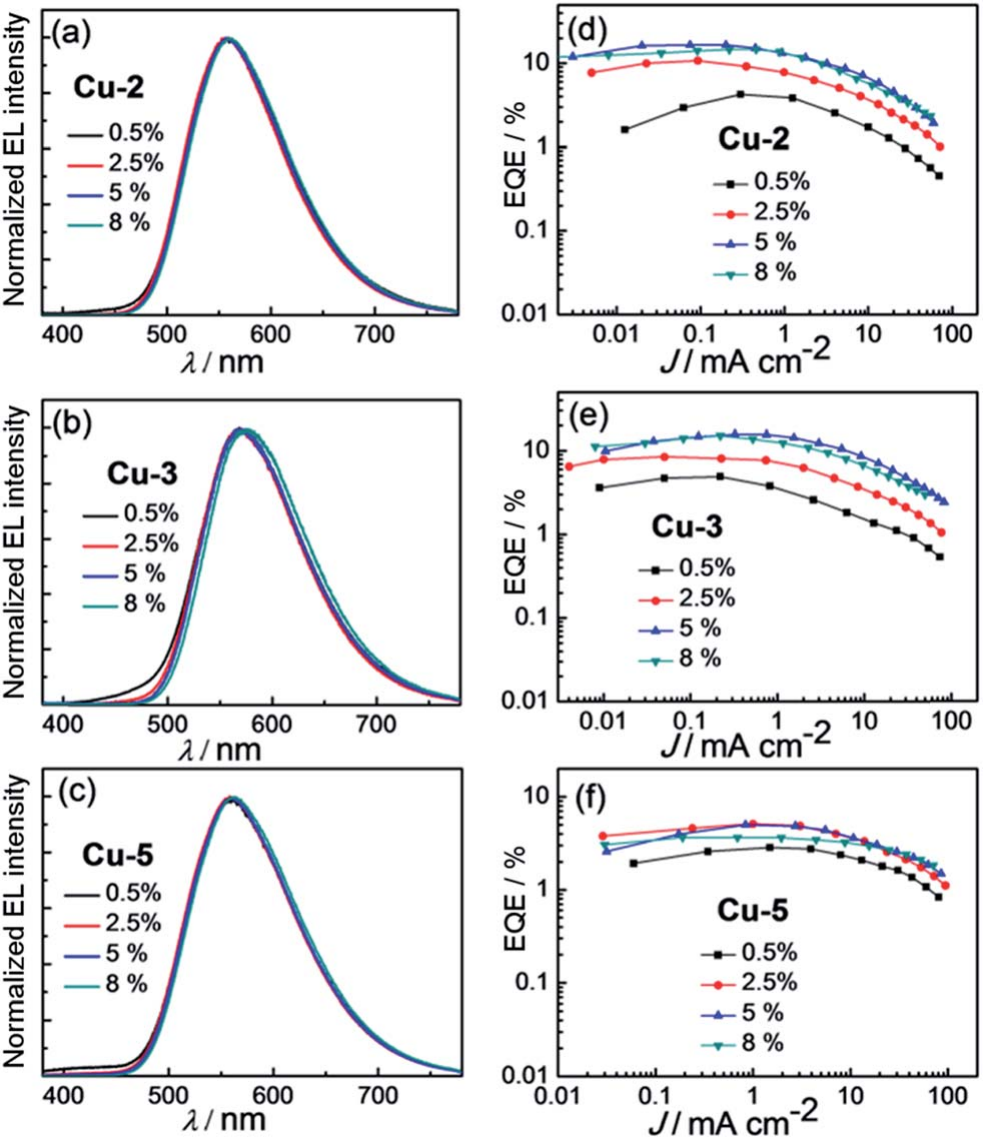
Normalized EL spectra of PYD2/DPEOP devices based on (a) **Cu-2**, (b) **Cu-3** and (c) **Cu-5** with different doping concentrations. EQE–current density characteristics of PYD2/DPEOP devices based on (d) **Cu-2**, (e) **Cu-3** and (f) **Cu-5** at different doping concentrations.

Since **Cu-1** and **Cu-4** are non-emissive in solutions and weakly emissive in the solid state (Table 1), only EL properties of **Cu-2**, **Cu-3** and **Cu-5** were examined. Solution-processed OLEDs with architecture of ITO/PEDOT:PSS/PYD2:Cu(i) emitter/DPEPO/TPBi/LiF/Al (PYD2/DPEPO device) were fabricated. In these devices, **Cu-2**, **Cu-3** or **Cu-5** was used as the emitter, PYD2 (2,6-dicarbazolo-1,5-pyridine) as the host, DPEPO [bis{2-[di(phenyl)phosphino]phenyl}ether oxide] as hole-blocking layer, and TPBi as electron-transporting layer. As depicted in Fig. 9 and Table 7, at low dopant concentration of 0.5 wt%, relatively lower efficiency of 4.25, 4.94 and 2.83% were recorded in **Cu-2**, **Cu-3** and **Cu-5** devices due to incomplete energy transfer from PYD2 to Cu(i) complex at such low dopant concentration. Upon increasing the Cu(i) dopant concentration beyond 0.5 wt%, energy transfer from PYD2 to these Cu(i) complexes became efficient and maximum EQEs of 16.57, 15.64 and 5.10%, corresponding to CEs of 49.80, 43.33 and 14.75 cd A^–1^ have been achieved for the OLEDs with 5 wt% **Cu-2**, 5 wt% **Cu-3** and 2.5 wt% **Cu-5**, respectively. The high EL efficiency of **Cu-2** and **Cu-3** devices is attributed to the emissions of **Cu-2** and **Cu-3** to be TADF instead of prompt fluorescence. It is worthy to note that high EQE was achieved in the **Cu-2** device in spite of the incomplete energy transfer from the host DPY2 to **Cu-2** displayed in the PL spectrum of **Cu-2** doped PYD thin film (Fig. S9a, ESI†), suggesting that trapping mechanism plays an important role in the emission of the **Cu-2** device. For the **Cu-5** device, on the other hand, energy transfer can hardly take place from PYD2 to **Cu-5** (Fig. S9b, ESI†). This could be the main reason for the relatively low efficiency of the **Cu-5** device.

**Table 7:** Key parameters of PYD2/DPEOP OLEDs with **Cu-2**, **Cu-3**, and **Cu-5**

Complex (wt%)	L^*a*^/cd m^–2^	CE^*b*^/cd A^–1^	PE^*c*^/lm W^–1^	EQE^*d*^ (%)	CIE^*e*^ (*x*, *y*)
**Cu-2** (0.5%)	1000	13.33	7.62	4.25	(0.43, 0.53)
**Cu-2** (2.5%)	2250	33.51	17.77	10.80	(0.44, 0.53)
**Cu-2** (5%)	3480	49.80	25.45	16.57	(0.44, 0.53)
**Cu-2** (8%)	3840	43.83	20.16	14.63	(0.44, 0.53)
**Cu-3** (0.5%)	1100	13.64	7.43	4.94	(0.45, 0.50)
**Cu-3** (2.5%)	2280	23.60	12.57	8.47	(0.47, 0.51)
**Cu-3** (5%)	5580	43.33	19.81	15.64	(0.48, 0.51)
**Cu-3** (8%)	4000	40.45	18.17	15.12	(0.49, 0.50)
**Cu-5** (0.5%)	1900	8.06	4.22	2.83	(0.43, 0.52)
**Cu-5** (2.5%)	3030	14.75	7.73	5.10	(0.44, 0.53)
**Cu-5** (5%)	3680	14.41	7.55	4.98	(0.44, 0.53)
**Cu-5** (8%)	3740	10.53	6.01	3.64	(0.45, 0.53)

^*a*^Max. luminance.

^*b*^Max. current efficiency.

^*c*^Max. power efficiency.

^*d*^Max. external quantum efficiency.

^*e*^CIE coordinates at 100 cd m^–2^.

### White OLEDs with luminescent Cu(i) and Zn(ii) complexes

**Fig. 10:**
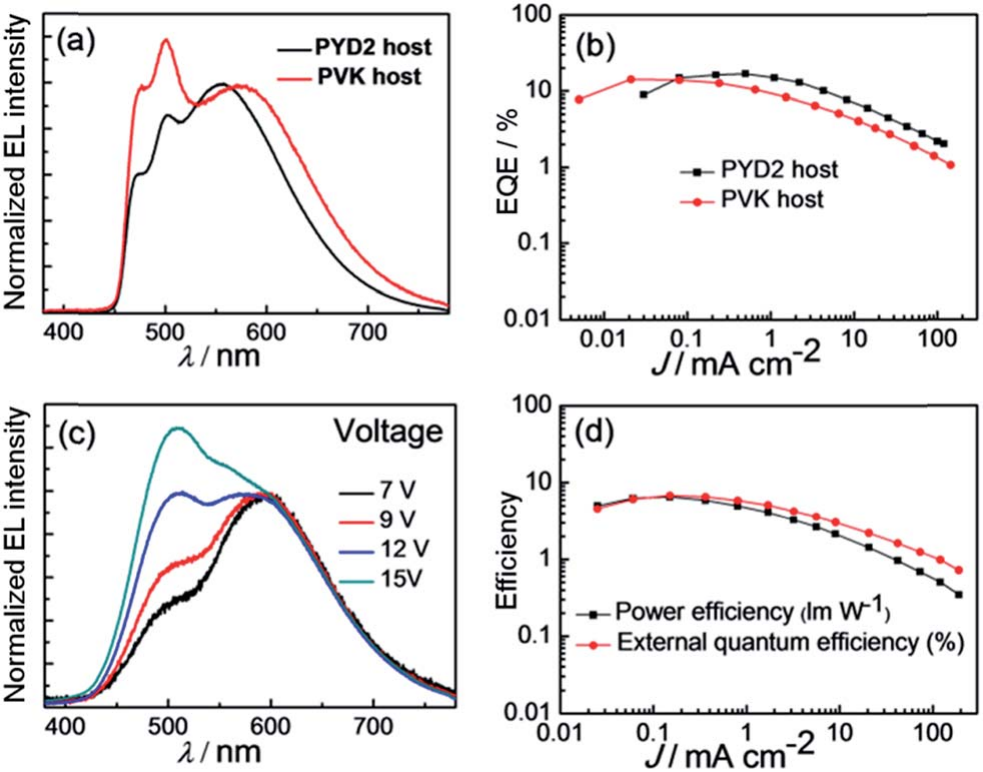
(a) Normalized EL spectra and (b) EQE–power efficiency–current density characteristics of the white OLED with 10 wt% FIrpic and 1 wt% **Cu-3** using PYD2/DPEPO or PVK/3TPYMB device structure. (c) EL spectra at different driving voltages and (d) EQE–power efficiency–current density characteristics of the white OLED with 10 wt% **Zn-1** and 1 wt% **Cu-3**.

Since EL maxima of PYD2/DPEPO devices with **Cu-2** and **Cu-3**, respectively, locate at 558 and 574 nm (see Fig. 9), **Cu-3** is more suitable as a long-wavelength emitter used in the fabrication of white-light devices. Thus, solution-processed OLED with the architecture of ITO/PEDOT:PSS/PYD2:FIrpic (10 wt%):**Cu-3** (1 wt%)/DPEPO/TPBi/LiF/Al was fabricated and characterized, in which bis[(4,6-difluorophenyl)pyridinato-*N*,*C* ^2^]-(picolinato)iridium (FIrpic)^5*c*^ and **Cu-3** were used as blue and yellow dopants, respectively. Although high EQE of 16.77% and power efficiency of 22.19 lm W^–1^ have been achieved with this [**Cu-3**, FIrpic] device, its CIE coordinates of (0.37, 0.48) and colour rendering index (CRI) of 61 are not satisfactory for lighting application. Generally, colour quality of a white OLED can be improved by covering a larger visible spectral region with its EL spectrum. It was reported that EL of the Cu(i) complex [Cu(dnbp)(DEPhos)]BF_4_ is strongly dependent on host and hole-blocking layer (HBL) materials used in the corresponding OLED.^13*c*^ Similarly, EL spectra of OLEDs with the architecture of ITO/PEDOT:PSS/PVK:OXD-7:**Cu-3**/3TPYMB/TPBi/LiF/Al (PVK/3TPYMB devices) were shifted to lower energy compared with that of PYD2/DPEPO as depicted in Fig. S25 (ESI†). By applying the PVK/3TPYMB device structure on the white OLED based on FIrpic and **Cu-3**, that is ITO/PEDOT:PSS/PVK:OXD-7:FIrpic (10 wt%):**Cu-3** (1 wt%)/3TPYMB/TPBi/LiF/Al, emission of **Cu-3** was red-shifted by ∼23 nm and therefore CRI of this white OLED was improved to 71 with CIE coordinates of (0.37, 0.45) as depicted in Fig. 10a. Since the EL efficiency of **Cu-3** was lower in the PVK/3TPYMB device structure (Table S1, ESI†), the maximum efficiency (14.27%) of the white OLED with PVK/3TPYMB structure was lower. For the same reason, emission ratio of **Cu-3**/FIrpic was also lower in this device as depicted in Fig. 10a. The PVK/3TPYMB structure was also used to fabricate white solution-processable OLEDs with solely first-row transition-metal complexes. The white device was constructed as ITO/PEDOT:PSS/PVK:OXD-7:**Zn-1** (10 wt%):**Cu-3** (1 wt%)/3TPYMB/TPBi/LiF/Al, in which **Zn-1** and **Cu-3** were used as blue and orange light-emitting materials, respectively.

Fig. 10c depicts the dependence of EL spectra for the white OLED on the driving voltages; at low voltage of 7 V, orange **Cu-3** emission dominated the EL spectrum, indicating energy transfer from **Zn-1** to **Cu-3**. Upon increasing the driving voltage, **Zn-1** emission increased and CIE coordinates were shifted from (0.42, 0.44) at 9 V to (0.35, 0.44) at 15 V. Nevertheless, the corresponding CRI only slightly decreased from 81 to 76 while the luminance of this device increased from 300 to 3150 cd m^–2^ as listed in Table S2 (ESI†). Power efficiency–EQE–current density characteristics of this OLED are depicted in Fig. 10d. Maximum EQE of 6.88%, corresponding to CE of 14.67 cd A^–1^, and power efficiency of 6.58 lm W^–1^ have been achieved. To our best knowledge, these performance data are the best among those of the reported white solution-processed OLEDs with fluorescent emitters.^4a–c^

Device stability of OLEDs with **Zn-1** (8 wt%), **Cu-3** (5 wt%), as well as that of the white OLED device with both **Zn-1** (10 wt%) and **Cu-3** (1 wt%), was investigated and the results are depicted in Fig. S27 (ESI†). The initial luminance for all devices was 200 cd m^–2^, and the half lifetimes (*T*_50_) were 18, 250 and 22 min for **Zn-1**, **Cu-3**, and white (**Zn-1** + **Cu-3**) OLEDs, respectively. These device lifetimes are not good for practical applications and could partly be attributed to the instability of the PVK host.^18^ Nevertheless, the device lifetime of the **Cu-3** device is comparable to the reported value of the OLED based on *fac*-tris(2-phenylpyridine)iridium doped in PVK host.^18*b*^

We conceived the possibility to develop blue and hence white solution-processed OLEDs with full Cu(i)-emitters. For this reason, we prepared the literature reported Cu(pop)(pz_2_Bph_2_) which is a blue emitter with *λ*_max_ at 464 nm and high PL efficiency of 90%;^11*d*^ we used **Cu-3** to be the orange emitter for the Cu-based white OLED. To fabricate such device, both Cu(i) complexes should be co-doped in a common host material, such as PYD-2. A solution-processed OLED with the architecture of ITO/PEDOT:PSS/PYD2:Cu(pop)(pz_2_Bph_2_) (10 wt%)/DPEPO/TPBi/LiF/Al was therefore investigated in order to test EL of Cu(pop)(pz_2_Bph_2_) in PYD-2 host. As depicted in Fig. S26,† the emission colour of this device was green instead of blue and the EQE was 3.2%. Thus the blue emission from Cu(pop)(pz_2_Bph_2_) can only be obtained in the powder state but not in thin doped film.

## General remarks

Phosphorescent metal complexes, such as that of Ir(iii) and Pt(ii), have been extensively investigated and used as emitters in high efficiency OLEDs.^1,2,19,20^ Nevertheless, strongly luminescent metal complexes from inexpensive earth-abundant metals should be appealing especially for low-cost solution-processed OLEDs. Because of the earth abundance and eco-friendly nature of zinc metal, Zn(ii) complexes are potentially useful light-emitting materials to realize production of low-price OLEDs in large scale. The photophysical and/or EL properties of several strongly blue luminescent Zn(ii) complexes had been reported in our previous works.^9b,21^ The new Zn(ii) complex **Zn-1** in this work has a high emission quantum yield of 0.96 (see Table 1) in thin PMMA film and was observed to deliver the highest EL efficiency of 5.55% (see Table 6) among those of blue OLEDs with other Zn(ii) complexes as light-emitting material.^10^

Recent works showed that Cu(i) complexes have the potential to be developed into efficient light-emitting materials for high performance OLEDs.^12,13^ Maximum EQEs of up to 21.3% and 15.0% for vacuum-deposited and solution-processed OLEDs, respectively, fabricated with luminescent Cu(i) complexes had been reported.^12b,13c^ The first report^22*a*^ on EL property of Cu(i) complexes appeared at almost the same time as that of Os(ii) and Pt(ii) complexes^23^ with the EL proposed to come from triplet excited state(s) (phosphorescence).^22^ Yersin and co-workers extensively developed the Cu(i) complexes which display TADF emission.^11^ Recently, Peters and co-workers demonstrated the DF mechanism to account for both PL and EL of the dinuclear Cu(i) complex supported by bis(phosphine)diarylamido ligand; this Cu(i) complex has a small S_1_ – T_1_ energy gap.^12*a*^ Adachi and co-workers reported the Cu(i) complexes [Cu(dnbp)(DPEPhos)]BF_4_ and [Cu(μI)dppb]_2_ to harvest both singlet and triplet excitons.^13*c*^ In the present work, PL of **Cu-2** and **Cu-3** has been studied by ns-TRE. Both the spectral evolution data and decay dynamics (see Fig. 4) from TRE experiments are compatible with the mechanism proposed by Yersin and co-workers in that the delayed fluorescence comes from thermal activated conversion from triplet states lying close in energy to the S_1_ singlet excited state. Maximum EQEs of 16.57 and 15.64% have been achieved for the solution-processed OLEDs with 5 wt% **Cu-2** and 5 wt% **Cu-3** as emitter, respectively. Such high EL efficiency of **Cu-2**- and **Cu-3**-devices is attributed to the emission of these two Cu(i) complexes to be TADF in nature.

In the EL studies of the blue and orange OLED devices, the roll-off of EQE at high luminance is the least pronounced for the devices with the highest dopant concentration, such as 20 wt% for Zn(ii) complexes and 8% for Cu(i) complexes. Since EQE roll-off of OLED devices is mainly caused by exciton–polaron annihilation and field-induced quenching,^24^ the high dopant concentration in the EML, the less exciton formed in the host molecules would be quenched at high luminance. This is because energy transfer from host to dopant is faster and therefore lifetime of host excitons is shorter in the OLED with high dopant concentration. In addition, the relatively lower maximum EQE of the device with the highest dopant concentration caused by singlet–singlet and/or triplet–triplet annihilation also contributes to the least pronounced EQE roll-off.

By combining the blue emission of Zn(ii) complexes and orange emission of Cu(i) complexes, solution-processed white OLEDs have been realized. Since the EL spectrum of the Cu(i) complex-device is affected by host and HBL materials, both CIE coordinates and CRI of the white OLED fabricated with long-wavelength Cu(i) emitter can be tuned by varying both host and HBL materials. With the device structure of ITO/PEDOT:PSS/PVK:OXD-7:**Zn-1**:**Cu-**/3TPYMB/TPBi/LiF/Al, a solution-processed white OLED with CIE coordinates of (0.42, 0.44) and CRI = 81 at 300 cd m^–2^ has been realized. The maximum EQE of 6.88% of this device is the *highest* among those of the reported solution-processed white OLEDs with fluorescent emitters.^4a–c^ It is conceived that both CIE coordinates and CRI could be further improved by using Zn(ii) complexes having higher energy emission.

## Conclusions

A panel of new fluorescent Zn(ii) complexes, **Zn-1–Zn-3**, with high emission quantum yields of up to 0.96 in thin film samples have been developed. Blue solution-processed OLEDs with EQE of up to 5.55% were accomplished with **Zn-1**. EL properties of the yellow to orange emitting Cu(i) complexes, **Cu-2**, **Cu-3** and **Cu-5**, were studied. Both EL spectrum and efficiency of solution-processed OLED fabricated with **Cu-2** or **Cu-3** can be varied by changing the host and BHL materials of the corresponding device. With PYD2/DPEOP as host/BHL, maximum EQEs of 16.57% and 15.64% have been achieved for the devices fabricated with **Cu-2** and **Cu-3**, respectively. By using **Zn-1** as the blue emitter and **Cu-3** as the orange one, a maximum EQE of 6.88% and good colour quality with CIE coordinates of (0.42, 0.44) and CRI = 81 at 300 cd m^–2^ were achieved.
