# Supplementary material for: Luminescent zinc(ii) and copper(i) complexes for high-performance solution-processed monochromic and white organic light-emitting devices
Source: Chem Sci. 2015 Jun 2;6(8):4623–35. doi: 10.1039/c4sc03161j (PMC5667404; doi:10.1039/c4sc03161j)
Supplement: Supplementary file 1 [file SC-006-C4SC03161J-s001.pdf]

Electronic Supplementary Material (ESI) for Chemical Science.
This journal is © The Royal Society of Chemistry 2015

Electronic Supplementary Information

Luminescent zinc(II) and copper(I) complexes for high-performance solution-processed monochromic and white organic light-emitting devices

Gang Cheng,[a,c,f] Gary Kwok-Ming So,[a] Wai-Pong To,[a] Yong Chen,[b] Chi-Chung Kwok,[a,f] Chensheng Ma,[a,e] Xiangguo Guan,[a] Xiaoyong Chang,[a] Wai-Ming Kwok[d] and Chi-Ming Che*[a,f]

[a] State Key Laboratory of Synthetic Chemistry, HKU-CAS Joint Laboratory on New Materials, and Department of Chemistry, The University of Hong Kong, Pokfulam Road, Hong Kong SAR, China. E-mail: cmche@hku.hk
[b] Key Laboratory of Photochemical Conversion and Optoelectronic Materials, Technical Institute of Physics and Chemistry, Chinese Academy of Sciences, Beijing 100190, China.
[c] State Key Laboratory on Integrated Optoelectronics, College of Electronic Science and Engineering, Jilin University, Changchun 130012, China.
[d] Department of Applied Biology and Chemical Technology, The Hong Kong Polytechnic University, Hung Hom, Kowloon, Hong Kong SAR, China.
[e] School of Chemistry and Chemical Engineering, Shenzhen University, Shenzhen 518060, China.
[f] HKU Shenzhen Institute of Research and Innovation, Shenzhen 518053, China.

Table of contents

**1.** Device fabrication and characterization	S3
**2.** Experimental details for time-resolved emission and temperature dependent emission measurements	S3
**3.** Experimental details for emission and lifetime measurements	S4
**4.** Experimental details for cyclic voltammetry and X-ray diffraction	S4
**5.** Thermogravimetric properties of **Zn-1**–**Zn-3**	S4
**Fig. S1** Thermogravimetric curves of **Zn-1**–**Zn-3**	S4
**6.** Photophysical properties of **Zn-1**–**Zn-3**	S5
**Fig. S2** Absorption and emission spectra of **Zn-1** in various solvents	S5
**Figs. S3–S4** Emission spectra of **Zn-1**–**Zn-3** in solid state and in 2-MeTHF glassy solution	S6–S7
**Fig. S5** PL spectra of **Zn-1**–**Zn-3** in PMMA thin film	S8
**7.** Photophysical properties of **Cu-1**–**Cu-5**	S9
**Fig. S6** Absorption spectra of **Cu-1**–**Cu-5** in CH_2Cl_2 solution	S9
**Fig. S7** Solid-state emission spectra of **Cu-1**–**Cu-5**	S9
**Fig. S8** Absorption and emission spectra of **Cu-3** in different solvents	S10
**Fig. S9.** PL emission of **Cu-2** and **Cu-5** in PYD2 thin film	S11
**8.** fs-Time-resolved fluorescence of **Zn-1**–**Zn-3**	S12
**Fig. S10** Time-resolved fluorescence of **Zn-1**–**Zn-3** in CH_2Cl_2	S12
**9.** ns-Time-resolved fluorescence of Cu complexes	S13
**Fig. S11** ns-TRE of **Cu-3** in CH_2Cl_2 after excitation at 350 nm and ns-TRE decay profile of **Cu-3** in CH_2Cl_2 recorded at 350 nm	S13
**Fig. S12** UV-vis absorption spectra of **Cu-1**, **Cu-2**, and **Cu-3** in CH_2Cl_2 recorded before and after the ns-TRE measurement with excitation at 350 nm	S13
**10.** Emission lifetime measurements at various temperatures for **Cu-2** and **Cu-5**	S14
**Figs. S1**3–**S14** Emission decay time of **Cu-2** and **Cu-5** versus temperature	S14–S15
**11.** Electrochemical properties of **Zn-1**–**Zn-3**	S16
**Figs. S15–S17** Cyclic voltammograms of **Zn-1**–**Zn-3** in CH_2Cl_2	S16–S17
**12.** Electrochemical properties of **Cu-1**–**Cu-5**	S18
**Figs. S18–S19** Cyclic voltammograms of **Cu-1**–**Cu-5** in CH_2Cl_2	S18
**13–15.** EL properties of **Zn-1**–**Zn-3**	S19–S21
**Figs. S20–S22** Current density-voltage, luminance-voltage, and current efficiency-luminance characteristics of PLEDs based on **Zn-1**–**Zn-3** at different doping concentrations	S19–S21
**16.** EL properties of Cu(I) complexes	S22
**Figs. S23–S24** Current density-luminance-voltage characteristics of PYD2/DPEOP devices based on **Cu-2–Cu-3** at different doping concentrations	S22
**Table S1** Key performance parameters of PVK/3TPYMB devices with **Cu-3**	S23
**Fig. S25** Normalized EL spectra, current efficiency-luminance, current density-voltage, and luminance-voltage characteristics of PVK/3TPYMB devices based on **Cu-3** at different doping concentrations	S23

**17**. EL properties of Cu(pop)(pz_2Bph_2)	S24
**Fig. S26** EL spectrum and current efficiency-luminance characteristics of PYD2/DPEOP devices based on 10 wt% Cu(pop)(pz_2Bph_2).	S24
**18.** Performances of the white PLEDs with 10 wt% **Zn-1** and 1 wt% **Cu-3**	S25
**Table S2** Key performance parameters of the white PLEDs with 10 wt% **Zn-1** and 1 wt% **Cu-3** at different voltages	S25
**Fig. S27**. Device lifetime of solution-processed OLEDs with **Zn-1** (8 wt%), **Cu-3** (5 wt%), as well as that of the white device with both **Zn-1** (10 wt%) and **Cu-3** (1 wt%)	S25
**19.** Synthesis of Zn(II) complexes	S26
Synthetic procedures and characterization data	S26–29
**Fig. S28** ^{1}H NMR spectrum of 4-bromo-1-(phenylsulfonyl)-1*H*-pyrrolo[2,3-*b*]pyridine in $CDCl_3$	S29
**Fig. S29** ^{13}C NMR spectrum of 4-bromo-1-(phenylsulfonyl)-1*H*-pyrrolo[2,3-*b*]pyridine in $CDCl_3$	S30
**Fig. S30** ^{1}H NMR spectrum of 4-phenyl-1-(phenylsulfonyl)-1*H*-pyrrolo[2,3-*b*]pyridine in $CDCl_3$	S30
**Fig. S31** ^{13}C NMR spectrum of 4-phenyl-1-(phenylsulfonyl)-1*H*-pyrrolo[2,3-*b*]pyridine in $CDCl_3$	S31
**Fig. S32** ^{1}H NMR spectrum of 1-(phenylsulfonyl)-4-(thiophen-2-yl)-1*H*-pyrrolo[2,3-*b*]pyridine in CD_2Cl_2	S31
**Fig. S33** ^{13}C NMR spectrum of 1-(phenylsulfonyl)-4-(thiophen-2-yl)-1*H*-pyrrolo[2,3-*b*]pyridine in CD_2Cl_2	S32
**Fig. S34** ^{1}H NMR spectrum of 4-(naphthalen-2-yl)-1-(phenylsulfonyl)-1*H*-pyrrolo[2,3-*b*]pyridine in CD_2Cl_2	S32
**Fig. S35** ^{13}C NMR spectrum of 4-(naphthalen-2-yl)-1-(phenylsulfonyl)-1*H*-pyrrolo[2,3-*b*]pyridine in CD_2Cl_2	S33
**Fig. S36** ^{1}H NMR spectrum of 4-phenyl-1*H*-pyrrolo[2,3-*b*]pyridine in CD_2Cl_2	S33
**Fig. S37** ^{13}C NMR spectrum of 4-phenyl-1*H*-pyrrolo[2,3-*b*]pyridine in [D_6]DMSO	S34
**Fig. S38** ^{1}H NMR spectrum of 4-(thiophen-2-yl)-1*H*-pyrrolo[2,3-*b*]pyridine in CD_2Cl_2	S34
**Fig. S39** ^{13}C NMR spectrum of 4-(thiophen-2-yl)-1*H*-pyrrolo[2,3-*b*]pyridine in [D_6]DMSO	S35
**Fig. S40** ^{1}H NMR spectrum of 4-(naphthalen-2-yl)-1*H*-pyrrolo[2,3-*b*]pyridine in CD_2Cl_2	S35
**Fig. S41** ^{13}C NMR spectrum of 4-(naphthalen-2-yl)-1*H*-pyrrolo[2,3-*b*]pyridine in [D_6]DMSO	S36
**Fig. S42** ^{1}H NMR spectrum of **Zn-1** in $CDCl_3$	S36
**Fig. S43** ^{13}C NMR spectrum of **Zn-1** in $CDCl_3$	S37
**Fig. S44** ^{1}H-^{13}C HSQC NMR spectrum of **Zn-1** in $CDCl_3$	S37
**Fig. S45** ^{1}H NMR spectrum of **Zn-2** in $CDCl_3$	S38
**Fig. S46** ^{13}C NMR spectrum of **Zn-2** in $CDCl_3$	S38
**Fig. S47** ^{1}H-^{13}C HSQC NMR spectrum of **Zn-2** in $CDCl_3$	S39
**Fig. S48** ^{1}H NMR spectrum of **Zn-3** in $CDCl_3$	S39
**Fig. S49** ^{13}C NMR spectrum of **Zn-3** in $CDCl_3$	S40
**Fig. S50** ^{1}H-^{13}C HSQC NMR spectrum of **Zn-3** in $CDCl_3$	S40
**20.** Synthesis of Cu(I) complexes	S41
Synethic procecdures and characterization data	S41–S42
**Fig. S51** ^{1}H NMR spectrum of **Cu-1** in [D_6]DMSO	S42
**Fig. S52** ^{13}C NMR spectrum of **Cu-1** in [D_6]DMSO	S43
**Fig. S53** ^{1}H NMR spectrum of **Cu-2** in [D_6]DMSO	S43
**Fig. S54** ^{13}C NMR spectrum of **Cu-2** in [D_6]DMSO	S44
**Fig. S55** ^{1}H NMR spectrum of **Cu-3** in $CDCl_3$	S44
**Fig. S56** ^{13}C NMR spectrum of **Cu-3** in $CDCl_3$	S45
**Fig. S57** ^{1}H NMR spectrum of **Cu-4** in [D_6]DMSO	S45
**Fig. S58** ^{13}C NMR spectrum of **Cu-4** in [D_6]DMSO	S46
**Fig. S59** ^{1}H NMR spectrum of **Cu-5** in $CDCl_3$	S46
**21.** X-ray crystal data of $Zn_4O(AID)_6$, **Cu-1** and **Cu-3**	S47
**Table S3** Crystal data of $Zn_4O(AID)_6$, **Cu-1** and **Cu-3**	S47
**Fig. S60** Perspective view of $Zn_4O(AID)_6$	S48
**Fig. S61** Perspective view of **Cu-1**	S48
**22.** Computational details	S49
**Fig. S62** Comparison of FMO diagram of **Cu-1** and **Cu-2** at ground state (S_0)	S49
**Fig. S63** Comparison of MO diagram of **Cu-1** and **Cu-2** at singlet excited state (S_1)	S49
Geometries of the stationary points in mol2 format	S50–S142
**23.** References	S143

1. Device fabrication and characterization

Materials: PEDOT:PSS [poly(3,4-ethylenedioxythiophene):poly(styrene sulfonic acid)] (Clevios P AI 4083) was purchased from Heraeus, PVK (polyvinylcarbazole) from Sigma-Aldrich, FIrpic, OXD-7, PYD2, 3TPYMB, DPEPO, and TPBi from Luminescence Technology Corp. All of these materials were used as received. The synthetic procedures of **Zn-1−Zn-3** and **Cu-1–Cu-5** are described below (see pages S24–S27 and S39–S40). All Zn(II) complexes were purified by gradient sublimation before use.

Substrate cleaning: Glass slides with pre-patterned ITO electrodes used as substrates of OLEDs were cleaned in an ultrasonic bath of Decon 90 detergent and deionized water, rinsed with deionized water, and then cleaned in sequential ultrasonic baths of deionized water, acetone, and isopropanol, and subsequently dried in an oven for 1 h.

Fabrication and characterization of devices: PEDOT:PSS were spin-coated onto the cleaned ITO-coated glass substrate and baked at 120 ˚C for 20 min to remove the residual water solvent in a clean room. Blends of PVK: OXD-7:emitter(s) or PYD2:emitter(s) were spin-coated from chlorobenzene atop the PEDOT:PSS layer inside a N_2-filled glove box. For the **Cu-5** device, the mixture of Cu-5 and PYD2 was spin-coated from dimethylformamide as EML. The thickness for all EMLs was about 60 nm. Afterwards, all devices except for those with PYD2 as the host were annealed at 110 ˚C for 10 min inside the glove box and subsequently transferred into a Kurt J. Lesker SPECTROS vacuum deposition system without exposing to air. Finally, TmPyPb or 3TPYMB or DPEPO (5 nm), TPBi (40 nm), LiF (1.2 nm), and Al (150 nm) were deposited in sequence by thermal evaporation at a pressure of 10^{-8} mbar. EL spectra were recorded by an Ocean Optics Maya 2000 pro spectrometer or a Photo Research Inc PR-655. Luminance, CIE coordination, and CRI were measured by a Photo Research Inc PR-655. Voltage-current characteristics were measured by a Keithley 2400 source-meter measurement unit. All devices were characterized at room temperature without encapsulation. EQE and power efficiency were calculated by assuming a Lambertian distribution.

2. Experimental details for time-resolved emission and temperature dependent emission measurements

The instrumental set-up for the femtosecond time-resolved emission (fs-TRE) and nanosecond time-resolved emission (ns-TRE) and the related spectral calibrations have been described previously.[1,2] Briefly, all the measurements were performed based on a commercial Ti:Sapphire regenerative amplifier laser system (800 nm, 40 fs, 1 kHz, and 3.5 mJ/pulse). A 350 nm excitation wavelength was used for all the measurements. The 350 nm pump pulse was produced from an optical parametric amplifier (TOPAS) pumped by the 800 nm fundamental laser pulse. The fs-TRE was measured using a Kerr-gate technique.[3] A Kerr device composed of a 1 mm thick Kerr medium (benzene contained in a quartz cell) equipped within a crossed polarizer pair was driven by the 800 nm laser to function as an ultrafast optical shutter to sample transient emission spectra at various selected pump/probe delays. The delay between the probe and pump pulse was controlled by an optical delay line. The fs-TRE signals were collected by a monochromator and detected with a liquid nitrogen cooled CCD detector. The instrument response function (IRF) of the fs-TRE is wavelength-dependent, varying from ~0.5 ps to ~2 ps. For the ns-TRE measurement, an intensified CCD (ICCD) detector, which was synchronized to the fs laser system, was used to detect transient emission spectra with the controlled pump/probe time delay covering from ~2 ns and afterwards. To eliminate the effect of rotational diffusion, the polarization direction of the pump laser was set at the magic angle in relative to that of probe for all the measurements. The measurements were done at room temperature and atmospheric pressure with the samples (concentration of ~1 mM) of ~15 mL flowed in a cell with 0.5 mm path length. The samples were monitored by UV-vis absorption and revealed no degradation after the time-resolved measurement.

3. Experimental details for emission and lifetime measurements

Steady-state excitation and emission spectra were obtained on a SPEX Fluorolog-3 spectrophotometer. All solutions for photophysical measurements were degassed with no less than three freeze-pump-thaw cycles prior to the measurements. For measurement of emission spectrum at low-temperature (77 K), the solution or solid sample was loaded in a 5-mm-diameter quartz tube that was immersed in a liquid nitrogen Dewar flask equipped with quartz windows. The emission spectra were corrected for monochromator and photomultiplier efficiency and for Xenon-lamp stability. Emission lifetime measurements were performed with a Quanta Ray GCR 150-10 pulsed Nd:YAG laser system (pulse output 355 nm, 8 ns). Luminescence quantum yields were measured relative to that of a degassed benzene solution of 9,10-bis(phenylethynyl)anthracene (BPEA, $\Phi_r = 0.85$) or acetonitrile solution of $Ru(bpy)_3(PF_6)_2$ ($\Phi_r = 0.062$) as a standard reference. Errors for λ values (±1 nm), τ (±10%), and Φ (±10%) were estimated.

4. Experimental details for cyclic voltammetry and X-ray diffraction

Cyclic voltammetry was performed using PAR potentiostat 273A equipped with PowerSuite program at a scan rate of 100 mV s^{-1}. The electrolytic cell used was a conventional two compartment cell. Electrochemical measurements were performed at room temperature after purging with nitrogen using 0.1 M tetrabutylammonium hexafluorophosphate (TBAP)/dichloromethane as the supporting electrolyte. The working electrode was a glassy carbon electrode (geometric area 0.35 cm^2) and the counter electrode was platinum gauze. A non-aqueous $Ag/AgNO_3$ (0.1 M in acetonitrile) reference electrode was contained in a separate compartment connected to the test solution via fine sintered glass disks. The ferrocenium/ferrocene couple was used as the internal standard.

X-ray diffraction data were collected by a Bruker X8 diffractometer with MICROSTAR copper rotating anode (Cu K-alpha, 1.54178 angstrom) and Platinum 135 CCD detector. Proteum was used for data collection strategy, integration and scaling. Shelx-97 program suite was used for structure solution (XS) and refinement (XL).

5. Thermogravimetric properties of Zn-1–Zn-3

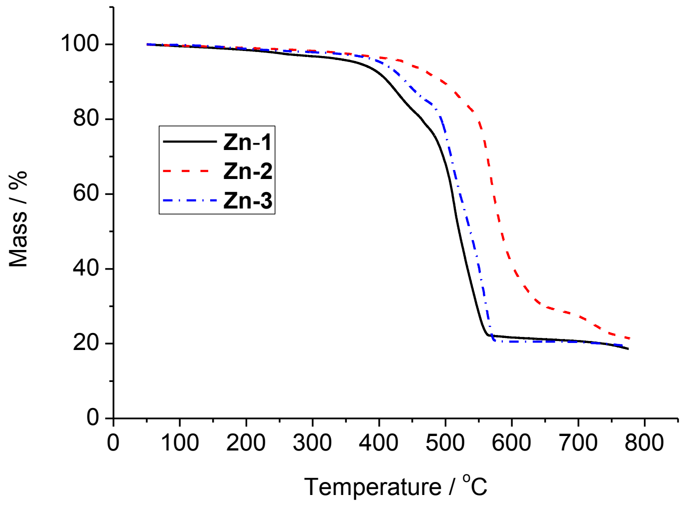

Fig. S1 Thermogravimetric curves of complexes **Zn-1**–**Zn-3** under a constant heating rate of 10 °C min^{-1}.

6. Photophysical properties of Zn-1–Zn-3

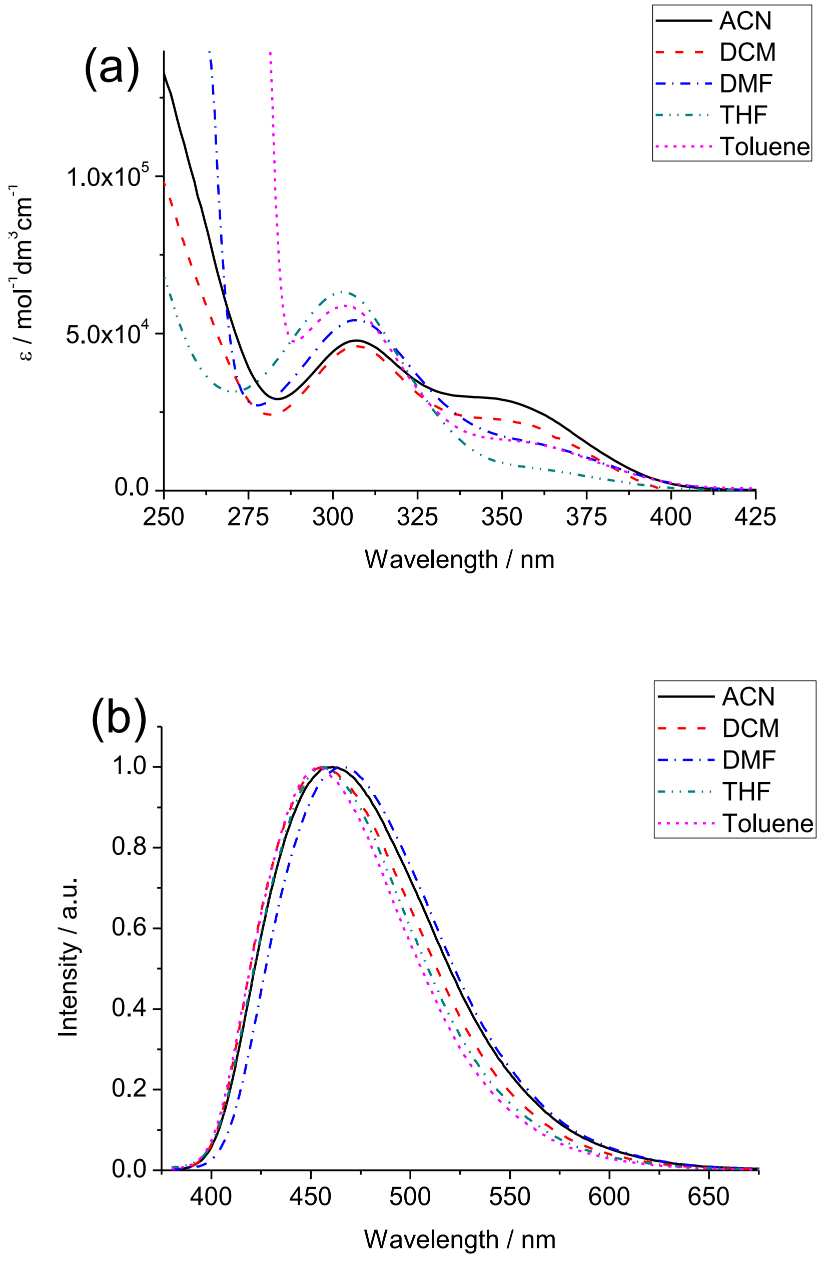

Fig. S2 (a) Absorption and (b) emission spectra of **Zn-1** in various solvents (ACN = MeCN; DCM = CH_2Cl_2).

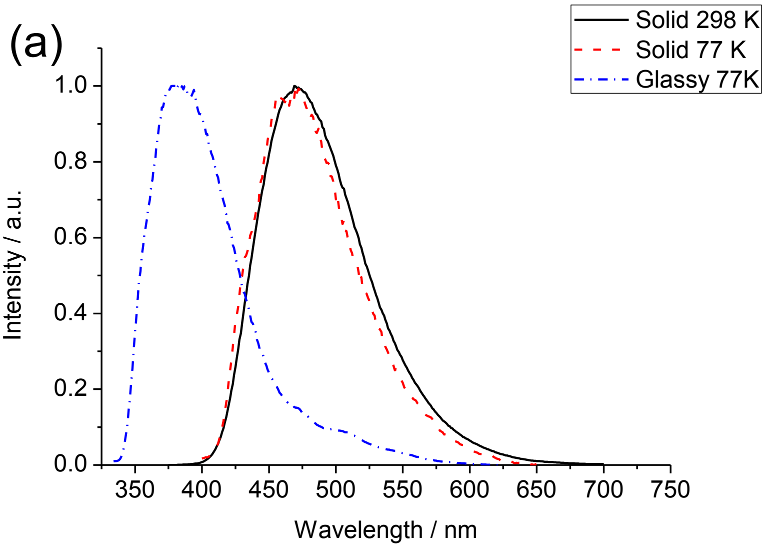

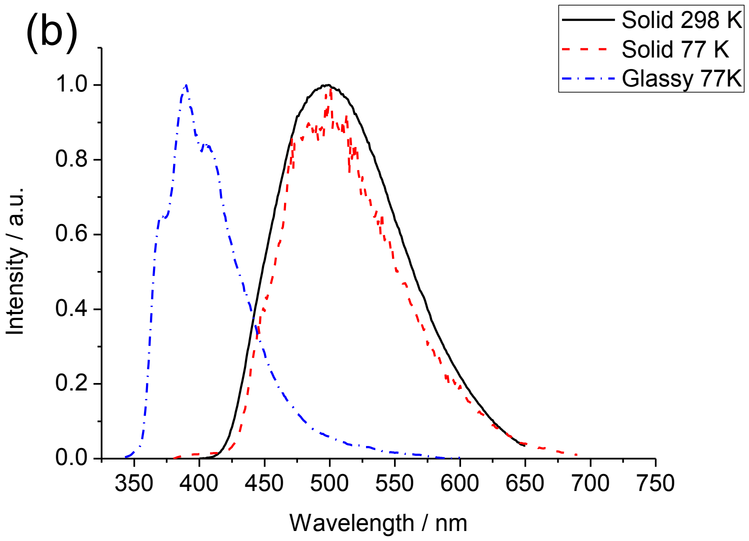

Fig. S3 Emission spectra of (a) **Zn-1** and (b) **Zn-2** in solid state at 298 and 77 K, and in 2-MeTHF glassy solution at 77 K.

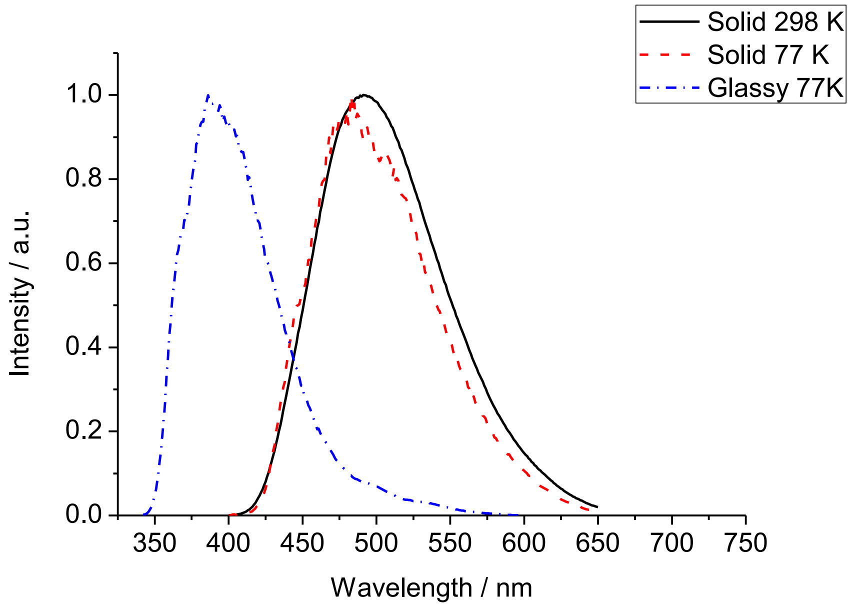

Fig. S4 Emission spectra of **Zn-3** in solid state at 298 and 77 K, and in 2-MeTHF glassy solution at 77 K.

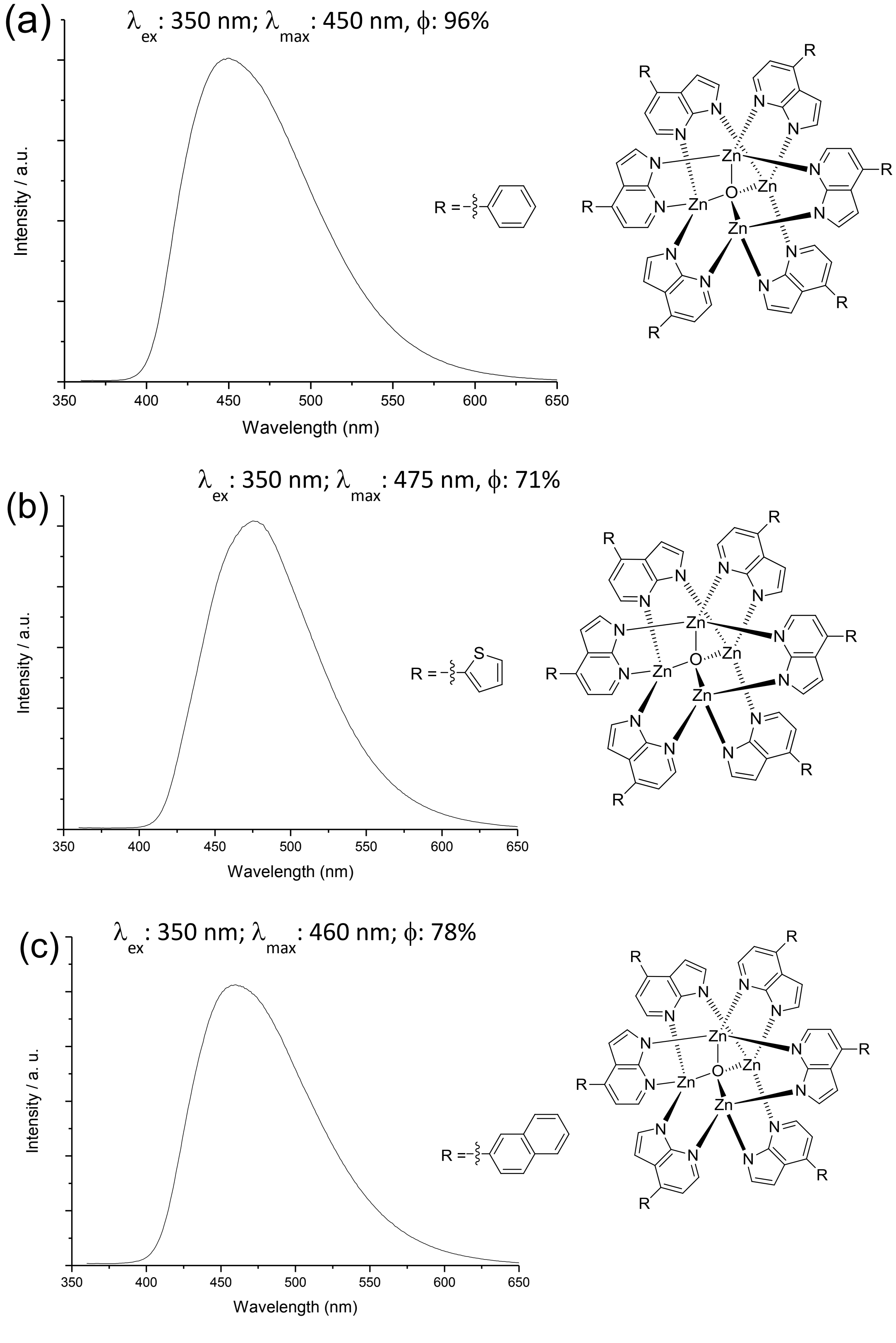

Fig. S5 PL spectra of (a) **Zn-1**, (b) **Zn-2**, and (c) **Zn-3** in PMMA thin film with concentration of 5 wt%.

7. Photophysical properties of Cu-1–Cu-5

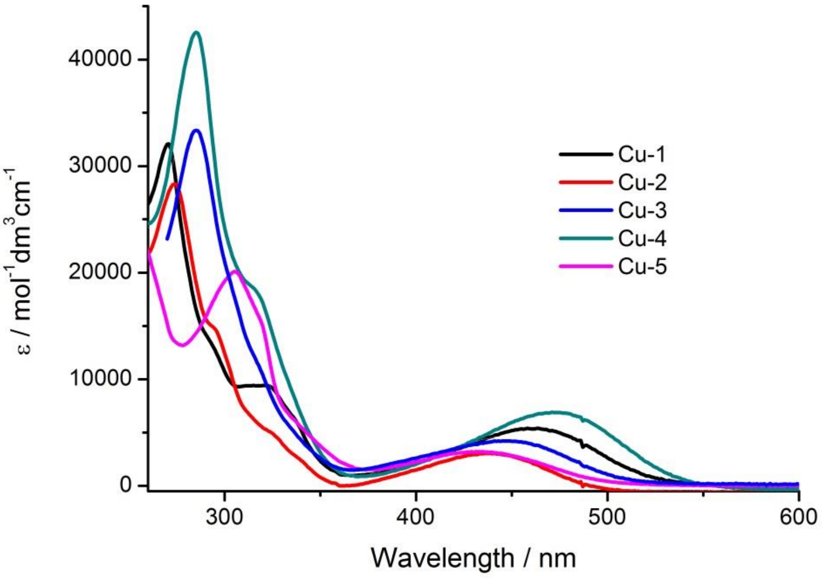

Fig. S6 Absorption spectra of complexes **Cu-1–Cu-5** in CH_2Cl_2 solution.

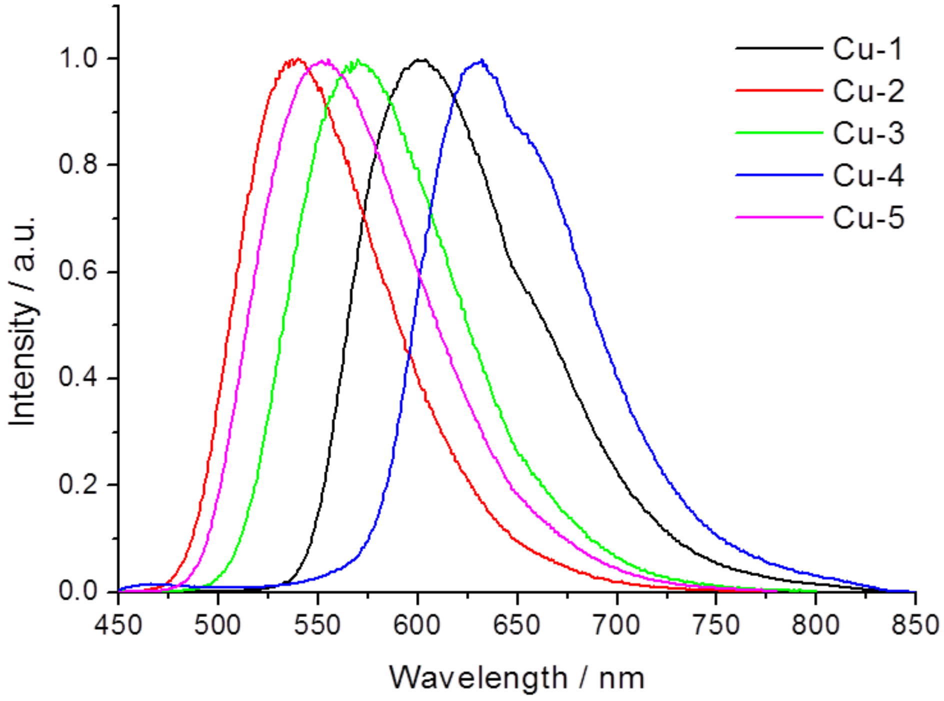

Fig. S7 Solid-state emission spectra of **Cu-1–Cu-5** at room temperature (λ_{ex} = 365 nm).

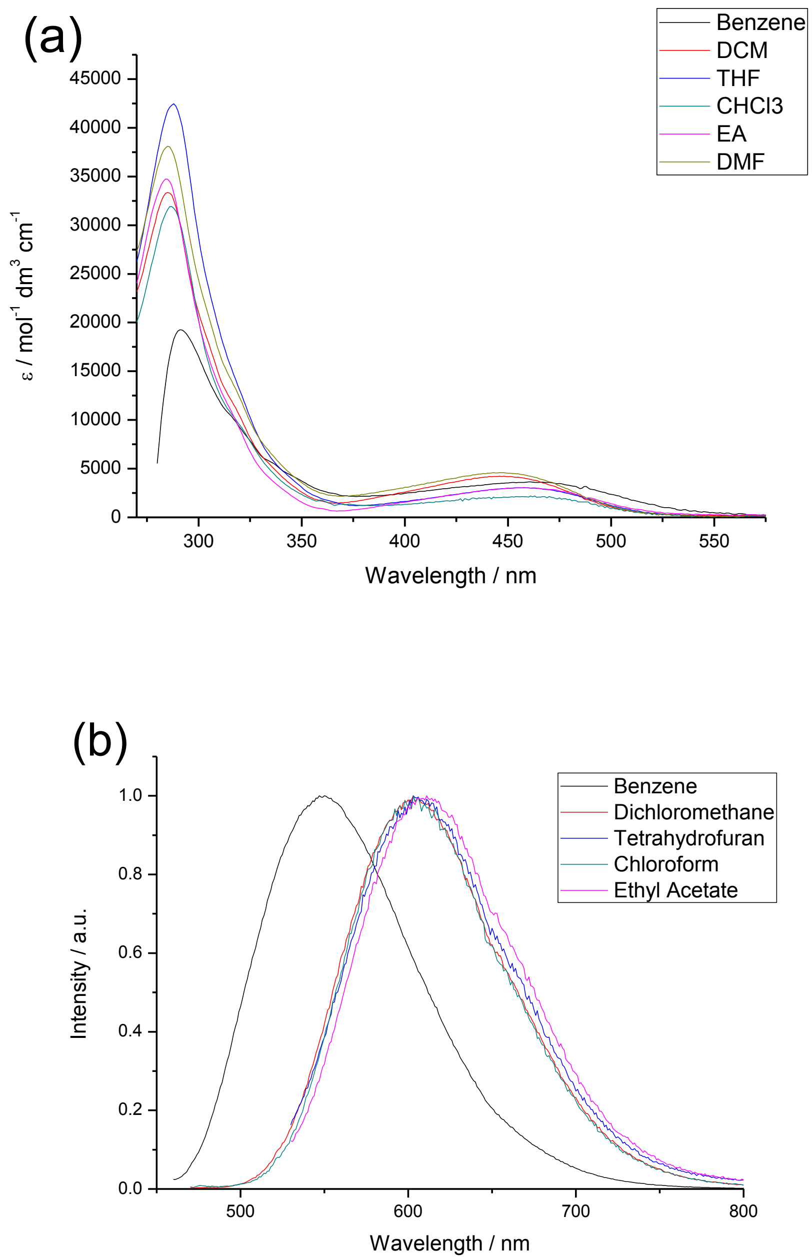

Fig. S8 (a) Absorption and (b) emission spectra of **Cu-3** in different solvents.

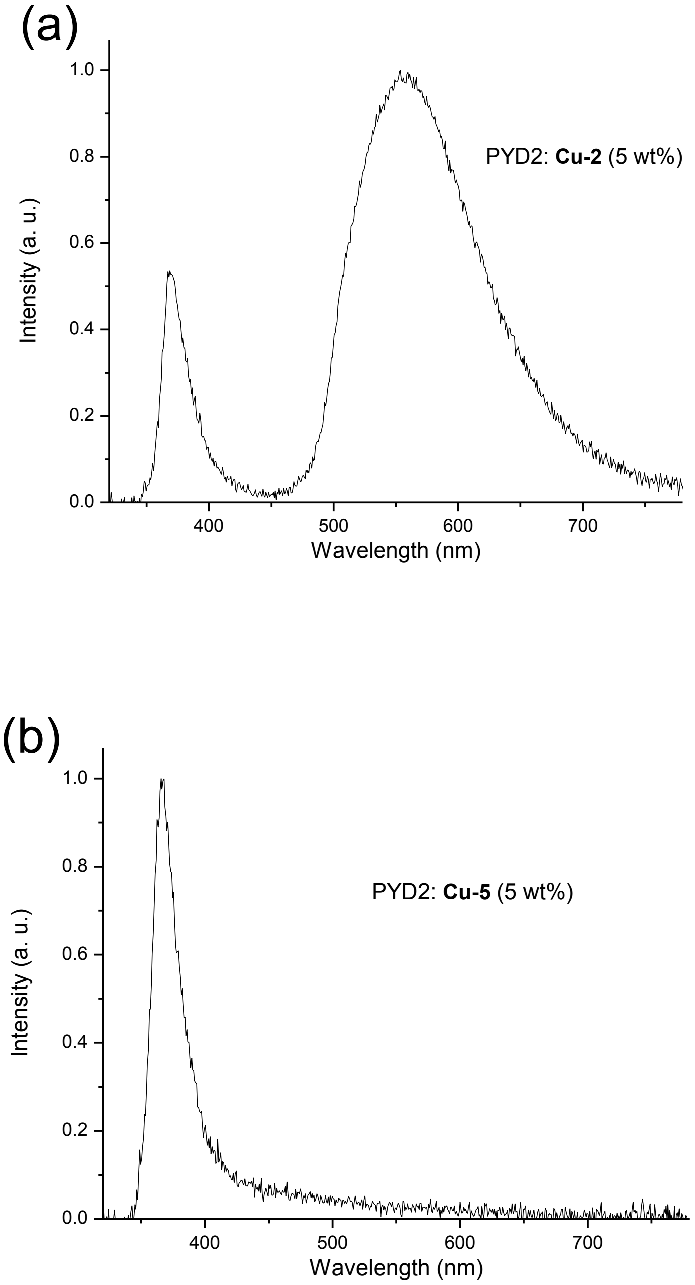

Fig. S9. PL emission of (a) **Cu-2** and (b) **Cu-5** in PYD2 thin film. λ_{ext} = 310 nm.

8. fs-Time-resolved fluorescence of Zn-1–Zn-3

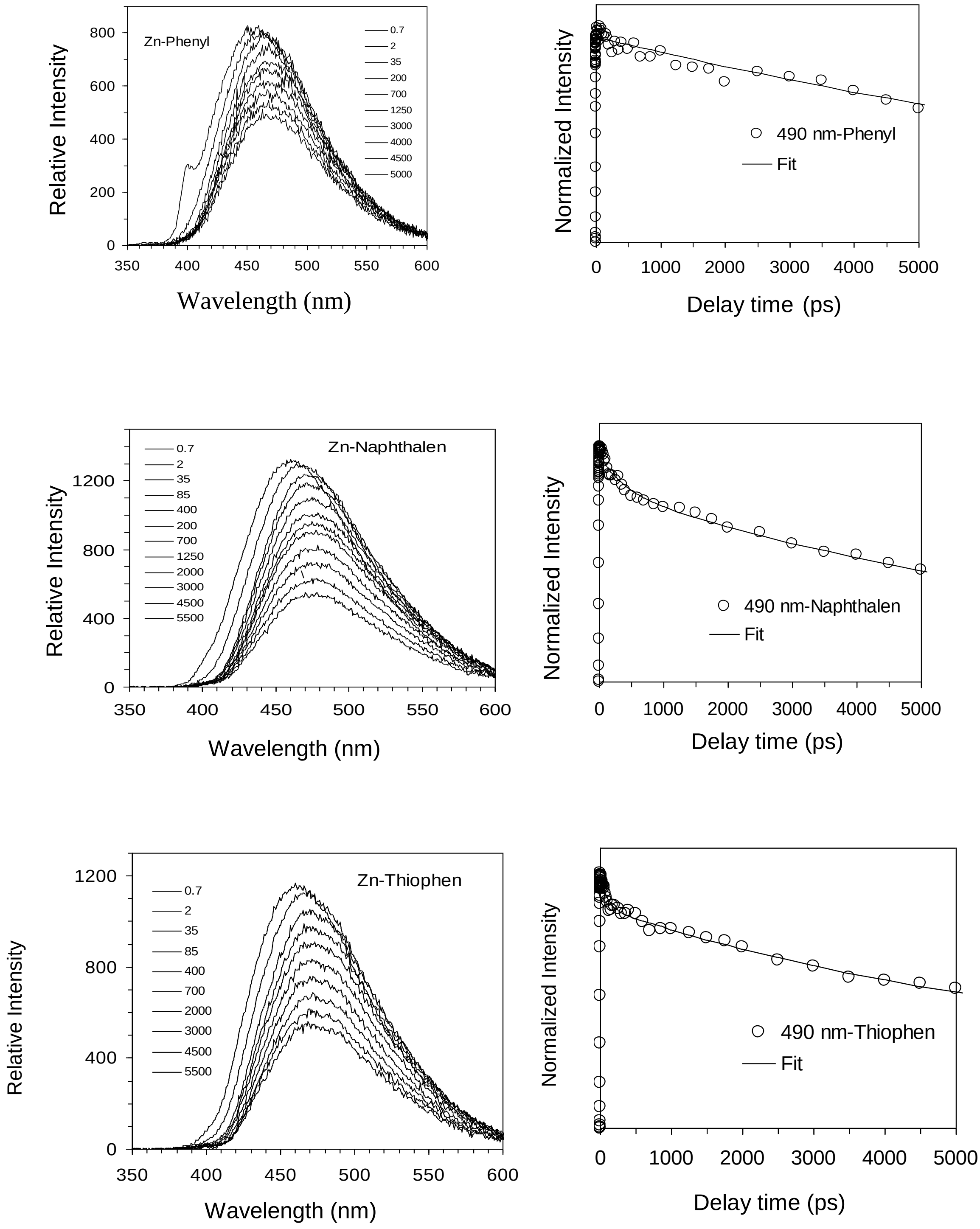

Fig. S10 Time-resolved fluorescence of **Zn-1** (Zn-Phenyl), **Zn-2** (Zn-Naphthalen), and **Zn-3** (Zn-Thiophen) in CH_2Cl_2 with excitation at 350 nm.

9. ns-Time-resolved fluorescence of Cu complexes

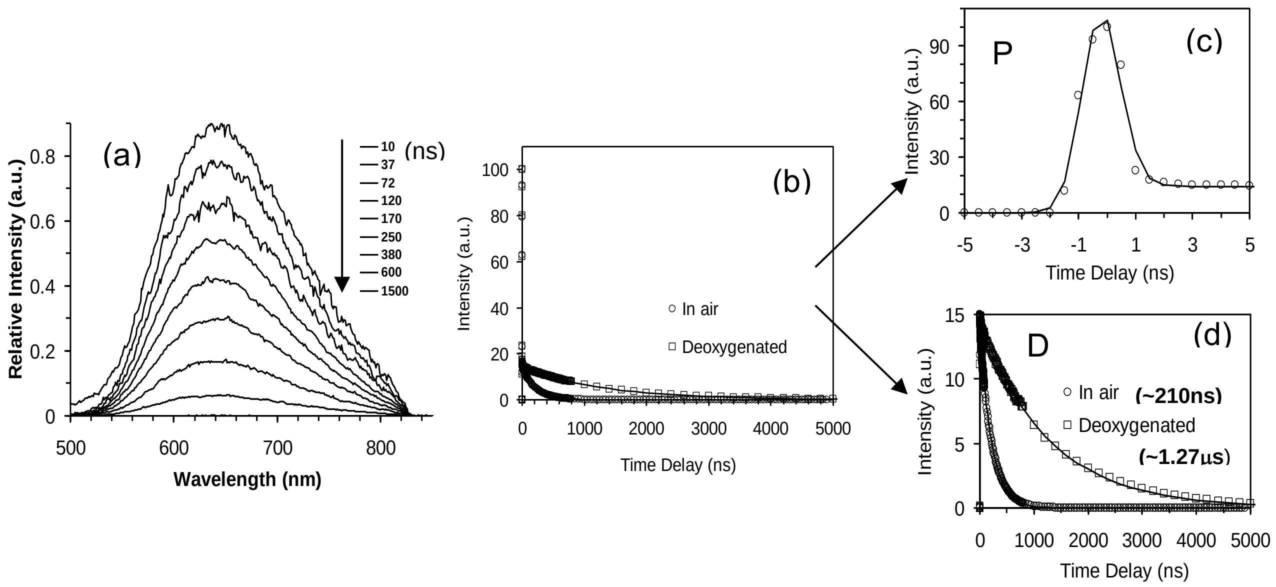

Fig. S11 (a) ns-TRE of **Cu-3** in CH_2Cl_2 (deoxygenated by purging the sample solution with nitrogen) recorded at indicated time intervals after excitation at 350 nm. (b)–(d) ns-TRE decay profile of **Cu-3** in CH_2Cl_2 recorded at 350 nm

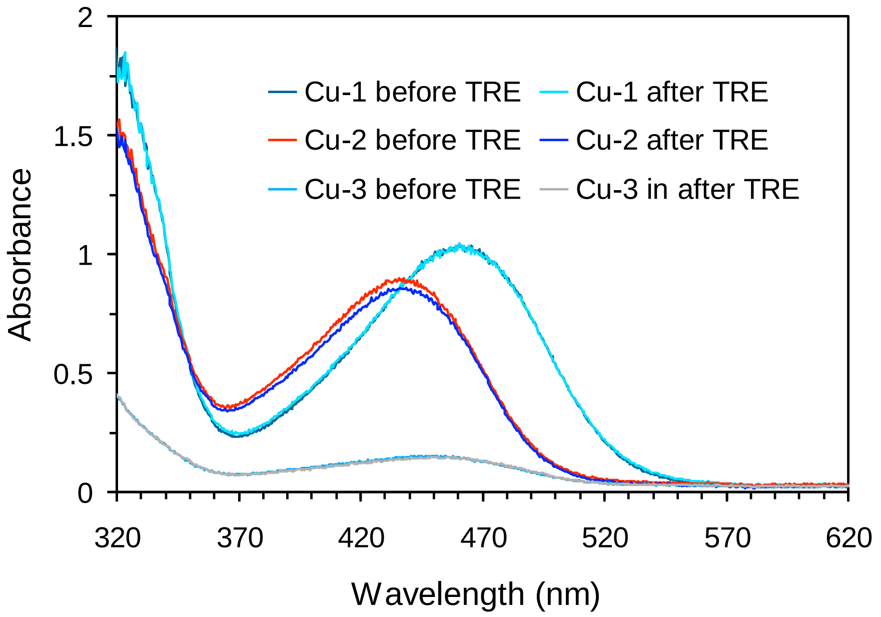

Fig. S12 UV-vis absorption spectra of **Cu-1**, **Cu-2**, and **Cu-3** in CH_2Cl_2 recorded before and after the nanosecond time-resolved emission measurement with excitation at 350 nm.

10. Emission lifetime measurements at various temperatures for Cu-2 and Cu-5

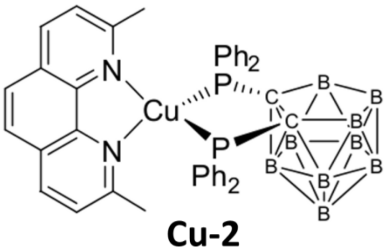

Cu-2

$$\tau(T) = \frac{3 + \exp\left[-\frac{\Delta E(S_1 - T_1)}{k_B T}\right]}{\frac{3}{\tau(T_1)} + \frac{1}{\tau(S_1)}\exp\left[-\frac{\Delta E(S_1 - T_1)}{k_B T}\right]} \quad (1)$$

$\Delta E(S_1 - T_1)$: 1195 cm^{-1}
$\tau(T_1)$: 564 μs
$\tau(S_1)$: 0.01 μs

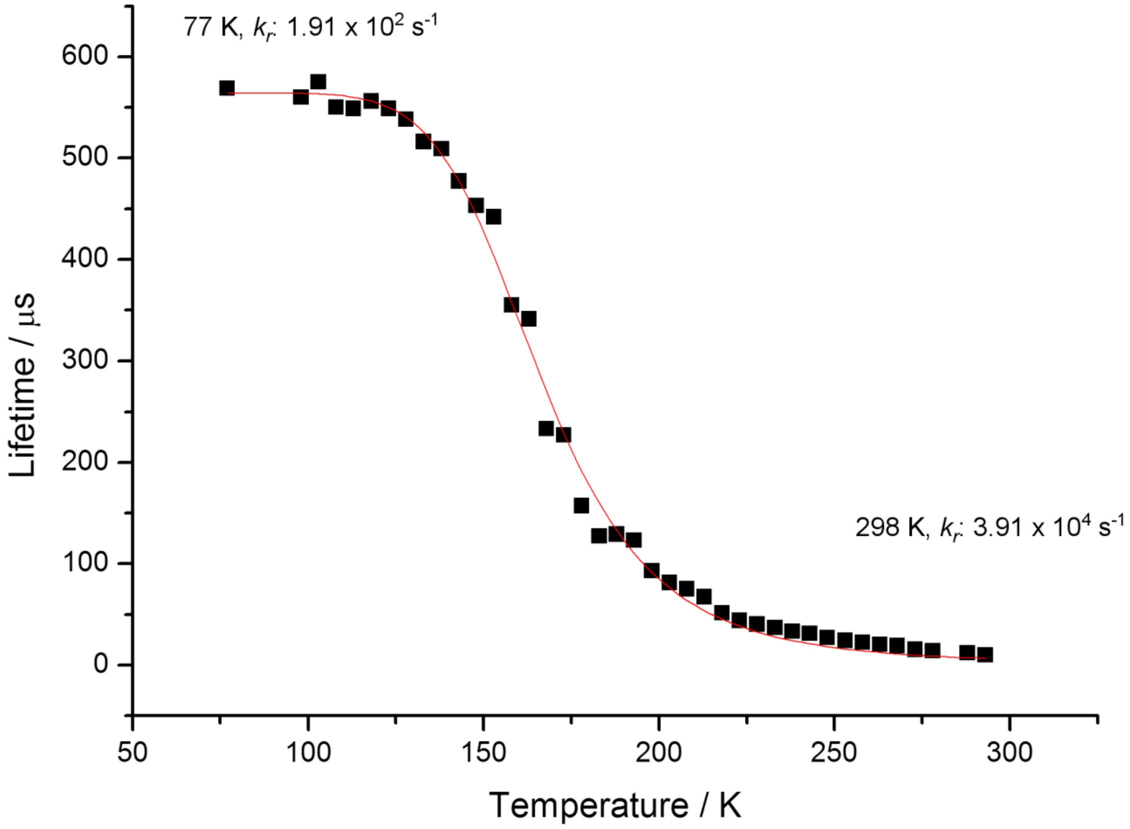

Fig. S13 Emission decay time of **Cu-2** powder versus temperature.

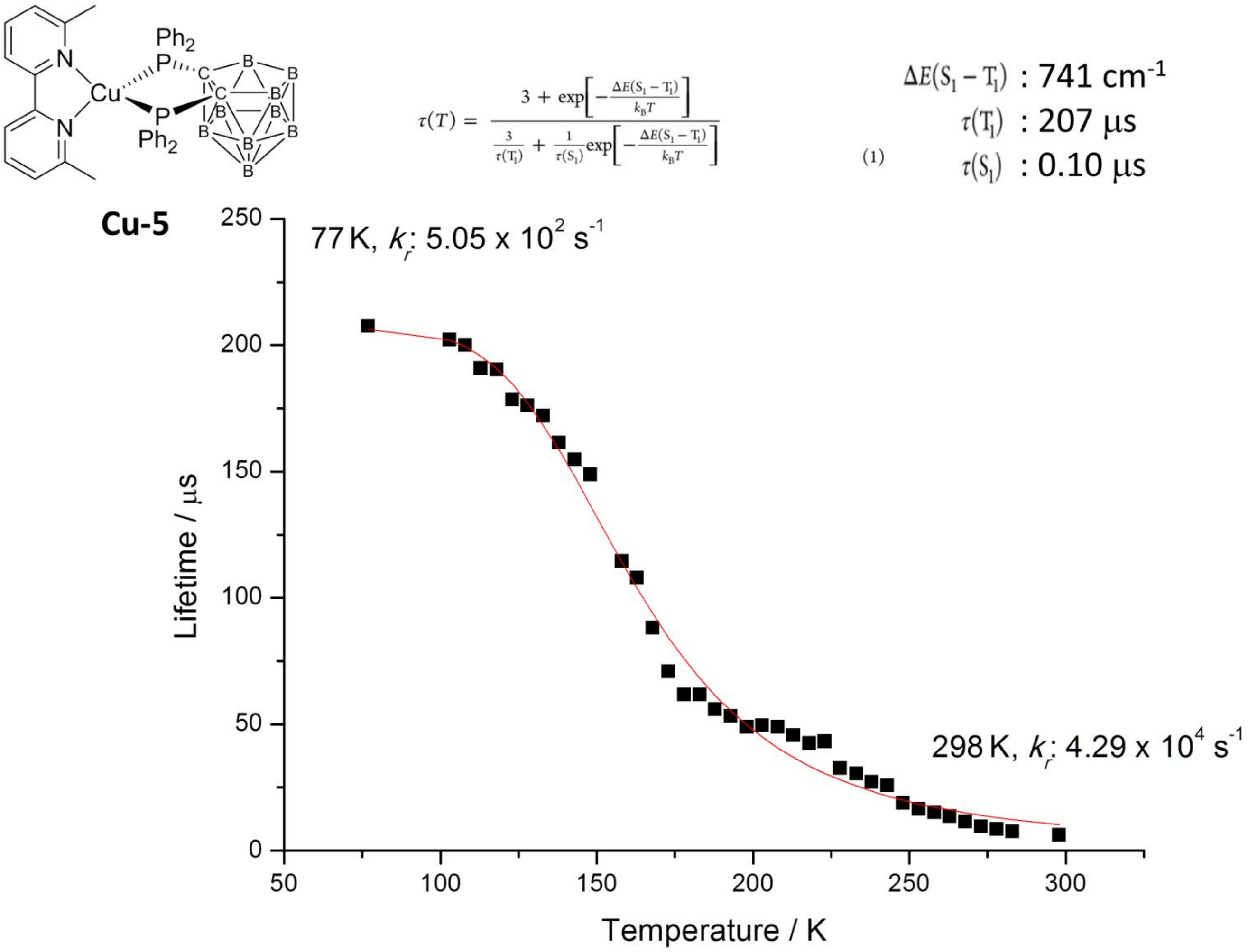

Fig. S14 Emission decay time of **Cu-5** powder versus temperature.

11. Electrochemical properties of Zn-1–Zn-3

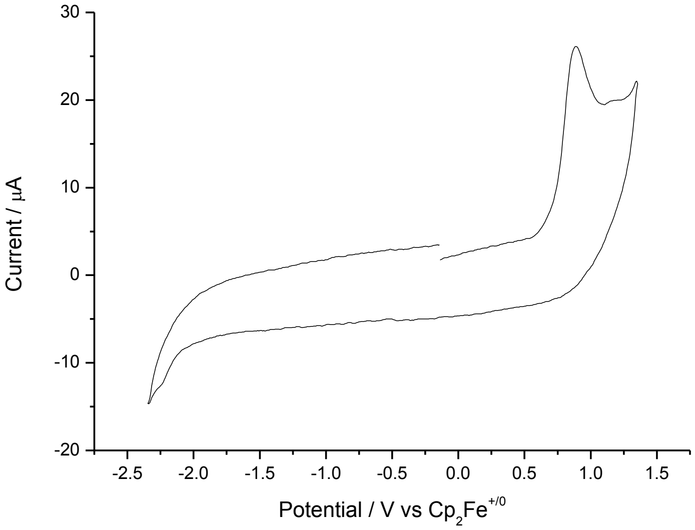

Fig. S15 Cyclic voltammogram of **Zn-1** in CH_2Cl_2 containing [^{n}Bu$_4$N]PF$_6$ (0.1 mol dm^{-3}) as supporting electrolyte. Scan rate: 100 mV s^{-1}.

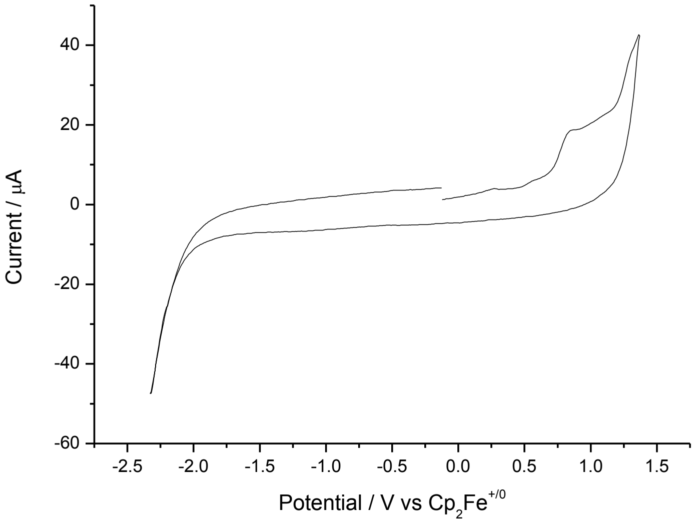

Fig. S16 Cyclic voltammogram of **Zn-2** in CH_2Cl_2 containing [^{n}Bu$_4$N]PF$_6$ (0.1 mol dm^{-3}) as supporting electrolyte. Scan rate: 100 mV s^{-1}.

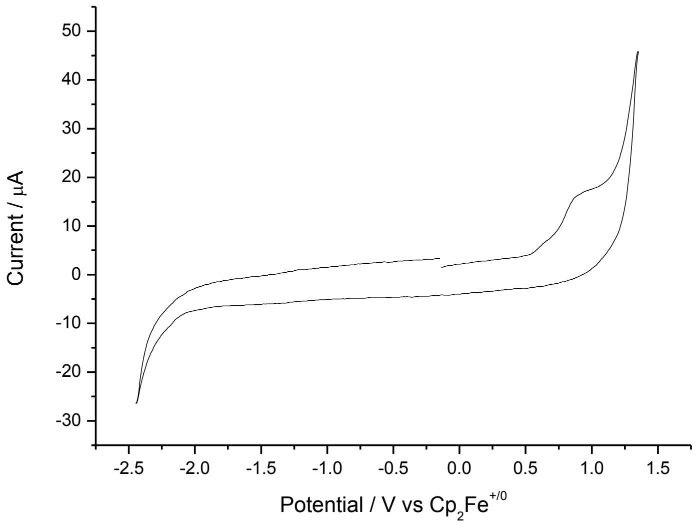

Fig. S17 Cyclic voltammogram of **Zn-3** in CH_2Cl_2 containing [$^{n}Bu_4N$]PF_6 (0.1 mol dm^{-3}) as supporting electrolyte. Scan rate: 100 mV s^{-1}.

12. Electrochemical properties of Cu-1–Cu-5

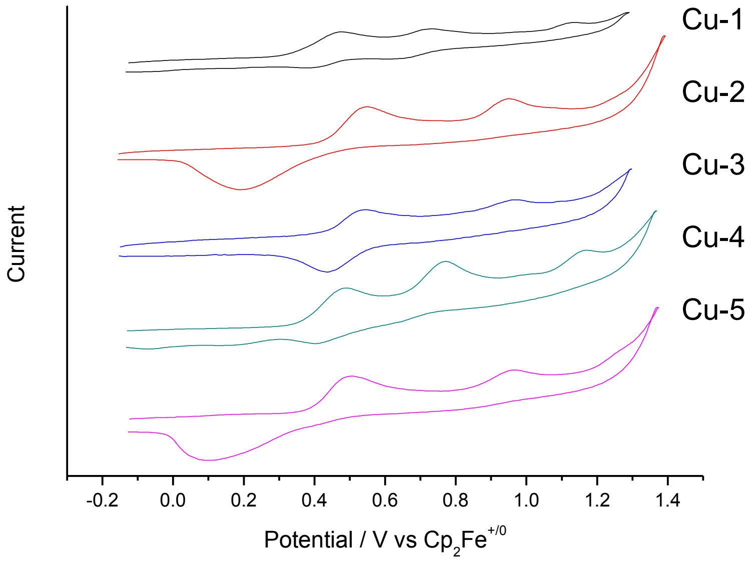

Fig. S18 Cyclic voltammogram of **Cu-1–Cu-5** in CH_2Cl_2 containing [^{n}Bu_4N]PF_6 (0.1 mol dm^{-3}) as supporting electrolyte (oxidation). Scan rate: 100 mV s^{-1}.

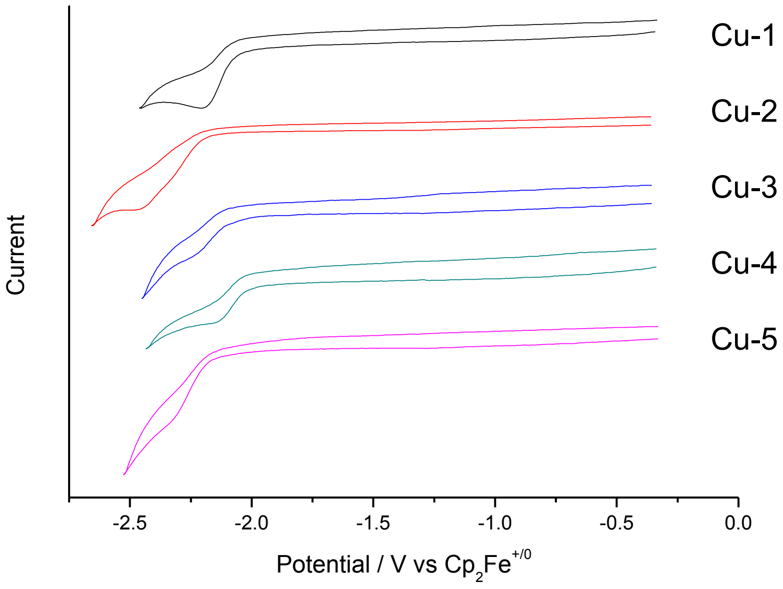

Fig. S19 Cyclic voltammogram of **Cu-1–Cu-5** in CH_2Cl_2 containing [^{n}Bu_4N]PF_6 (0.1 mol dm^{-3}) as supporting electrolyte (reduction). Scan rate: 100 mV s^{-1}.

13. EL properties of Zn-1

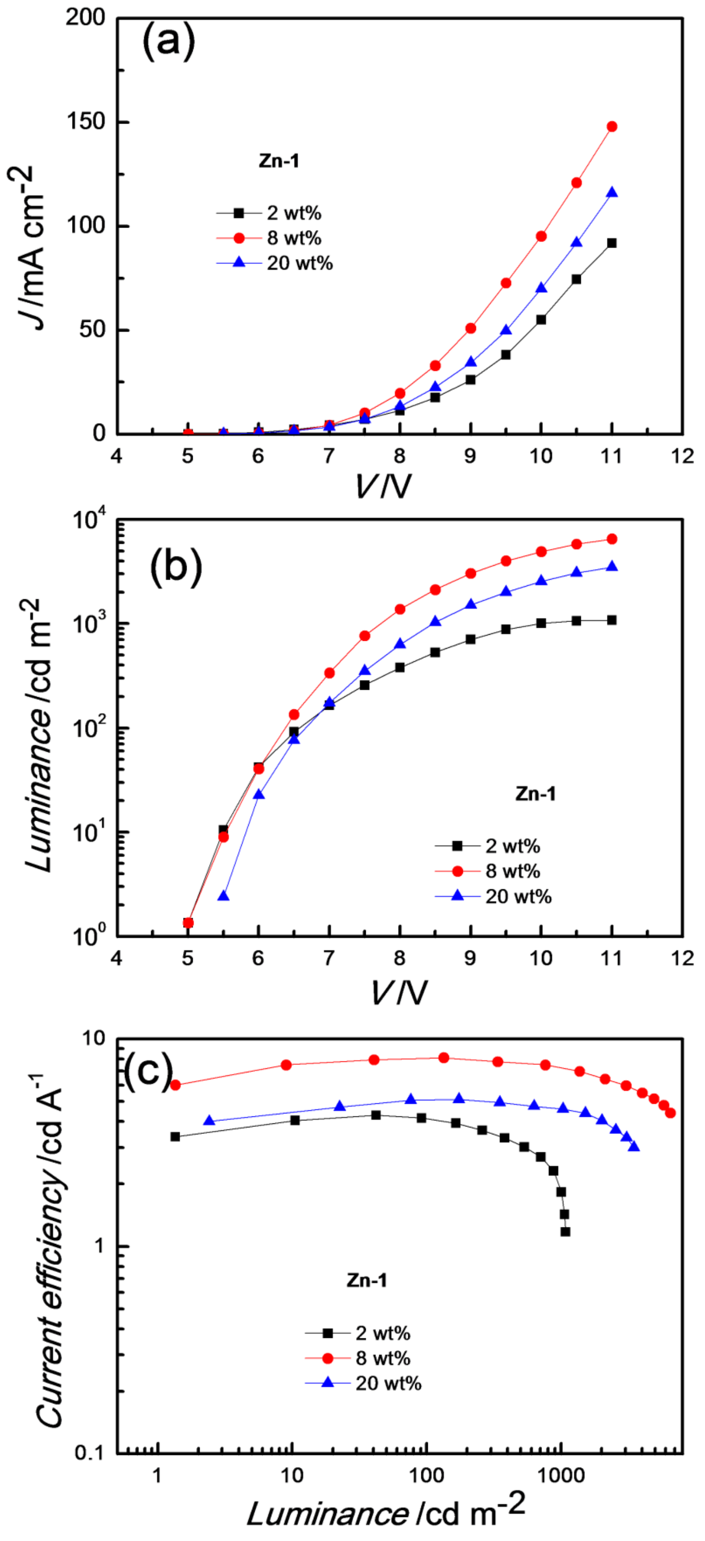

Fig. S20 (a) Current density-voltage, (b) luminance-voltage, and (c) current efficiency-luminance characteristics of PLEDs based on **Zn-1** at different doping concentrations.

14. EL properties of Zn-2

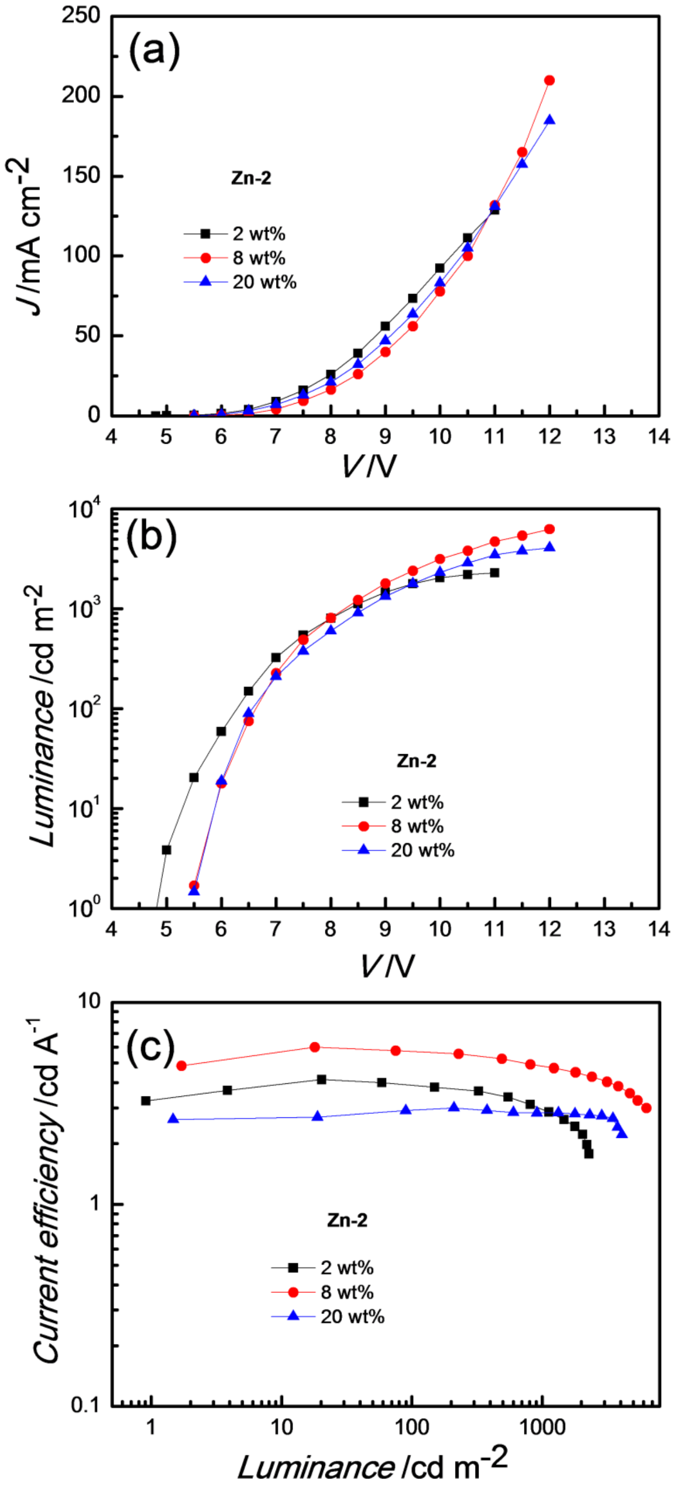

Fig. S21 (a) Current density-voltage, (b) luminance-voltage, and (c) current efficiency-luminance characteristics of PLEDs based on **Zn-2** at different doping concentrations.

15. EL properties of Zn-3

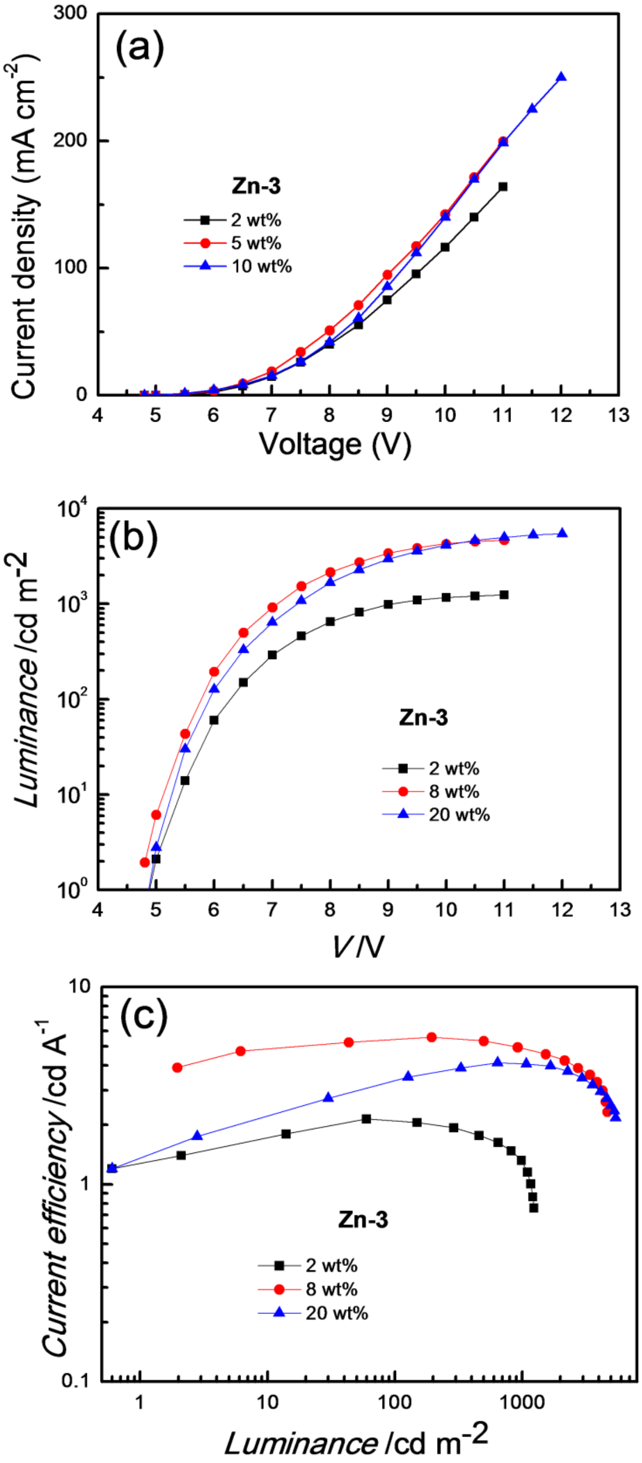

Fig. S22 (a) Current density-voltage, (b) luminance-voltage, and (c) current efficiency-luminance characteristics of PLEDs based on **Zn-3** at different doping concentrations.

16. EL properties of Cu(I) complexes

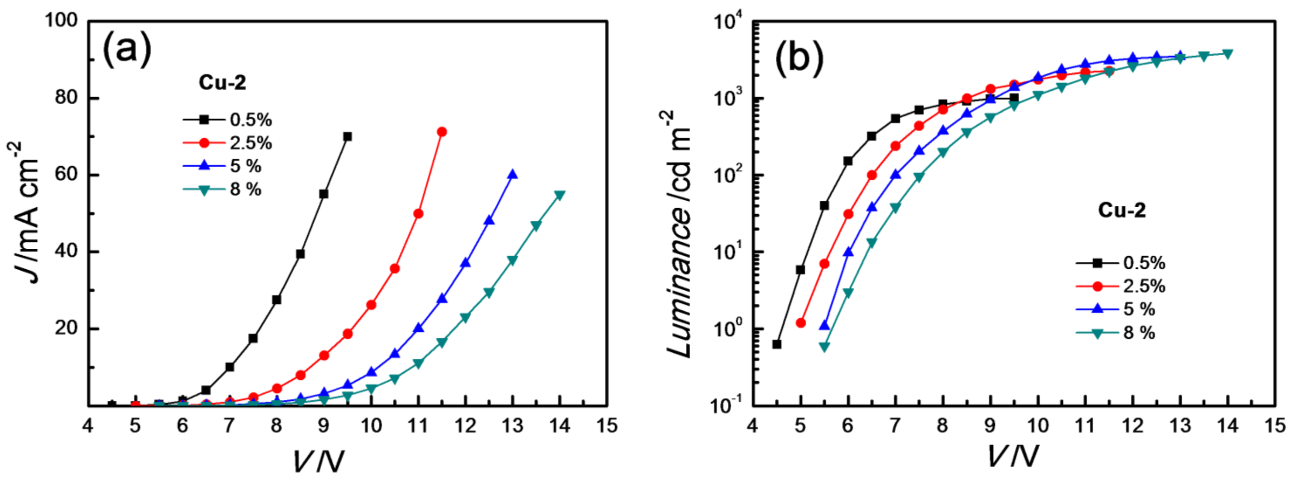

Fig. S23 Current density-luminance-voltage characteristics of PYD2/DPEOP devices based on **Cu-2** at different doping concentrations.

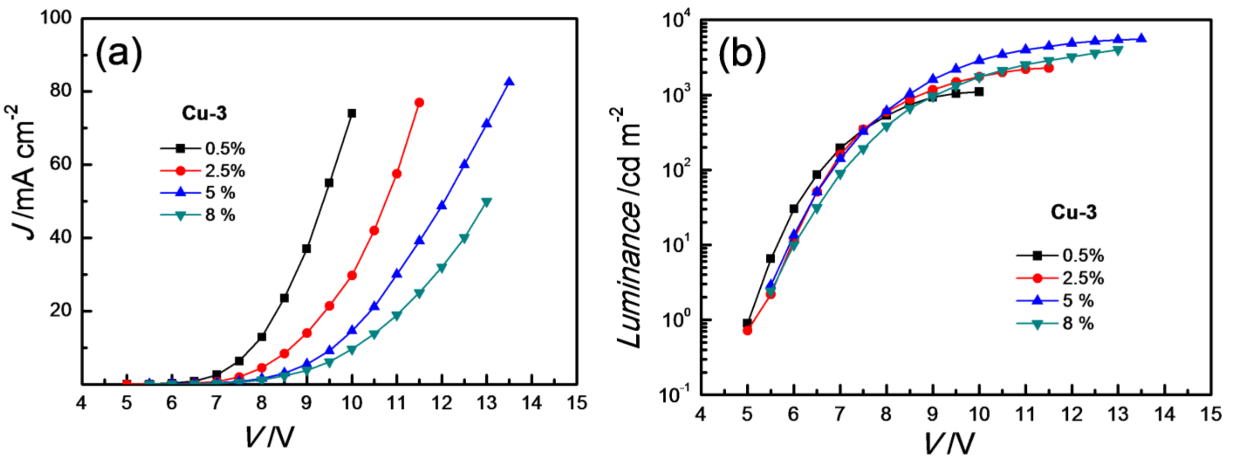

Fig. S24 Current density-luminance-voltage characteristics of PYD2/DPEOP devices based on **Cu-3** at different doping concentrations.

Table S1 Key performance parameters of PVK/3TPYMB devices with **Cu-3**

Doping concentration of **Cu-3**	L[a] (cd m^{-2})	Max. CE[b] (cd A^{-1})	Max. PE[c] (lm W^{-1})	Max. EQE[d] (%)	CIE coordinates (x, y) at 1000 cd m^{-2}
2 wt%	1200	23.33	12.22	10.77	(0.54, 0.45)
5 wt%	1350	22.58	11.88	10.59	(0.54, 0.45)
10 wt%	2520	22.88	13.20	11.43	(0.55, 0.44)

[a] Luminance at 17 V. [b] Current efficiency. [c] Power efficiency. [d] External quantum efficiency.

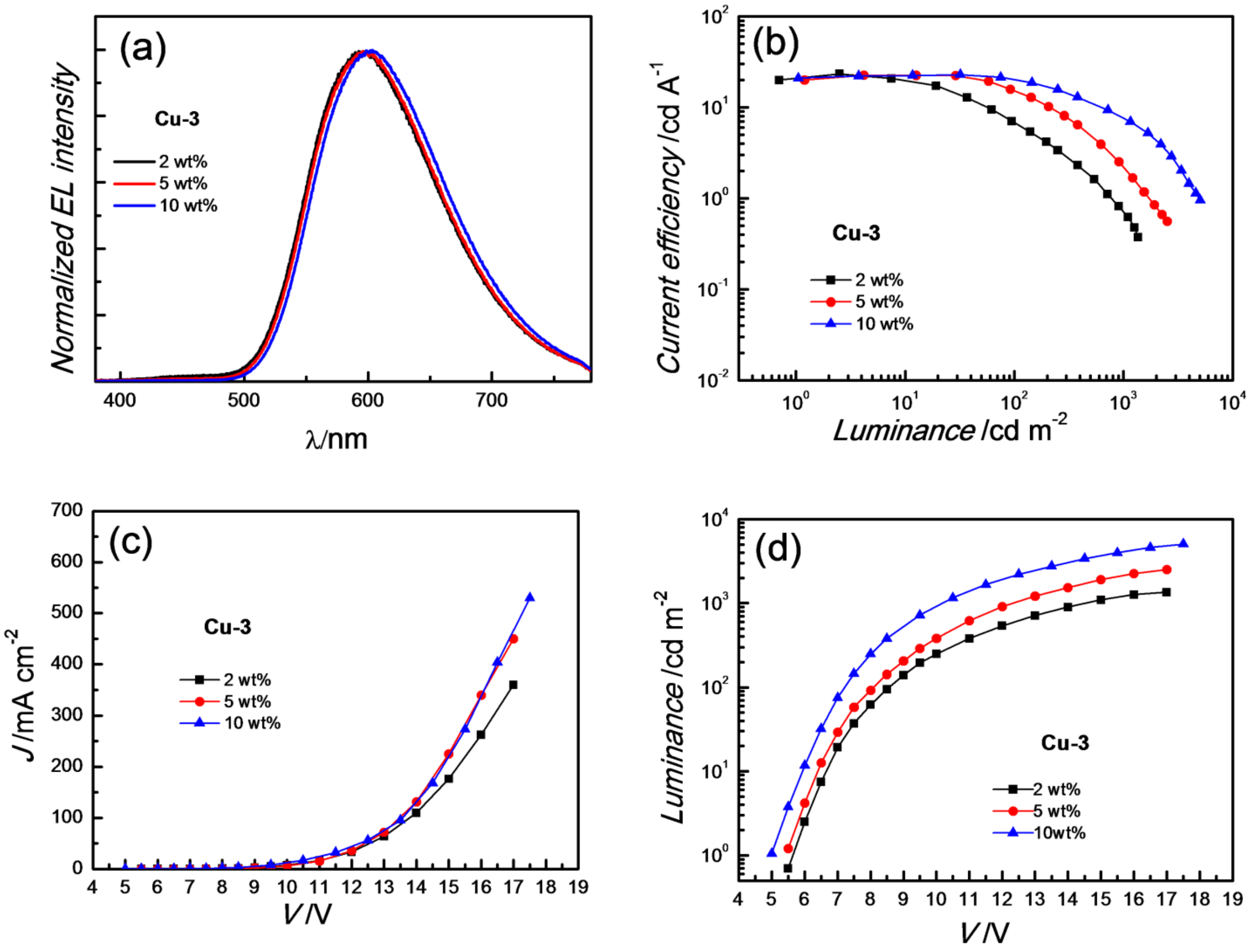

Fig. S25 (a) Normalized EL spectra, (b) current efficiency-luminance, (c) current density-voltage, and (d) luminance-voltage characteristics of PVK/3TPYMB devices based on **Cu-3** at different doping concentrations.

17. EL properties of Cu(pop)(pz_2Bph_2)

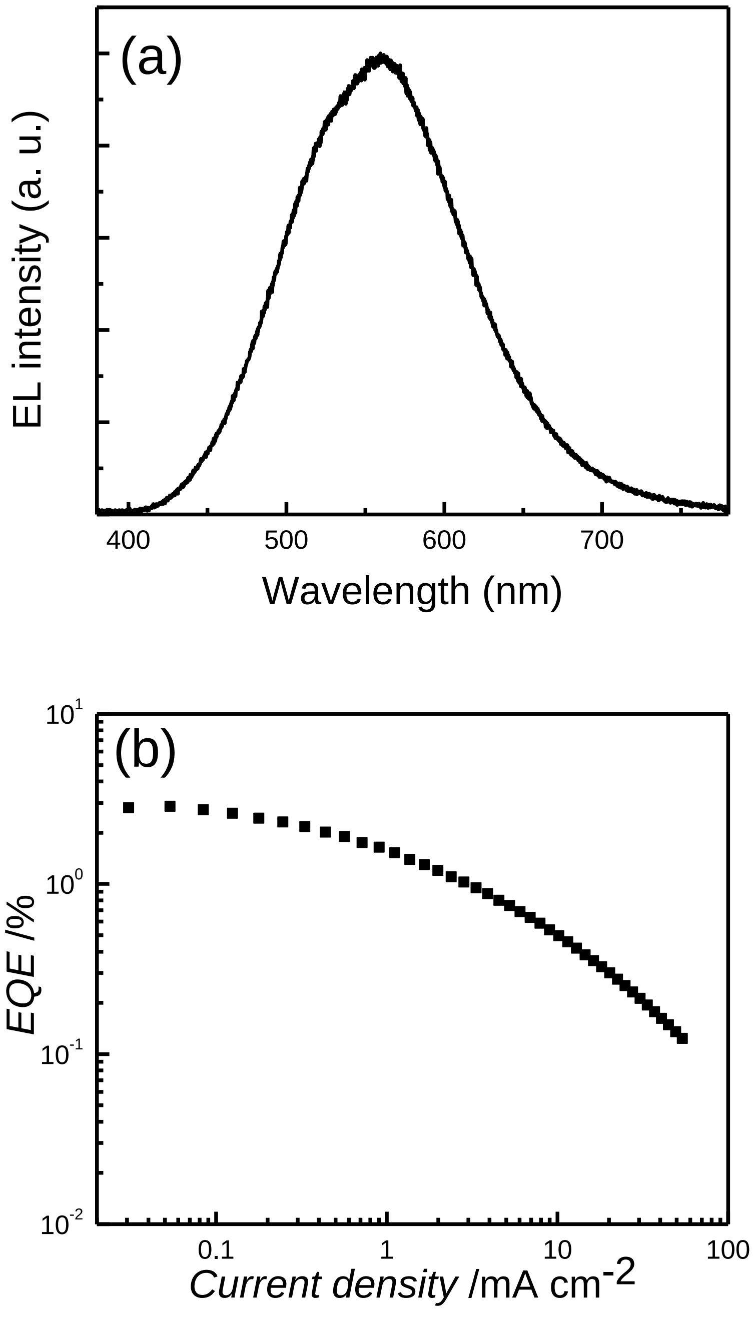

Fig. S26 (a) EL spectrum and (b) current efficiency-luminance characteristics of PYD2/DPEOP devices based on 10 wt% Cu(pop)(pz_2Bph_2).

18. Performances of the white OLEDs with 10 wt% Zn-1 and 1 wt% Cu-3

Table S2 Key performance parameters of the white PLEDs with 10 wt% **Zn-1** and 1 wt% **Cu-3** at different voltages

Voltage (V)	Luminance (cd m^{-2})	CE (cd A^{-1})	EQE (%)	CIE coordinates (x, y)	CRI
7	22	14.67	6.88	(0.44, 0.44)	80
9	300	9.38	4.27	(0.42, 0.44)	81
12	1540	3.67	1.62	(0.38, 0.44)	79
15	3150	1.66	0.73	(0.35, 0.44)	76

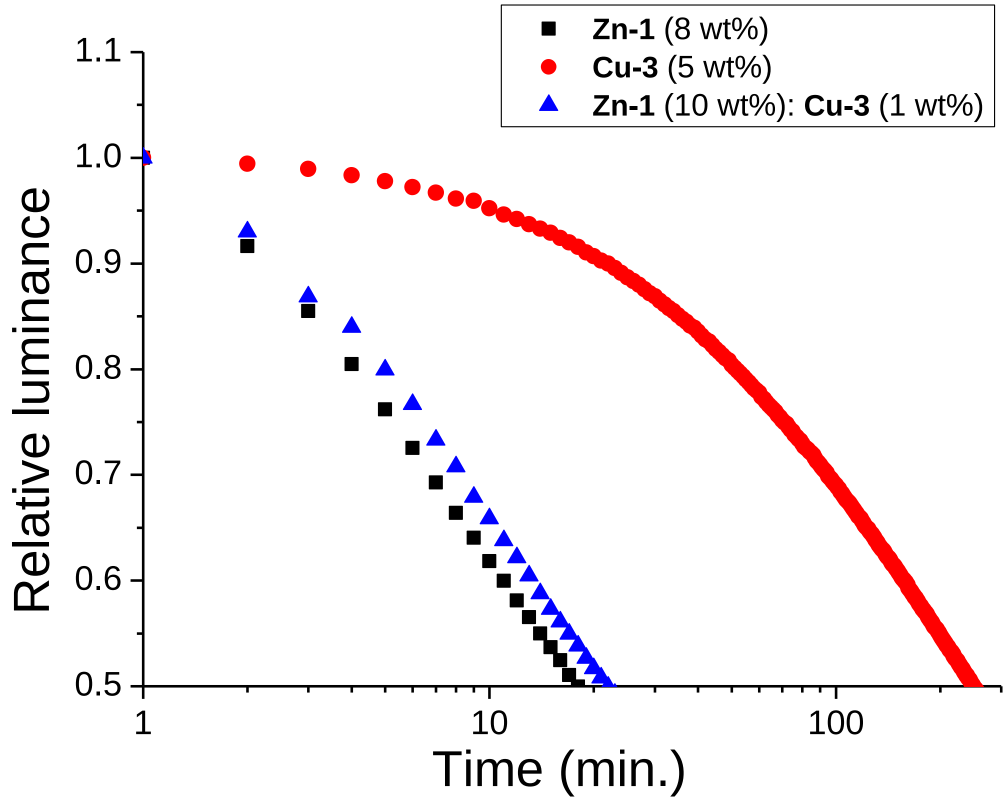

Fig. S27 Device lifetime of solution-processed OLEDs with **Zn-1** (8 wt%), **Cu-3** (5 wt%), as well as that of the white device with both **Zn-1** (10 wt%) and **Cu-3** (1 wt%).

19. Synthesis of Zn(II) complexes

Synethic procedures

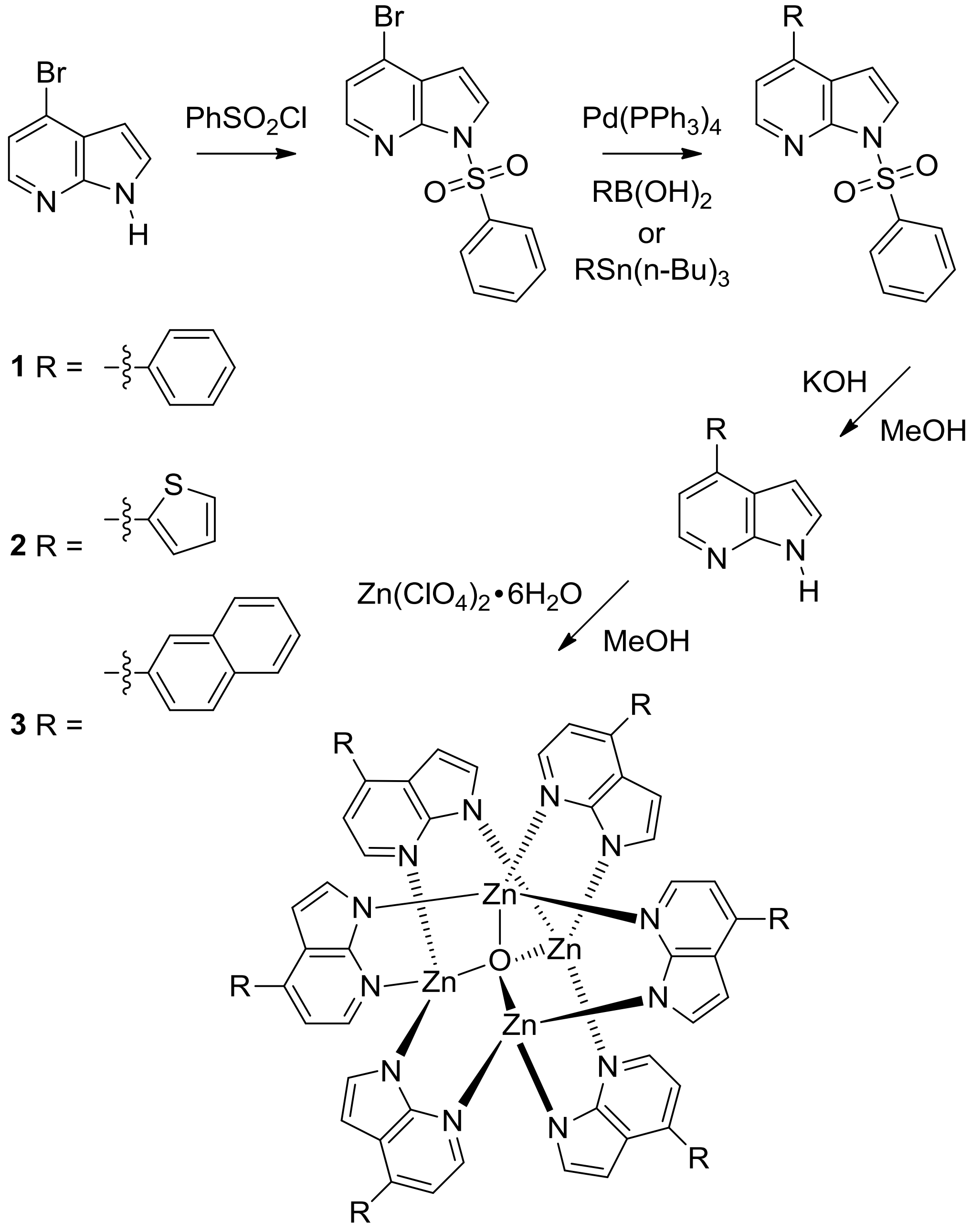

4-Bromo-1-(phenylsulfonyl)-1*H*-pyrrolo[2,3-*b*]pyridine. Sodium hydroxide (4.3 g, 107.5 mmol) and benzenesulfonyl chloride (5.5 mL, 43.1 mmol) were added to a solution of 4-bromo-1*H*-pyrrolo[2,3-*b*]pyridine (6.9 g, 35.0 mmol) and benzyltriethylammonium chloride (0.21 g, 0.9 mmol) in dichloromethane (150 mL) under ice-cooling. The reaction mixture was stirred at room temperature for 3 h. The resultant mixture was filtered through Celite and the filtrate was concentrated under reduced pressure. The crude product was purified by column chromatography over silica gel using *n*-hexane/dichloromethane mixture (1:1) as eluent to afford a white solid. The solid was further purified by recrystallization in *n*-hexane/ethyl acetate mixture to give a white solid. Yield: 8.62 g (73%). ^{1}H NMR (400 MHz, $CDCl_3$): δ 8.22 (d, *J* = 5.2 Hz, 1H), 8.21–8.12 (m, 2H), 7.79 (d, *J* = 4.0 Hz, 1H), 7.59 (t, *J* = 7.4 Hz, 1H), 7.49 (t, *J* = 7.7 Hz, 2H), 7.36 (d, *J* = 5.2 Hz, 1H), 6.65 (d, *J* = 4.0 Hz, 1H). ^{13}C NMR (101 MHz, $CDCl_3$): δ 146.83, 145.16, 138.11, 134.38, 129.19, 128.15, 127.00, 125.83, 124.44, 122.23, 105.13. EI-MS (+ve): *m*/*z* 338 $[M + H]^+$.

4-Phenyl-1-(phenylsulfonyl)-1*H*-pyrrolo[2,3-*b*]pyridine. Suzuki coupling was employed to synthesize the compound. A mixture of 4-bromo-1-(phenylsulfonyl)-1*H*-pyrrolo[2,3-*b*]pyridine (3.60 g, 10.68 mmol), phenylboronic acid (1.43 g, 11.73 mmol), tetrakis(triphenylphosphine)palladium(0) (0.62 g, 0.54 mmol) and potassium carbonate solution (2 M, 50 mL) in toluene (250 mL) was refluxed under nitrogen atmosphere for 24 h. After cooling, the toluene layer was washed with water (3 × 250 mL), brine solution (100 mL) and dried over anhydrous magnesium sulphate. The solvent was removed under reduced pressure and the crude product was purified by column chromatography over silica gel using *n*-hexane/dichloromethane mixture (1:1) as eluent to afford a yellow solid. Yield: 2.96 g (83%). ^{1}H NMR (400 MHz, $CDCl_3$): δ 8.48 (d, *J* = 5.0 Hz, 1H), 8.27–8.20 (m, 2H), 7.78 (d, *J* = 4.1 Hz, 1H), 7.62–7.54 (m, 3H), 7.54–7.41 (m, 5H), 7.24 (d, *J* = 5.0 Hz, 1H), 6.78 (d, *J* = 4.1 Hz, 1H). ^{13}C NMR (101 MHz, $CDCl_3$): δ 147.94, 145.31, 143.34, 138.51, 137.68, 134.15, 129.13, 129.10, 128.95, 128.57, 128.18, 126.57, 120.99, 118.20, 104.90. EI-MS (+ve): *m*/*z* 334 $[M]^+$.

1-(Phenylsulfonyl)-4-(thiophen-2-yl)-1*H*-pyrrolo[2,3-*b*]pyridine. Stille coupling was employed to synthesize the compound. A mixture of 4-bromo-1-(phenylsulfonyl)-1*H*-pyrrolo[2,3-*b*]pyridine (3.60 g, 10.68 mmol), tributyl(thiophen-2-yl)stannane (3.73 mL, 11.74 mmol) and tetrakis(triphenylphosphine)palladium(0) (0.62 g, 0.54 mmol) in anhydrous *N*,*N*-dimethylformamide (40 mL) was refluxed under nitrogen atmosphere for 24 h. After cooling, the resultant mixture was filtered through Celite and the filtrate was concentrated under reduced pressure. The crude product was purified by column chromatography over silica gel using *n*-hexane/dichloromethane mixture (1:1) as eluent to afford a yellow solid. Yield: 3.49 g (96%). ^{1}H NMR (400 MHz, CD_2Cl_2): δ 8.37 (d, *J* = 5.1 Hz, 1H), 8.25–8.16 (m, 2H), 7.82 (d, *J* = 4.1 Hz, 1H), 7.65–7.44 (m, 5H), 7.36 (d, *J* = 5.2 Hz, 1H), 7.19 (dd, *J* = 5.1, 3.7 Hz, 1H), 7.02 (d, *J* = 4.2 Hz, 1H). ^{13}C NMR (101 MHz, CD_2Cl_2): δ 148.47, 145.22, 140.04, 138.58, 135.84, 134.55, 129.40, 128.61, 128.45, 128.06, 127.60, 127.04, 119.63, 116.96, 105.30. EI-MS (+ve): *m*/*z* 340 $[M]^+$.

4-(Naphthalen-2-yl)-1-(phenylsulfonyl)-1*H*-pyrrolo[2,3-*b*]pyridine. Suzuki coupling was employed to synthesize the compound. A mixture of 4-bromo-1-(phenylsulfonyl)-1*H*-pyrrolo[2,3-*b*]pyridine (3.60 g, 10.68 mmol), naphthalen-2-ylboronic acid (2.02 g, 11.74 mmol), tetrakis(triphenylphosphine)palladium(0) (0.62 g, 0.54 mmol) and potassium carbonate solution (2 M, 50 mL) in toluene (250 mL) was refluxed under nitrogen atmosphere for 24 h. After cooling, the toluene layer was washed with water (3 × 250 mL), brine solution (100 mL) and dried over anhydrous magnesium sulphate. The solvent was removed under reduced pressure and the crude product was purified by column chromatography over silica gel using *n*-hexane/dichloromethane mixture (1:1) as eluent to afford a beige solid. Yield: 3.61 g (88%). ^{1}H NMR (400 MHz, CD_2Cl_2): δ 8.49 (d, *J* = 5.0 Hz, 1H), 8.29–8.20 (m, 2H), 8.08 (s, 1H), 7.98 (d, *J* = 8.5 Hz, 1H), 7.96–7.88 (m, 2H), 7.83 (d, *J* = 4.1 Hz, 1H), 7.71 (dd, *J* = 8.5, 1.7 Hz, 1H), 7.64–7.47 (m, 5H), 7.37 (d, *J* = 5.0 Hz, 1H), 6.87 (d, *J* = 4.1 Hz, 1H). ^{13}C NMR (101 MHz, CD_2Cl_2): δ 148.24, 145.37, 143.45, 138.70, 135.32, 134.51, 133.76, 133.62, 129.41, 129.08, 128.69, 128.45, 128.28, 128.07, 127.23, 127.07, 126.99, 126.36, 121.37, 118.71, 105.15. EI-MS (+ve): *m*/*z* 384 $[M]^+$.

4-Phenyl-1*H*-pyrrolo[2,3-*b*]pyridine. A mixture of 4-phenyl-1-(phenylsulfonyl)-1*H*-pyrrolo[2,3-*b*]pyridine (1.10 g, 3.29 mmol) and 10 % potassium hydroxide (20 mL) in methanol (200 mL) was stirred at room temperature for 24 h. The methanol in the reaction mixture was removed under reduced pressure. The resultant mixture was diluted with water (30 mL) and extracted with dichloromethane (3 × 50 mL). The combined organic layer was dried over anhydrous magnesium sulphate. The solvent was removed under reduced pressure and the crude product was purified by column chromatography over silica gel using dichloromethane/methanol mixture (9:1) as eluent to afford a pink solid. Yield: 0.59 g (92%). ^{1}H NMR (400 MHz, CD_2Cl_2): δ 10.76 (s, 1H), 8.40 (d, *J* = 5.0 Hz, 1H), 7.86–7.76 (m, 2H), 7.62–7.51 (m, 2H), 7.51–7.43 (m, 2H), 7.22 (d, *J* = 4.9 Hz, 1H), 6.74 (d, *J* = 3.4 Hz, 1H). ^{13}C NMR (101 MHz, $[D_6]$DMSO): δ 149.15, 142.93, 140.26, 138.49, 128.96, 128.33, 128.20, 126.60, 117.23, 114.18, 98.95. EI-MS (+ve): *m*/*z* 194 $[M]^+$.

4-(Thiophen-2-yl)-1*H*-pyrrolo[2,3-*b*]pyridine. A mixture of 1-(phenylsulfonyl)-4-(thiophen-2-yl)-1*H*-pyrrolo[2,3-*b*]pyridine (1.12 g, 3.29 mmol) and 10% potassium hydroxide (20 mL) in methanol (200 mL) was stirred at room temperature for 24 h. The methanol in the reaction mixture was removed under reduced pressure.

The resultant mixture was diluted with water (30 mL) and extracted with dichloromethane (3 × 50 mL). The combined organic layer was dried over anhydrous magnesium sulphate. The solvent was removed under reduced pressure and the crude product was purified by column chromatography over silica gel using dichloromethane/methanol mixture (9:1) as eluent to afford a yellow solid. Yield: 0.55 g (83%). ^{1}H NMR (400 MHz, CD_2Cl_2): δ 10.82 (s, 1H), 8.34 (t, *J* = 4.7 Hz, 1H), 7.70 (td, *J* = 4.1, 1.1 Hz, 1H), 7.60–7.44 (m, 2H), 7.34 (t, *J* = 4.7 Hz, 1H), 7.27–7.18 (m, 1H), 6.93 (t, *J* = 3.7 Hz, 1H). ^{13}C NMR (101 MHz, [D$_6$]DMSO): δ 149.50, 142.81, 140.75, 132.75, 128.40, 127.55, 126.91, 126.77, 115.58, 112.60, 99.43. EI-MS (+ve): *m*/*z* 200 [M]$^+$.

4-(Naphthalen-2-yl)-1*H*-pyrrolo[2,3-*b*]pyridine. A mixture of 4-(naphthalen-2-yl)-1-(phenylsulfonyl)-1*H*-pyrrolo[2,3-*b*]pyridine (1.26 g, 3.28 mmol) and 10% potassium hydroxide (20 mL) in methanol (200 mL) was stirred at room temperature for 24 h. The methanol in the reaction mixture was removed under reduced pressure. The resultant mixture was diluted with water (30 mL) and extracted with dichloromethane (3 × 50 mL). The combined organic layer was dried over anhydrous magnesium sulphate. The solvent was removed under reduced pressure and the crude product was purified by column chromatography over silica gel using dichloromethane/methanol mixture (9:1) as eluent to afford a beige solid. Yield: 0.60 g (75%). ^{1}H NMR (400 MHz, CD_2Cl_2): δ 9.78 (s, 1H), 8.41 (d, *J* = 5.0 Hz, 1H), 8.26 (s, 1H), 8.02 (d, *J* = 8.5 Hz, 1H), 8.00–7.88 (m, 3H), 7.60–7.53 (m, 2H), 7.47 (t, *J* = 2.9 Hz, 1H), 7.33 (d, *J* = 5.0 Hz, 1H), 6.81 (dd, *J* = 3.5, 1.9 Hz, 1H). ^{13}C NMR (101 MHz, [D$_6$]DMSO): δ 149.23, 142.97, 140.13, 135.97, 133.15, 132.66, 128.47, 128.40, 127.56, 127.28, 126.74, 126.59, 126.53, 126.13, 117.42, 114.51, 99.13. EI-MS (+ve): *m*/*z* 244 [M]$^+$.

[Zn_4O(AID-4-Phenyl)$_6$] (Zn-1). A solution of zinc perchlorate hexahydrate (0.37 g, 1 mmol) in methanol (30 mL) was added dropwise to a hot solution of 4-phenyl-1*H*-pyrrolo[2,3-*b*]pyridine (0.29 g, 1.5 mmol) and triethylamine (1 mL) in methanol (20 mL). The reaction mixture was stirred at 60 ^{o}C for 3 h. After cooling, the resultant mixture was filtered and the residue was washed with hot methanol (3 × 10 mL). The solid collected was dissolved in dichloromethane (20 mL) and filtered through Celite. The filtrate was concentrated under reduced pressure and added dropwise to methanol (10 mL). The white solid precipitated was collected by centrifugation and dried under vacuum at 40 ^{o}C for 24 h. Yield: 0.25 g (71%). ^{1}H NMR (500 MHz, $CDCl_3$): δ 8.06–7.80 (m, 6H), 7.80–7.54 (m, 18H), 7.54–7.27 (m, 18H), 7.10–6.99 (m, 6H), 6.85–6.67 (m, 6H). ^{13}C NMR (126 MHz, $CDCl_3$): δ 156.30–155.37 (m), 144.76–143.33 (m), 139.80–137.56 (m), 129.85–127.79 (m), 124.36–123.23 (m), 113.24–112.11 (m), 101.29–100.27 (m). FAB-MS (+ve): *m*/*z* 1436 [M]$^+$. Elemental analyses calcd. for $C_{78}H_{54}N_{12}OZn_4 \cdot CH_2Cl_2$: C 62.35, H 3.71, N 11.04; found: C 62.34, H 3.91, N 10.78.

[Zn_4O(AID-4-Thiophen-2-yl)$_6$] (Zn-2). A solution of zinc perchlorate hexahydrate (0.37 g, 1 mmol) in methanol (30 mL) was added dropwise to a hot solution of 4-(thiophen-2-yl)-1*H*-pyrrolo[2,3-*b*]pyridine (0.30 g, 1.5 mmol) and triethylamine (1 mL) in methanol (20 mL). The reaction mixture was stirred at 60 ^{o}C for 3 h. After cooling, the resultant mixture was filtered and the residue was washed with hot methanol (3 × 10 mL). The solid collected was dissolved in dichloromethane (20 mL) and filtered through Celite. The filtrate was concentrated under reduced pressure and added dropwise to methanol (10 mL). The yellow solid precipitated was collected by centrifugation and dried under vacuum at 40 ^{o}C for 24 h. Yield: 0.31 g (84%). ^{1}H NMR (500 MHz, $CDCl_3$): δ 8.00–7.34 (m, 24H), 7.23–7.06 (m, 12H), 7.06–6.89 (m, 6H). ^{13}C NMR (126 MHz, $CDCl_3$): δ 156.58–155.79 (m), 142.07–141.15 (m), 139.95–137.28 (m), 136.91–135.60 (m), 128.78–126.39 (m), 122.70–121.39 (m), 111.86–110.88 (m), 101.87–100.71 (m). FAB-MS (+ve): *m*/*z* 1473 [M + H]$^+$. Elemental analyses calcd. for $C_{66}H_{42}N_{12}OS_6Zn_4$: C 53.81, H 2.87, N 11.41; found: C 53.82, H 3.09, N 11.34.

[Zn_4O(AID-4-Naphthalen-2-yl)$_6$] (Zn-3). A solution of zinc perchlorate hexahydrate (0.37 g, 1 mmol) in methanol (30 mL) was added dropwise to a hot solution of 4-(naphthalen-2-yl)-1*H*-pyrrolo[2,3-*b*]pyridine (0.37 g, 1.5 mmol) and triethylamine (1 mL) in methanol (20 mL). The reaction mixture was stirred at 60 ^{o}C for 3 h. After cooling, the resultant mixture was filtered and the residue was washed with hot methanol (3 × 10 mL). The solid collected was dissolved in dichloromethane (20 mL) and filtered through Celite. The filtrate was concentrated under reduced pressure and added dropwise to methanol (10 mL). The yellow solid precipitated was collected by

centrifugation and dried under vacuum at 40 °C for 24 h. Yield: 0.26 g (60%). ^{1}H NMR (500 MHz, $CDCl_3$): δ 8.37–8.18 (m, 6H), 8.17–7.60 (m, 36H), 7.59–7.43 (m, 12H), 7.24–7.12 (m, 6H), 7.04–6.81 (m, 6H). ^{13}C NMR (126 MHz, $CDCl_3$): δ 156.29–155.57 (m), 144.54–143.56 (m), 139.78–137.63 (m), 137.17–136.29 (m), 134.03–132.58 (m), 129.63–125.40 (m), 124.54–123.44 (m), 113.56–112.57 (m), 101.42–100.39 (m). FAB-MS (+ve): *m*/*z* 1737 $[M]^{+}$. Elemental analyses calcd. for $C_{102}H_{66}N_{12}OZn_4$: C 70.52, H 3.83, N 9.67; found: C 70.57, H 4.13, N 9.33.

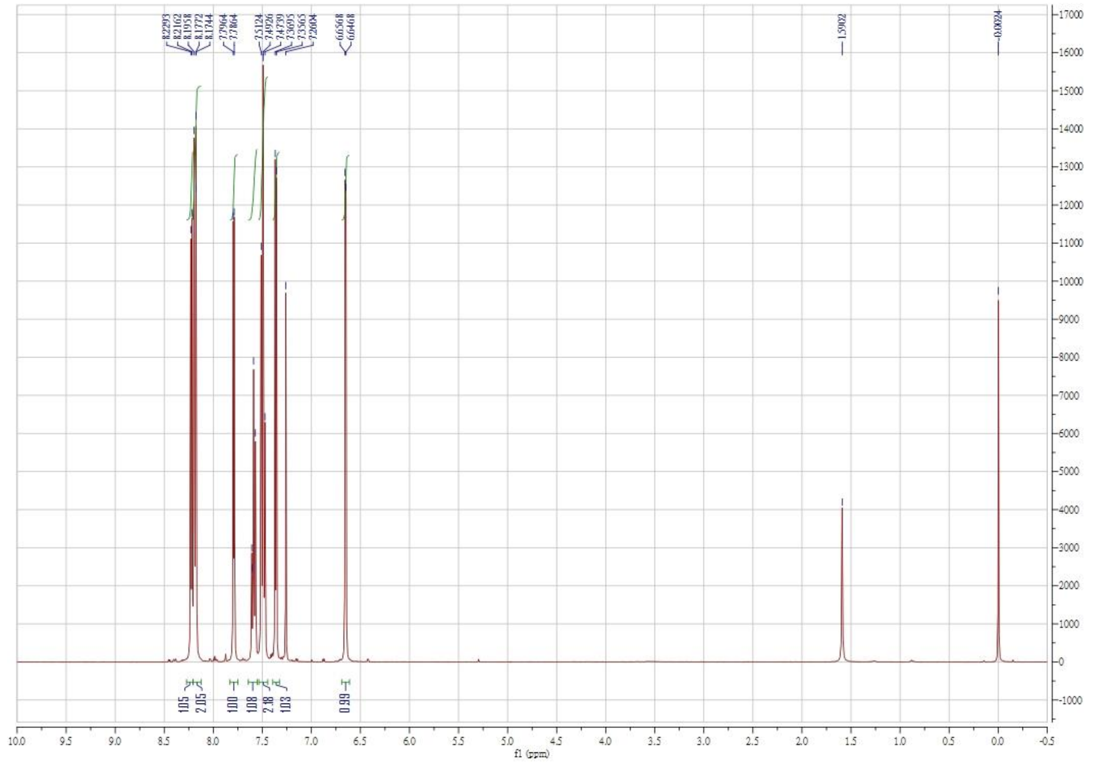

Fig. S28 ^{1}H NMR spectrum of 4-bromo-1-(phenylsulfonyl)-1*H*-pyrrolo[2,3-*b*]pyridine in $CDCl_3$.

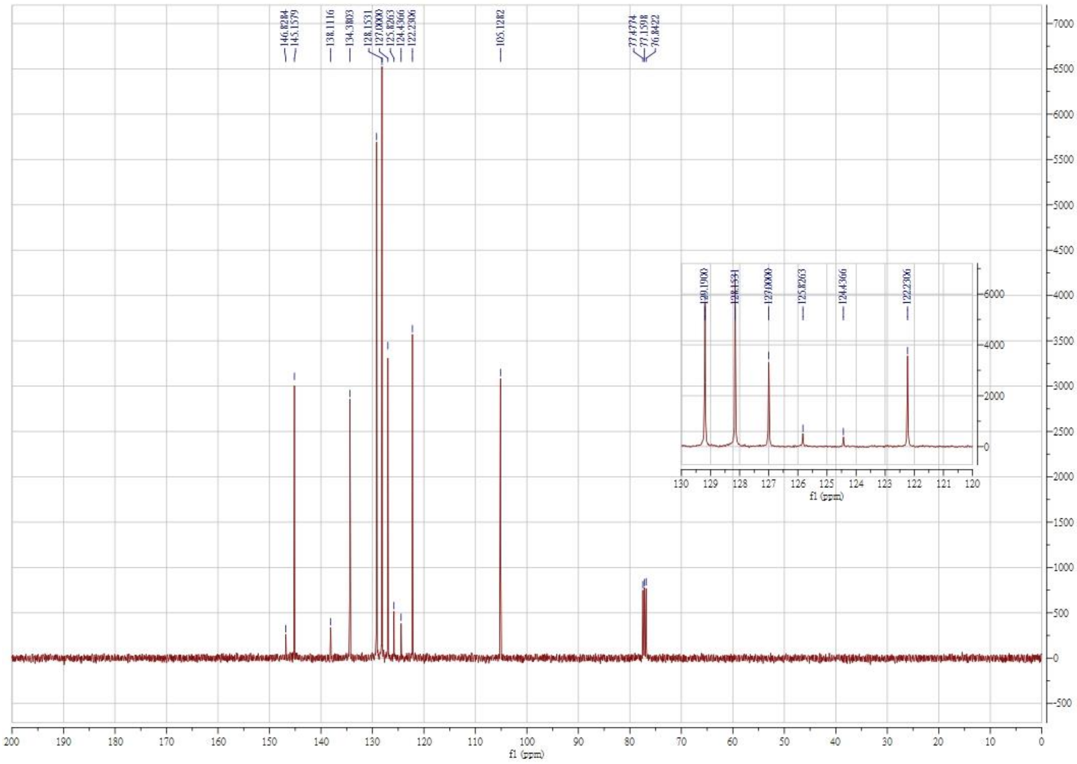

Fig. S29 ^{13}C NMR spectrum of 4-bromo-1-(phenylsulfonyl)-1*H*-pyrrolo[2,3-*b*]pyridine in $CDCl_3$.

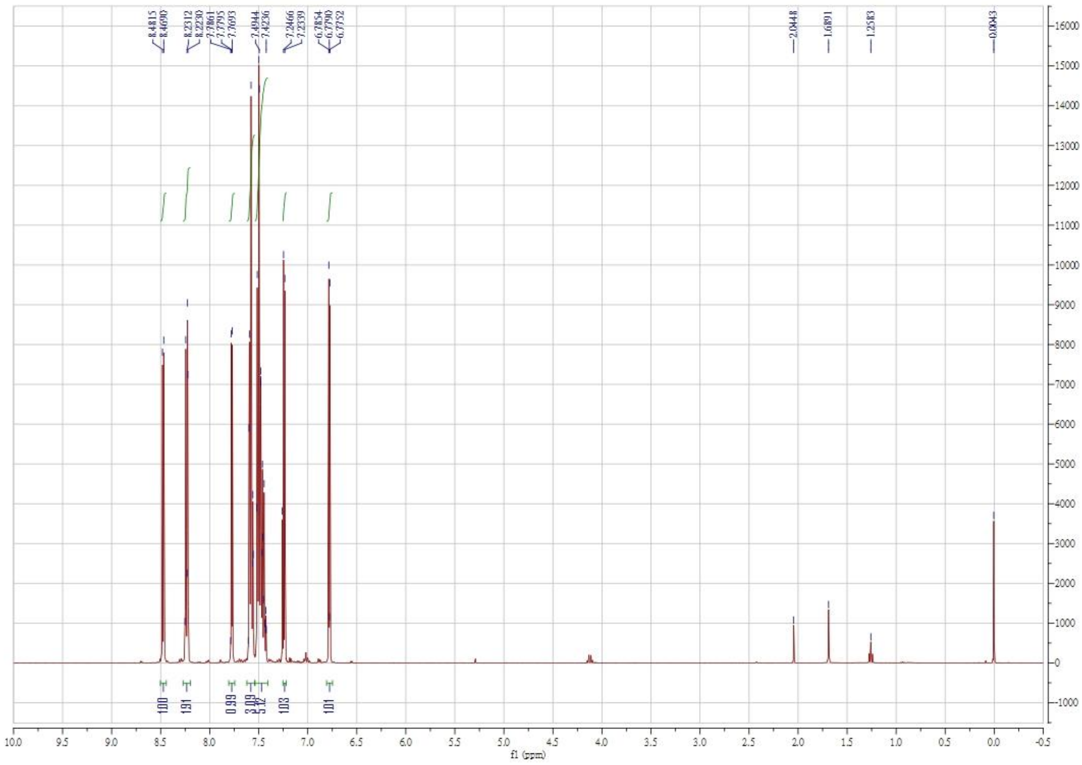

Fig. S30 ^{1}H NMR spectrum of 4-phenyl-1-(phenylsulfonyl)-1*H*-pyrrolo[2,3-*b*]pyridine in $CDCl_3$.

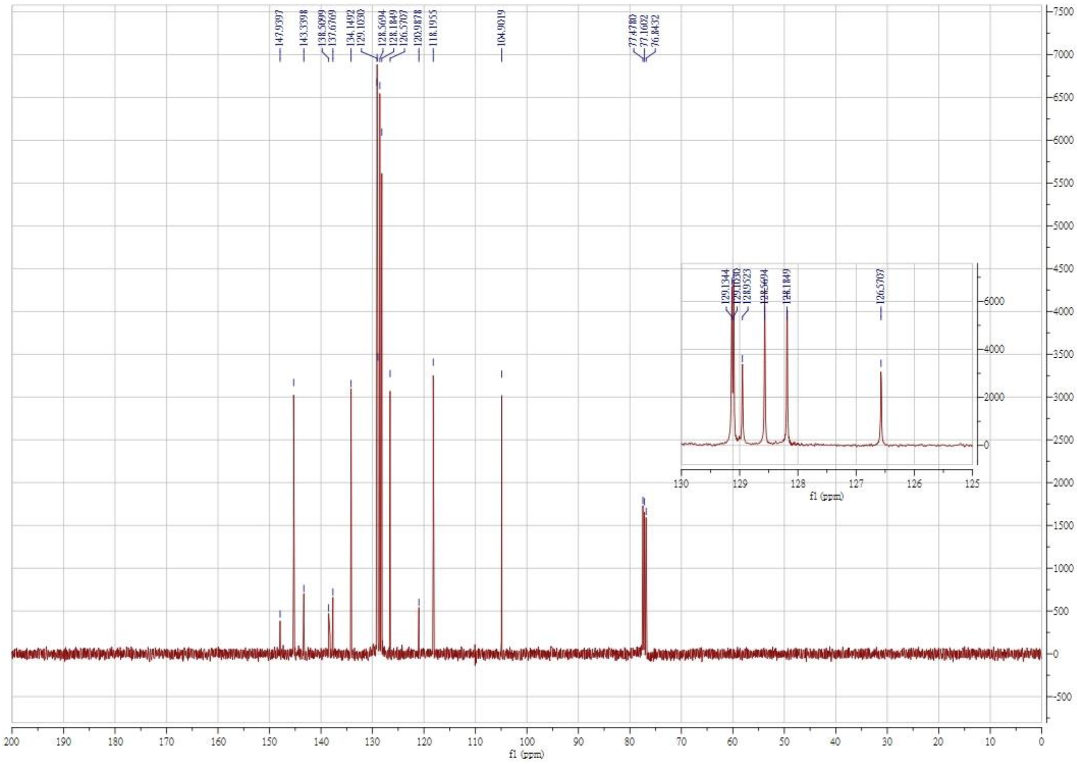

Fig. S31 ^{13}C NMR spectrum of 4-phenyl-1-(phenylsulfonyl)-1*H*-pyrrolo[2,3-*b*]pyridine in $CDCl_3$.

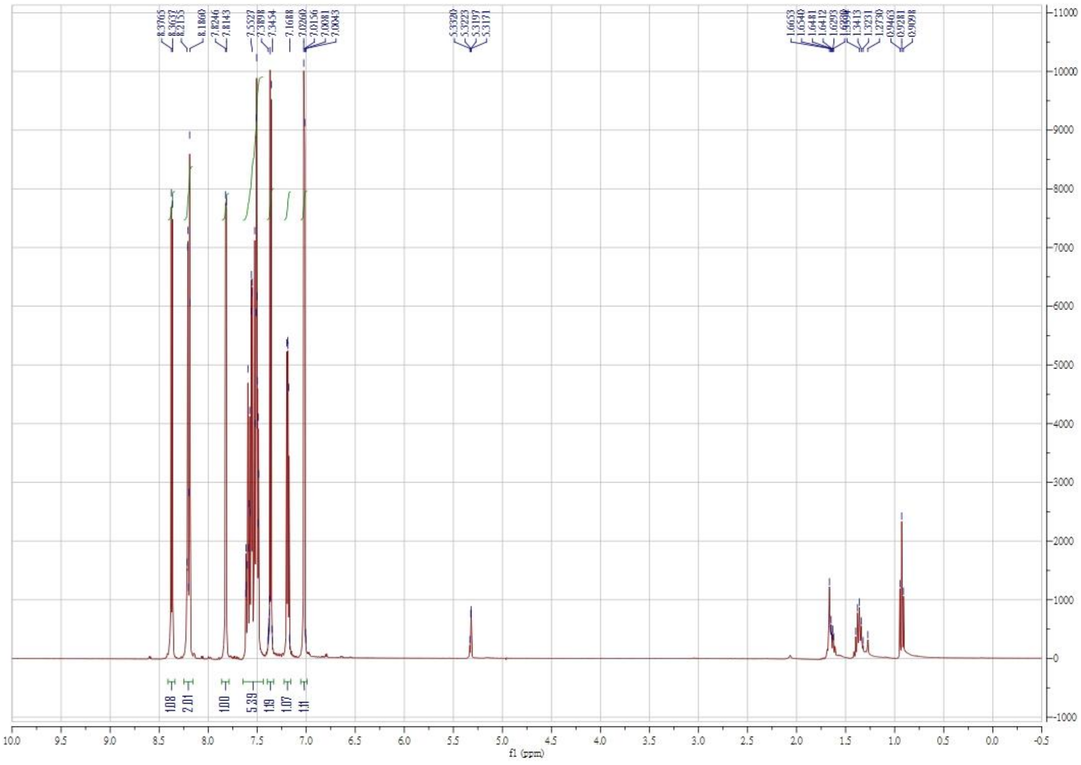

Fig. S32 ^{1}H NMR spectrum of 1-(phenylsulfonyl)-4-(thiophen-2-yl)-1*H*-pyrrolo[2,3-*b*]pyridine in CD_2Cl_2.

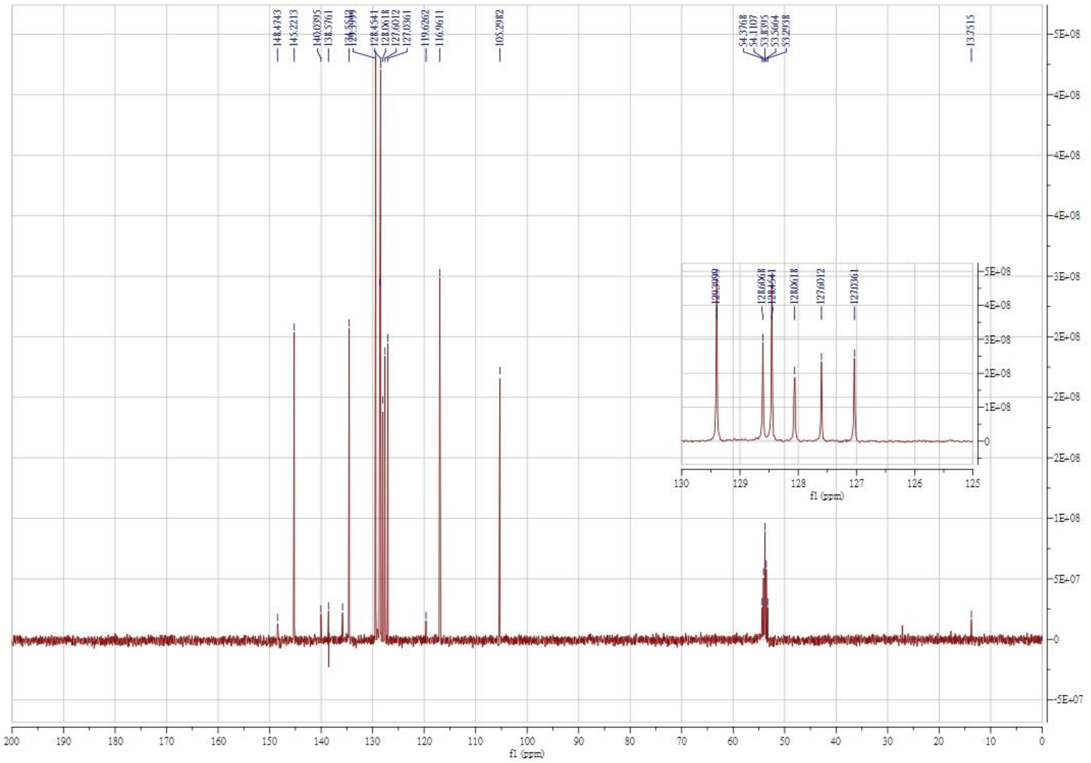

Fig. S33 ^{13}C NMR spectrum of 1-(phenylsulfonyl)-4-(thiophen-2-yl)-1*H*-pyrrolo[2,3-*b*]pyridine in CD_2Cl_2.

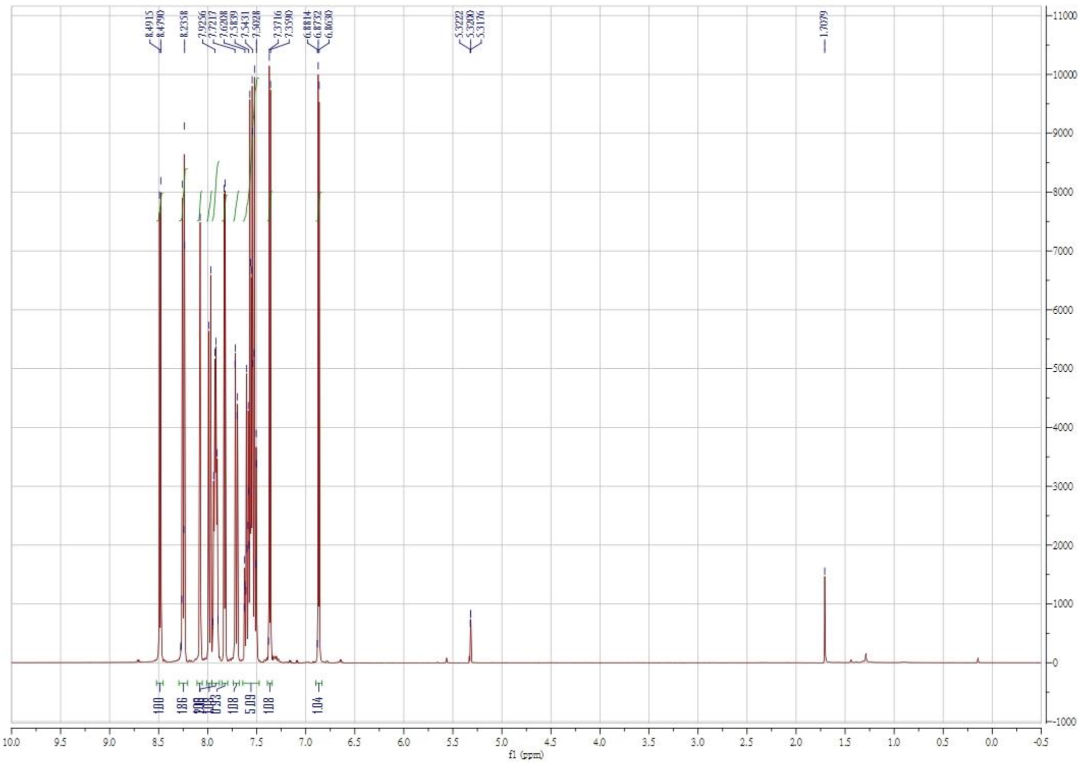

Fig. S34 ^{1}H NMR spectrum of 4-(naphthalen-2-yl)-1-(phenylsulfonyl)-1*H*-pyrrolo[2,3-*b*]pyridine in CD_2Cl_2.

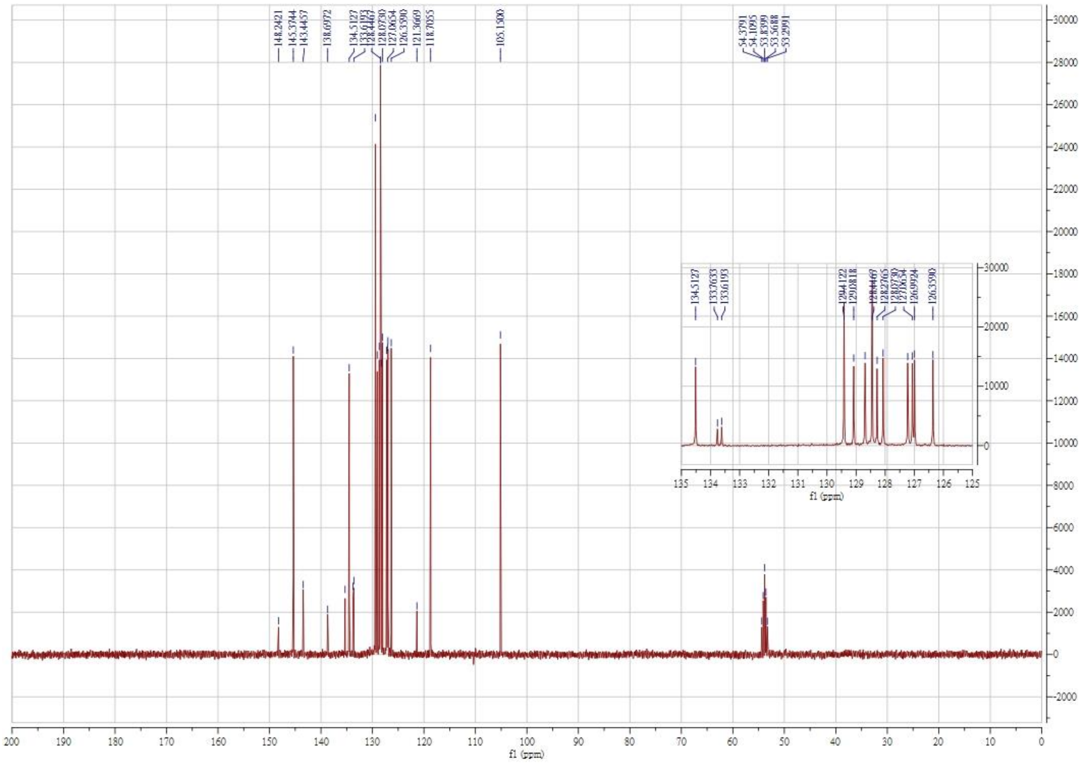

Fig. S35 ^{13}C NMR spectrum of 4-(naphthalen-2-yl)-1-(phenylsulfonyl)-1*H*-pyrrolo[2,3-*b*]pyridine in CD_2Cl_2.

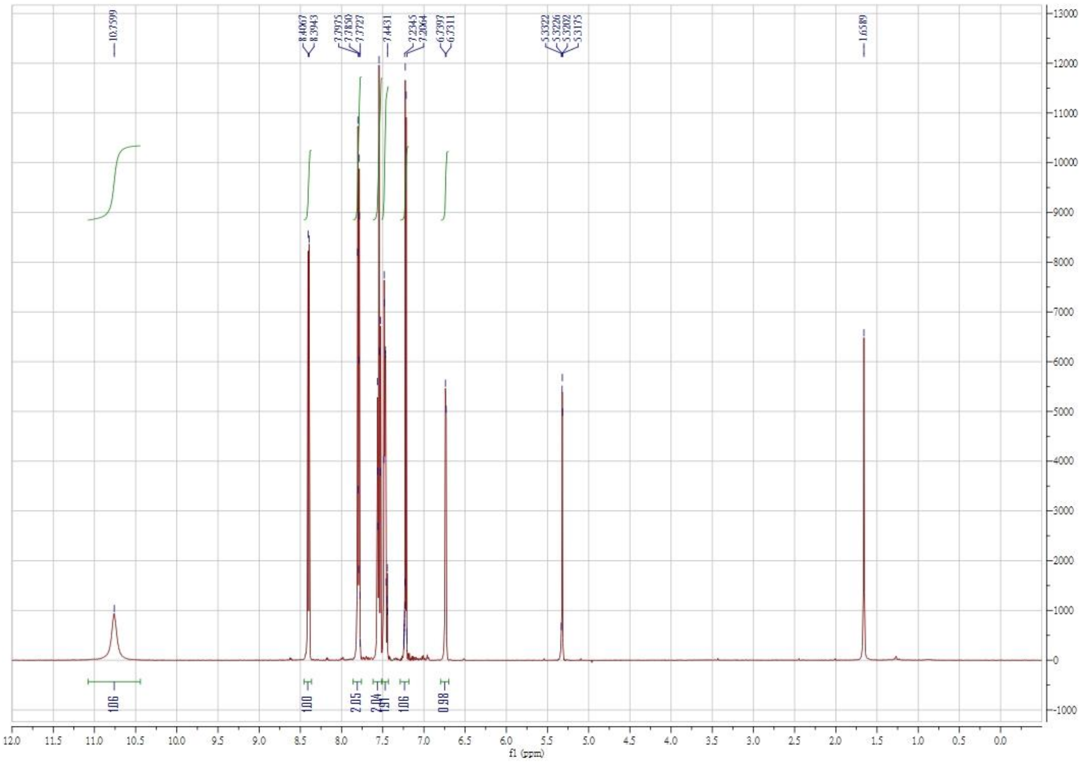

Fig. S36 ^{1}H NMR spectrum of 4-phenyl-1*H*-pyrrolo[2,3-*b*]pyridine in CD_2Cl_2.

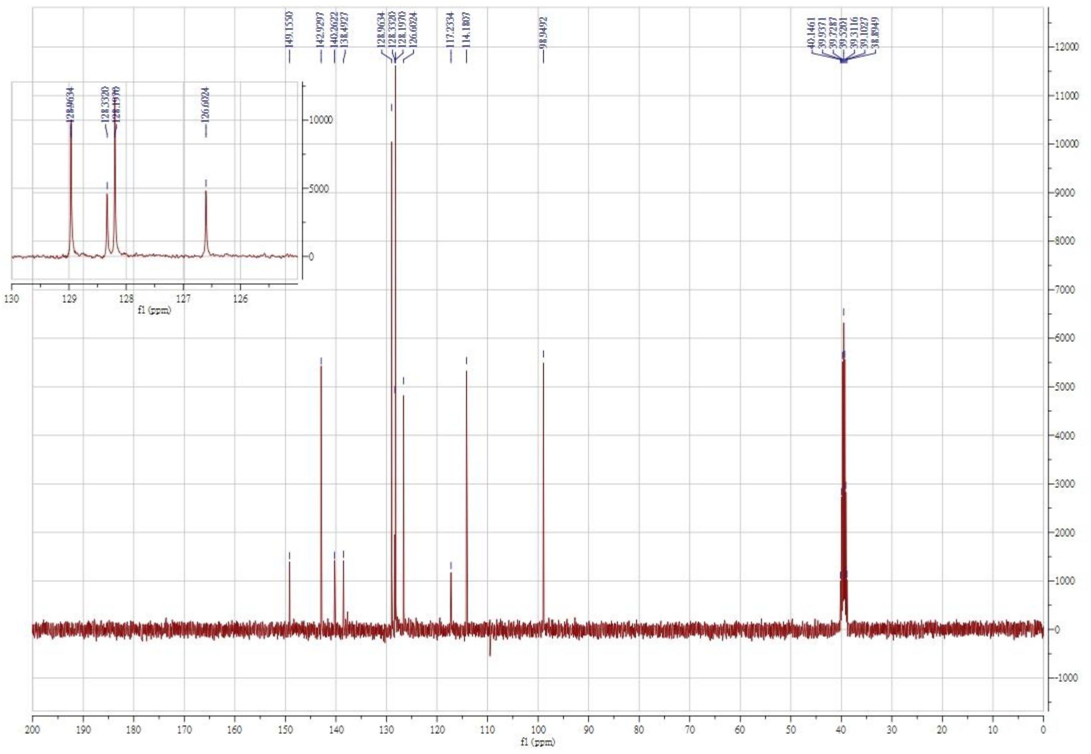

Fig. S37 ^{13}C NMR spectrum of 4-phenyl-1*H*-pyrrolo[2,3-*b*]pyridine in [D$_6$]DMSO.

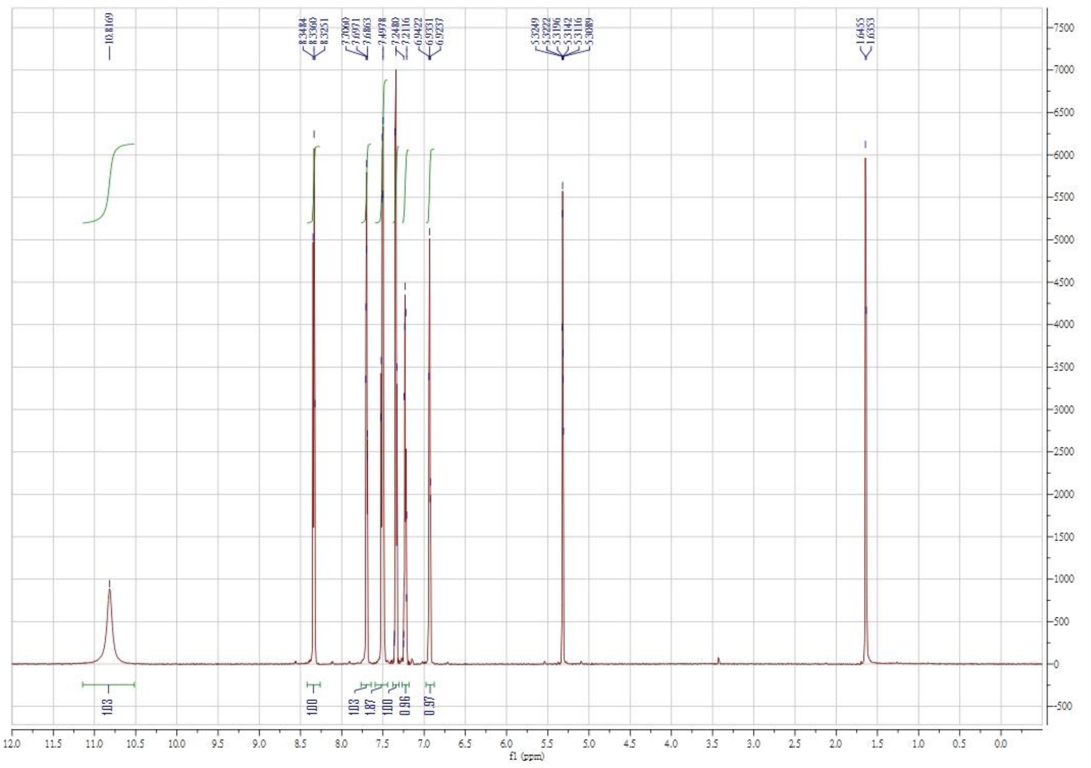

Fig. S38 ^{1}H NMR spectrum of 4-(thiophen-2-yl)-1*H*-pyrrolo[2,3-*b*]pyridine in CD_2Cl_2.

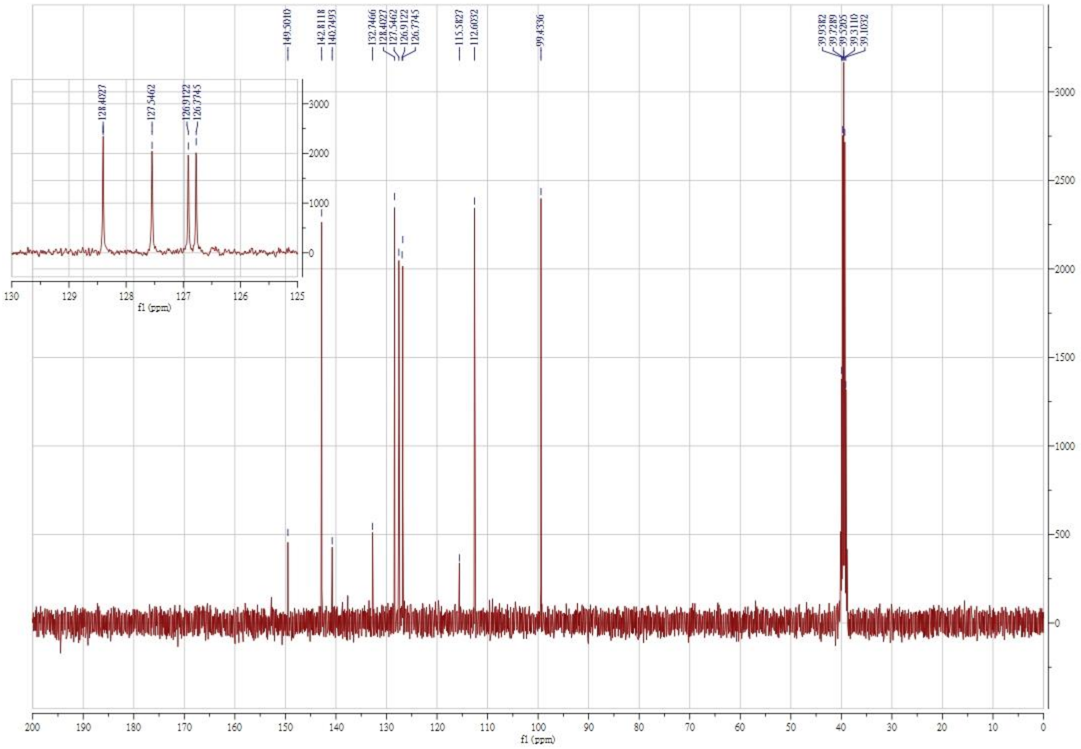

Fig. S39 ^{13}C NMR spectrum of 4-(thiophen-2-yl)-1*H*-pyrrolo[2,3-*b*]pyridine in [D$_6$]DMSO.

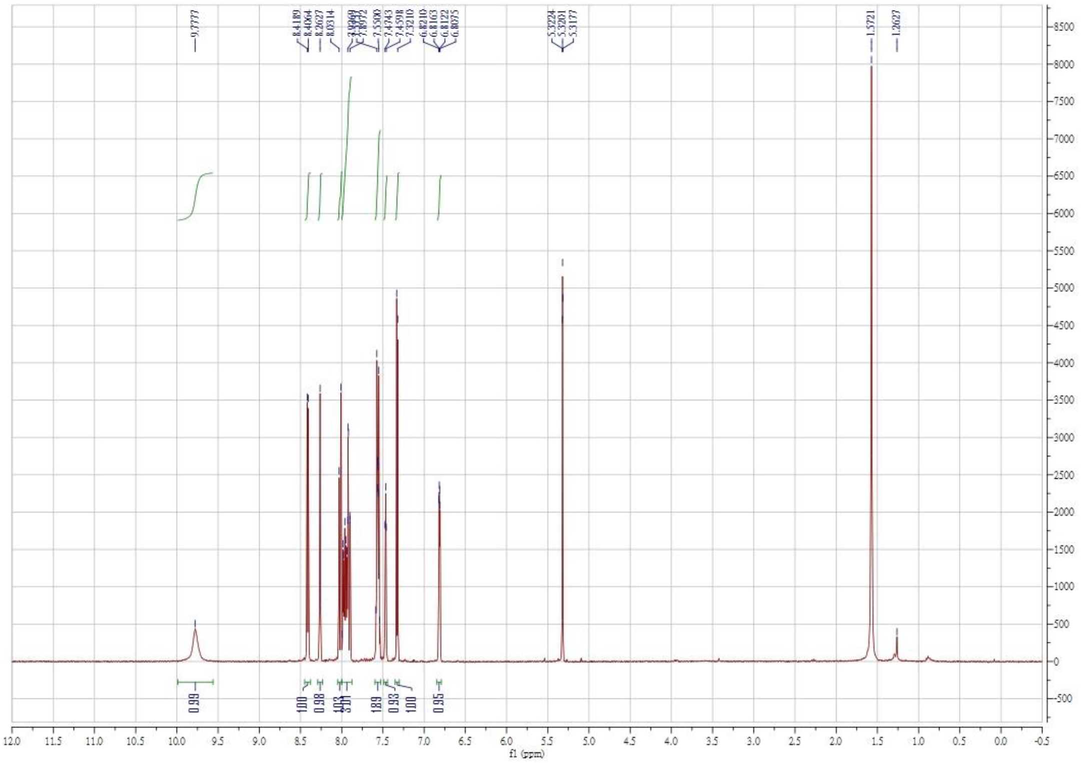

Fig. S40 ^{1}H NMR spectrum of 4-(naphthalen-2-yl)-1*H*-pyrrolo[2,3-*b*]pyridine in CD_2Cl_2.

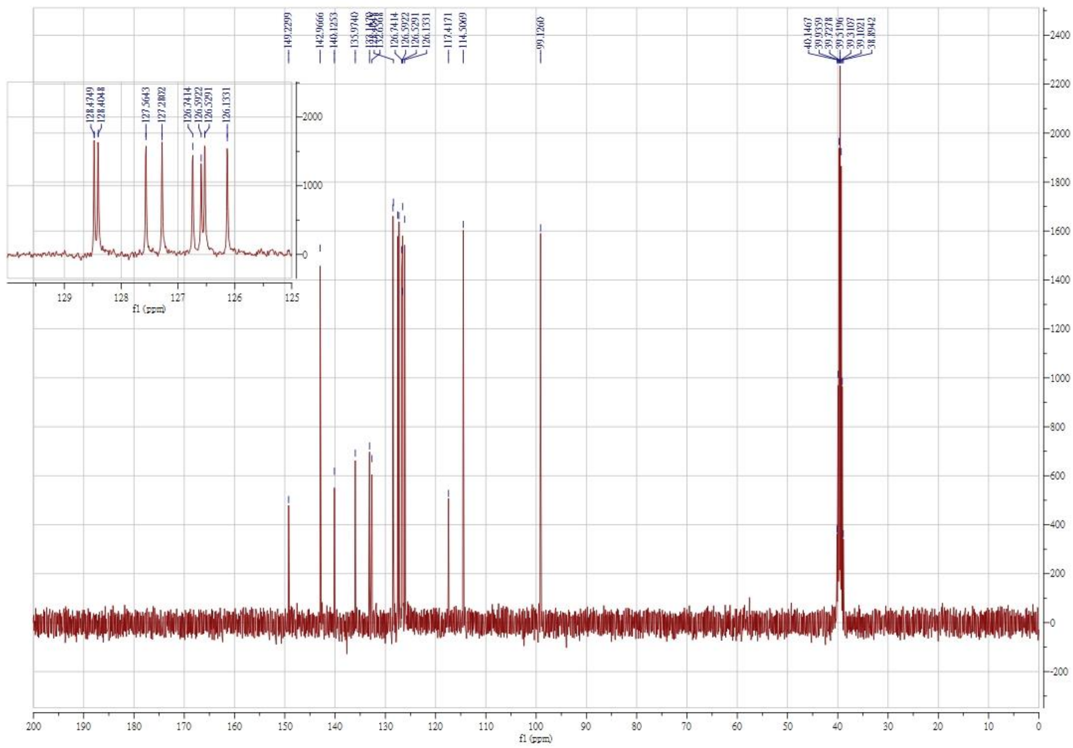

Fig. S41 ^{13}C NMR spectrum of 4-(naphthalen-2-yl)-1*H*-pyrrolo[2,3-*b*]pyridine in [D_6]DMSO.

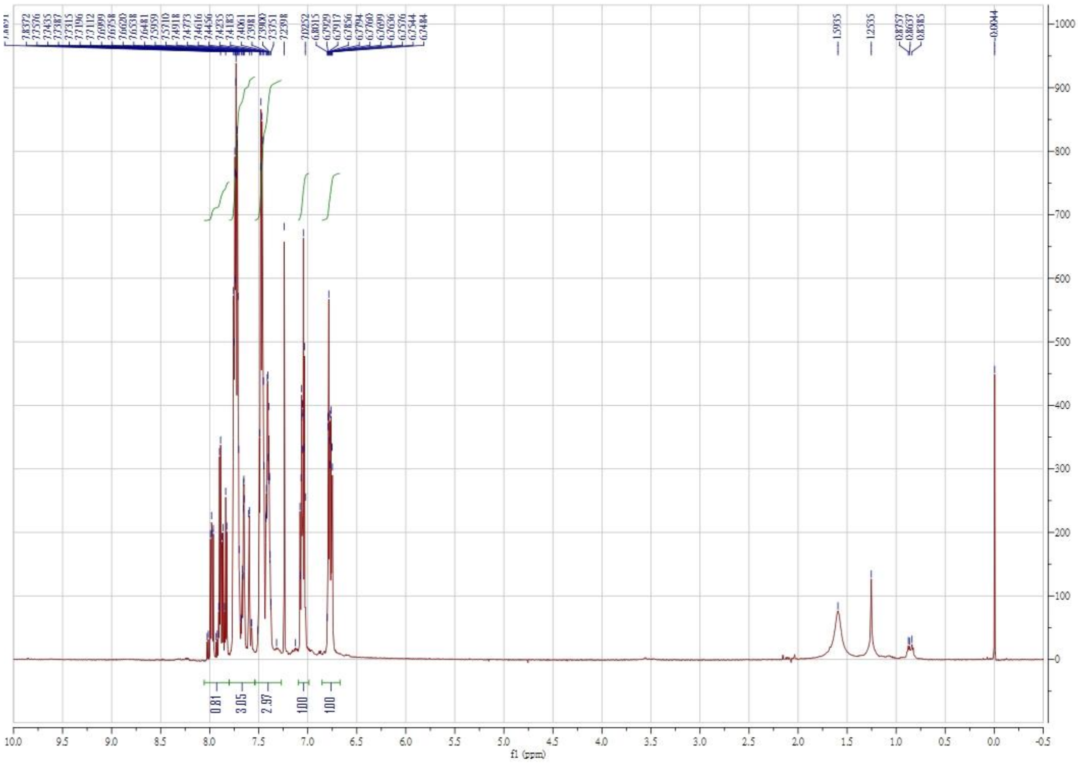

Fig. S42 ^{1}H NMR spectrum of [Zn_4O(AID-4-Phenyl)$_6$] (**Zn-1**) in $CDCl_3$.

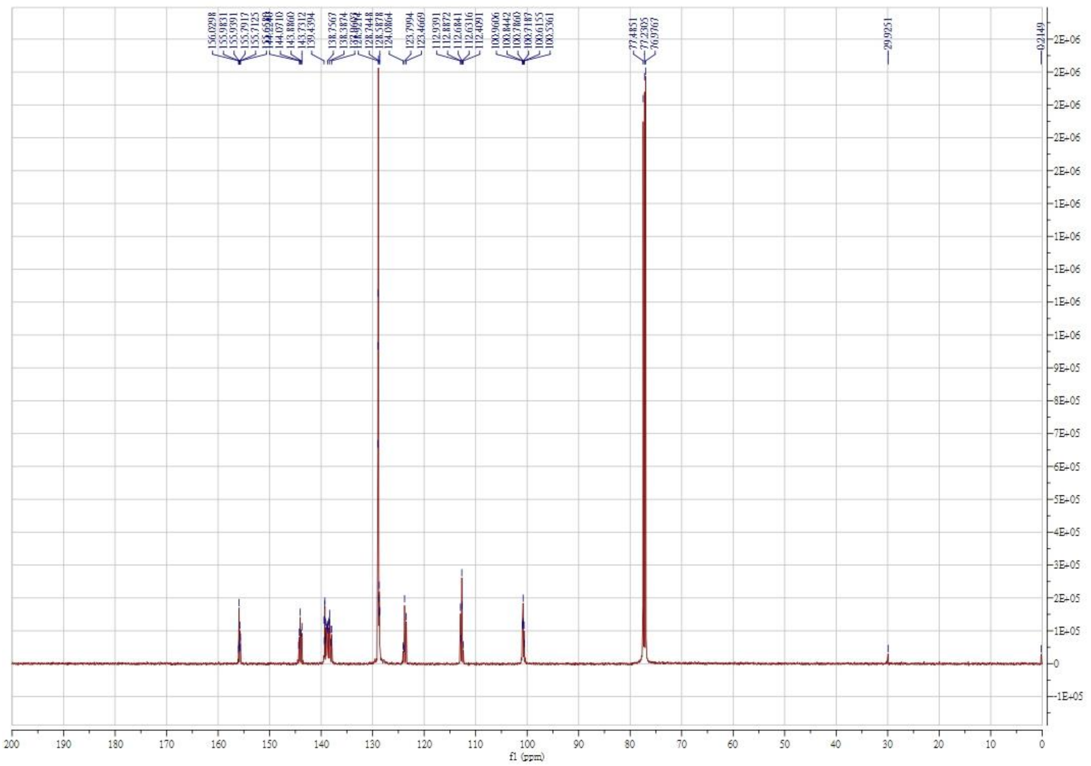

Fig. S43 ^{13}C NMR spectrum of $[Zn_4O(AID\text{-}4\text{-}Phenyl)_6]$ (**Zn-1**) in $CDCl_3$.

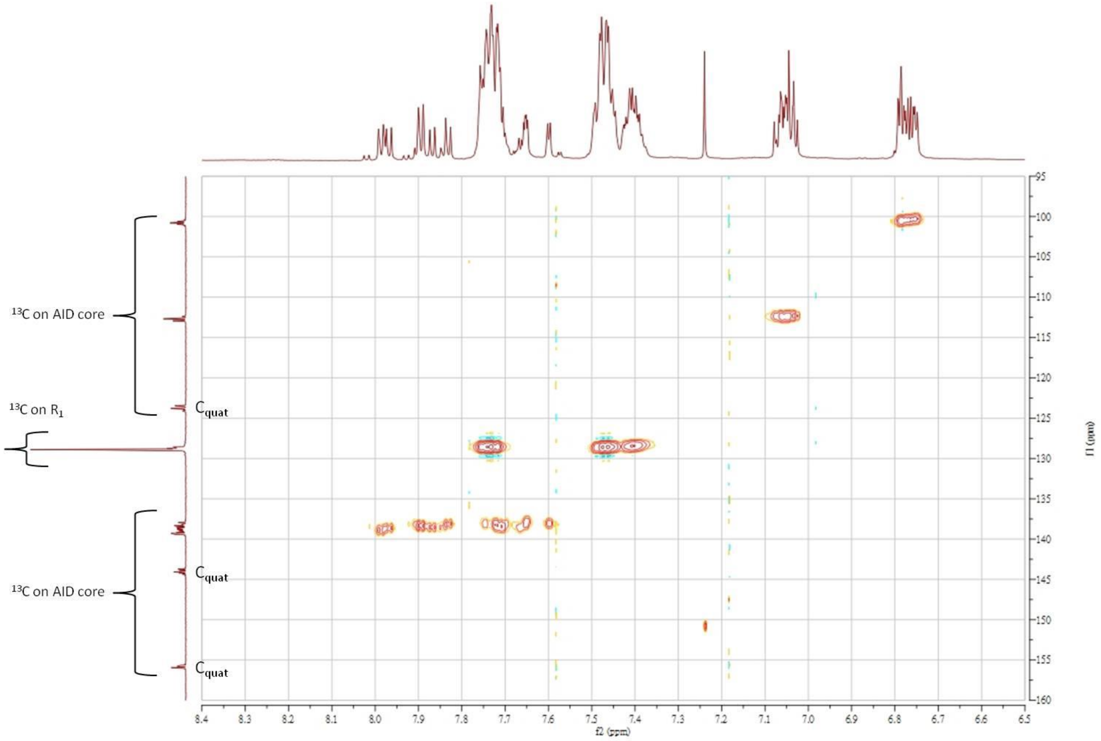

Fig. S44 ^{1}H-^{13}C HSQC NMR spectrum of $[Zn_4O(AID\text{-}4\text{-}Phenyl)_6]$ (**Zn-1**) in $CDCl_3$.

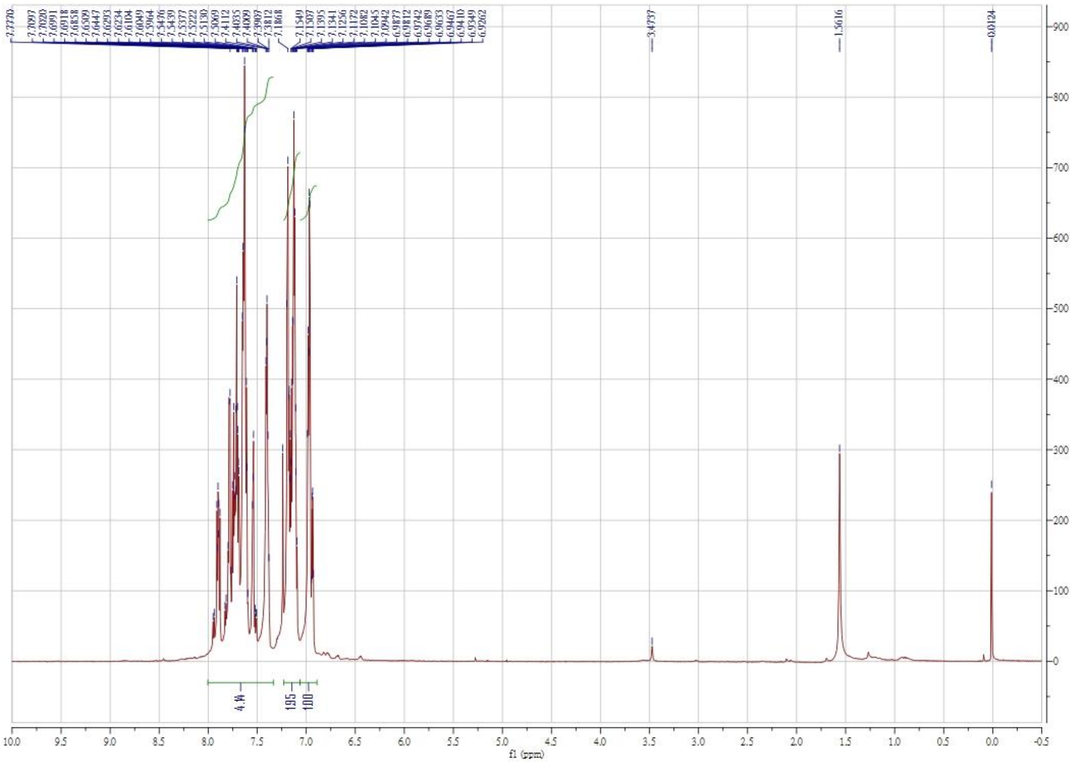

Fig. S45 ^{1}H NMR spectrum of $[Zn_4O(AID\text{-}4\text{-}Thiophen\text{-}2\text{-}yl)_6]$ (**Zn-2**) in $CDCl_3$.

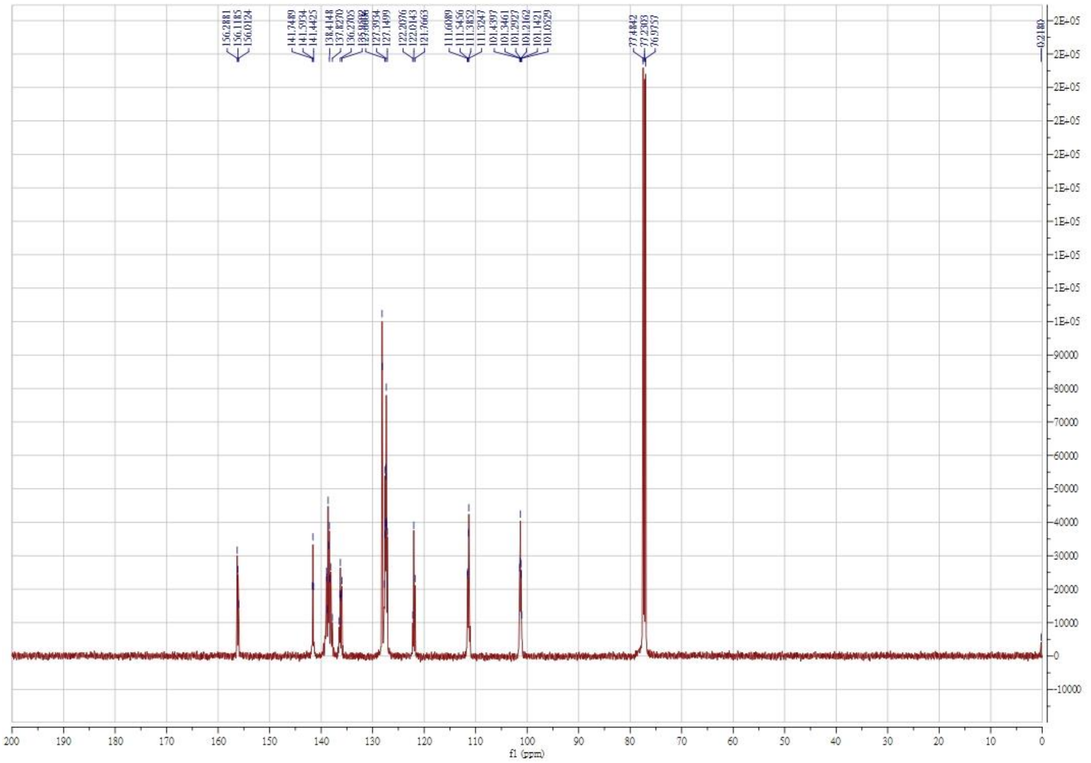

Fig. S46 ^{13}C NMR spectrum of $[Zn_4O(AID\text{-}4\text{-}Thiophen\text{-}2\text{-}yl)_6]$ (**Zn-2**) in $CDCl_3$.

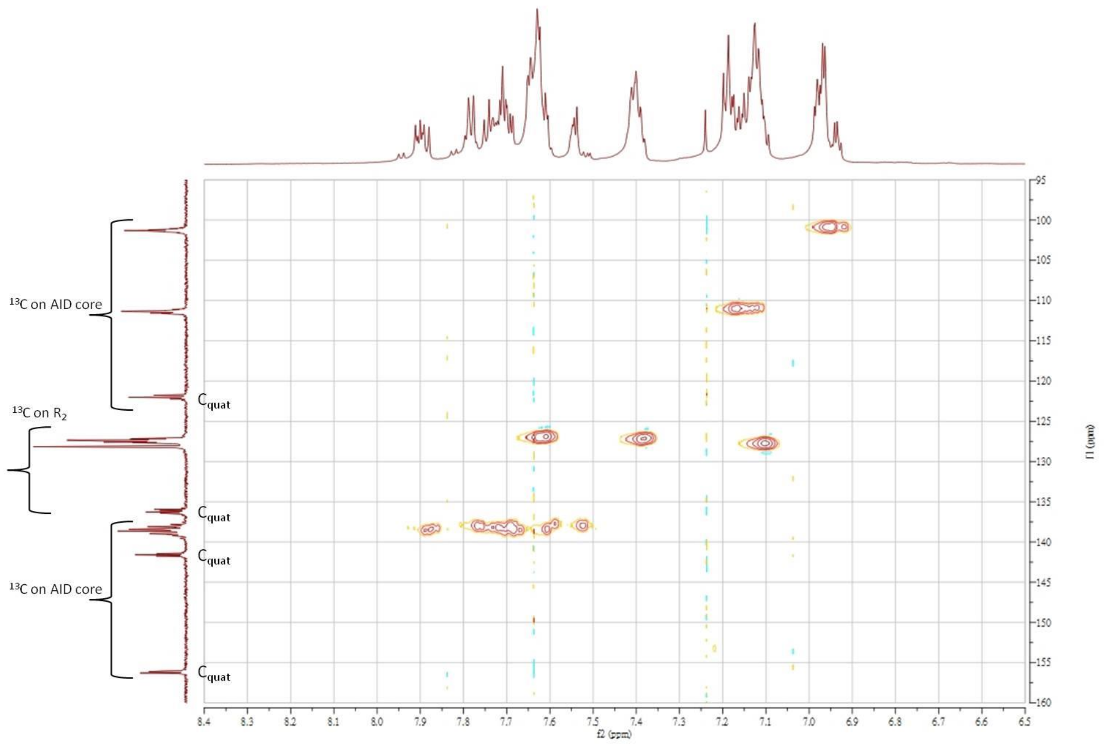

Fig. S47 ^{1}H-^{13}C HSQC NMR spectrum of [Zn_4O(AID-4-Thiophen-2-yl)$_6$] (**Zn-2**) in $CDCl_3$.

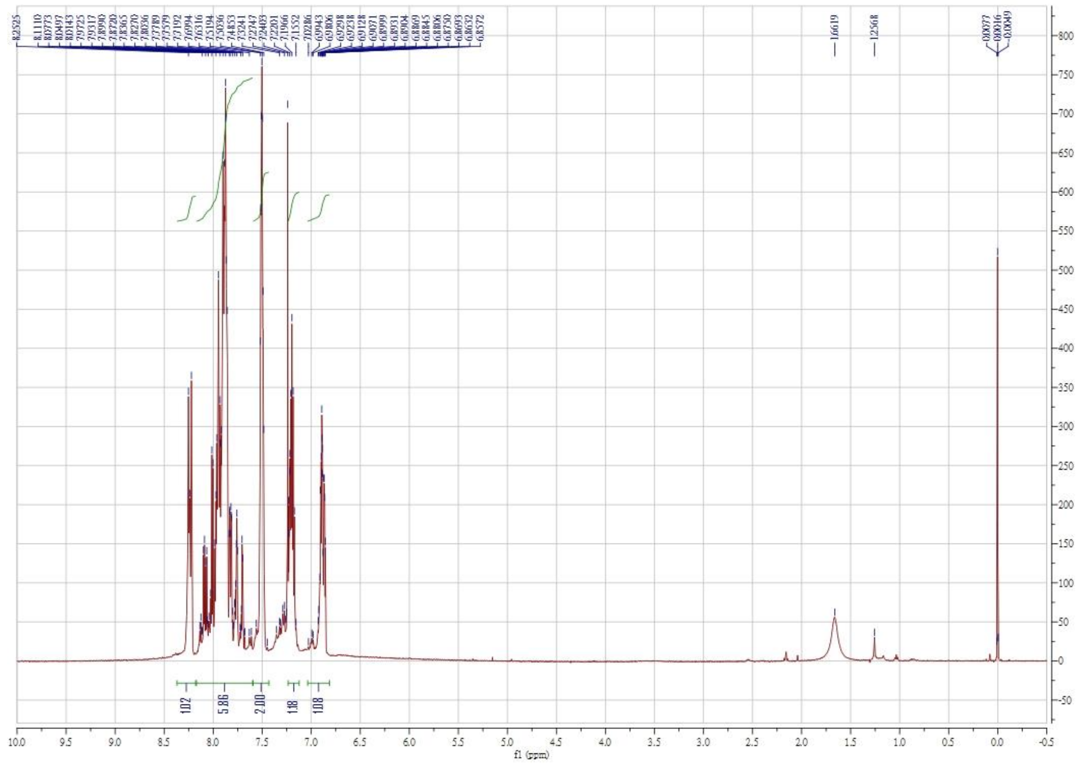

Fig. S48 ^{1}H NMR spectrum of [Zn_4O(AID-4-Naphthalen-2-yl)$_6$] (**Zn-3**) in $CDCl_3$.

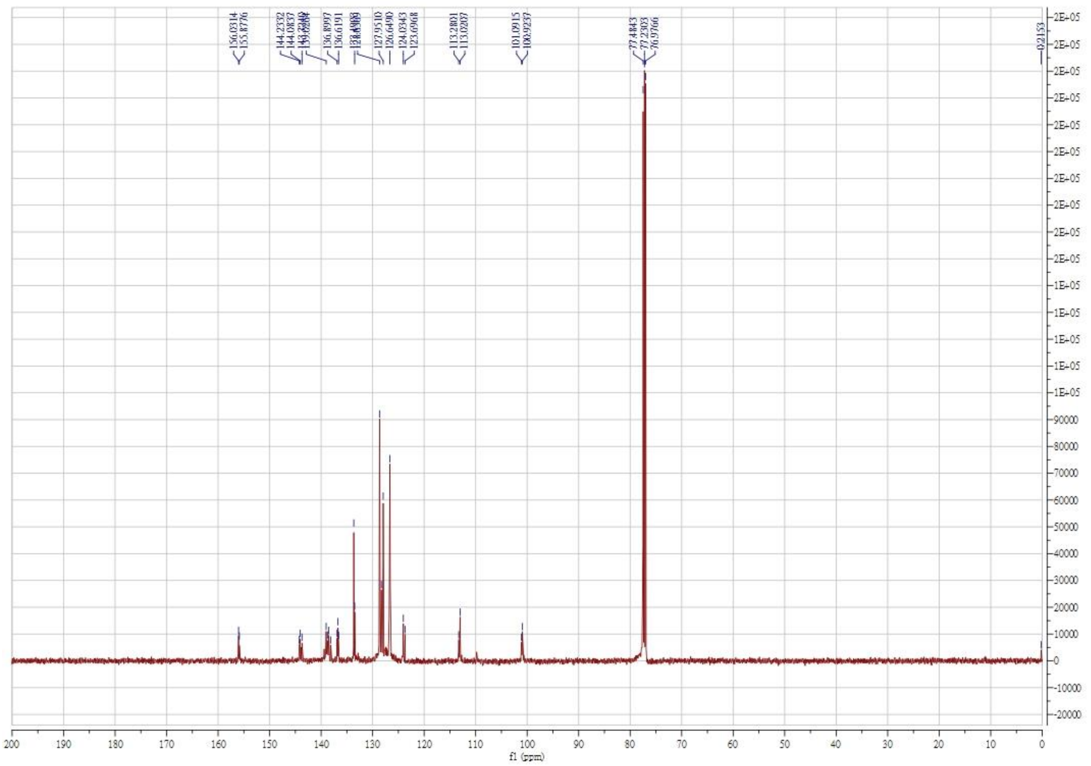

Fig. S49 ^{13}C NMR spectrum of [Zn_4O(AID-4-Naphthalen-2-yl)$_6$] (**Zn-3**) in $CDCl_3$.

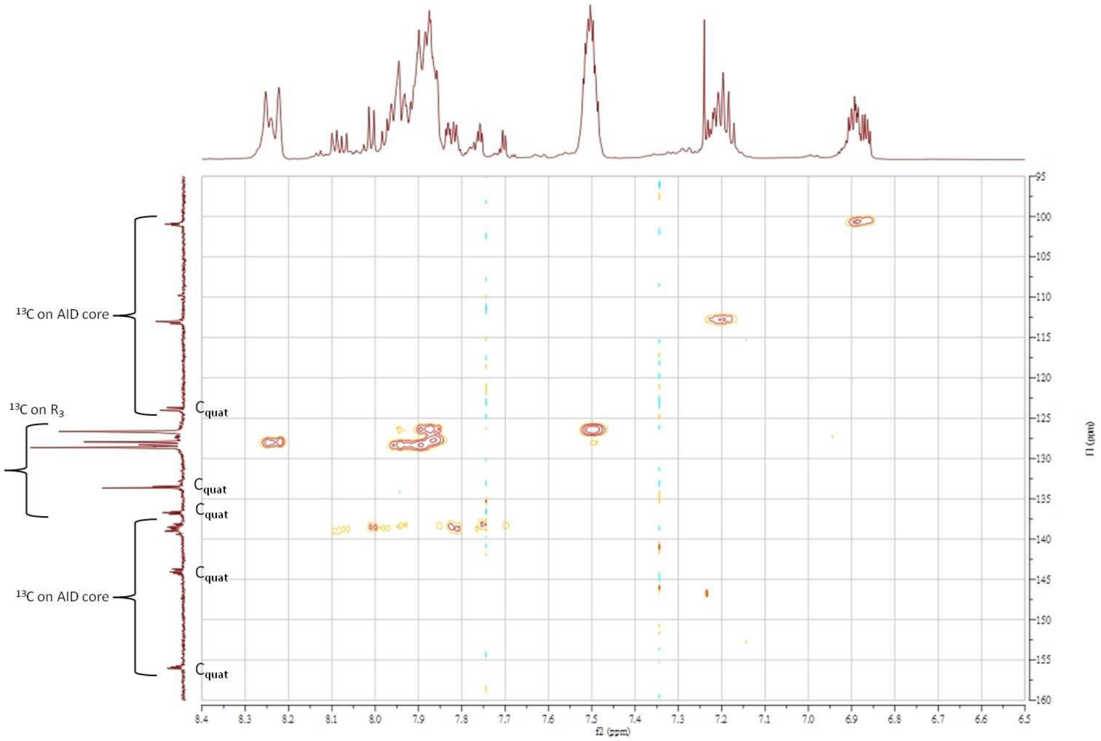

Fig. S50 ^{1}H-^{13}C HSQC NMR spectrum of [Zn_4O(AID-4-Naphthalen-2-yl)$_6$] (**Zn-3**) in $CDCl_3$.

20. Synthesis of Cu(I) complexes

Synethic procedures

1,10-Phenanthroline, 2,9-dimethylphenanthroline, 2,9-dimethyl-4,7-diphenyl-1,10-phenanthroline, 4,7-diphenylphenanthroline and 6,6'-dimethyl-2,2'-bipyridine were purchased from commercial sources and used as received. $[Me_4N][(PPh_2)_2C_2B_9H_{10}]$ was synthesized according to a literature method.[8]

Cu-1: A mixture of $[Me_4N][(PPh_2)_2C_2B_9H_{10}]$ (57.6 mg, 100 μmol) and $[Cu(MeCN)_4]PF_6$ (37.3 mg, 100 μmol) was stirred in EtOH (6 mL) for 1 h under Ar and afforded a white suspension. An ethanolic solution (5 mL) of 1,10-phenanthroline (18.4 mg, 102 μmol) degassed by bubbling with Ar was added to the white suspension and this mixture was then refluxed for 1 h. After cooling the reaction mixture to room temperature, the precipitates were filtered and washed with hexane. The copper complex was purified by column chromatography on silica gel using dichloromethane as eluent. Upon concentration under reduced pressure, hexane was added to induce precipitation and the product was filtered, washed with diethyl ether and air-dried. Yield: 52 mg (70%). ^{1}H NMR (400 MHz, [D$_6$]DMSO): δ 9.90 (d, J = 4.6 Hz, 1H), 8.92 (d, J = 8.0 Hz, 1H), 8.60 (d, J = 7.9 Hz, 1H), 8.33 (dd, J = 8.2, 4.8 Hz, 1H), 8.24 (d, J = 8.9 Hz, 1H), 8.17 (d, J = 8.9 Hz, 1H), 7.27–7.44 (m, 21H), 6.41 (d, J = 4.7 Hz, 1H), 0.60 (br, B–H), -1.91 (B–H). ^{13}C NMR (126 MHz, [D$_6$]DMSO): δ 150.6 (d, J = 8.2 Hz), 148.4, 142.6, 142.3, 137.9, 137.3, 134.9 (t, J = 19.0 Hz), 134.6 (t, J = 15.3 Hz), 134.0 (t, J = 7.2 Hz), 131.9 (t, J = 7.2 Hz), 130.3, 129.4, 128.7, 128.6, 128.2 (t, J = 5.3 Hz), 127.9 (t, J = 4.0 Hz), 127.2, 127.0, 125.9, 124.2. ^{31}P NMR (162 MHz, [D$_6$]DMSO): δ 18.1. FAB-MS (+ve): m/z 745.0 $[M]^+$. Elemental analyses calcd. for $C_{38}H_{38}B_9CuN_2P_2\cdot 0.5C_6H_{14}$: C 62.33, H 5.75, N 3.55; found: C 61.85, H 5.63, N 3.46.

Cu-2: A mixture of $[Me_4N][(PPh_2)_2C_2B_9H_{10}]$ (45 mg, 78.1 μmol) and $[Cu(MeCN)_4]PF_6$ (29.1 mg, 78.1 μmol) was stirred in EtOH (4 mL) for 1 h under Ar and afforded a white suspension. A hot ethanolic solution (6 mL) of 2,9-dimethyl-1,10-phenanthroline (16.6 mg, 79.7 μmol) degassed by bubbling with Ar was added to the white suspension and this mixture was then refluxed for 1 h. After cooling the reaction mixture to room temperature, the precipitates were filtered and washed with hexane. The copper complex was purified by column chromatography on silica gel using dichloromethane as eluent. Upon concentration under reduced pressure, hexane was added to induce precipitation and the product was filtered, washed with diethyl ether and air-dried. Yield: 41 mg (68%). ^{1}H NMR (400 MHz, [D$_6$]DMSO): δ 8.82 (d, J = 8.3 Hz, 1H), 8.47 (d, J = 8.3 Hz, 1H), 8.16 (m, 2H), 8.07 (d, J = 8.8 Hz, 1H), 7.25–7.37 (m, 21H), 3.41 (s, 3H), 0.95 (br, B–H), 0.54 (s, 3H), -1.86 (B–H). ^{13}C NMR (126 MHz, [D$_6$]DMSO): δ 160.5, 160.2, 142.6, 142.1, 138.4, 138.2 (t, J = 15.4 Hz), 137.7, 135.4 (t, J = 10.2 Hz), 134.9 (t, J = 17.9 Hz), 131.9 (t, J = 6.6 Hz), 130.7, 128.4, 128.1, 128.1, 128.0, 127.6, 127.1, 126.2, 126.1, 126.0, 125.3, 29.7, 22.4. ^{31}P NMR (162 MHz, [D$_6$]DMSO): δ 15.3. FAB-MS (+ve): m/z 773.1 $[M]^+$. Elemental analysis calcd. for $C_{40}H_{42}B_9CuN_2P_2$: C 62.11, H 5.47, N 3.62; found: C 61.93, H 5.46, N 3.52.

Cu-3: A mixture of $[Me_4N][(PPh_2)_2C_2B_9H_{10}]$ (40 mg, 69.5 μmol) and $[Cu(MeCN)_4]PF_6$ (25.9 mg, 69.5 μmol) was stirred in EtOH (5 mL) for 1 h under Ar and afforded a white suspension. An ethanolic solution (5 mL) of 2,9-dimethyl-4,7-diphenyl-1,10-phenanthroline (25.5 mg, 70.8 μmol) degassed by bubbling with Ar was added to the white suspension and this mixture was then refluxed for 1 h. After cooling the reaction mixture to room temperature, the precipitates were filtered and washed with hexane. The copper complex was purified by column chromatography on silica gel using dichloromethane as eluent. Upon concentration under reduced pressure, hexane was added to induce precipitation and the product was filtered, washed with diethyl ether and air-dried. Yield: 51 mg (79%). ^{1}H NMR (400 MHz, $CDCl_3$): δ 7.94 (d, J = 9.3 Hz, 1H), 7.88 (m, 2H), 7.62 (m, 5H), 7.45-7.53 (m, 11H), 7.37-7.40 (m, 2H), 7.31 (t, J = 7.2 Hz, 2H), 7.17-7.23 (m, 10H), 7.07 (s, 1H), 3.48 (s, 3H), 0.71 (s, 3H), -1.74 (br, B–H). ^{13}C NMR (126 MHz, $CDCl_3$): δ 160.7, 159.7, 150.2, 149.2, 144.6, 144.0, 139.5 (t, J = 15.8 Hz), 136.7, 136.7, 136.2 (t, J = 10.0 Hz), 135.6 (t, J = 18.4 Hz), 132.6 (t, J = 6.7 Hz), 130.5, 129.6, 129.4, 129.1, 129.0, 128.9, 128.2, 127.9, 127.9, 127.8, 127.8, 126.0, 125.9, 125.4, 125.3, 123.9, 123.5, 30.8, 23.3. ^{31}P NMR (162 MHz, $CDCl_3$): δ 16.2. FAB-MS (+ve): 924.2 $[M – H]^+$. Elemental analysis calcd. for $C_{52}H_{50}B_9CuN_2P_2\cdot H_2O$: C 66.18, H 5.55, N 2.97; found: C 66.50, H 5.62, N 2.84.

Cu-4: A mixture of $[Me_4N][(PPh_2)_2C_2B_9H_{10}]$ (46 mg, 80 μmol) and $[Cu(MeCN)_4]PF_6$ (29.8 mg, 80 μmol) was stirred in EtOH (5 mL) for 1 h under Ar and afforded a white suspension. An ethanolic solution (5 mL) of 4,7-diphenyl-1,10-phenanthroline (27.1 mg, 81.6 μmol) degassed by bubbling with Ar was added to the white suspension and this mixture was then refluxed for 1 h. After cooling the reaction mixture to room temperature, the precipitates were filtered and washed with hexane. The copper complex was purified by column chromatography on silica gel using dichloromethane as eluent. Upon concentration under reduced pressure, hexane was added to

induce precipitation and the product was filtered, washed with diethyl ether and air-dried. Yield: 49 mg (68%). ^{1}H NMR (400 MHz, [D$_6$]DMSO): δ 9.95 (d, *J* = 5.0 Hz, 1H), 8.29 (d, *J* = 5.0 Hz, 1H), 8.07 (d, *J* = 9.5 Hz, 1H), 8.02 (d, *J* = 9.5 Hz, 1H), 7.63–7.77 (m, 5H), 7.56 (m, 5H), 7.46 (m, 4H), 7.28–7.40 (m, 17H), 6.53 (d, *J* = 4.9 Hz, 1H), 0.63 (br, B–H), -1.91 (B–H). ^{13}C NMR (126 MHz, [D$_6$]DMSO): δ 150.2 (d, *J* = 7.5 Hz), 149.2, 148.6, 148.2, 143.4, 143.1, 136.0, 135.7, 134.9 (t, *J* = 18.9 Hz), 134.5 (t, *J* = 15.2 Hz), 134.1 (t, *J* = 9.3 Hz), 132.0 (t, *J* = 7.4 Hz), 130.3, 129.8, 129.7, 129.4, 129.3, 129.0, 128.9, 128.7, 128.2 (t, *J* = 5.3 Hz), 128.0 (t, *J* = 3.6 Hz), 126.9, 126.0, 124.8, 124.7, 124.3. ^{31}P NMR (162 MHz, [D$_6$]DMSO): δ 18.1. FAB-MS (+ve): *m*/*z* 896.5 [M – H]$^+$. Elemental analyses calcd. for $C_{50}H_{46}B_9CuN_2P_2{\cdot}0.5H_2O$: C 66.23, H 5.22, N 3.09; found: C 66.31, H 5.12, N 3.00.

Cu-5: A mixture of $[Me_4N][(PPh_2)_2C_2B_9H_{10}]$ (49 mg, 85 μmol) and $[Cu(MeCN)_4]PF_6$ (31.6 mg, 85 μmol) was stirred in EtOH (5 mL) for 1 h under Ar and afforded a white suspension. An ethanolic solution (5 mL) of 6,6'-dimethyl-2,2'-bipyridine (15.7 mg, 85 μmol) degassed by bubbling with Ar was added to the white suspension and this mixture was then refluxed for 1 h. After cooling the reaction mixture to room temperature, the precipitates were filtered and washed with hexane. The copper complex was purified by column chromatography on silica gel using dichloromethane as eluent. Upon concentration under reduced pressure, hexane was added to induce precipitation and the product was filtered, washed with diethyl ether and air-dried. Yield: 52 mg (82%). ^{1}H NMR (400 MHz, $CDCl_3$): δ 7.99-8.06 (m, 2H), 7.89 (d, *J* = 7.9 Hz, 1H), 7.66 (t, *J* = 8.0 Hz, 2H), 7.28-7.46 (m, 10H), 7.15-7.21 (m, 10H), 6.81 (d, *J* = 7.4 Hz, 1H), 3.19 (s, 3H), 0.50 (s, 3H), -1.79 (br, B–H). ^{13}C NMR could not be obtained due to low solubility in $CDCl_3$ and instability in [D$_6$]DMSO. ^{31}P NMR (162 MHz, [D$_6$]DMSO): δ 14.7. FAB-MS (+ve): *m*/*z* 749.1 [M]$^+$. Elemental analysis calcd. for $C_{38}H_{42}B_9CuN_2P_2{\cdot}CH_2Cl_2$: C 56.13, H 5.31, N 3.36; found: C 56.52, H 5.30, N 3.44.

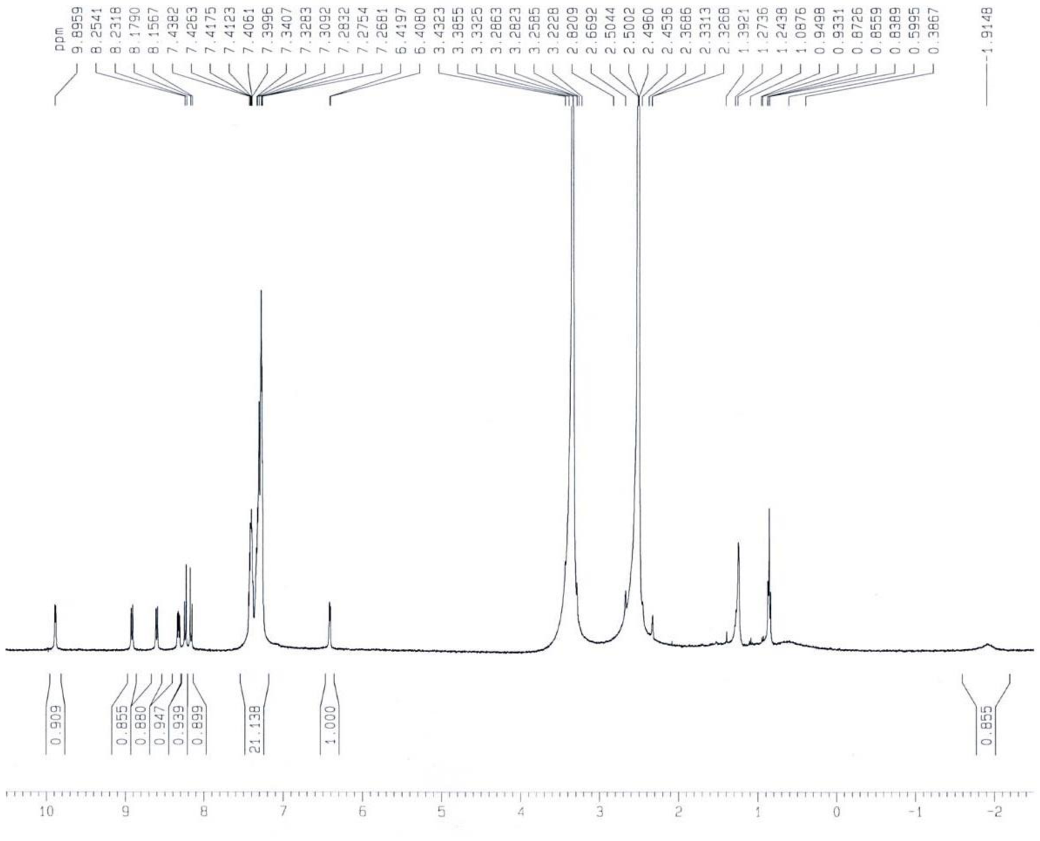

Fig. S51 ^{1}H NMR spectrum of **Cu-1** in [D$_6$]DMSO.

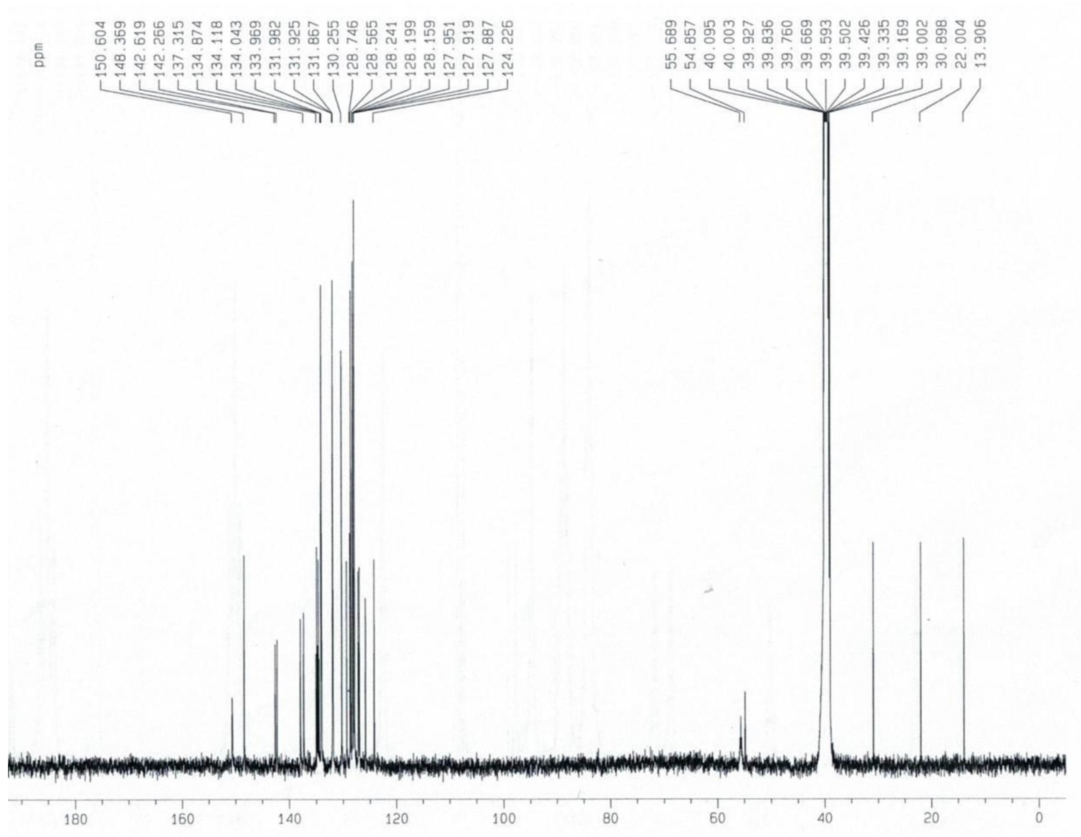

Fig. S52 ^{13}C NMR spectrum of **Cu-1** in [D_6]DMSO.

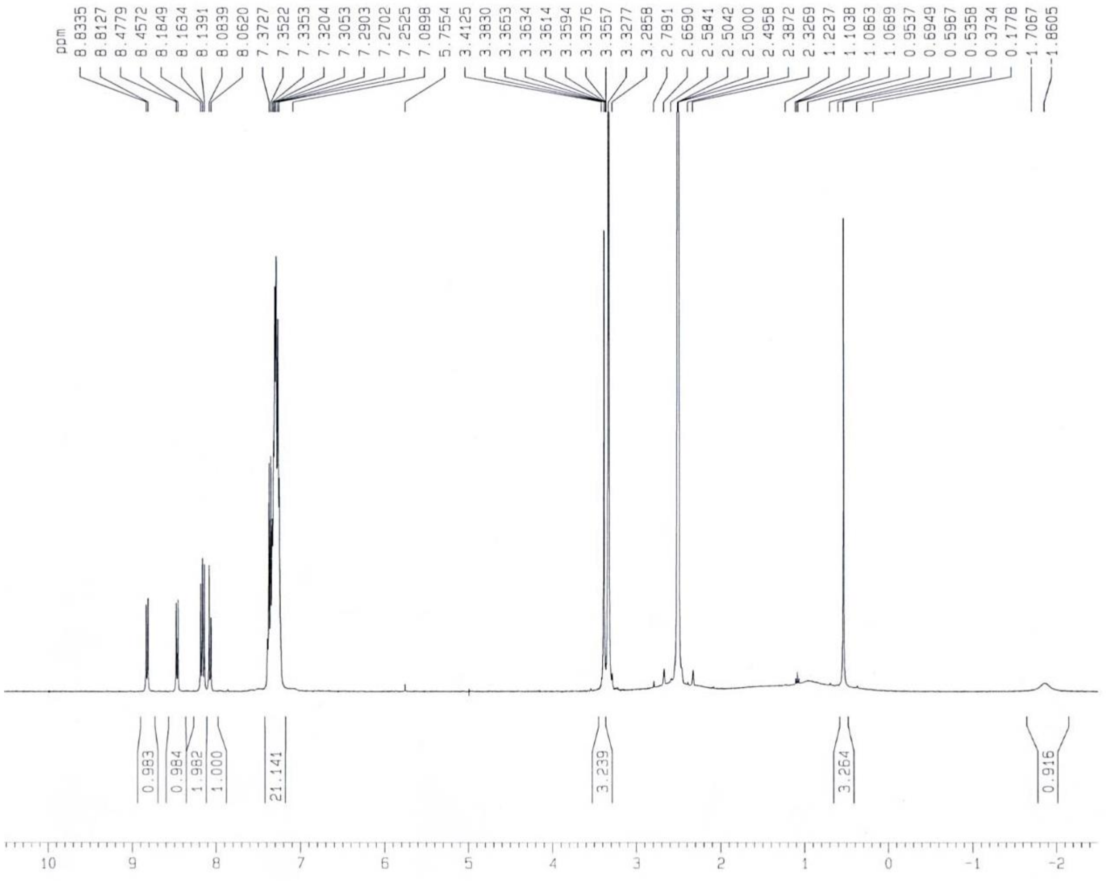

Fig. S53 ^{1}H NMR spectrum of **Cu-2** in [D_6]DMSO.

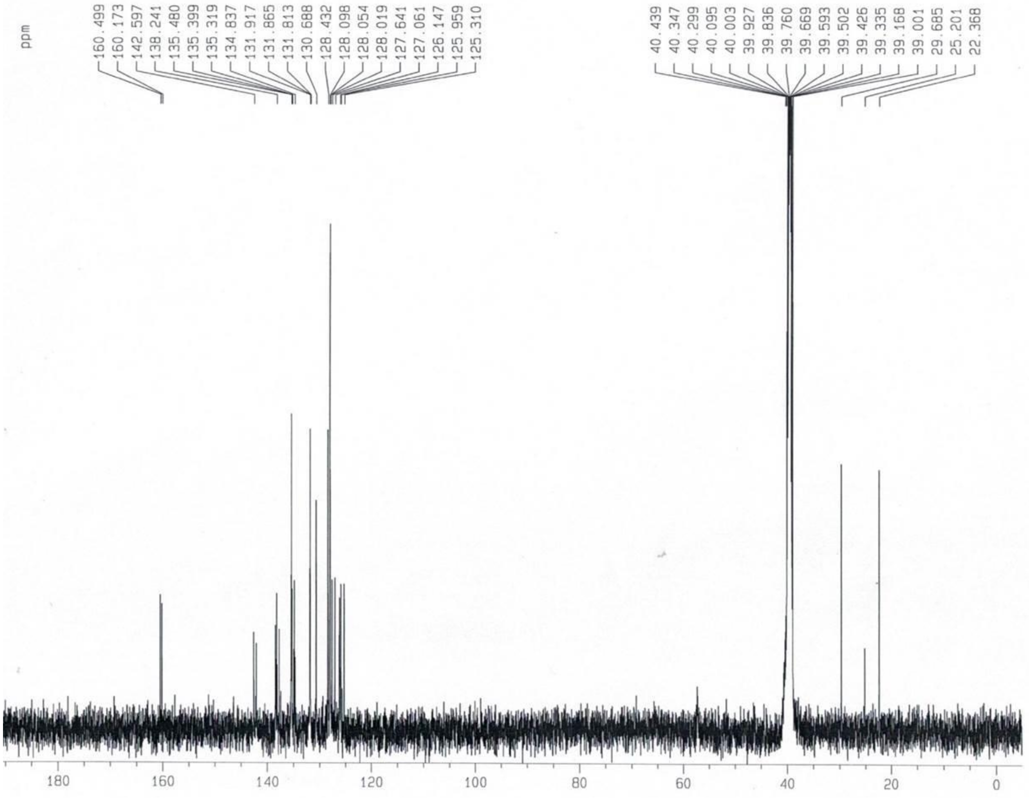

Fig. S54 ^{13}C NMR spectrum of **Cu-2** in [D$_6$]DMSO.

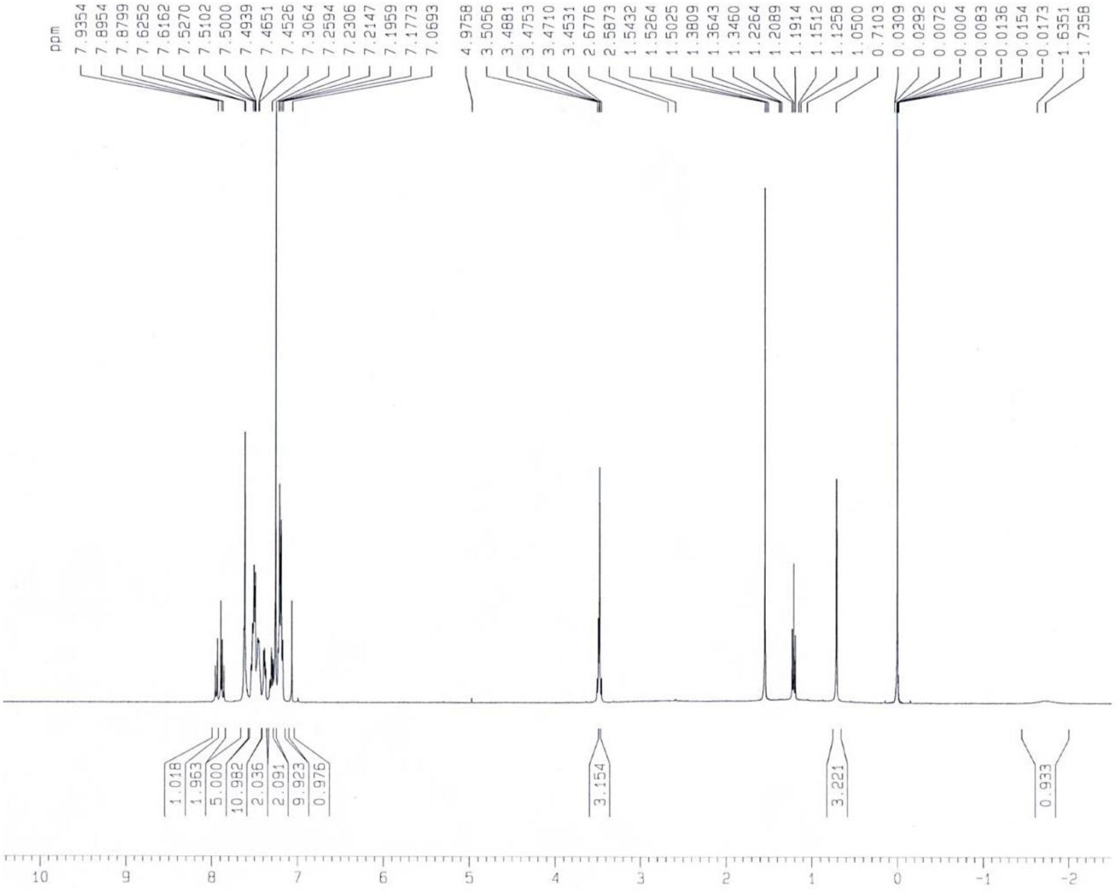

Fig. S55 ^{1}H NMR spectrum of **Cu-3** in $CDCl_3$.

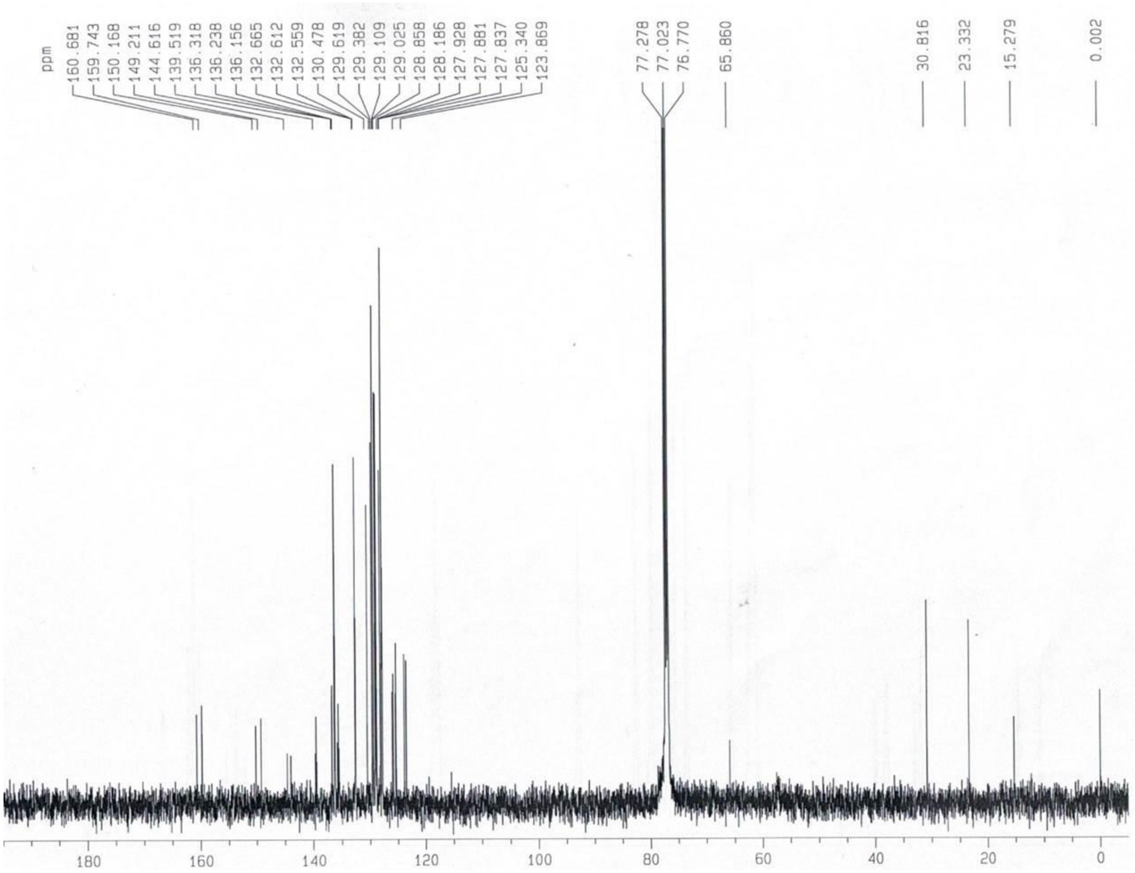

Fig. S56 ^{13}C NMR spectrum of **Cu-3** in $CDCl_3$.

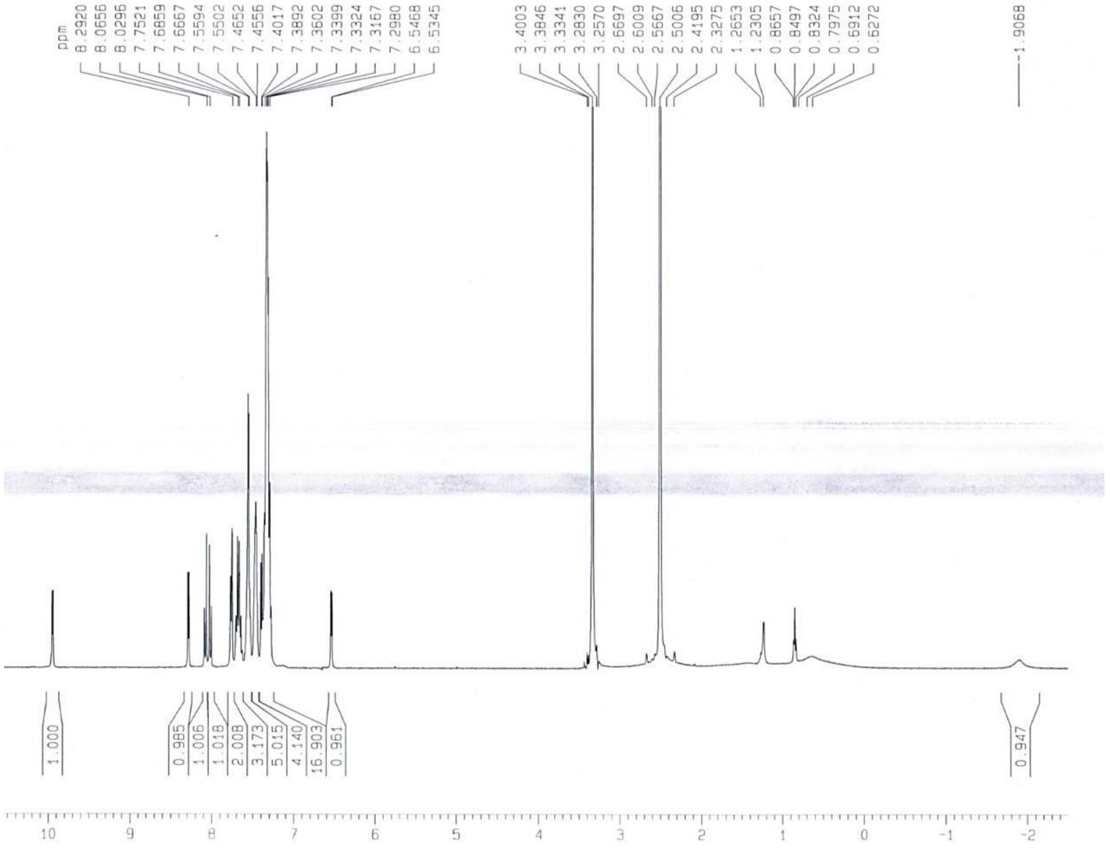

Fig. S57 ^{1}H NMR spectrum of **Cu-4** in $[D_6]DMSO$.

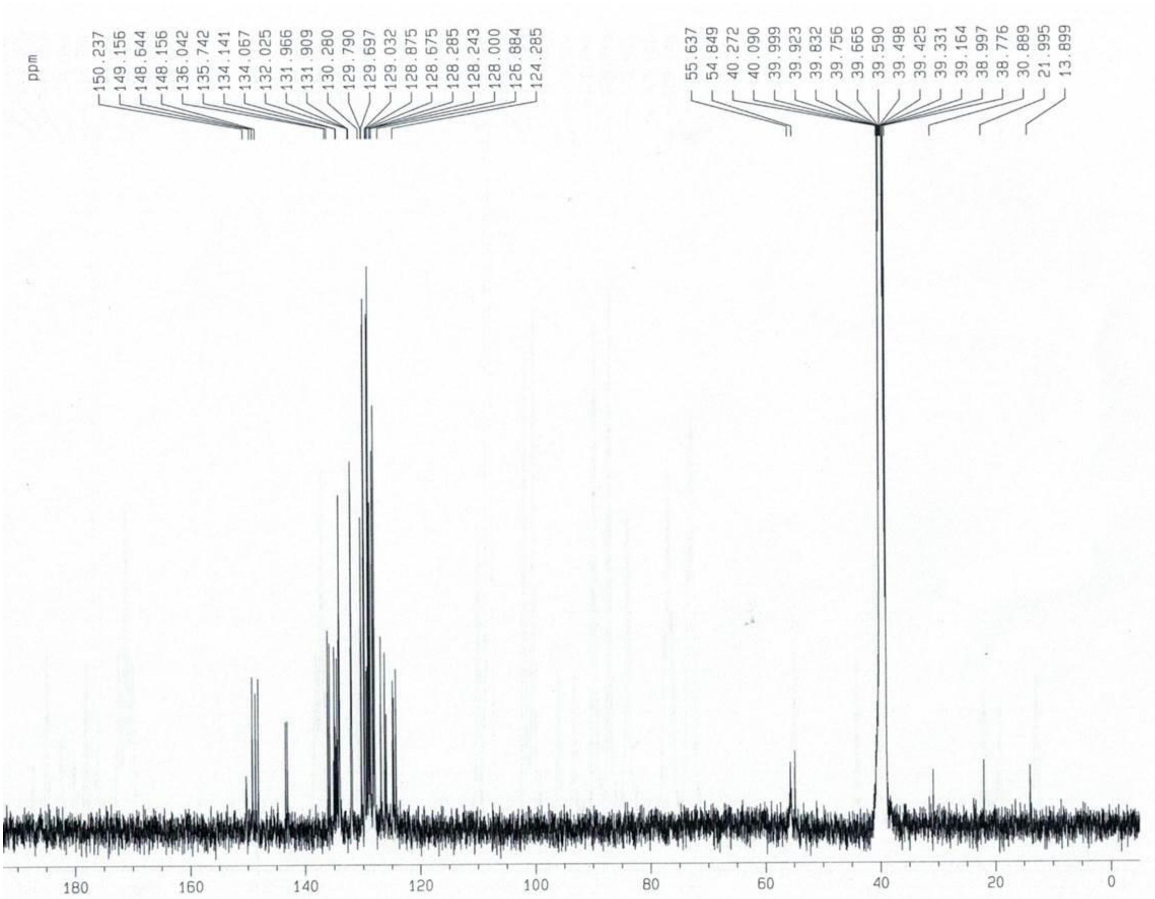

Fig. S58 ^{13}C NMR spectrum of **Cu-4** in [D$_6$]DMSO.

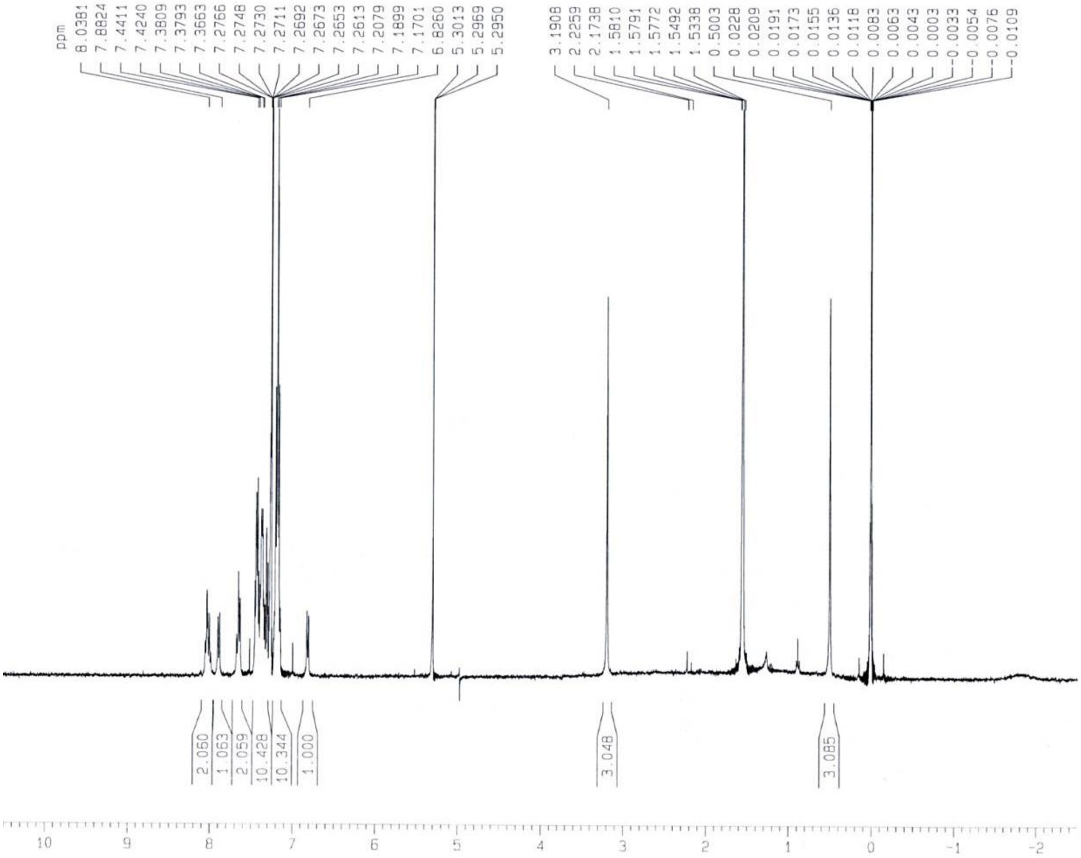

Fig. S59 ^{1}H NMR spectrum of **Cu-5** in $CDCl_3$.

21. X-ray crystal data of $Zn_4O(AID)_6$, Cu-1 and Cu-3

Table S3 Crystal data of $Zn_4O(AID)_6$, **Cu-1** and **Cu-3**

Complex	$Zn_4O(AID)_6$	**Cu-1**	**Cu-3**
Empirical formula	$C_{46}H_{40}N_{12}O_2Zn_4$	$C_{42}H_{48}B_9CuN_2OP_2$	$C_{53}H_{52}B_9Cl_2CuN_2P_2$
Formula weight	1054.46	819.59	1010.63
Temperature/K	293(2)	100	100
Crystal system	triclinic	triclinic	monoclinic
Space group	P-1	P-1	$P2_1/c$
a/Å	10.283(2)	10.828(5)	15.5324(6)
b/Å	10.474(2)	13.677(5)	15.5957(6)
c/Å	20.857(4)	14.950(7)	20.6244(9)
α/°	95.09(3)	90.64(2)	90
β/°	94.25(3)	106.555(18)	98.7890(18)
γ/°	96.25(3)	95.534(17)	90
Volume/Å ^{3}	2216.3(8)	2110.5(16)	4937.4(3)
Z	2	2	4
ρcalcg/cm^3	1.58	1.29	1.36
μ/mm^{-1}	2.888	1.712	2.53
F(000)	1072	852	2088
Crystal size/mm^3	0.08 × 0.06 × 0.04	0.3 × 0.3 × 0.3	0.2 × 0.2 × 0.04
Radiation	CuKα (λ = 1.54178)	CuKα (λ = 1.54178)	CuKα (λ = 1.54178)
2θ range for data collection/°	4.26 to 134.34	6.172 to 133.976	5.758 to 135.5
Index ranges	-12 ≤ h ≤ 11, -12 ≤ k ≤ 12, -24 ≤ l ≤ 24	-12 ≤ h ≤ 12, -16 ≤ k ≤ 15, -17 ≤ l ≤ 17	-18 ≤ h ≤ 18, -18 ≤ k ≤ 18, -24 ≤ l ≤ 24
Reflections collected	24479	38050	67504
Independent reflections	6818 [R_{int} = 0.0642]	7368 [R_{int} = 0.0495]	8693 [R_{int} = 0.0596]
Data/restraints/parameters	6818/24/512	7368/3/532	8693/0/640
Goodness-of-fit on F^2	1.121	1.058	1.103
Final *R* indexes [I>=2σ (I)]	R_1 = 0.1133, wR_2 = 0.2773	R_1 = 0.0333, wR_2 = 0.0920	R_1 = 0.0603, wR_2 = 0.1666
Final *R* indexes [all data]	R_1 = 0.1253, wR_2 = 0.2831	R_1 = 0.0336, wR_2 = 0.0923	R_1 = 0.0670, wR_2 = 0.1720
Largest diff. peak/hole / e Å $^{-3}$	1.33/-1.63	1.08/-0.38	0.95/-1.15

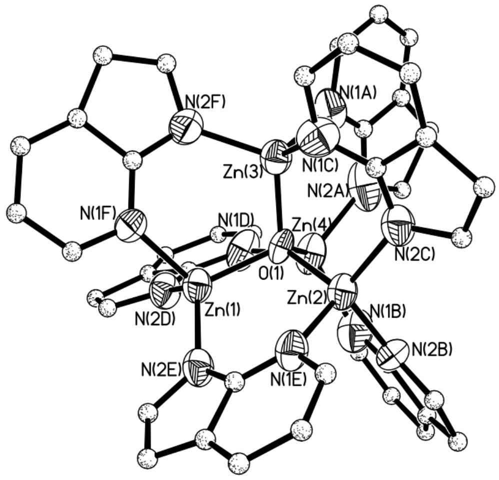

Fig. S60 Perspective view of $Zn_4O(AID)_6$ (all hydrogen atoms are omitted for clarity).

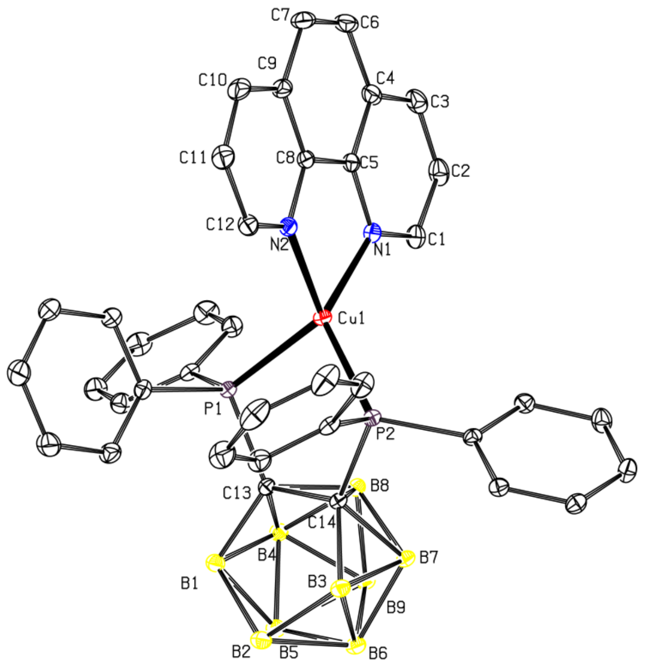

Fig. S61 Perspective view of **Cu-1** (all hydrogen atoms are omitted for clarity).

22. Computational details

Density functional theory (DFT) and time-dependent density functional theroy (TDDFT) calculations have been performed to understand the geometries and the electronic structures of copper(I) complexes (**Cu-1 to Cu-5)**, and Zn(II) complexes **(Zn-1 to Zn-3)** using Gaussian 09 package.[4] PBE0[5]/6-31G*(lanl2dz)[6] was used for the geometry optimization. PBE0 with triplet-zeta basis set (6-311G*) was used for TDDFT calculations of copper(I) complexes. Due to the very large size of the Zn(II) complexes, only double-zeta with pseudo potential basis set (6-31G*(lanl2dz) was employed for TDDFT calculations. The Solvent effects have been studied using self-consistent reaction field (SCRF) method based on PCM models.[7] The choice of solvents (dichloromethne，a dielectric constant $\varepsilon = 8.93$) was based on the solvent media for experiments.

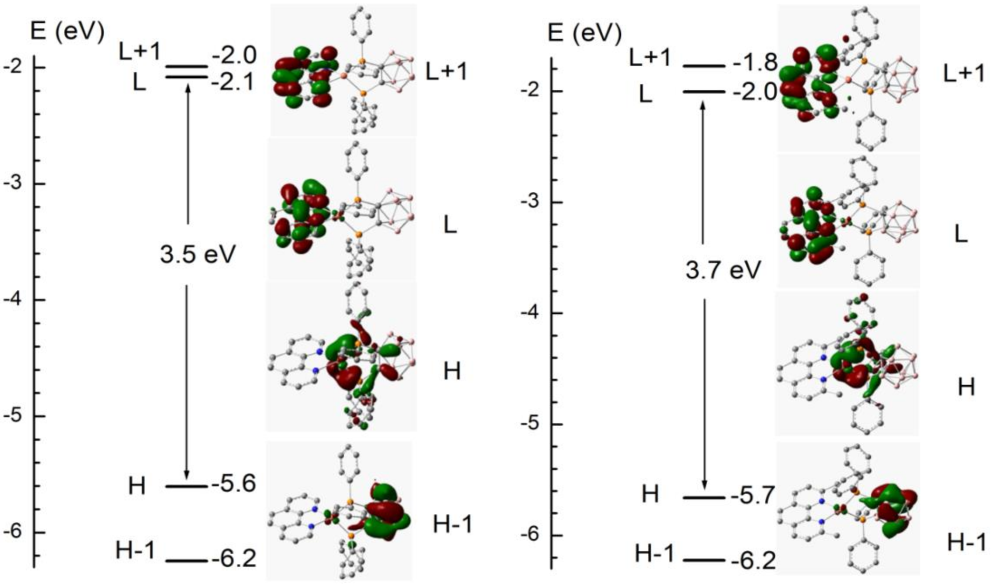

Fig. S62 Comparison of FMO diagram of **Cu-1** and **Cu-2** at ground state (**S$_0$**).

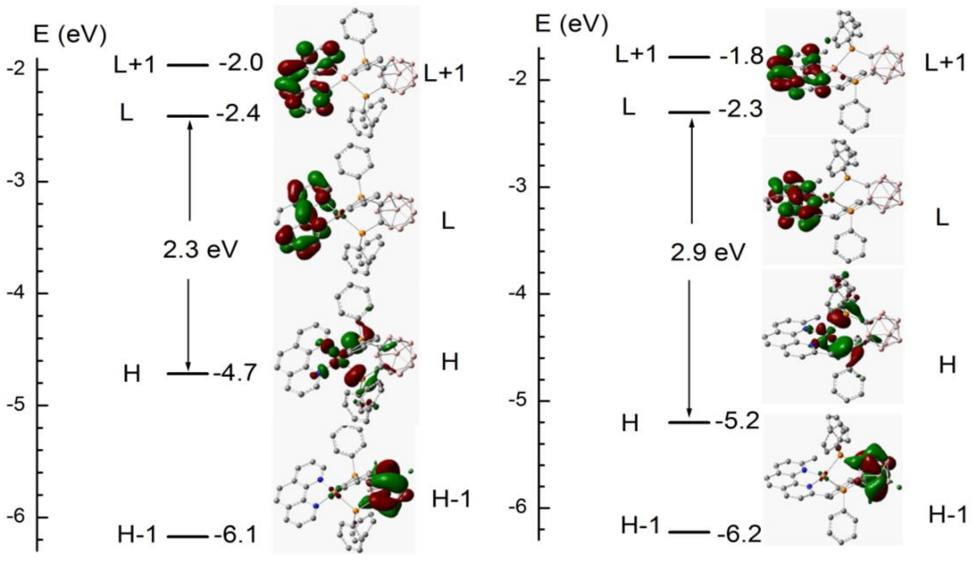

Cu-1 **Cu-2**

Fig. 63 Comparison of MO diagram of **Cu-1** and **Cu-2** at singlet excited state (**S$_1$**).

Geometries of the stationary points in mol2 format

Cu-1 S_0
@<TRIPOS>MOLECULE
Molecule Name
90 110
SMALL
NO_CHARGES

@<TRIPOS>ATOM
1 Cu1 -0.8105 0.2355 -0.1878 Cu
2 P2 0.4332 -1.7160 -0.1330 P
3 P3 1.1436 1.4510 -0.0391 P
4 N4 -2.3908 0.3586 1.2274 N
5 N5 -2.4432 0.6215 -1.4570 N
6 C6 -3.5848 0.6038 0.6445 C
7 C7 -2.3446 0.1910 2.5406 C
8 C8 -3.4853 0.2699 3.3554 C
9 H9 -3.3897 0.1247 4.4265 H
10 C10 -4.7071 0.5336 2.7746 C
11 H11 -5.6092 0.6055 3.3770 H
12 C12 -4.7871 0.7086 1.3790 C
13 C13 -6.0117 0.9807 0.6884 C
14 H14 -6.9269 1.0629 1.2687 H
15 C15 -6.0354 1.1328 -0.6627 C
16 H16 -6.9695 1.3387 -1.1787 H
17 C17 -4.8375 1.0196 -1.4390 C
18 C18 -4.8105 1.1526 -2.8408 C
19 H19 -5.7326 1.3587 -3.3784 H
20 C20 -3.6138 1.0154 -3.5116 C
21 H21 -3.5573 1.1082 -4.5912 H
22 C22 -2.4494 0.7465 -2.7769 C
23 C23 -3.6112 0.7542 -0.7880 C
24 C24 -0.0702 -3.2193 -1.0399 C
25 C25 0.5198 -4.4678 -0.8063 C
26 H26 1.3101 -4.5610 -0.0671 H
27 C27 0.1067 -5.5812 -1.5285 C
28 H28 0.5743 -6.5451 -1.3467 H
29 C29 -0.8957 -5.4610 -2.4905 C
30 H30 -1.2133 -6.3334 -3.0555 H
31 C31 -1.4888 -4.2244 -2.7261 C
32 H32 -2.2719 -4.1278 -3.4736 H
33 C33 -1.0803 -3.1086 -1.9992 C
34 H34 -1.5451 -2.1410 -2.1721 H
35 C35 0.6379 -2.3212 1.5814 C
36 C36 -0.3812 -3.1055 2.1425 C
37 H37 -1.2145 -3.4315 1.5244 H
38 C38 -0.3267 -3.4870 3.4788 C
39 H39 -1.1172 -4.1075 3.8936 H
40 C40 0.7392 -3.0812 4.2809 C
41 H41 0.7823 -3.3799 5.3249 H
42 C42 1.7496 -2.2960 3.7344 C
43 H43 2.5866 -1.9753 4.3487 H
44 C44 1.7012 -1.9201 2.3939 C
45 H45 2.5003 -1.3158 1.9799 H
46 C46 1.5577 1.7796 1.7189 C
47 C47 2.7561 1.4256 2.3453 C
48 H48 3.5426 0.9445 1.7725 H
49 C49 2.9536 1.7072 3.6966 C

50 H50 3.8925 1.4285 4.1680 H
51 C51 1.9656 2.3505 4.4355 C
52 H52 2.1263 2.5722 5.4873 H
53 C53 0.7693 2.7126 3.8182 C
54 H54 -0.0067 3.2206 4.3855 H
55 C55 0.5649 2.4216 2.4738 C
56 H56 -0.3741 2.6989 1.9980 H
57 C57 1.3520 3.0900 -0.8186 C
58 C58 0.4886 3.4426 -1.8604 C
59 H59 -0.2810 2.7420 -2.1745 H
60 C60 0.6109 4.6769 -2.4915 C
61 H61 -0.0605 4.9387 -3.3051 H
62 C62 1.5898 5.5754 -2.0754 C
63 H63 1.6855 6.5411 -2.5648 H
64 C64 2.4440 5.2367 -1.0281 C
65 H65 3.2086 5.9362 -0.7011 H
66 C66 2.3278 4.0009 -0.4006 C
67 H67 2.9997 3.7396 0.4111 H
68 C68 2.4379 0.3448 -0.7469 C
69 C69 2.0880 -1.2080 -0.7577 C
70 B70 4.0186 0.6993 -0.6415 B
71 H71 4.3858 1.7162 -0.1264 H
72 B72 3.2324 0.8357 -2.2134 B
73 H73 2.9922 1.8844 -2.7239 H
74 B74 1.9423 -0.3764 -2.2401 B
75 H75 0.8529 -0.1774 -2.6945 H
76 B76 2.6994 -1.9676 -2.1725 B
77 H77 2.1218 -2.9098 -2.6110 H
78 B78 3.4605 -2.0897 -0.5503 B
79 H79 3.4370 -3.1534 -0.0036 H
80 B80 4.4365 -1.7757 -2.0122 B
81 H81 5.1759 -2.5827 -2.4894 H
82 B82 4.8689 -0.8933 -0.5307 B
83 H83 5.9294 -1.1202 -0.0277 H
84 H84 3.9749 -1.1590 0.3380 H
85 B85 3.4642 -0.6924 -3.0756 B
86 H86 3.5128 -0.7352 -4.2669 H
87 B87 4.7581 -0.0009 -2.0568 B
88 H88 5.7823 0.4182 -2.5063 H
89 H89 -1.4898 0.6245 -3.2738 H
90 H90 -1.3653 -0.0111 2.9657 H
@<TRIPOS>BOND
1 1 2 1
2 1 3 1
3 2 24 1
4 2 35 1
5 2 69 1
6 3 46 1
7 3 57 1
8 3 68 1
9 4 6 Ar
10 4 7 Ar
11 5 22 Ar
12 5 23 Ar
13 6 12 Ar
14 6 23 Ar
15 7 8 Ar
16 7 90 1
17 8 9 1
18 8 10 2

19 10 11 1
20 10 12 Ar
21 12 13 Ar
22 13 14 1
23 13 15 2
24 15 16 1
25 15 17 Ar
26 17 18 Ar
27 17 23 Ar
28 18 19 1
29 18 20 2
30 20 21 1
31 20 22 Ar
32 22 89 1
33 24 25 Ar
34 24 33 Ar
35 25 26 1
36 25 27 Ar
37 27 28 1
38 27 29 Ar
39 29 30 1
40 29 31 Ar
41 31 32 1
42 31 33 Ar
43 33 34 1
44 35 36 Ar
45 35 44 Ar
46 36 37 1
47 36 38 Ar
48 38 39 1
49 38 40 Ar
50 40 41 1
51 40 42 Ar
52 42 43 1
53 42 44 Ar
54 44 45 1
55 46 47 Ar
56 46 55 Ar
57 47 48 1
58 47 49 Ar
59 49 50 1
60 49 51 Ar
61 51 52 1
62 51 53 Ar
63 53 54 1
64 53 55 Ar
65 55 56 1
66 57 58 Ar
67 57 66 Ar
68 58 59 1
69 58 60 Ar
70 60 61 1
71 60 62 Ar
72 62 63 1
73 62 64 Ar
74 64 65 1
75 64 66 Ar
76 66 67 1
77 68 69 1
78 68 70 1

79 68 72 1
80 68 74 1
81 69 74 1
82 69 76 1
83 69 78 1
84 70 71 1
85 70 72 1
86 70 82 1
87 70 87 1
88 72 73 1
89 72 74 1
90 72 85 1
91 72 87 1
92 74 75 1
93 74 76 1
94 74 85 1
95 76 77 1
96 76 78 1
97 76 80 1
98 76 85 1
99 78 79 1
100 78 80 1
101 78 82 1
102 80 81 1
103 80 82 1
104 80 85 1
105 80 87 1
106 82 83 1
107 82 87 1
108 85 86 1
109 85 87 1
110 87 88 1

Cu-1 S_1
@<TRIPOS>MOLECULE
Molecule Name
90 110
SMALL
NO_CHARGES

@<TRIPOS>ATOM
1 Cu1 -0.8105 0.2355 -0.1878 Cu
2 P2 0.4332 -1.7160 -0.1330 P
3 P3 1.1436 1.4510 -0.0391 P
4 N4 -2.3908 0.3586 1.2274 N
5 N5 -2.4432 0.6215 -1.4570 N
6 C6 -3.5848 0.6038 0.6445 C
7 C7 -2.3446 0.1910 2.5406 C
8 C8 -3.4853 0.2699 3.3554 C
9 H9 -3.3897 0.1247 4.4265 H
10 C10 -4.7071 0.5336 2.7746 C
11 H11 -5.6092 0.6055 3.3770 H
12 C12 -4.7871 0.7086 1.3790 C
13 C13 -6.0117 0.9807 0.6884 C
14 H14 -6.9269 1.0629 1.2687 H
15 C15 -6.0354 1.1328 -0.6627 C
16 H16 -6.9695 1.3387 -1.1787 H
17 C17 -4.8375 1.0196 -1.4390 C
18 C18 -4.8105 1.1526 -2.8408 C

19	H19	-5.7326	1.3587	-3.3784	H
20	C20	-3.6138	1.0154	-3.5116	C
21	H21	-3.5573	1.1082	-4.5912	H
22	C22	-2.4494	0.7465	-2.7769	C
23	C23	-3.6112	0.7542	-0.7880	C
24	C24	-0.0702	-3.2193	-1.0399	C
25	C25	0.5198	-4.4678	-0.8063	C
26	H26	1.3101	-4.5610	-0.0671	H
27	C27	0.1067	-5.5812	-1.5285	C
28	H28	0.5743	-6.5451	-1.3467	H
29	C29	-0.8957	-5.4610	-2.4905	C
30	H30	-1.2133	-6.3334	-3.0555	H
31	C31	-1.4888	-4.2244	-2.7261	C
32	H32	-2.2719	-4.1278	-3.4736	H
33	C33	-1.0803	-3.1086	-1.9992	C
34	H34	-1.5451	-2.1410	-2.1721	H
35	C35	0.6379	-2.3212	1.5814	C
36	C36	-0.3812	-3.1055	2.1425	C
37	H37	-1.2145	-3.4315	1.5244	H
38	C38	-0.3267	-3.4870	3.4788	C
39	H39	-1.1172	-4.1075	3.8936	H
40	C40	0.7392	-3.0812	4.2809	C
41	H41	0.7823	-3.3799	5.3249	H
42	C42	1.7496	-2.2960	3.7344	C
43	H43	2.5866	-1.9753	4.3487	H
44	C44	1.7012	-1.9201	2.3939	C
45	H45	2.5003	-1.3158	1.9799	H
46	C46	1.5577	1.7796	1.7189	C
47	C47	2.7561	1.4256	2.3453	C
48	H48	3.5426	0.9445	1.7725	H
49	C49	2.9536	1.7072	3.6966	C
50	H50	3.8925	1.4285	4.1680	H
51	C51	1.9656	2.3505	4.4355	C
52	H52	2.1263	2.5722	5.4873	H
53	C53	0.7693	2.7126	3.8182	C
54	H54	-0.0067	3.2206	4.3855	H
55	C55	0.5649	2.4216	2.4738	C
56	H56	-0.3741	2.6989	1.9980	H
57	C57	1.3520	3.0900	-0.8186	C
58	C58	0.4886	3.4426	-1.8604	C
59	H59	-0.2810	2.7420	-2.1745	H
60	C60	0.6109	4.6769	-2.4915	C
61	H61	-0.0605	4.9387	-3.3051	H
62	C62	1.5898	5.5754	-2.0754	C
63	H63	1.6855	6.5411	-2.5648	H
64	C64	2.4440	5.2367	-1.0281	C
65	H65	3.2086	5.9362	-0.7011	H
66	C66	2.3278	4.0009	-0.4006	C
67	H67	2.9997	3.7396	0.4111	H
68	C68	2.4379	0.3448	-0.7469	C
69	C69	2.0880	-1.2080	-0.7577	C
70	B70	4.0186	0.6993	-0.6415	B
71	H71	4.3858	1.7162	-0.1264	H
72	B72	3.2324	0.8357	-2.2134	B
73	H73	2.9922	1.8844	-2.7239	H
74	B74	1.9423	-0.3764	-2.2401	B
75	H75	0.8529	-0.1774	-2.6945	H
76	B76	2.6994	-1.9676	-2.1725	B
77	H77	2.1218	-2.9098	-2.6110	H
78	B78	3.4605	-2.0897	-0.5503	B

79 H79 3.4370 -3.1534 -0.0036 H
80 B80 4.4365 -1.7757 -2.0122 B
81 H81 5.1759 -2.5827 -2.4894 H
82 B82 4.8689 -0.8933 -0.5307 B
83 H83 5.9294 -1.1202 -0.0277 H
84 H84 3.9749 -1.1590 0.3380 H
85 B85 3.4642 -0.6924 -3.0756 B
86 H86 3.5128 -0.7352 -4.2669 H
87 B87 4.7581 -0.0009 -2.0568 B
88 H88 5.7823 0.4182 -2.5063 H
89 H89 -1.4898 0.6245 -3.2738 H
90 H90 -1.3653 -0.0111 2.9657 H
@<TRIPOS>BOND
1 1 2 1
2 1 3 1
3 2 24 1
4 2 35 1
5 2 69 1
6 3 46 1
7 3 57 1
8 3 68 1
9 4 6 Ar
10 4 7 Ar
11 5 22 Ar
12 5 23 Ar
13 6 12 Ar
14 6 23 Ar
15 7 8 Ar
16 7 90 1
17 8 9 1
18 8 10 2
19 10 11 1
20 10 12 Ar
21 12 13 Ar
22 13 14 1
23 13 15 2
24 15 16 1
25 15 17 Ar
26 17 18 Ar
27 17 23 Ar
28 18 19 1
29 18 20 2
30 20 21 1
31 20 22 Ar
32 22 89 1
33 24 25 Ar
34 24 33 Ar
35 25 26 1
36 25 27 Ar
37 27 28 1
38 27 29 Ar
39 29 30 1
40 29 31 Ar
41 31 32 1
42 31 33 Ar
43 33 34 1
44 35 36 Ar
45 35 44 Ar
46 36 37 1
47 36 38 Ar

48 38 39 1
49 38 40 Ar
50 40 41 1
51 40 42 Ar
52 42 43 1
53 42 44 Ar
54 44 45 1
55 46 47 Ar
56 46 55 Ar
57 47 48 1
58 47 49 Ar
59 49 50 1
60 49 51 Ar
61 51 52 1
62 51 53 Ar
63 53 54 1
64 53 55 Ar
65 55 56 1
66 57 58 Ar
67 57 66 Ar
68 58 59 1
69 58 60 Ar
70 60 61 1
71 60 62 Ar
72 62 63 1
73 62 64 Ar
74 64 65 1
75 64 66 Ar
76 66 67 1
77 68 69 1
78 68 70 1
79 68 72 1
80 68 74 1
81 69 74 1
82 69 76 1
83 69 78 1
84 70 71 1
85 70 72 1
86 70 82 1
87 70 87 1
88 72 73 1
89 72 74 1
90 72 85 1
91 72 87 1
92 74 75 1
93 74 76 1
94 74 85 1
95 76 77 1
96 76 78 1
97 76 80 1
98 76 85 1
99 78 79 1
100 78 80 1
101 78 82 1
102 80 81 1
103 80 82 1
104 80 85 1
105 80 87 1
106 82 83 1
107 82 87 1

108 85 86 1
109 85 87 1
110 87 88 1

Cu-1 T_1
@<TRIPOS>MOLECULE
Molecule Name
90 108
SMALL
NO_CHARGES

@<TRIPOS>ATOM
1 Cu1 -0.8814 0.2475 0.1903 Cu
2 P2 0.5892 -1.5609 -0.3586 P
3 P3 1.1165 1.4942 0.1972 P
4 N4 -2.4019 -0.6787 1.1060 N
5 N5 -2.3505 1.4164 -0.5560 N
6 C6 -3.6118 -0.0998 0.7717 C
7 C7 -2.4113 -1.7175 1.9648 C
8 C8 -3.5662 -2.2301 2.5215 C
9 H9 -3.5030 -3.0652 3.2107 H
10 C10 -4.8093 -1.6597 2.1810 C
11 H11 -5.7335 -2.0502 2.5975 H
12 C12 -4.8439 -0.5846 1.2984 C
13 C13 -6.0518 0.0720 0.8775 C
14 H14 -6.9972 -0.2911 1.2729 H
15 C15 -6.0284 1.1143 0.0048 C
16 H16 -6.9550 1.5894 -0.3077 H
17 C17 -4.7934 1.6179 -0.5299 C
18 C18 -4.7078 2.6706 -1.4351 C
19 H19 -5.6120 3.1676 -1.7757 H
20 C20 -3.4443 3.0753 -1.9032 C
21 H21 -3.3428 3.8858 -2.6163 H
22 C22 -2.3127 2.4298 -1.4385 C
23 C23 -3.5852 0.9893 -0.1073 C
24 C24 -0.3215 -2.7368 -1.4040 C
25 C25 0.2211 -3.9816 -1.7529 C
26 H26 1.1944 -4.2793 -1.3735 H
27 C27 -0.4847 -4.8345 -2.5939 C
28 H28 -0.0598 -5.7970 -2.8633 H
29 C29 -1.7303 -4.4536 -3.0935 C
30 H30 -2.2780 -5.1234 -3.7505 H
31 C31 -2.2733 -3.2186 -2.7498 C
32 H32 -3.2445 -2.9209 -3.1342 H
33 C33 -1.5733 -2.3614 -1.9043 C
34 H34 -2.0082 -1.4038 -1.6290 H
35 C35 1.4131 -2.5614 0.9167 C
36 C36 0.7563 -3.6809 1.4493 C
37 H37 -0.2039 -3.9949 1.0498 H
38 C38 1.3412 -4.4125 2.4787 C
39 H39 0.8270 -5.2828 2.8762 H
40 C40 2.5812 -4.0352 2.9896 C
41 H41 3.0375 -4.6094 3.7908 H
42 C42 3.2350 -2.9206 2.4690 C
43 H43 4.2018 -2.6197 2.8616 H
44 C44 2.6544 -2.1840 1.4415 C
45 H45 3.1735 -1.3127 1.0569 H
46 C46 1.9159 1.1496 1.8041 C
47 C47 3.2646 1.4348 2.0539 C

48 H48 3.8910 1.8447 1.2685 H
49 C49 3.8063 1.1842 3.3108 C
50 H50 4.8543 1.4017 3.4962 H
51 C51 3.0086 0.6631 4.3295 C
52 H52 3.4362 0.4723 5.3098 H
53 C53 1.6644 0.3908 4.0912 C
54 H54 1.0382 -0.0133 4.8813 H
55 C55 1.1204 0.6301 2.8318 C
56 H56 0.0701 0.4073 2.6514 H
57 C57 1.0085 3.3138 0.1273 C
58 C58 -0.1552 3.8671 0.6796 C
59 H59 -0.9523 3.2164 1.0300 H
60 C60 -0.3001 5.2481 0.7756 C
61 H61 -1.2078 5.6647 1.2025 H
62 C62 0.7119 6.0874 0.3167 C
63 H63 0.5980 7.1656 0.3857 H
64 C64 1.8717 5.5422 -0.2306 C
65 H65 2.6647 6.1931 -0.5875 H
66 C66 2.0273 4.1620 -0.3225 C
67 H67 2.9371 3.7528 -0.7456 H
68 C68 2.1675 0.7828 -1.1285 C
69 C69 1.8856 -0.7568 -1.3830 C
70 B70 3.6924 1.2490 -1.4320 B
71 H71 4.2104 2.1446 -0.8328 H
72 B72 2.4273 1.6382 -2.6104 B
73 H73 2.0033 2.7390 -2.7775 H
74 B74 1.2309 0.3331 -2.5155 B
75 H75 0.0516 0.5141 -2.5478 H
76 B76 2.0168 -1.1502 -3.0492 B
77 H77 1.3541 -2.0338 -3.4889 H
78 B78 3.2720 -1.5283 -1.8206 B
79 H79 3.4533 -2.6770 -1.5480 H
80 B80 3.7034 -0.8226 -3.3997 B
81 H81 4.2692 -1.4323 -4.2587 H
82 B82 4.5719 -0.2359 -1.9704 B
83 H83 5.7466 -0.4477 -1.8933 H
84 H84 4.0229 -0.7413 -0.9438 H
85 B85 2.4095 0.3554 -3.8284 B
86 H86 2.0745 0.5686 -4.9541 H
87 B87 3.9439 0.9444 -3.1293 B
88 H88 4.7541 1.5509 -3.7671 H
89 H89 -1.3257 2.7325 -1.7761 H
90 H90 -1.4415 -2.1410 2.2116 H
@<TRIPOS>BOND
1 2 24 1
2 2 35 1
3 2 69 1
4 3 46 1
5 3 57 1
6 3 68 1
7 4 6 1
8 4 7 Ar
9 5 22 Ar
10 5 23 Ar
11 6 12 Ar
12 6 23 Ar
13 7 8 2
14 7 90 1
15 8 9 1
16 8 10 Ar

17 10 11 1
18 10 12 Ar
19 12 13 Ar
20 13 14 1
21 13 15 2
22 15 16 1
23 15 17 Ar
24 17 18 Ar
25 17 23 Ar
26 18 19 1
27 18 20 Ar
28 20 21 1
29 20 22 2
30 22 89 1
31 24 25 Ar
32 24 33 Ar
33 25 26 1
34 25 27 Ar
35 27 28 1
36 27 29 Ar
37 29 30 1
38 29 31 Ar
39 31 32 1
40 31 33 Ar
41 33 34 1
42 35 36 Ar
43 35 44 Ar
44 36 37 1
45 36 38 Ar
46 38 39 1
47 38 40 Ar
48 40 41 1
49 40 42 Ar
50 42 43 1
51 42 44 Ar
52 44 45 1
53 46 47 Ar
54 46 55 Ar
55 47 48 1
56 47 49 Ar
57 49 50 1
58 49 51 Ar
59 51 52 1
60 51 53 Ar
61 53 54 1
62 53 55 Ar
63 55 56 1
64 57 58 Ar
65 57 66 Ar
66 58 59 1
67 58 60 Ar
68 60 61 1
69 60 62 Ar
70 62 63 1
71 62 64 Ar
72 64 65 1
73 64 66 Ar
74 66 67 1
75 68 69 1
76 68 70 1

77 68 72 1
78 68 74 1
79 69 74 1
80 69 76 1
81 69 78 1
82 70 71 1
83 70 72 1
84 70 82 1
85 70 87 1
86 72 73 1
87 72 74 1
88 72 85 1
89 72 87 1
90 74 75 1
91 74 76 1
92 74 85 1
93 76 77 1
94 76 78 1
95 76 80 1
96 76 85 1
97 78 79 1
98 78 80 1
99 78 82 1
100 80 81 1
101 80 82 1
102 80 85 1
103 80 87 1
104 82 83 1
105 82 87 1
106 85 86 1
107 85 87 1
108 87 88 1

Cu-2 S_0
@<TRIPOS>MOLECULE
Molecule Name
96 116
SMALL
NO_CHARGES

@<TRIPOS>ATOM
1 Cu1 -0.7880 0.0125 -0.1462 Cu
2 P2 0.8854 -1.6379 -0.1410 P
3 P3 0.8986 1.6433 -0.0981 P
4 N4 -2.3571 -0.0380 1.3286 N
5 N5 -2.5368 0.0431 -1.3937 N
6 C6 -3.5906 -0.0286 0.7671 C
7 C7 -2.2440 -0.0802 2.6533 C
8 C8 -3.3858 -0.1107 3.4868 C
9 H9 -3.2486 -0.1418 4.5634 H
10 C10 -4.6403 -0.1013 2.9341 C
11 H11 -5.5292 -0.1241 3.5599 H
12 C12 -4.7784 -0.0592 1.5324 C
13 C13 -6.0527 -0.0453 0.8877 C
14 H14 -6.9471 -0.0697 1.5048 H
15 C15 -6.1430 -0.0025 -0.4671 C
16 H16 -7.1109 0.0080 -0.9612 H
17 C17 -4.9651 0.0290 -1.2748 C
18 C18 -5.0161 0.0731 -2.6804 C

19	H19	-5.9804	0.0845	-3.1826	H
20	C20	-3.8459	0.1016	-3.3978	C
21	H21	-3.8564	0.1362	-4.4824	H
22	C22	-2.6053	0.0856	-2.7258	C
23	C23	-3.6847	0.0160	-0.6720	C
24	C24	-0.8835	-0.1133	3.2651	C
25	H25	-0.6697	-1.1102	3.6704	H
26	H26	-0.8043	0.6083	4.0853	H
27	H27	-0.1195	0.1210	2.5252	H
28	C28	-1.3289	0.1119	-3.4997	C
29	H29	-0.7065	0.9609	-3.1993	H
30	H30	-1.5167	0.1807	-4.5743	H
31	H31	-0.7393	-0.7906	-3.3079	H
32	C32	0.6629	-3.1483	-1.1526	C
33	C33	1.5970	-4.1931	-1.1610	C
34	H34	2.5160	-4.1002	-0.5895	H
35	C35	1.3536	-5.3422	-1.9038	C
36	H36	2.0879	-6.1431	-1.9113	H
37	C37	0.1754	-5.4672	-2.6402	C
38	H38	-0.0094	-6.3676	-3.2201	H
39	C39	-0.7645	-4.4423	-2.6257	C
40	H40	-1.6894	-4.5395	-3.1883	H
41	C41	-0.5214	-3.2890	-1.8809	C
42	H42	-1.2640	-2.4954	-1.8445	H
43	C43	1.1745	-2.3653	1.5197	C
44	C44	0.3486	-3.4188	1.9369	C
45	H45	-0.3651	-3.8544	1.2421	H
46	C46	0.4407	-3.9249	3.2294	C
47	H47	-0.2002	-4.7498	3.5302	H
48	C48	1.3568	-3.3848	4.1311	C
49	H49	1.4331	-3.7834	5.1392	H
50	C50	2.1789	-2.3370	3.7266	C
51	H51	2.9032	-1.9125	4.4166	H
52	C52	2.0870	-1.8306	2.4324	C
53	H53	2.7394	-1.0189	2.1332	H
54	C54	1.1904	2.3060	1.5900	C
55	C55	2.2371	1.8921	2.4174	C
56	H56	3.0108	1.2414	2.0242	H
57	C57	2.3220	2.3479	3.7321	C
58	H58	3.1501	2.0235	4.3568	H
59	C59	1.3680	3.2259	4.2363	C
60	H60	1.4410	3.5858	5.2592	H
61	C61	0.3250	3.6529	3.4146	C
62	H62	-0.4179	4.3506	3.7927	H
63	C63	0.2341	3.1929	2.1060	C
64	H64	-0.5826	3.5349	1.4738	H
65	C65	0.6524	3.1898	-1.0484	C
66	C66	-0.5748	3.3731	-1.6925	C
67	H67	-1.3254	2.5876	-1.6391	H
68	C68	-0.8482	4.5564	-2.3773	C
69	H69	-1.8063	4.6863	-2.8740	H
70	C70	0.1050	5.5681	-2.4172	C
71	H71	-0.1029	6.4919	-2.9508	H
72	C72	1.3271	5.3987	-1.7662	C
73	H73	2.0724	6.1888	-1.7939	H
74	C74	1.6010	4.2206	-1.0817	C
75	H75	2.5556	4.0916	-0.5807	H
76	C76	2.4608	0.8169	-0.6368	C
77	C77	2.4454	-0.7891	-0.6406	C
78	B78	3.9179	1.4812	-0.3473	B

```
79 H79     4.0055     2.5383     0.2084 H
80 B80     3.3221     1.4479    -2.0058 B
81 H81     2.9393     2.4147    -2.5846 H
82 B82     2.3150    -0.0016    -2.1459 B
83 H83     1.2853    -0.0327    -2.7362 H
84 B84     3.3694    -1.3989    -1.9547 B
85 H85     3.0568    -2.4327    -2.4507 H
86 B86     3.9424    -1.3604    -0.2582 B
87 H87     4.0776    -2.4018     0.3136 H
88 B88     4.9992    -0.8541    -1.5985 B
89 H89     5.9398    -1.4922    -1.9643 H
90 B90     5.0630     0.0977    -0.1015 B
91 H91     6.0762     0.0963     0.5324 H
92 H92     4.1366    -0.3252     0.6562 H
93 B93     3.9614     0.0037    -2.7923 B
94 H94     4.1563    -0.0294    -3.9686 H
95 B95     4.9537     0.9488    -1.6467 B
96 H96     5.9153     1.5715    -1.9847 H
@<TRIPOS>BOND
1 1 2 1
2 1 3 1
3 2 32 1
4 2 43 1
5 2 77 1
6 3 54 1
7 3 65 1
8 3 76 1
9 4 6 Ar
10 4 7 Ar
11 5 22 Ar
12 5 23 Ar
13 6 12 Ar
14 6 23 Ar
15 7 8 Ar
16 7 24 1
17 8 9 1
18 8 10 2
19 10 11 1
20 10 12 Ar
21 12 13 Ar
22 13 14 1
23 13 15 2
24 15 16 1
25 15 17 Ar
26 17 18 Ar
27 17 23 Ar
28 18 19 1
29 18 20 2
30 20 21 1
31 20 22 Ar
32 22 28 1
33 24 25 1
34 24 26 1
35 24 27 1
36 28 29 1
37 28 30 1
38 28 31 1
39 32 33 Ar
40 32 41 Ar
41 33 34 1
```

42 33 35 Ar
43 35 36 1
44 35 37 Ar
45 37 38 1
46 37 39 Ar
47 39 40 1
48 39 41 Ar
49 41 42 1
50 43 44 Ar
51 43 52 Ar
52 44 45 1
53 44 46 Ar
54 46 47 1
55 46 48 Ar
56 48 49 1
57 48 50 Ar
58 50 51 1
59 50 52 Ar
60 52 53 1
61 54 55 Ar
62 54 63 Ar
63 55 56 1
64 55 57 Ar
65 57 58 1
66 57 59 Ar
67 59 60 1
68 59 61 Ar
69 61 62 1
70 61 63 Ar
71 63 64 1
72 65 66 Ar
73 65 74 Ar
74 66 67 1
75 66 68 Ar
76 68 69 1
77 68 70 Ar
78 70 71 1
79 70 72 Ar
80 72 73 1
81 72 74 Ar
82 74 75 1
83 76 77 1
84 76 78 1
85 76 80 1
86 76 82 1
87 77 82 1
88 77 84 1
89 77 86 1
90 78 79 1
91 78 80 1
92 78 90 1
93 78 95 1
94 80 81 1
95 80 82 1
96 80 93 1
97 80 95 1
98 82 83 1
99 82 84 1
100 82 93 1
101 84 85 1

102 84 86 1
103 84 88 1
104 84 93 1
105 86 87 1
106 86 88 1
107 86 90 1
108 88 89 1
109 88 90 1
110 88 93 1
111 88 95 1
112 90 91 1
113 90 95 1
114 93 94 1
115 93 95 1
116 95 96 1

Cu-2 S_1
@<TRIPOS>MOLECULE
Molecule Name
96 114
SMALL
NO_CHARGES

@<TRIPOS>ATOM
1 Cu1 -0.8204 -0.0490 0.0105 Cu
2 P2 1.0492 1.4690 0.4273 P
3 P3 0.8895 -1.6615 -0.2474 P
4 N4 -2.2590 0.7609 -1.2335 N
5 N5 -2.4937 -0.6050 1.1577 N
6 C6 -3.5182 0.5396 -0.7049 C
7 C7 -2.1466 1.2716 -2.4688 C
8 C8 -3.2614 1.6587 -3.2170 C
9 H9 -3.1154 2.0817 -4.2052 H
10 C10 -4.5381 1.5296 -2.6680 C
11 H11 -5.4163 1.8562 -3.2193 H
12 C12 -4.6912 0.9589 -1.4082 C
13 C13 -5.9720 0.7693 -0.7954 C
14 H14 -6.8556 1.1033 -1.3340 H
15 C15 -6.0820 0.1921 0.4274 C
16 H16 -7.0555 0.0621 0.8942 H
17 C17 -4.9251 -0.2745 1.1363 C
18 C18 -5.0094 -0.8840 2.3826 C
19 H19 -5.9803 -1.0226 2.8507 H
20 C20 -3.8367 -1.3065 3.0221 C
21 H21 -3.8724 -1.7734 4.0005 H
22 C22 -2.6080 -1.1484 2.3899 C
23 C23 -3.6348 -0.1157 0.5386 C
24 C24 -0.7789 1.4633 -3.0454 C
25 H25 -0.4020 2.4687 -2.8265 H
26 H26 -0.8043 1.3487 -4.1330 H
27 H27 -0.0691 0.7398 -2.6380 H
28 C28 -1.3607 -1.5681 3.1040 C
29 H29 -0.6465 -2.0520 2.4350 H
30 H30 -1.5936 -2.2595 3.9184 H
31 H31 -0.8595 -0.6967 3.5428 H
32 C32 0.5879 2.4753 1.8761 C
33 C33 1.3704 3.5651 2.2852 C
34 H34 2.2715 3.8262 1.7376 H
35 C35 0.9936 4.3158 3.3927 C

36	H36	1.6068	5.1557	3.7065	H
37	C37	-0.1656	3.9916	4.0981	C
38	H38	-0.4572	4.5820	4.9622	H
39	C39	-0.9517	2.9173	3.6918	C
40	H40	-1.8600	2.6668	4.2321	H
41	C41	-0.5778	2.1611	2.5824	C
42	H42	-1.2059	1.3337	2.2607	H
43	C43	1.4976	2.6949	-0.8363	C
44	C44	0.8010	3.9133	-0.8710	C
45	H45	0.0736	4.1505	-0.1009	H
46	C46	1.0391	4.8312	-1.8894	C
47	H47	0.4994	5.7737	-1.8970	H
48	C48	1.9630	4.5429	-2.8919	C
49	H49	2.1484	5.2604	-3.6860	H
50	C50	2.6462	3.3281	-2.8733	C
51	H51	3.3668	3.0942	-3.6517	H
52	C52	2.4130	2.4084	-1.8568	C
53	H53	2.9522	1.4684	-1.8617	H
54	C54	1.1097	-1.6801	-2.0562	C
55	C55	2.2534	-1.1991	-2.7020	C
56	H56	3.1068	-0.8687	-2.1196	H
57	C57	2.3193	-1.1905	-4.0933	C
58	H58	3.2172	-0.8263	-4.5843	H
59	C59	1.2474	-1.6581	-4.8497	C
60	H60	1.3033	-1.6517	-5.9344	H
61	C61	0.1051	-2.1422	-4.2121	C
62	H62	-0.7312	-2.5146	-4.7965	H
63	C63	0.0321	-2.1507	-2.8244	C
64	H64	-0.8616	-2.5328	-2.3369	H
65	C65	0.4685	-3.3929	0.1396	C
66	C66	-0.8805	-3.6849	0.3753	C
67	H67	-1.6125	-2.8814	0.4115	H
68	C68	-1.2896	-5.0042	0.5612	C
69	H69	-2.3381	-5.2206	0.7445	H
70	C70	-0.3559	-6.0347	0.5154	C
71	H71	-0.6727	-7.0630	0.6649	H
72	C72	0.9874	-5.7485	0.2708	C
73	H73	1.7170	-6.5521	0.2271	H
74	C74	1.4024	-4.4360	0.0746	C
75	H75	2.4470	-4.2245	-0.1263	H
76	C76	2.4538	-1.1043	0.5231	C
77	C77	2.5345	0.4519	0.8246	C
78	B78	3.8721	-1.8173	0.1689	B
79	H79	3.9205	-2.7460	-0.5834	H
80	B80	3.1763	-2.0491	1.7782	B
81	H81	2.6931	-3.0761	2.1382	H
82	B82	2.2639	-0.5739	2.1545	B
83	H83	1.2027	-0.5650	2.6851	H
84	B84	3.4182	0.7491	2.2650	B
85	H85	3.1505	1.7027	2.9220	H
86	B86	4.0837	0.9821	0.6176	B
87	H87	4.3198	2.0995	0.2727	H
88	B88	5.0242	0.1554	1.8860	B
89	H89	5.9826	0.6434	2.4085	H
90	B90	5.1120	-0.5026	0.2417	B
91	H91	6.1615	-0.4658	-0.3310	H
92	H92	4.2724	0.1360	-0.4691	H
93	B93	3.8668	-0.8266	2.8525	B
94	H94	4.0036	-1.0270	4.0213	H
95	B95	4.8568	-1.6164	1.5933	B

96 H96 5.7570 -2.3612 1.8496 H
@<TRIPOS>BOND
1 2 32 1
2 2 43 1
3 2 77 1
4 3 54 1
5 3 65 1
6 3 76 1
7 4 6 1
8 4 7 Ar
9 5 22 Ar
10 5 23 1
11 6 12 Ar
12 6 23 Ar
13 7 8 Ar
14 7 24 1
15 8 9 1
16 8 10 Ar
17 10 11 1
18 10 12 Ar
19 12 13 Ar
20 13 14 1
21 13 15 2
22 15 16 1
23 15 17 Ar
24 17 18 Ar
25 17 23 Ar
26 18 19 1
27 18 20 Ar
28 20 21 1
29 20 22 Ar
30 22 28 1
31 24 25 1
32 24 26 1
33 24 27 1
34 28 29 1
35 28 30 1
36 28 31 1
37 32 33 Ar
38 32 41 Ar
39 33 34 1
40 33 35 Ar
41 35 36 1
42 35 37 Ar
43 37 38 1
44 37 39 Ar
45 39 40 1
46 39 41 Ar
47 41 42 1
48 43 44 Ar
49 43 52 Ar
50 44 45 1
51 44 46 Ar
52 46 47 1
53 46 48 Ar
54 48 49 1
55 48 50 Ar
56 50 51 1
57 50 52 Ar
58 52 53 1

59 54 55 Ar
60 54 63 Ar
61 55 56 1
62 55 57 Ar
63 57 58 1
64 57 59 Ar
65 59 60 1
66 59 61 Ar
67 61 62 1
68 61 63 Ar
69 63 64 1
70 65 66 Ar
71 65 74 Ar
72 66 67 1
73 66 68 Ar
74 68 69 1
75 68 70 Ar
76 70 71 1
77 70 72 Ar
78 72 73 1
79 72 74 Ar
80 74 75 1
81 76 77 1
82 76 78 1
83 76 80 1
84 76 82 1
85 77 82 1
86 77 84 1
87 77 86 1
88 78 79 1
89 78 80 1
90 78 90 1
91 78 95 1
92 80 81 1
93 80 82 1
94 80 93 1
95 80 95 1
96 82 83 1
97 82 84 1
98 82 93 1
99 84 85 1
100 84 86 1
101 84 88 1
102 84 93 1
103 86 87 1
104 86 88 1
105 86 90 1
106 88 89 1
107 88 90 1
108 88 93 1
109 88 95 1
110 90 91 1
111 90 95 1
112 93 94 1
113 93 95 1
114 95 96 1

Cu-2 T_1
@<TRIPOS>MOLECULE
Molecule Name

96 114
SMALL
NO_CHARGES

@<TRIPOS>ATOM
1 Cu1 -0.8145 -0.0517 -0.0655 Cu
2 P2 1.1649 -1.4012 -0.4177 P
3 P3 0.7339 1.7026 0.2279 P
4 N4 -2.1999 -0.8893 1.2167 N
5 N5 -2.4790 0.3297 -1.2133 N
6 C6 -3.4725 -0.7606 0.6877 C
7 C7 -2.0595 -1.4111 2.4488 C
8 C8 -3.1500 -1.8575 3.1961 C
9 H9 -2.9792 -2.2669 4.1861 H
10 C10 -4.4362 -1.7843 2.6606 C
11 H11 -5.2946 -2.1446 3.2217 H
12 C12 -4.6217 -1.2255 1.3998 C
13 C13 -5.9121 -1.0801 0.7998 C
14 H14 -6.7799 -1.4426 1.3454 H
15 C15 -6.0509 -0.4978 -0.4191 C
16 H16 -7.0329 -0.3918 -0.8739 H
17 C17 -4.9156 -0.0024 -1.1428 C
18 C18 -5.0318 0.6033 -2.3902 C
19 H19 -6.0100 0.7246 -2.8466 H
20 C20 -3.8673 1.0402 -3.0466 C
21 H21 -3.9253 1.4915 -4.0316 H
22 C22 -2.6291 0.8893 -2.4496 C
23 C23 -3.6172 -0.1363 -0.5648 C
24 C24 -0.6839 -1.5305 3.0236 C
25 H25 -0.2479 -2.5078 2.7883 H
26 H26 -0.7138 -1.4355 4.1130 H
27 H27 -0.0192 -0.7599 2.6272 H
28 C28 -1.3947 1.3020 -3.1859 C
29 H29 -0.7506 1.9454 -2.5802 H
30 H30 -1.6508 1.8426 -4.1010 H
31 H31 -0.8024 0.4238 -3.4687 H
32 C32 0.8076 -2.4753 -1.8457 C
33 C33 1.7205 -3.4556 -2.2629 C
34 H34 2.6613 -3.5861 -1.7359 H
35 C35 1.4196 -4.2674 -3.3505 C
36 H36 2.1324 -5.0214 -3.6714 H
37 C37 0.2079 -4.1152 -4.0253 C
38 H38 -0.0229 -4.7531 -4.8738 H
39 C39 -0.7074 -3.1539 -3.6074 C
40 H40 -1.6566 -3.0392 -4.1227 H
41 C41 -0.4105 -2.3360 -2.5187 C
42 H42 -1.1379 -1.5980 -2.1873 H
43 C43 1.7131 -2.5516 0.8805 C
44 C44 1.1876 -3.8514 0.9144 C
45 H45 0.5223 -4.1953 0.1287 H
46 C46 1.5180 -4.7146 1.9556 C
47 H47 1.1112 -5.7216 1.9647 H
48 C48 2.3645 -4.2904 2.9774 C
49 H49 2.6219 -4.9662 3.7880 H
50 C50 2.8773 -2.9946 2.9579 C
51 H51 3.5353 -2.6542 3.7521 H
52 C52 2.5505 -2.1294 1.9197 C
53 H53 2.9511 -1.1222 1.9254 H
54 C54 0.9038 1.7655 2.0432 C

55 C55 2.0796 1.4420 2.7272 C
56 H56 2.9835 1.2060 2.1756 H
57 C57 2.1069 1.4686 4.1201 C
58 H58 3.0280 1.2251 4.6418 H
59 C59 0.9657 1.8167 4.8378 C
60 H60 0.9917 1.8386 5.9235 H
61 C61 -0.2091 2.1443 4.1610 C
62 H62 -1.1008 2.4217 4.7155 H
63 C63 -0.2440 2.1157 2.7721 C
64 H64 -1.1656 2.3700 2.2538 H
65 C65 0.1728 3.3846 -0.1962 C
66 C66 -1.1822 3.5608 -0.4999 C
67 H67 -1.8420 2.6991 -0.5606 H
68 C68 -1.6898 4.8405 -0.7170 C
69 H69 -2.7424 4.9671 -0.9533 H
70 C70 -0.8492 5.9460 -0.6338 C
71 H71 -1.2435 6.9434 -0.8072 H
72 C72 0.4994 5.7749 -0.3208 C
73 H73 1.1561 6.6370 -0.2479 H
74 C74 1.0120 4.5027 -0.0934 C
75 H75 2.0595 4.3807 0.1608 H
76 C76 2.3576 1.2764 -0.5016 C
77 C77 2.5688 -0.2678 -0.8026 C
78 B78 3.7067 2.0989 -0.1125 B
79 H79 3.6640 3.0304 0.6363 H
80 B80 3.0370 2.2775 -1.7387 B
81 H81 2.4844 3.2640 -2.1111 H
82 B82 2.2537 0.7359 -2.1386 B
83 H83 1.2136 0.6473 -2.6995 H
84 B84 3.5109 -0.4913 -2.2204 B
85 H85 3.3354 -1.4601 -2.8856 H
86 B86 4.1486 -0.6767 -0.5565 B
87 H87 4.4619 -1.7732 -0.2061 H
88 B88 5.0541 0.2257 -1.7983 B
89 H89 6.0615 -0.1836 -2.2956 H
90 B90 5.0460 0.8828 -0.1522 B
91 H91 6.0801 0.9264 0.4472 H
92 H92 4.2407 0.1798 0.5359 H
93 B93 3.8494 1.1159 -2.7938 B
94 H94 3.9995 1.3295 -3.9585 H
95 B95 4.7409 1.9768 -1.5093 B
96 H96 5.5864 2.7904 -1.7415 H
@<TRIPOS>BOND
1 2 32 1
2 2 43 1
3 2 77 1
4 3 54 1
5 3 65 1
6 3 76 1
7 4 6 1
8 4 7 Ar
9 5 22 Ar
10 5 23 1
11 6 12 Ar
12 6 23 Ar
13 7 8 Ar
14 7 24 1
15 8 9 1
16 8 10 Ar
17 10 11 1

18 10 12 Ar
19 12 13 Ar
20 13 14 1
21 13 15 2
22 15 16 1
23 15 17 Ar
24 17 18 Ar
25 17 23 Ar
26 18 19 1
27 18 20 Ar
28 20 21 1
29 20 22 2
30 22 28 1
31 24 25 1
32 24 26 1
33 24 27 1
34 28 29 1
35 28 30 1
36 28 31 1
37 32 33 Ar
38 32 41 Ar
39 33 34 1
40 33 35 Ar
41 35 36 1
42 35 37 Ar
43 37 38 1
44 37 39 Ar
45 39 40 1
46 39 41 Ar
47 41 42 1
48 43 44 Ar
49 43 52 Ar
50 44 45 1
51 44 46 Ar
52 46 47 1
53 46 48 Ar
54 48 49 1
55 48 50 Ar
56 50 51 1
57 50 52 Ar
58 52 53 1
59 54 55 Ar
60 54 63 Ar
61 55 56 1
62 55 57 Ar
63 57 58 1
64 57 59 Ar
65 59 60 1
66 59 61 Ar
67 61 62 1
68 61 63 Ar
69 63 64 1
70 65 66 Ar
71 65 74 Ar
72 66 67 1
73 66 68 Ar
74 68 69 1
75 68 70 Ar
76 70 71 1
77 70 72 Ar

78 72 73 1
79 72 74 Ar
80 74 75 1
81 76 77 1
82 76 78 1
83 76 80 1
84 76 82 1
85 77 82 1
86 77 84 1
87 77 86 1
88 78 79 1
89 78 80 1
90 78 90 1
91 78 95 1
92 80 81 1
93 80 82 1
94 80 93 1
95 80 95 1
96 82 83 1
97 82 84 1
98 82 93 1
99 84 85 1
100 84 86 1
101 84 88 1
102 84 93 1
103 86 87 1
104 86 88 1
105 86 90 1
106 88 89 1
107 88 90 1
108 88 93 1
109 88 95 1
110 90 91 1
111 90 95 1
112 93 94 1
113 93 95 1
114 95 96 1

Cu-3 S_0
@<TRIPOS>MOLECULE
Molecule Name
116 138
SMALL
NO_CHARGES

@<TRIPOS>ATOM
1 Cu1 0.4206 -0.1873 -0.0049 Cu
2 P2 2.0646 -0.0997 1.6711 P
3 P3 2.1421 -0.1927 -1.6059 P
4 N4 -1.3154 -1.4391 -0.0196 N
5 N5 -1.1641 1.2590 -0.0669 N
6 C6 3.6936 -0.6816 -0.7297 C
7 C7 3.6460 -0.6222 0.8749 C
8 C8 1.8220 -1.0460 3.2199 C
9 C9 0.6362 -1.7693 3.3743 C
10 H10 -0.0945 -1.7687 2.5687 H
11 C11 0.3765 -2.4637 4.5551 C
12 H12 -0.5489 -3.0235 4.6625 H
13 C13 1.3008 -2.4319 5.5937 C

14	H14	1.1030	-2.9724	6.5157	H
15	C15	2.4800	-1.6996	5.4547	C
16	H16	3.2019	-1.6707	6.2664	H
17	C17	2.7401	-1.0073	4.2781	C
18	H18	3.6594	-0.4381	4.1741	H
19	C19	2.3311	1.5931	2.3304	C
20	C20	1.4910	2.0492	3.3562	C
21	H21	0.7791	1.3681	3.8157	H
22	C22	1.5666	3.3639	3.8046	C
23	H23	0.9141	3.6956	4.6083	H
24	C24	2.4801	4.2490	3.2338	C
25	H25	2.5429	5.2747	3.5873	H
26	C26	3.3166	3.8057	2.2135	C
27	H27	4.0390	4.4828	1.7655	H
28	C28	3.2409	2.4895	1.7644	C
29	H29	3.9030	2.1601	0.9723	H
30	C30	1.9316	-1.2126	-3.1140	C
31	C31	0.7114	-1.8708	-3.2935	C
32	H32	-0.0550	-1.7879	-2.5261	H
33	C33	0.4648	-2.6066	-4.4518	C
34	H34	-0.4883	-3.1135	-4.5791	H
35	C35	1.4379	-2.6844	-5.4421	C
36	H36	1.2509	-3.2580	-6.3462	H
37	C37	2.6530	-2.0194	-5.2770	C
38	H38	3.4139	-2.0763	-6.0507	H
39	C39	2.9002	-1.2838	-4.1241	C
40	H40	3.8492	-0.7713	-3.9984	H
41	C41	2.4359	1.4645	-2.3426	C
42	C42	1.4972	1.9288	-3.2756	C
43	H43	0.6924	1.2744	-3.6033	H
44	C44	1.5910	3.2136	-3.7979	C
45	H45	0.8622	3.5512	-4.5305	H
46	C46	2.6189	4.0629	-3.3887	C
47	H47	2.6943	5.0671	-3.7976	H
48	C48	3.5551	3.6100	-2.4648	C
49	H49	4.3716	4.2561	-2.1530	H
50	C50	3.4673	2.3191	-1.9462	C
51	H51	4.2277	1.9639	-1.2592	H
52	C52	-2.4796	-0.7437	-0.0569	C
53	C53	-1.3616	-2.7720	-0.0088	C
54	C54	-2.5853	-3.4635	-0.0131	C
55	H55	-2.5744	-4.5482	0.0316	H
56	C56	-3.7914	-2.7906	-0.0316	C
57	C57	-3.7506	-1.3698	-0.0787	C
58	C58	-4.9234	-0.5636	-0.1987	C
59	H59	-5.8852	-1.0552	-0.2974	H
60	C60	-4.8463	0.7932	-0.2205	C
61	H61	-5.7468	1.3868	-0.3360	H
62	C62	-3.5896	1.4642	-0.1246	C
63	C63	-3.4667	2.8828	-0.1235	C
64	C64	-2.1933	3.4122	-0.1267	C
65	H65	-2.0560	4.4897	-0.1163	H
66	C66	-1.0545	2.5835	-0.0961	C
67	C67	-2.3993	0.7006	-0.0814	C
68	C68	-5.0557	-3.5605	-0.0022	C
69	C69	-6.0393	-3.3099	0.9636	C
70	H70	-5.8811	-2.5247	1.6982	H
71	C71	-7.1978	-4.0787	1.0067	C
72	H72	-7.9465	-3.8806	1.7687	H
73	C73	-7.3942	-5.1030	0.0833	C

74 H74 -8.3011 -5.7002 0.1165 H
75 C75 -6.4222 -5.3606 -0.8800 C
76 H76 -6.5695 -6.1565 -1.6048 H
77 C77 -5.2590 -4.5990 -0.9191 C
78 H78 -4.5050 -4.7953 -1.6770 H
79 C79 -4.6388 3.7878 -0.1102 C
80 C80 -4.7487 4.8089 -1.0616 C
81 H81 -3.9927 4.8967 -1.8377 H
82 C82 -5.8242 5.6910 -1.0318 C
83 H83 -5.9007 6.4721 -1.7831 H
84 C84 -6.7996 5.5717 -0.0451 C
85 H85 -7.6377 6.2625 -0.0205 H
86 C86 -6.6948 4.5652 0.9121 C
87 H87 -7.4456 4.4743 1.6922 H
88 C88 -5.6246 3.6775 0.8792 C
89 H89 -5.5357 2.9064 1.6401 H
90 B90 4.5778 -2.0660 -1.2904 B
91 H91 4.2172 -2.6844 -2.2408 H
92 B92 5.1610 -0.4052 -1.3770 B
93 H93 5.2651 0.1088 -2.4536 H
94 B94 3.5445 -2.1586 0.1438 B
95 H95 2.5210 -2.7590 0.1754 H
96 B96 4.5688 -1.9039 1.5530 B
97 H97 4.2416 -2.3632 2.5992 H
98 B98 5.1279 -0.2045 1.4585 B
99 H99 5.2384 0.4091 2.4790 H
100 B100 5.1955 -2.7901 0.1954 B
101 H101 5.3993 -3.9625 0.2784 H
102 B102 6.1965 -1.6731 -0.7743 B
103 H103 7.1730 -2.0264 -1.3647 H
104 B104 6.2766 -0.0950 0.0177 B
105 H105 7.2845 0.5475 0.0140 H
106 H106 5.3360 0.6703 0.3934 H
107 B107 6.2064 -1.5546 1.0262 B
108 H108 7.1370 -1.8865 1.6966 H
109 C109 -0.0736 -3.5261 0.0279 C
110 H110 0.5578 -3.2625 -0.8266 H
111 H111 0.4984 -3.2770 0.9277 H
112 H112 -0.2441 -4.6057 0.0144 H
113 C113 0.3039 3.2008 -0.0563 C
114 H114 0.4864 3.6631 0.9217 H
115 H115 1.0748 2.4496 -0.2230 H
116 H116 0.4058 3.9779 -0.8213 H
@<TRIPOS>BOND
1 1 2 1
2 1 3 1
3 2 7 1
4 2 8 1
5 2 19 1
6 3 6 1
7 3 30 1
8 3 41 1
9 4 52 Ar
10 4 53 Ar
11 5 66 Ar
12 5 67 Ar
13 6 7 1
14 6 90 1
15 6 92 1
16 6 94 1

17 7 94 1
18 7 96 1
19 7 98 1
20 8 9 Ar
21 8 17 Ar
22 9 10 1
23 9 11 Ar
24 11 12 1
25 11 13 Ar
26 13 14 1
27 13 15 Ar
28 15 16 1
29 15 17 Ar
30 17 18 1
31 19 20 Ar
32 19 28 Ar
33 20 21 1
34 20 22 Ar
35 22 23 1
36 22 24 Ar
37 24 25 1
38 24 26 Ar
39 26 27 1
40 26 28 Ar
41 28 29 1
42 30 31 Ar
43 30 39 Ar
44 31 32 1
45 31 33 Ar
46 33 34 1
47 33 35 Ar
48 35 36 1
49 35 37 Ar
50 37 38 1
51 37 39 Ar
52 39 40 1
53 41 42 Ar
54 41 50 Ar
55 42 43 1
56 42 44 Ar
57 44 45 1
58 44 46 Ar
59 46 47 1
60 46 48 Ar
61 48 49 1
62 48 50 Ar
63 50 51 1
64 52 57 Ar
65 52 67 Ar
66 53 54 Ar
67 53 109 1
68 54 55 1
69 54 56 2
70 56 57 Ar
71 56 68 1
72 57 58 Ar
73 58 59 1
74 58 60 2
75 60 61 1
76 60 62 Ar

77 62 63 Ar
78 62 67 Ar
79 63 64 2
80 63 79 1
81 64 65 1
82 64 66 Ar
83 66 113 1
84 68 69 Ar
85 68 77 Ar
86 69 70 1
87 69 71 Ar
88 71 72 1
89 71 73 Ar
90 73 74 1
91 73 75 Ar
92 75 76 1
93 75 77 Ar
94 77 78 1
95 79 80 Ar
96 79 88 Ar
97 80 81 1
98 80 82 Ar
99 82 83 1
100 82 84 Ar
101 84 85 1
102 84 86 Ar
103 86 87 1
104 86 88 Ar
105 88 89 1
106 90 91 1
107 90 92 1
108 90 94 1
109 90 100 1
110 90 102 1
111 92 93 1
112 92 102 1
113 92 104 1
114 94 95 1
115 94 96 1
116 94 100 1
117 96 97 1
118 96 98 1
119 96 100 1
120 96 107 1
121 98 99 1
122 98 104 1
123 98 107 1
124 100 101 1
125 100 102 1
126 100 107 1
127 102 103 1
128 102 104 1
129 102 107 1
130 104 105 1
131 104 107 1
132 107 108 1
133 109 110 1
134 109 111 1
135 109 112 1
136 113 114 1

137 113 115 1
138 113 116 1

Cu-3 S$_1$
@<TRIPOS>MOLECULE
Molecule Name
116 136
SMALL
NO_CHARGES

@<TRIPOS>ATOM
1 Cu1 0.4072 -0.1929 0.0017 Cu
2 P2 2.1445 0.2550 1.5803 P
3 P3 2.2099 -0.5363 -1.4945 P
4 N4 -1.2297 -1.4240 0.0708 N
5 N5 -1.1076 1.2774 -0.1866 N
6 C6 3.7419 -0.8824 -0.5679 C
7 C7 3.7106 -0.4571 0.9544 C
8 C8 1.6756 -0.4745 3.1825 C
9 C9 0.4833 -1.1974 3.2837 C
10 H10 -0.1528 -1.3268 2.4110 H
11 C11 0.0967 -1.7378 4.5087 C
12 H12 -0.8318 -2.2969 4.5795 H
13 C13 0.8972 -1.5580 5.6319 C
14 H14 0.5972 -1.9822 6.5862 H
15 C15 2.0827 -0.8289 5.5376 C
16 H16 2.7075 -0.6867 6.4147 H
17 C17 2.4716 -0.2823 4.3208 C
18 H18 3.3927 0.2893 4.2514 H
19 C19 2.4104 2.0097 1.9842 C
20 C20 1.5844 2.6130 2.9437 C
21 H21 0.8511 2.0196 3.4818 H
22 C22 1.7053 3.9708 3.2204 C
23 H23 1.0658 4.4237 3.9728 H
24 C24 2.6444 4.7439 2.5405 C
25 H25 2.7417 5.8030 2.7619 H
26 C26 3.4588 4.1535 1.5771 C
27 H27 4.1956 4.7483 1.0448 H
28 C28 3.3401 2.7969 1.2963 C
29 H29 3.9815 2.3502 0.5461 H
30 C30 1.8728 -1.7541 -2.8078 C
31 C31 0.5678 -2.2385 -2.9461 C
32 H32 -0.2001 -1.9474 -2.2332 H
33 C33 0.2494 -3.0907 -4.0019 C
34 H34 -0.7656 -3.4643 -4.1017 H
35 C35 1.2294 -3.4614 -4.9165 C
36 H36 0.9824 -4.1302 -5.7365 H
37 C37 2.5286 -2.9719 -4.7856 C
38 H38 3.2944 -3.2577 -5.5010 H
39 C39 2.8535 -2.1143 -3.7414 C
40 H40 3.8646 -1.7314 -3.6462 H
41 C41 2.4093 1.0162 -2.4282 C
42 C42 1.3711 1.3687 -3.3042 C
43 H43 0.5200 0.7052 -3.4351 H
44 C44 1.4290 2.5590 -4.0175 C
45 H45 0.6237 2.8167 -4.6994 H
46 C46 2.5184 3.4155 -3.8612 C
47 H47 2.5655 4.3446 -4.4227 H
48 C48 3.5509 3.0714 -2.9936 C

49	H49	4.4117	3.7250	-2.8823	H
50	C50	3.5006	1.8775	-2.2780	C
51	H51	4.3320	1.5942	-1.6409	H
52	C52	-2.4213	-0.7189	-0.0100	C
53	C53	-1.3086	-2.7846	0.1981	C
54	C54	-2.5081	-3.4516	0.2565	C
55	H55	-2.5007	-4.5269	0.4029	H
56	C56	-3.7504	-2.7663	0.2119	C
57	C57	-3.6920	-1.3598	0.0390	C
58	C58	-4.8571	-0.5579	-0.1731	C
59	H59	-5.8165	-1.0574	-0.2529	H
60	C60	-4.7941	0.7947	-0.3143	C
61	H61	-5.6992	1.3625	-0.5024	H
62	C62	-3.5468	1.4755	-0.2655	C
63	C63	-3.4232	2.8768	-0.3987	C
64	C64	-2.1438	3.4144	-0.4763	C
65	H65	-2.0075	4.4869	-0.5744	H
66	C66	-1.0132	2.5911	-0.3664	C
67	C67	-2.3558	0.6935	-0.1498	C
68	C68	-4.9966	-3.5305	0.3432	C
69	C69	-6.0566	-3.1049	1.1650	C
70	H70	-5.9551	-2.1801	1.7262	H
71	C71	-7.2091	-3.8688	1.3053	C
72	H72	-8.0075	-3.5187	1.9549	H
73	C73	-7.3374	-5.0845	0.6361	C
74	H74	-8.2389	-5.6806	0.7471	H
75	C75	-6.2921	-5.5299	-0.1709	C
76	H76	-6.3780	-6.4755	-0.7003	H
77	C77	-5.1395	-4.7672	-0.3123	C
78	H78	-4.3395	-5.1153	-0.9608	H
79	C79	-4.5920	3.7824	-0.4584	C
80	C80	-4.6817	4.7524	-1.4652	C
81	H81	-3.9097	4.7950	-2.2293	H
82	C82	-5.7548	5.6363	-1.5080	C
83	H83	-5.8136	6.3740	-2.3038	H
84	C84	-6.7525	5.5736	-0.5381	C
85	H85	-7.5898	6.2654	-0.5701	H
86	C86	-6.6693	4.6211	0.4746	C
87	H87	-7.4373	4.5728	1.2420	H
88	C88	-5.5998	3.7325	0.5145	C
89	H89	-5.5283	3.0035	1.3170	H
90	B90	4.5933	-2.3720	-0.8110	B
91	H91	4.2074	-3.1815	-1.5929	H
92	B92	5.1992	-0.7833	-1.2909	B
93	H93	5.2803	-0.5282	-2.4571	H
94	B94	3.5791	-2.1248	0.6249	B
95	H95	2.5474	-2.6783	0.8130	H
96	B96	4.6259	-1.5468	1.9189	B
97	H97	4.2975	-1.7256	3.0467	H
98	B98	5.2049	0.0781	1.4257	B
99	H99	5.3221	0.9044	2.2795	H
100	B100	5.2254	-2.7358	0.7988	B
101	H101	5.4216	-3.8573	1.1499	H
102	B102	6.2240	-1.8879	-0.4162	B
103	H103	7.1891	-2.3761	-0.9206	H
104	B104	6.3317	-0.1667	-0.0174	B
105	H105	7.3458	0.4418	-0.1825	H
106	H106	5.4124	0.6886	0.1945	H
107	B107	6.2584	-1.3503	1.3052	B
108	H108	7.1917	-1.5262	2.0269	H

```
109 C109    -0.0248    -3.5423     0.3281 C
110 H110     0.6708    -3.3078    -0.4830 H
111 H111     0.4837    -3.3000     1.2683 H
112 H112    -0.2059    -4.6205     0.3161 H
113 C113     0.3421     3.2220    -0.4283 C
114 H114     0.5460     3.7983     0.4815 H
115 H115     1.1224     2.4696    -0.5391 H
116 H116     0.4078     3.9098    -1.2779 H
@<TRIPOS>BOND
1 2 7 1
2 2 8 1
3 2 19 1
4 3 6 1
5 3 30 1
6 3 41 1
7 4 52 1
8 4 53 Ar
9 5 66 Ar
10 5 67 Ar
11 6 7 1
12 6 90 1
13 6 92 1
14 6 94 1
15 7 94 1
16 7 96 1
17 7 98 1
18 8 9 Ar
19 8 17 Ar
20 9 10 1
21 9 11 Ar
22 11 12 1
23 11 13 Ar
24 13 14 1
25 13 15 Ar
26 15 16 1
27 15 17 Ar
28 17 18 1
29 19 20 Ar
30 19 28 Ar
31 20 21 1
32 20 22 Ar
33 22 23 1
34 22 24 Ar
35 24 25 1
36 24 26 Ar
37 26 27 1
38 26 28 Ar
39 28 29 1
40 30 31 Ar
41 30 39 Ar
42 31 32 1
43 31 33 Ar
44 33 34 1
45 33 35 Ar
46 35 36 1
47 35 37 Ar
48 37 38 1
49 37 39 Ar
50 39 40 1
51 41 42 Ar
```

52 41 50 Ar
53 42 43 1
54 42 44 Ar
55 44 45 1
56 44 46 Ar
57 46 47 1
58 46 48 Ar
59 48 49 1
60 48 50 Ar
61 50 51 1
62 52 57 Ar
63 52 67 Ar
64 53 54 2
65 53 109 1
66 54 55 1
67 54 56 Ar
68 56 57 Ar
69 56 68 1
70 57 58 Ar
71 58 59 1
72 58 60 2
73 60 61 1
74 60 62 Ar
75 62 63 Ar
76 62 67 Ar
77 63 64 Ar
78 63 79 1
79 64 65 1
80 64 66 Ar
81 66 113 1
82 68 69 Ar
83 68 77 Ar
84 69 70 1
85 69 71 Ar
86 71 72 1
87 71 73 Ar
88 73 74 1
89 73 75 Ar
90 75 76 1
91 75 77 Ar
92 77 78 1
93 79 80 Ar
94 79 88 Ar
95 80 81 1
96 80 82 Ar
97 82 83 1
98 82 84 Ar
99 84 85 1
100 84 86 Ar
101 86 87 1
102 86 88 Ar
103 88 89 1
104 90 91 1
105 90 92 1
106 90 94 1
107 90 100 1
108 90 102 1
109 92 93 1
110 92 102 1
111 92 104 1

112 94 95 1
113 94 96 1
114 94 100 1
115 96 97 1
116 96 98 1
117 96 100 1
118 96 107 1
119 98 99 1
120 98 104 1
121 98 107 1
122 100 101 1
123 100 102 1
124 100 107 1
125 102 103 1
126 102 104 1
127 102 107 1
128 104 105 1
129 104 107 1
130 107 108 1
131 109 110 1
132 109 111 1
133 109 112 1
134 113 114 1
135 113 115 1
136 113 116 1

Cu-3 T_1
@<TRIPOS>MOLECULE
Molecule Name
116 136
SMALL
NO_CHARGES

@<TRIPOS>ATOM
1 Cu1 0.3994 -0.2594 -0.0274 Cu
2 P2 2.0645 -0.1435 1.6479 P
3 P3 2.1896 -0.1188 -1.5696 P
4 N4 -1.2317 -1.4179 -0.1131 N
5 N5 -1.1126 1.2791 -0.0416 N
6 C6 3.7267 -0.6618 -0.7105 C
7 C7 3.6592 -0.6525 0.8801 C
8 C8 1.6965 -1.1735 3.1067 C
9 C9 0.5106 -1.9133 3.1405 C
10 H10 -0.1720 -1.8810 2.2950 H
11 C11 0.1884 -2.6696 4.2667 C
12 H12 -0.7352 -3.2408 4.2848 H
13 C13 1.0451 -2.6841 5.3630 C
14 H14 0.7947 -3.2735 6.2406 H
15 C15 2.2210 -1.9327 5.3418 C
16 H16 2.8858 -1.9352 6.2009 H
17 C17 2.5451 -1.1743 4.2232 C
18 H18 3.4566 -0.5833 4.2159 H
19 C19 2.2730 1.5154 2.3799 C
20 C20 1.3469 1.9147 3.3556 C
21 H21 0.5867 1.2205 3.7032 H
22 C22 1.3995 3.1962 3.8934 C
23 H23 0.6812 3.4884 4.6540 H
24 C24 2.3718 4.0983 3.4625 C
25 H25 2.4148 5.0975 3.8863 H

26	C26	3.2883	3.7120	2.4878	C
27	H27	4.0512	4.4065	2.1479	H
28	C28	3.2370	2.4304	1.9463	C
29	H29	3.9614	2.1470	1.1925	H
30	C30	1.9058	-1.0827	-3.0921	C
31	C31	0.6656	-1.7026	-3.2763	C
32	H32	-0.0939	-1.6373	-2.5007	H
33	C33	0.3917	-2.3847	-4.4612	C
34	H34	-0.5745	-2.8626	-4.5955	H
35	C35	1.3521	-2.4462	-5.4659	C
36	H36	1.1401	-2.9787	-6.3888	H
37	C37	2.5848	-1.8148	-5.2938	C
38	H38	3.3322	-1.8540	-6.0811	H
39	C39	2.8617	-1.1289	-4.1172	C
40	H40	3.8197	-0.6333	-3.9919	H
41	C41	2.4646	1.5654	-2.2162	C
42	C42	1.5402	2.0523	-3.1529	C
43	H43	0.7421	1.4106	-3.5177	H
44	C44	1.6433	3.3536	-3.6312	C
45	H45	0.9265	3.7148	-4.3633	H
46	C46	2.6639	4.1886	-3.1766	C
47	H47	2.7447	5.2047	-3.5523	H
48	C48	3.5830	3.7123	-2.2454	C
49	H49	4.3881	4.3520	-1.8950	H
50	C50	3.4851	2.4082	-1.7659	C
51	H51	4.2268	2.0401	-1.0658	H
52	C52	-2.4233	-0.7164	-0.1314	C
53	C53	-1.3076	-2.8064	-0.1933	C
54	C54	-2.5030	-3.4663	-0.2418	C
55	H55	-2.4904	-4.5514	-0.2585	H
56	C56	-3.7579	-2.7869	-0.1824	C
57	C57	-3.6943	-1.3487	-0.1904	C
58	C58	-4.8434	-0.5329	-0.3418	C
59	H59	-5.8024	-1.0115	-0.5064	H
60	C60	-4.7820	0.8430	-0.3123	C
61	H61	-5.6876	1.4257	-0.4400	H
62	C62	-3.5464	1.5005	-0.1480	C
63	C63	-3.4134	2.9179	-0.0851	C
64	C64	-2.1394	3.4442	-0.0405	C
65	H65	-1.9923	4.5183	0.0176	H
66	C66	-1.0062	2.6007	-0.0210	C
67	C67	-2.3557	0.7091	-0.1030	C
68	C68	-4.9913	-3.5605	-0.0986	C
69	C69	-6.0764	-3.1746	0.7192	C
70	H70	-5.9982	-2.2726	1.3192	H
71	C71	-7.2217	-3.9552	0.8128	C
72	H72	-8.0334	-3.6363	1.4618	H
73	C73	-7.3277	-5.1493	0.0978	C
74	H74	-8.2251	-5.7569	0.1716	H
75	C75	-6.2620	-5.5561	-0.7066	C
76	H76	-6.3295	-6.4822	-1.2719	H
77	C77	-5.1142	-4.7804	-0.8001	C
78	H78	-4.3055	-5.1027	-1.4507	H
79	C79	-4.5806	3.8278	-0.0603	C
80	C80	-4.6375	4.9173	-0.9394	C
81	H81	-3.8439	5.0556	-1.6691	H
82	C82	-5.7089	5.8045	-0.8991	C
83	H83	-5.7442	6.6377	-1.5954	H
84	C84	-6.7328	5.6236	0.0287	C
85	H85	-7.5676	6.3182	0.0620	H

86 C86 -6.6804 4.5498 0.9154 C
87 H87 -7.4693 4.4090 1.6491 H
88 C88 -5.6149 3.6559 0.8702 C
89 H89 -5.5702 2.8312 1.5762 H
90 B90 4.6073 -2.0295 -1.3103 B
91 H91 4.2583 -2.6118 -2.2877 H
92 B92 5.1995 -0.3675 -1.3385 B
93 H93 5.3153 0.1775 -2.3972 H
94 B94 3.5559 -2.1672 0.1084 B
95 H95 2.5300 -2.7593 0.1104 H
96 B96 4.5630 -1.9551 1.5372 B
97 H97 4.2220 -2.4376 2.5684 H
98 B98 5.1321 -0.2552 1.5038 B
99 H99 5.2312 0.3196 2.5460 H
100 B100 5.2047 -2.8019 0.1602 B
101 H101 5.4094 -3.9771 0.2103 H
102 B102 6.2196 -1.6592 -0.7614 B
103 H103 7.2047 -1.9985 -1.3497 H
104 B104 6.2964 -0.1070 0.0806 B
105 H105 7.3105 0.5271 0.1085 H
106 H106 5.3587 0.6538 0.4699 H
107 B107 6.2076 -1.5965 1.0403 B
108 H108 7.1309 -1.9540 1.7111 H
109 C109 -0.0199 -3.5619 -0.1917 C
110 H110 0.6300 -3.2543 -1.0182 H
111 H111 0.5406 -3.3962 0.7352 H
112 H112 -0.2018 -4.6359 -0.2868 H
113 C113 0.3473 3.2254 0.0534 C
114 H114 0.4696 3.7628 1.0014 H
115 H115 1.1312 2.4740 -0.0191 H
116 H116 0.4831 3.9502 -0.7570 H
@<TRIPOS>BOND
1 2 7 1
2 2 8 1
3 2 19 1
4 3 6 1
5 3 30 1
6 3 41 1
7 4 52 1
8 4 53 1
9 5 66 Ar
10 5 67 Ar
11 6 7 1
12 6 90 1
13 6 92 1
14 6 94 1
15 7 94 1
16 7 96 1
17 7 98 1
18 8 9 Ar
19 8 17 Ar
20 9 10 1
21 9 11 Ar
22 11 12 1
23 11 13 Ar
24 13 14 1
25 13 15 Ar
26 15 16 1
27 15 17 Ar
28 17 18 1

29 19 20 Ar
30 19 28 Ar
31 20 21 1
32 20 22 Ar
33 22 23 1
34 22 24 Ar
35 24 25 1
36 24 26 Ar
37 26 27 1
38 26 28 Ar
39 28 29 1
40 30 31 Ar
41 30 39 Ar
42 31 32 1
43 31 33 Ar
44 33 34 1
45 33 35 Ar
46 35 36 1
47 35 37 Ar
48 37 38 1
49 37 39 Ar
50 39 40 1
51 41 42 Ar
52 41 50 Ar
53 42 43 1
54 42 44 Ar
55 44 45 1
56 44 46 Ar
57 46 47 1
58 46 48 Ar
59 48 49 1
60 48 50 Ar
61 50 51 1
62 52 57 Ar
63 52 67 Ar
64 53 54 2
65 53 109 1
66 54 55 1
67 54 56 Ar
68 56 57 Ar
69 56 68 1
70 57 58 Ar
71 58 59 1
72 58 60 2
73 60 61 1
74 60 62 Ar
75 62 63 Ar
76 62 67 Ar
77 63 64 2
78 63 79 1
79 64 65 1
80 64 66 Ar
81 66 113 1
82 68 69 Ar
83 68 77 Ar
84 69 70 1
85 69 71 Ar
86 71 72 1
87 71 73 Ar
88 73 74 1

89 73 75 Ar
90 75 76 1
91 75 77 Ar
92 77 78 1
93 79 80 Ar
94 79 88 Ar
95 80 81 1
96 80 82 Ar
97 82 83 1
98 82 84 Ar
99 84 85 1
100 84 86 Ar
101 86 87 1
102 86 88 Ar
103 88 89 1
104 90 91 1
105 90 92 1
106 90 94 1
107 90 100 1
108 90 102 1
109 92 93 1
110 92 102 1
111 92 104 1
112 94 95 1
113 94 96 1
114 94 100 1
115 96 97 1
116 96 98 1
117 96 100 1
118 96 107 1
119 98 99 1
120 98 104 1
121 98 107 1
122 100 101 1
123 100 102 1
124 100 107 1
125 102 103 1
126 102 104 1
127 102 107 1
128 104 105 1
129 104 107 1
130 107 108 1
131 109 110 1
132 109 111 1
133 109 112 1
134 113 114 1
135 113 115 1
136 113 116 1

Cu-4 S_0
@<TRIPOS>MOLECULE
Molecule Name
110 132
SMALL
NO_CHARGES

@<TRIPOS>ATOM
1 Cu1 -0.4042 0.1697 -0.0837 Cu
2 P2 -1.8823 -0.1905 1.6671 P

3 P3	-2.2138	0.3798	-1.4998	P
4 N4	1.2734	1.4318	-0.0118	N
5 N5	1.1708	-1.2228	-0.3097	N
6 C6	-3.5854	0.9756	-0.4212	C
7 C7	-3.4249	0.6588	1.1288	C
8 C8	-1.5160	0.3550	3.3713	C
9 C9	-0.4534	1.2407	3.5707	C
10 H10	0.1291	1.5739	2.7150	H
11 C11	-0.1415	1.6920	4.8510	C
12 H12	0.6837	2.3845	4.9955	H
13 C13	-0.8851	1.2525	5.9421	C
14 H14	-0.6430	1.6023	6.9423	H
15 C15	-1.9414	0.3623	5.7520	C
16 H16	-2.5262	0.0215	6.6021	H
17 C17	-2.2576	-0.0852	4.4745	C
18 H18	-3.0886	-0.7691	4.3276	H
19 C19	-2.2589	-1.9683	1.8856	C
20 C20	-1.3349	-2.7577	2.5870	C
21 H21	-0.4762	-2.2887	3.0621	H
22 C22	-1.5159	-4.1320	2.6956	C
23 H23	-0.7982	-4.7271	3.2548	H
24 C24	-2.6156	-4.7432	2.0941	C
25 H25	-2.7579	-5.8173	2.1791	H
26 C26	-3.5304	-3.9692	1.3876	C
27 H27	-4.3914	-4.4345	0.9154	H
28 C28	-3.3548	-2.5909	1.2843	C
29 H29	-4.0798	-1.9994	0.7372	H
30 C30	-2.2006	1.4435	-2.9852	C
31 C31	-1.2458	2.4623	-3.0618	C
32 H32	-0.5389	2.5925	-2.2459	H
33 C33	-1.1993	3.3036	-4.1695	C
34 H34	-0.4580	4.0970	-4.2168	H
35 C35	-2.0994	3.1238	-5.2165	C
36 H36	-2.0633	3.7780	-6.0837	H
37 C37	-3.0442	2.1017	-5.1532	C
38 H38	-3.7479	1.9591	-5.9688	H
39 C39	-3.0963	1.2636	-4.0443	C
40 H40	-3.8382	0.4724	-3.9960	H
41 C41	-2.6867	-1.2598	-2.1763	C
42 C42	-1.6654	-1.9757	-2.8178	C
43 H43	-0.6708	-1.5389	-2.8902	H
44 C44	-1.9111	-3.2303	-3.3650	C
45 H45	-1.1109	-3.7674	-3.8682	H
46 C46	-3.1807	-3.7972	-3.2648	C
47 H47	-3.3748	-4.7798	-3.6867	H
48 C48	-4.1981	-3.0977	-2.6232	C
49 H49	-5.1924	-3.5302	-2.5464	H
50 C50	-3.9574	-1.8345	-2.0842	C
51 H51	-4.7628	-1.2877	-1.6040	H
52 C52	2.4528	0.7800	-0.1285	C
53 C53	1.2941	2.7508	0.1150	C
54 H54	0.3272	3.2388	0.2134	H
55 C55	2.4781	3.4930	0.1449	C
56 H56	2.4305	4.5690	0.2800	H
57 C57	3.7066	2.8560	0.0466	C
58 C58	3.7036	1.4416	-0.1131	C
59 C59	4.8883	0.6611	-0.3057	C
60 H60	5.8420	1.1729	-0.3783	H
61 C61	4.8368	-0.6934	-0.4231	C
62 H62	5.7503	-1.2553	-0.5870	H

63 C63 3.5945 -1.4030 -0.3713 C
64 C64 3.4879 -2.8194 -0.4655 C
65 C65 2.2135 -3.3651 -0.5101 C
66 H66 2.0831 -4.4411 -0.5703 H
67 C67 1.0874 -2.5388 -0.4337 C
68 H68 0.0852 -2.9578 -0.4605 H
69 C69 2.3975 -0.6563 -0.2735 C
70 C70 4.9511 3.6545 0.1095 C
71 C71 5.9679 3.3493 1.0239 C
72 H72 5.8477 2.5045 1.6970 H
73 C73 7.1101 4.1398 1.0979 C
74 H74 7.8852 3.8985 1.8201 H
75 C75 7.2562 5.2408 0.2573 C
76 H76 8.1504 5.8551 0.3145 H
77 C77 6.2501 5.5538 -0.6533 C
78 H78 6.3581 6.4100 -1.3131 H
79 C79 5.1033 4.7701 -0.7231 C
80 H80 4.3237 5.0093 -1.4418 H
81 C81 4.6702 -3.7084 -0.5010 C
82 C82 4.7844 -4.6895 -1.4936 C
83 H83 4.0229 -4.7569 -2.2664 H
84 C84 5.8724 -5.5561 -1.5096 C
85 H85 5.9523 -6.3052 -2.2923 H
86 C86 6.8562 -5.4623 -0.5282 C
87 H87 7.7039 -6.1416 -0.5395 H
88 C88 6.7471 -4.4969 0.4699 C
89 H89 7.5038 -4.4273 1.2464 H
90 C90 5.6643 -3.6242 0.4832 C
91 H91 5.5709 -2.8881 1.2774 H
92 B92 -4.2165 2.5718 -0.6923 B
93 H93 -3.8198 3.2509 -1.5868 H
94 B94 -5.1146 1.0779 -0.9557 B
95 H95 -5.3875 0.7987 -2.0878 H
96 B96 -3.0849 2.2551 0.6308 B
97 H97 -1.9518 2.6405 0.6428 H
98 B98 -4.0292 1.9523 2.0876 B
99 H99 -3.5418 2.1510 3.1542 H
100 B100 -4.9043 0.4101 1.8030 B
101 H101 -5.0469 -0.3275 2.7338 H
102 B102 -4.5739 3.1458 0.9438 B
103 H103 -4.5499 4.3069 1.2173 H
104 B104 -5.8384 2.3940 -0.0702 B
105 H105 -6.7730 3.0034 -0.4968 H
106 B106 -6.1574 0.7428 0.4844 B
107 H107 -7.2686 0.3016 0.4938 H
108 H108 -5.3612 -0.2358 0.6654 H
109 B109 -5.7374 1.9845 1.6852 B
110 H110 -6.5364 2.3623 2.4880 H
@<TRIPOS>BOND
1 1 2 1
2 1 3 1
3 2 7 1
4 2 8 1
5 2 19 1
6 3 6 1
7 3 30 1
8 3 41 1
9 4 52 Ar
10 4 53 Ar
11 5 67 Ar

12 5 69 Ar
13 6 7 1
14 6 92 1
15 6 94 1
16 6 96 1
17 7 96 1
18 7 98 1
19 7 100 1
20 8 9 Ar
21 8 17 Ar
22 9 10 1
23 9 11 Ar
24 11 12 1
25 11 13 Ar
26 13 14 1
27 13 15 Ar
28 15 16 1
29 15 17 Ar
30 17 18 1
31 19 20 Ar
32 19 28 Ar
33 20 21 1
34 20 22 Ar
35 22 23 1
36 22 24 Ar
37 24 25 1
38 24 26 Ar
39 26 27 1
40 26 28 Ar
41 28 29 1
42 30 31 Ar
43 30 39 Ar
44 31 32 1
45 31 33 Ar
46 33 34 1
47 33 35 Ar
48 35 36 1
49 35 37 Ar
50 37 38 1
51 37 39 Ar
52 39 40 1
53 41 42 Ar
54 41 50 Ar
55 42 43 1
56 42 44 Ar
57 44 45 1
58 44 46 Ar
59 46 47 1
60 46 48 Ar
61 48 49 1
62 48 50 Ar
63 50 51 1
64 52 58 Ar
65 52 69 Ar
66 53 54 1
67 53 55 Ar
68 55 56 1
69 55 57 Ar
70 57 58 Ar
71 57 70 1

72 58 59 Ar
73 59 60 1
74 59 61 2
75 61 62 1
76 61 63 Ar
77 63 64 Ar
78 63 69 Ar
79 64 65 Ar
80 64 81 1
81 65 66 1
82 65 67 Ar
83 67 68 1
84 70 71 Ar
85 70 79 Ar
86 71 72 1
87 71 73 Ar
88 73 74 1
89 73 75 Ar
90 75 76 1
91 75 77 Ar
92 77 78 1
93 77 79 Ar
94 79 80 1
95 81 82 Ar
96 81 90 Ar
97 82 83 1
98 82 84 Ar
99 84 85 1
100 84 86 Ar
101 86 87 1
102 86 88 Ar
103 88 89 1
104 88 90 Ar
105 90 91 1
106 92 93 1
107 92 94 1
108 92 96 1
109 92 102 1
110 92 104 1
111 94 95 1
112 94 104 1
113 94 106 1
114 96 97 1
115 96 98 1
116 96 102 1
117 98 99 1
118 98 100 1
119 98 102 1
120 98 109 1
121 100 101 1
122 100 106 1
123 100 109 1
124 102 103 1
125 102 104 1
126 102 109 1
127 104 105 1
128 104 106 1
129 104 109 1
130 106 107 1
131 106 109 1

132 109 110 1

Cu-4 S_1
@<TRIPOS>MOLECULE
Molecule Name
110 130
SMALL
NO_CHARGES

@<TRIPOS>ATOM
1 Cu1 -0.3005 0.1310 -0.1821 Cu
2 P2 -2.0036 -1.2711 0.8863 P
3 P3 -2.2133 1.4188 -0.7441 P
4 N4 1.2749 1.4301 -0.0565 N
5 N5 1.1873 -1.2138 -0.3747 N
6 C6 -3.3481 1.3302 0.6958 C
7 C7 -3.2194 -0.0327 1.4948 C
8 C8 -1.2594 -2.0688 2.3428 C
9 C9 -0.0205 -1.6085 2.8034 C
10 H10 0.5102 -0.8310 2.2609 H
11 C11 0.5445 -2.1532 3.9540 C
12 H12 1.5058 -1.7898 4.3054 H
13 C13 -0.1192 -3.1634 4.6451 C
14 H14 0.3238 -3.5903 5.5406 H
15 C15 -1.3522 -3.6289 4.1886 C
16 H16 -1.8734 -4.4141 4.7286 H
17 C17 -1.9240 -3.0847 3.0441 C
18 H18 -2.8902 -3.4429 2.7012 H
19 C19 -2.9207 -2.5852 0.0247 C
20 C20 -2.3713 -3.8743 -0.0542 C
21 H21 -1.4489 -4.1108 0.4691 H
22 C22 -3.0176 -4.8715 -0.7782 C
23 H23 -2.5854 -5.8671 -0.8212 H
24 C24 -4.2149 -4.5962 -1.4354 C
25 H25 -4.7195 -5.3764 -1.9981 H
26 C26 -4.7628 -3.3173 -1.3666 C
27 H27 -5.6955 -3.0928 -1.8756 H
28 C28 -4.1198 -2.3162 -0.6455 C
29 H29 -4.5576 -1.3246 -0.6126 H
30 C30 -1.9932 3.1097 -1.3972 C
31 C31 -0.8636 3.2735 -2.2136 C
32 H32 -0.1641 2.4512 -2.3455 H
33 C33 -0.6247 4.4866 -2.8509 C
34 H34 0.2547 4.6004 -3.4783 H
35 C35 -1.5071 5.5503 -2.6751 C
36 H36 -1.3198 6.5001 -3.1679 H
37 C37 -2.6307 5.3928 -1.8671 C
38 H38 -3.3238 6.2179 -1.7299 H
39 C39 -2.8805 4.1788 -1.2317 C
40 H40 -3.7634 4.0695 -0.6143 H
41 C41 -3.0031 0.5380 -2.1384 C
42 C42 -2.2443 -0.4032 -2.8427 C
43 H43 -1.2280 -0.6345 -2.5273 H
44 C44 -2.7821 -1.0570 -3.9487 C
45 H45 -2.1846 -1.7865 -4.4875 H
46 C46 -4.0847 -0.7785 -4.3529 C
47 H47 -4.5080 -1.2907 -5.2124 H
48 C48 -4.8464 0.1602 -3.6566 C
49 H49 -5.8616 0.3816 -3.9735 H

50	C50	-4.3089	0.8232	-2.5579	C
51	H51	-4.9047	1.5571	-2.0246	H
52	C52	2.4787	0.7610	-0.1003	C
53	C53	1.2886	2.7216	0.2810	C
54	H54	0.3231	3.2161	0.3356	H
55	C55	2.4503	3.4318	0.5343	C
56	H56	2.3845	4.4783	0.8108	H
57	C57	3.7061	2.7988	0.4341	C
58	C58	3.7224	1.4335	0.0875	C
59	C59	4.9101	0.6734	-0.1741	C
60	H60	5.8690	1.1803	-0.1457	H
61	C61	4.8620	-0.6458	-0.5040	C
62	H62	5.7831	-1.1660	-0.7451	H
63	C63	3.6255	-1.3744	-0.5620	C
64	C64	3.5203	-2.7484	-0.8622	C
65	C65	2.2159	-3.2858	-0.9948	C
66	H66	2.0808	-4.3375	-1.2238	H
67	C67	1.1102	-2.5035	-0.7400	C
68	H68	0.1115	-2.9269	-0.7962	H
69	C69	2.4327	-0.6249	-0.3539	C
70	C70	4.9318	3.5809	0.6941	C
71	C71	5.9337	3.1095	1.5560	C
72	H72	5.8093	2.1472	2.0451	H
73	C73	7.0654	3.8759	1.8175	C
74	H74	7.8260	3.4967	2.4947	H
75	C75	7.2179	5.1279	1.2249	C
76	H76	8.1022	5.7252	1.4288	H
77	C77	6.2255	5.6121	0.3739	C
78	H78	6.3357	6.5871	-0.0930	H
79	C79	5.0915	4.8492	0.1156	C
80	H80	4.3259	5.2275	-0.5571	H
81	C81	4.6828	-3.6353	-1.0531	C
82	C82	4.7061	-4.5364	-2.1302	C
83	H83	3.8862	-4.5323	-2.8440	H
84	C84	5.7721	-5.4115	-2.3088	C
85	H85	5.7727	-6.0923	-3.1559	H
86	C86	6.8375	-5.4130	-1.4098	C
87	H87	7.6697	-6.0977	-1.5476	H
88	C88	6.8235	-4.5333	-0.3286	C
89	H89	7.6405	-4.5373	0.3880	H
90	C90	5.7584	-3.6557	-0.1502	C
91	H91	5.7420	-2.9992	0.7155	H
92	B92	-3.5348	2.6790	1.7617	B
93	H93	-3.0041	3.7242	1.5488	H
94	B94	-4.8224	2.0062	0.7437	B
95	H95	-5.2433	2.6643	-0.1619	H
96	B96	-2.4712	1.3282	2.1923	B
97	H97	-1.2797	1.4090	2.2052	H
98	B98	-3.4028	0.2122	3.1852	B
99	H99	-2.8357	-0.5099	3.9402	H
100	B100	-4.6774	-0.4691	2.1185	B
101	H101	-4.9660	-1.6184	2.2668	H
102	B102	-3.6539	1.9256	3.3574	B
103	H103	-3.3096	2.5005	4.3454	H
104	B104	-5.1174	2.3578	2.4263	B
105	H105	-5.8702	3.2219	2.7695	H
106	B106	-5.8470	0.8988	1.7410	B
107	H107	-7.0362	0.7768	1.6941	H
108	H108	-5.3411	0.0101	0.9881	H
109	B109	-5.0523	0.7909	3.3221	B

110 H110 -5.6835 0.5832 4.3164 H
@<TRIPOS>BOND
1 2 7 1
2 2 8 1
3 2 19 1
4 3 6 1
5 3 30 1
6 3 41 1
7 4 52 Ar
8 4 53 Ar
9 5 67 Ar
10 5 69 Ar
11 6 7 1
12 6 92 1
13 6 94 1
14 6 96 1
15 7 96 1
16 7 98 1
17 7 100 1
18 8 9 Ar
19 8 17 Ar
20 9 10 1
21 9 11 Ar
22 11 12 1
23 11 13 Ar
24 13 14 1
25 13 15 Ar
26 15 16 1
27 15 17 Ar
28 17 18 1
29 19 20 Ar
30 19 28 Ar
31 20 21 1
32 20 22 Ar
33 22 23 1
34 22 24 Ar
35 24 25 1
36 24 26 Ar
37 26 27 1
38 26 28 Ar
39 28 29 1
40 30 31 Ar
41 30 39 Ar
42 31 32 1
43 31 33 Ar
44 33 34 1
45 33 35 Ar
46 35 36 1
47 35 37 Ar
48 37 38 1
49 37 39 Ar
50 39 40 1
51 41 42 Ar
52 41 50 Ar
53 42 43 1
54 42 44 Ar
55 44 45 1
56 44 46 Ar
57 46 47 1
58 46 48 Ar

59 48 49 1
60 48 50 Ar
61 50 51 1
62 52 58 Ar
63 52 69 Ar
64 53 54 1
65 53 55 2
66 55 56 1
67 55 57 Ar
68 57 58 Ar
69 57 70 1
70 58 59 Ar
71 59 60 1
72 59 61 2
73 61 62 1
74 61 63 Ar
75 63 64 Ar
76 63 69 Ar
77 64 65 Ar
78 64 81 1
79 65 66 1
80 65 67 2
81 67 68 1
82 70 71 Ar
83 70 79 Ar
84 71 72 1
85 71 73 Ar
86 73 74 1
87 73 75 Ar
88 75 76 1
89 75 77 Ar
90 77 78 1
91 77 79 Ar
92 79 80 1
93 81 82 Ar
94 81 90 Ar
95 82 83 1
96 82 84 Ar
97 84 85 1
98 84 86 Ar
99 86 87 1
100 86 88 Ar
101 88 89 1
102 88 90 Ar
103 90 91 1
104 92 93 1
105 92 94 1
106 92 96 1
107 92 102 1
108 92 104 1
109 94 95 1
110 94 104 1
111 94 106 1
112 96 97 1
113 96 98 1
114 96 102 1
115 98 99 1
116 98 100 1
117 98 102 1
118 98 109 1

119 100 101 1
120 100 106 1
121 100 109 1
122 102 103 1
123 102 104 1
124 102 109 1
125 104 105 1
126 104 106 1
127 104 109 1
128 106 107 1
129 106 109 1
130 109 110 1

Cu-4 T_1
@<TRIPOS>MOLECULE
Molecule Name
110 130
SMALL
NO_CHARGES

@<TRIPOS>ATOM
1 Cu1 -0.3266 0.1039 -0.1106 Cu
2 P2 -2.0144 -1.1769 1.0031 P
3 P3 -2.1792 1.3486 -0.8695 P
4 N4 1.2191 1.3651 0.1329 N
5 N5 1.1364 -1.2197 -0.4201 N
6 C6 -3.3907 1.3610 0.5071 C
7 C7 -3.2918 0.0744 1.4283 C
8 C8 -1.3159 -1.8297 2.5495 C
9 C9 -0.0746 -1.3530 2.9852 C
10 H10 0.4801 -0.6377 2.3825 H
11 C11 0.4605 -1.7996 4.1911 C
12 H12 1.4245 -1.4257 4.5234 H
13 C13 -0.2369 -2.7260 4.9619 C
14 H14 0.1830 -3.0763 5.9006 H
15 C15 -1.4729 -3.2072 4.5300 C
16 H16 -2.0186 -3.9282 5.1316 H
17 C17 -2.0142 -2.7620 3.3295 C
18 H18 -2.9806 -3.1338 3.0015 H
19 C19 -2.8390 -2.5861 0.2015 C
20 C20 -2.2629 -3.8624 0.2862 C
21 H21 -1.3751 -4.0246 0.8908 H
22 C22 -2.8383 -4.9373 -0.3849 C
23 H23 -2.3879 -5.9224 -0.3037 H
24 C24 -3.9887 -4.7516 -1.1484 C
25 H25 -4.4383 -5.5923 -1.6690 H
26 C26 -4.5620 -3.4853 -1.2408 C
27 H27 -5.4587 -3.3320 -1.8339 H
28 C28 -3.9902 -2.4065 -0.5738 C
29 H29 -4.4449 -1.4260 -0.6650 H
30 C30 -1.8915 2.9986 -1.5926 C
31 C31 -0.6537 3.1635 -2.2303 C
32 H32 0.0869 2.3684 -2.1944 H
33 C33 -0.3625 4.3445 -2.9065 C
34 H34 0.6012 4.4608 -3.3939 H
35 C35 -1.3010 5.3725 -2.9474 C
36 H36 -1.0729 6.2974 -3.4697 H
37 C37 -2.5341 5.2120 -2.3190 C
38 H38 -3.2710 6.0095 -2.3514 H

39	C39	-2.8362	4.0301	-1.6483	C
40	H40	-3.8031	3.9175	-1.1727	H
41	C41	-2.8869	0.3749	-2.2447	C
42	C42	-2.0574	-0.5582	-2.8777	C
43	H43	-1.0395	-0.7161	-2.5242	H
44	C44	-2.5255	-1.2937	-3.9636	C
45	H45	-1.8737	-2.0156	-4.4471	H
46	C46	-3.8275	-1.1061	-4.4195	C
47	H47	-4.1963	-1.6826	-5.2632	H
48	C48	-4.6580	-0.1756	-3.7952	C
49	H49	-5.6728	-0.0248	-4.1523	H
50	C50	-4.1916	0.5692	-2.7163	C
51	H51	-4.8428	1.2940	-2.2387	H
52	C52	2.4327	0.7234	-0.0131	C
53	C53	1.2328	2.6686	0.4556	C
54	H54	0.2637	3.1455	0.5701	H
55	C55	2.3929	3.3881	0.6482	C
56	H56	2.3281	4.4299	0.9433	H
57	C57	3.6570	2.7677	0.5033	C
58	C58	3.6738	1.4075	0.1417	C
59	C59	4.8604	0.6544	-0.1510	C
60	H60	5.8165	1.1673	-0.1427	H
61	C61	4.8166	-0.6676	-0.4702	C
62	H62	5.7380	-1.1844	-0.7173	H
63	C63	3.5824	-1.3997	-0.5226	C
64	C64	3.4780	-2.7703	-0.8287	C
65	C65	2.1736	-3.3113	-0.9550	C
66	H66	2.0410	-4.3626	-1.1872	H
67	C67	1.0648	-2.5241	-0.7397	C
68	H68	0.0658	-2.9427	-0.8230	H
69	C69	2.3892	-0.6484	-0.3166	C
70	C70	4.8792	3.5628	0.7347	C
71	C71	5.9211	3.0931	1.5497	C
72	H72	5.8309	2.1202	2.0252	H
73	C73	7.0493	3.8732	1.7846	C
74	H74	7.8405	3.4932	2.4254	H
75	C75	7.1594	5.1397	1.2139	C
76	H76	8.0406	5.7481	1.3978	H
77	C77	6.1274	5.6236	0.4112	C
78	H78	6.2032	6.6101	-0.0385	H
79	C79	4.9981	4.8459	0.1780	C
80	H80	4.2026	5.2248	-0.4586	H
81	C81	4.6425	-3.6529	-1.0331	C
82	C82	4.6669	-4.5351	-2.1253	C
83	H83	3.8443	-4.5237	-2.8359	H
84	C84	5.7377	-5.4005	-2.3225	C
85	H85	5.7398	-6.0664	-3.1814	H
86	C86	6.8061	-5.4104	-1.4271	C
87	H87	7.6422	-6.0872	-1.5796	H
88	C88	6.7905	-4.5497	-0.3310	C
89	H89	7.6105	-4.5604	0.3821	H
90	C90	5.7205	-3.6818	-0.1340	C
91	H91	5.7033	-3.0386	0.7416	H
92	B92	-3.6646	2.7932	1.4396	B
93	H93	-3.1410	3.8275	1.1649	H
94	B94	-4.8756	2.0082	0.4100	B
95	H95	-5.2519	2.5822	-0.5692	H
96	B96	-2.6068	1.5061	2.0454	B
97	H97	-1.4195	1.6051	2.1232	H
98	B98	-3.5787	0.4589	3.0754	B

99 H99 -3.0447 -0.1851 3.9199 H
100 B100 -4.7772 -0.3383 1.9989 B
101 H101 -5.0567 -1.4762 2.2297 H
102 B102 -3.8670 2.1747 3.0846 B
103 H103 -3.5927 2.8380 4.0383 H
104 B104 -5.2790 2.4945 2.0354 B
105 H105 -6.0643 3.3689 2.2580 H
106 B106 -5.9436 0.9680 1.4357 B
107 H107 -7.1257 0.8187 1.3302 H
108 H108 -5.3804 0.0283 0.7933 H
109 B109 -5.2429 1.0123 3.0643 B
110 H110 -5.9289 0.8767 4.0344 H
@<TRIPOS>BOND
1 2 7 1
2 2 8 1
3 2 19 1
4 3 6 1
5 3 30 1
6 3 41 1
7 4 52 Ar
8 4 53 Ar
9 5 67 Ar
10 5 69 Ar
11 6 7 1
12 6 92 1
13 6 94 1
14 6 96 1
15 7 96 1
16 7 98 1
17 7 100 1
18 8 9 Ar
19 8 17 Ar
20 9 10 1
21 9 11 Ar
22 11 12 1
23 11 13 Ar
24 13 14 1
25 13 15 Ar
26 15 16 1
27 15 17 Ar
28 17 18 1
29 19 20 Ar
30 19 28 Ar
31 20 21 1
32 20 22 Ar
33 22 23 1
34 22 24 Ar
35 24 25 1
36 24 26 Ar
37 26 27 1
38 26 28 Ar
39 28 29 1
40 30 31 Ar
41 30 39 Ar
42 31 32 1
43 31 33 Ar
44 33 34 1
45 33 35 Ar
46 35 36 1
47 35 37 Ar

48 37 38 1
49 37 39 Ar
50 39 40 1
51 41 42 Ar
52 41 50 Ar
53 42 43 1
54 42 44 Ar
55 44 45 1
56 44 46 Ar
57 46 47 1
58 46 48 Ar
59 48 49 1
60 48 50 Ar
61 50 51 1
62 52 58 Ar
63 52 69 Ar
64 53 54 1
65 53 55 2
66 55 56 1
67 55 57 Ar
68 57 58 Ar
69 57 70 1
70 58 59 Ar
71 59 60 1
72 59 61 2
73 61 62 1
74 61 63 Ar
75 63 64 Ar
76 63 69 Ar
77 64 65 Ar
78 64 81 1
79 65 66 1
80 65 67 2
81 67 68 1
82 70 71 Ar
83 70 79 Ar
84 71 72 1
85 71 73 Ar
86 73 74 1
87 73 75 Ar
88 75 76 1
89 75 77 Ar
90 77 78 1
91 77 79 Ar
92 79 80 1
93 81 82 Ar
94 81 90 Ar
95 82 83 1
96 82 84 Ar
97 84 85 1
98 84 86 Ar
99 86 87 1
100 86 88 Ar
101 88 89 1
102 88 90 Ar
103 90 91 1
104 92 93 1
105 92 94 1
106 92 96 1
107 92 102 1

108 92 104 1
109 94 95 1
110 94 104 1
111 94 106 1
112 96 97 1
113 96 98 1
114 96 102 1
115 98 99 1
116 98 100 1
117 98 102 1
118 98 109 1
119 100 101 1
120 100 106 1
121 100 109 1
122 102 103 1
123 102 104 1
124 102 109 1
125 104 105 1
126 104 106 1
127 104 109 1
128 106 107 1
129 106 109 1
130 109 110 1

Cu-5 S_0
@<TRIPOS>MOLECULE
Molecule Name
94 112
SMALL
NO_CHARGES

@<TRIPOS>ATOM
1 Cu1 0.2949 -0.9795 -0.0761 Cu
2 P2 1.3143 1.1389 -0.3028 P
3 P3 -1.7902 0.1056 0.0354 P
4 N4 0.7262 -2.7550 -1.2090 N
5 N5 1.0359 -2.2856 1.4577 N
6 C6 -1.5549 1.7983 -0.6936 C
7 C7 -0.0344 2.3011 -0.8317 C
8 C8 2.7223 1.3276 -1.4576 C
9 C9 3.1085 0.2179 -2.2148 C
10 H10 2.5656 -0.7175 -2.1042 H
11 C11 4.1957 0.2965 -3.0849 C
12 H12 4.4852 -0.5729 -3.6688 H
13 C13 4.9115 1.4847 -3.1936 C
14 H14 5.7609 1.5488 -3.8682 H
15 C15 4.5445 2.5927 -2.4279 C
16 H16 5.1069 3.5191 -2.5063 H
17 C17 3.4584 2.5161 -1.5635 C
18 H18 3.1793 3.3807 -0.9677 H
19 C19 2.0162 1.8237 1.2484 C
20 C20 3.3172 1.4475 1.6140 C
21 H21 3.9231 0.8532 0.9349 H
22 C22 3.8502 1.8362 2.8394 C
23 H23 4.8630 1.5416 3.1011 H
24 C24 3.0925 2.6030 3.7239 C
25 H25 3.5101 2.9085 4.6793 H
26 C26 1.7978 2.9759 3.3722 C
27 H27 1.1972 3.5746 4.0517 H

28	C28	1.2642	2.5860	2.1461	C
29	H29	0.2528	2.8808	1.8932	H
30	C30	-3.2575	-0.6873	-0.7251	C
31	C31	-3.1011	-1.9745	-1.2491	C
32	H32	-2.1223	-2.4468	-1.2240	H
33	C33	-4.1928	-2.6654	-1.7751	C
34	H34	-4.0561	-3.6649	-2.1787	H
35	C35	-5.4521	-2.0744	-1.7740	C
36	H36	-6.3053	-2.6089	-2.1827	H
37	C37	-5.6212	-0.7972	-1.2363	C
38	H38	-6.6059	-0.3378	-1.2250	H
39	C39	-4.5344	-0.1090	-0.7093	C
40	H40	-4.6729	0.8816	-0.2871	H
41	C41	-2.3858	0.3562	1.7553	C
42	C42	-2.8788	-0.7730	2.4265	C
43	H43	-2.9749	-1.7192	1.8985	H
44	C44	-3.2539	-0.6971	3.7632	C
45	H45	-3.6417	-1.5802	4.2640	H
46	C46	-3.1326	0.5069	4.4578	C
47	H47	-3.4229	0.5666	5.5031	H
48	C48	-2.6443	1.6320	3.8004	C
49	H49	-2.5564	2.5779	4.3282	H
50	C50	-2.2754	1.5584	2.4575	C
51	H51	-1.9273	2.4513	1.9503	H
52	C52	1.1825	-3.7664	-0.4426	C
53	C53	0.4632	-2.9839	-2.5102	C
54	C54	0.6280	-4.2488	-3.0773	C
55	H55	0.4053	-4.3955	-4.1289	H
56	C56	1.0639	-5.2999	-2.2850	C
57	C57	1.3466	-5.0570	-0.9492	C
58	C58	2.2326	-4.3393	1.7626	C
59	C59	2.4719	-4.0144	3.0923	C
60	C60	1.9659	-2.8269	3.5919	C
61	H61	2.1214	-2.5419	4.6277	H
62	C62	1.2509	-1.9729	2.7451	C
63	C63	1.4948	-3.4577	0.9743	C
64	B64	-2.5062	2.2727	-2.0641	B
65	H65	-3.3313	1.5530	-2.5338	H
66	B66	-2.6396	2.9862	-0.4583	B
67	H67	-3.6387	2.7936	0.1734	H
68	B68	-0.8171	1.7796	-2.2496	B
69	H69	-0.4764	0.7715	-2.7736	H
70	B70	0.1632	3.2383	-2.2538	B
71	H71	1.2133	3.2398	-2.8112	H
72	B72	0.0336	3.9305	-0.6097	B
73	H73	1.0004	4.4580	-0.1456	H
74	B74	-1.3996	3.2668	-3.0147	B
75	H75	-1.4987	3.3539	-4.2025	H
76	B76	-2.5531	4.0045	-1.8719	B
77	H77	-3.4757	4.6796	-2.2288	H
78	B78	-1.6999	4.5316	-0.4153	B
79	H79	-2.0002	5.5519	0.1352	H
80	H80	-0.9518	3.8763	0.3733	H
81	B81	-0.8655	4.6327	-1.9763	B
82	H82	-0.5934	5.6913	-2.4648	H
83	C83	-0.0075	-1.8391	-3.3472	C
84	H84	-0.9298	-1.4101	-2.9443	H
85	H85	0.7382	-1.0381	-3.3656	H
86	H86	-0.1958	-2.1572	-4.3755	H
87	C87	0.7012	-0.6886	3.2759	C

88 H88 1.4989 -0.0664 3.6957 H
89 H89 0.1991 -0.1257 2.4897 H
90 H90 -0.0252 -0.8828 4.0735 H
91 H91 3.0469 -4.6839 3.7249 H
92 H92 2.6342 -5.2569 1.3509 H
93 H93 1.6712 -5.8684 -0.3100 H
94 H94 1.1812 -6.2967 -2.6991 H
@<TRIPOS>BOND
1 1 3 1
2 2 7 1
3 2 8 1
4 2 19 1
5 3 6 1
6 3 30 1
7 3 41 1
8 4 52 Ar
9 4 53 Ar
10 5 62 Ar
11 5 63 Ar
12 6 7 1
13 6 64 1
14 6 66 1
15 6 68 1
16 7 68 1
17 7 70 1
18 7 72 1
19 8 9 Ar
20 8 17 Ar
21 9 10 1
22 9 11 Ar
23 11 12 1
24 11 13 Ar
25 13 14 1
26 13 15 Ar
27 15 16 1
28 15 17 Ar
29 17 18 1
30 19 20 Ar
31 19 28 Ar
32 20 21 1
33 20 22 Ar
34 22 23 1
35 22 24 Ar
36 24 25 1
37 24 26 Ar
38 26 27 1
39 26 28 Ar
40 28 29 1
41 30 31 Ar
42 30 39 Ar
43 31 32 1
44 31 33 Ar
45 33 34 1
46 33 35 Ar
47 35 36 1
48 35 37 Ar
49 37 38 1
50 37 39 Ar
51 39 40 1
52 41 42 Ar

53 41 50 Ar
54 42 43 1
55 42 44 Ar
56 44 45 1
57 44 46 Ar
58 46 47 1
59 46 48 Ar
60 48 49 1
61 48 50 Ar
62 50 51 1
63 52 57 Ar
64 52 63 1
65 53 54 Ar
66 53 83 1
67 54 55 1
68 54 56 Ar
69 56 57 Ar
70 56 94 1
71 57 93 1
72 58 59 Ar
73 58 63 Ar
74 58 92 1
75 59 60 2
76 59 91 1
77 60 61 1
78 60 62 Ar
79 62 87 1
80 64 65 1
81 64 66 1
82 64 68 1
83 64 74 1
84 64 76 1
85 66 67 1
86 66 76 1
87 66 78 1
88 68 69 1
89 68 70 1
90 68 74 1
91 70 71 1
92 70 72 1
93 70 74 1
94 70 81 1
95 72 73 1
96 72 78 1
97 72 81 1
98 74 75 1
99 74 76 1
100 74 81 1
101 76 77 1
102 76 78 1
103 76 81 1
104 78 79 1
105 78 81 1
106 81 82 1
107 83 84 1
108 83 85 1
109 83 86 1
110 87 88 1
111 87 89 1
112 87 90 1

Cu-5 S_1

```
@<TRIPOS>MOLECULE
Molecule Name
94 111
SMALL
NO_CHARGES


@<TRIPOS>ATOM
1 Cu1    -0.8579    -0.5805    -0.0595 Cu
2 P2     1.5716    -0.8053    -0.3634 P
3 P3    -0.3274     1.7219     0.1818 P
4 N4    -2.5363    -0.9636    -1.2462 N
5 N5    -1.7473    -1.9638     1.1923 N
6 C6     1.3384     2.1210    -0.4613 C
7 C7     2.2727     0.8650    -0.7296 C
8 C8     1.7821    -1.9009    -1.8066 C
9 C9     0.6458    -2.3896    -2.4605 C
10 H10    -0.3482    -2.1215    -2.1088 H
11 C11     0.7753    -3.2404    -3.5567 C
12 H12    -0.1141    -3.6138    -4.0558 H
13 C13     2.0404    -3.6122    -4.0019 C
14 H14     2.1428    -4.2752    -4.8563 H
15 C15     3.1780    -3.1421    -3.3454 C
16 H16     4.1661    -3.4382    -3.6855 H
17 C17     3.0534    -2.2937    -2.2511 C
18 H18     3.9439    -1.9429    -1.7378 H
19 C19     2.6164    -1.5599     0.9226 C
20 C20     2.8506    -2.9425     0.9086 C
21 H21     2.4837    -3.5524     0.0892 H
22 C22     3.5589    -3.5461     1.9444 C
23 H23     3.7402    -4.6166     1.9149 H
24 C24     4.0314    -2.7834     3.0094 C
25 H25     4.5844    -3.2560     3.8161 H
26 C26     3.7880    -1.4110     3.0383 C
27 H27     4.1495    -0.8087     3.8667 H
28 C28     3.0814    -0.8046     2.0063 C
29 H29     2.8917     0.2620     2.0517 H
30 C30    -1.5872     2.9652    -0.2567 C
31 C31    -2.8582     2.4946    -0.6082 C
32 H32    -3.0390     1.4259    -0.6913 H
33 C33    -3.8968     3.3936    -0.8442 C
34 H34    -4.8789     3.0190    -1.1180 H
35 C35    -3.6721     4.7619    -0.7310 C
36 H36    -4.4801     5.4635    -0.9187 H
37 C37    -2.4101     5.2337    -0.3694 C
38 H38    -2.2340     6.3012    -0.2725 H
39 C39    -1.3704     4.3437    -0.1245 C
40 H40    -0.3956     4.7195     0.1665 H
41 C41    -0.2844     1.7843     2.0035 C
42 C42    -1.4827     1.4802     2.6702 C
43 H43    -2.3860     1.2680     2.1030 H
44 C44    -1.5253     1.4551     4.0590 C
45 H45    -2.4580     1.2219     4.5641 H
46 C46    -0.3739     1.7258     4.7980 C
47 H47    -0.4072     1.7049     5.8835 H
48 C48     0.8169     2.0304     4.1427 C
49 H49     1.7126     2.2559     4.7143 H
50 C50     0.8658     2.0593     2.7508 C
```

51 H51 1.7886 2.3317 2.2491 H
52 C52 -3.3391 -1.9000 -0.6187 C
53 C53 -2.8004 -0.6144 -2.5275 C
54 C54 -3.8878 -1.1161 -3.2224 C
55 H55 -4.0741 -0.7864 -4.2384 H
56 C56 -4.7262 -2.0608 -2.5936 C
57 C57 -4.4451 -2.4549 -1.3111 C
58 C58 -3.9124 -2.9874 1.5506 C
59 C59 -3.5500 -3.3356 2.8269 C
60 C60 -2.2703 -2.9817 3.2991 C
61 H61 -1.9491 -3.2378 4.3027 H
62 C62 -1.4111 -2.2914 2.4590 C
63 C63 -3.0015 -2.2933 0.7153 C
64 B64 1.5101 3.3345 -1.6796 B
65 H65 0.5709 3.9408 -2.0899 H
66 B66 2.1135 3.4829 -0.0239 B
67 H67 1.5992 4.2716 0.7135 H
68 B68 1.5768 1.6087 -2.0922 B
69 H69 0.7329 1.0442 -2.7036 H
70 B70 3.2677 1.1315 -2.1034 B
71 H71 3.6043 0.2043 -2.7653 H
72 B72 3.8442 1.2621 -0.4124 B
73 H73 4.6299 0.4482 -0.0350 H
74 B74 2.8236 2.7114 -2.6842 B
75 H75 2.9040 2.9805 -3.8443 H
76 B76 3.1389 3.8837 -1.3747 B
77 H77 3.5026 5.0041 -1.5824 H
78 B78 3.8709 3.0558 0.0089 B
79 H79 4.7314 3.5828 0.6511 H
80 H80 3.4676 2.0481 0.6713 H
81 B81 4.2618 2.4963 -1.6269 B
82 H82 5.3636 2.6212 -2.0744 H
83 C83 -1.8632 0.3333 -3.2100 C
84 H84 -1.6102 1.1855 -2.5750 H
85 H85 -0.9262 -0.1728 -3.4693 H
86 H86 -2.3034 0.7137 -4.1355 H
87 C87 -0.0448 -1.9191 2.9389 C
88 H88 0.7136 -2.5663 2.4866 H
89 H89 0.2004 -0.8867 2.6732 H
90 H90 0.0269 -2.0216 4.0248 H
91 H91 -4.2504 -3.8578 3.4726 H
92 H92 -4.9125 -3.2054 1.1936 H
93 H93 -5.0475 -3.2210 -0.8363 H
94 H94 -5.5701 -2.4888 -3.1272 H
@<TRIPOS>BOND
1 2 7 1
2 2 8 1
3 2 19 1
4 3 6 1
5 3 30 1
6 3 41 1
7 4 52 1
8 4 53 Ar
9 5 62 Ar
10 5 63 Ar
11 6 7 1
12 6 64 1
13 6 66 1
14 6 68 1
15 7 68 1

16 7 70 1
17 7 72 1
18 8 9 Ar
19 8 17 Ar
20 9 10 1
21 9 11 Ar
22 11 12 1
23 11 13 Ar
24 13 14 1
25 13 15 Ar
26 15 16 1
27 15 17 Ar
28 17 18 1
29 19 20 Ar
30 19 28 Ar
31 20 21 1
32 20 22 Ar
33 22 23 1
34 22 24 Ar
35 24 25 1
36 24 26 Ar
37 26 27 1
38 26 28 Ar
39 28 29 1
40 30 31 Ar
41 30 39 Ar
42 31 32 1
43 31 33 Ar
44 33 34 1
45 33 35 Ar
46 35 36 1
47 35 37 Ar
48 37 38 1
49 37 39 Ar
50 39 40 1
51 41 42 Ar
52 41 50 Ar
53 42 43 1
54 42 44 Ar
55 44 45 1
56 44 46 Ar
57 46 47 1
58 46 48 Ar
59 48 49 1
60 48 50 Ar
61 50 51 1
62 52 57 Ar
63 52 63 Ar
64 53 54 2
65 53 83 1
66 54 55 1
67 54 56 Ar
68 56 57 2
69 56 94 1
70 57 93 1
71 58 59 2
72 58 63 Ar
73 58 92 1
74 59 60 Ar
75 59 91 1

76 60 61 1
77 60 62 2
78 62 87 1
79 64 65 1
80 64 66 1
81 64 68 1
82 64 74 1
83 64 76 1
84 66 67 1
85 66 76 1
86 66 78 1
87 68 69 1
88 68 70 1
89 68 74 1
90 70 71 1
91 70 72 1
92 70 74 1
93 70 81 1
94 72 73 1
95 72 78 1
96 72 81 1
97 74 75 1
98 74 76 1
99 74 81 1
100 76 77 1
101 76 78 1
102 76 81 1
103 78 79 1
104 78 81 1
105 81 82 1
106 83 84 1
107 83 85 1
108 83 86 1
109 87 88 1
110 87 89 1
111 87 90 1

Cu-5 T_1
@<TRIPOS>MOLECULE
Molecule Name
94 111
SMALL
NO_CHARGES

@<TRIPOS>ATOM
1 Cu1 -0.8231 0.6213 0.0769 Cu
2 P2 1.5922 0.7488 0.3869 P
3 P3 -0.3962 -1.6934 -0.2000 P
4 N4 -2.4560 1.0852 1.2436 N
5 N5 -1.6740 1.9999 -1.1894 N
6 C6 1.2471 -2.1645 0.4555 C
7 C7 2.2275 -0.9486 0.7431 C
8 C8 1.8577 1.8413 1.8229 C
9 C9 0.7510 2.4038 2.4678 C
10 H10 -0.2563 2.1909 2.1159 H
11 C11 0.9303 3.2580 3.5545 C
12 H12 0.0641 3.6895 4.0476 H
13 C13 2.2145 3.5592 3.9978 C
14 H14 2.3551 4.2247 4.8448 H

15	C15	3.3233	3.0160	3.3483	C
16	H16	4.3269	3.2579	3.6859	H
17	C17	3.1500	2.1644	2.2632	C
18	H18	4.0188	1.7583	1.7538	H
19	C19	2.6556	1.4620	-0.9093	C
20	C20	2.9344	2.8358	-0.8992	C
21	H21	2.5878	3.4599	-0.0817	H
22	C22	3.6602	3.4130	-1.9381	C
23	H23	3.8755	4.4773	-1.9130	H
24	C24	4.1067	2.6314	-3.0006	C
25	H25	4.6734	3.0831	-3.8097	H
26	C26	3.8207	1.2674	-3.0239	C
27	H27	4.1622	0.6508	-3.8502	H
28	C28	3.0960	0.6875	-1.9890	C
29	H29	2.8733	-0.3727	-2.0305	H
30	C30	-1.7093	-2.8773	0.2455	C
31	C31	-2.9541	-2.3615	0.6246	C
32	H32	-3.0921	-1.2879	0.7196	H
33	C33	-4.0225	-3.2221	0.8704	C
34	H34	-4.9839	-2.8123	1.1662	H
35	C35	-3.8537	-4.5968	0.7390	C
36	H36	-4.6849	-5.2683	0.9347	H
37	C37	-2.6182	-5.1133	0.3481	C
38	H38	-2.4859	-6.1857	0.2365	H
39	C39	-1.5495	-4.2616	0.0930	C
40	H40	-0.5956	-4.6716	-0.2206	H
41	C41	-0.3514	-1.7808	-2.0215	C
42	C42	-1.5400	-1.4531	-2.6932	C
43	H43	-2.4355	-1.2016	-2.1299	H
44	C44	-1.5820	-1.4522	-4.0821	C
45	H45	-2.5075	-1.2010	-4.5918	H
46	C46	-0.4393	-1.7710	-4.8155	C
47	H47	-0.4724	-1.7693	-5.9012	H
48	C48	0.7418	-2.0985	-4.1546	C
49	H49	1.6306	-2.3603	-4.7215	H
50	C50	0.7897	-2.1039	-2.7621	C
51	H51	1.7042	-2.3935	-2.2555	H
52	C52	-3.2774	2.0008	0.6024	C
53	C53	-2.7734	0.6839	2.5067	C
54	C54	-3.9036	1.1255	3.1600	C
55	H55	-4.1115	0.7768	4.1657	H
56	C56	-4.7638	2.0434	2.5124	C
57	C57	-4.4430	2.4773	1.2540	C
58	C58	-3.7082	3.2801	-1.4980	C
59	C59	-3.3082	3.6422	-2.7576	C
60	C60	-2.0754	3.1637	-3.2491	C
61	H61	-1.7306	3.4182	-4.2454	H
62	C62	-1.2951	2.3584	-2.4418	C
63	C63	-2.8892	2.4424	-0.6997	C
64	B64	1.3683	-3.3994	1.6603	B
65	H65	0.4051	-3.9750	2.0585	H
66	B66	1.9732	-3.5509	0.0065	B
67	H67	1.4336	-4.3138	-0.7401	H
68	B68	1.4974	-1.6827	2.0938	B
69	H69	0.6748	-1.0958	2.7108	H
70	B70	3.2046	-1.2683	2.1184	B
71	H71	3.5714	-0.3627	2.7938	H
72	B72	3.7834	-1.3995	0.4282	B
73	H73	4.6002	-0.6107	0.0637	H
74	B74	2.6997	-2.8374	2.6776	B

```
75 H75     2.7640    -3.1219     3.8350 H
76 B76     2.9775    -4.0043     1.3559 B
77 H77     3.2976    -5.1400     1.5513 H
78 B78     3.7459    -3.1884    -0.0146 B
79 H79     4.5890    -3.7392    -0.6597 H
80 H80     3.3838    -2.1586    -0.6662 H
81 B81     4.1499    -2.6626     1.6294 B
82 H82     5.2441    -2.8334     2.0801 H
83 C83    -1.8281    -0.2437     3.2025 C
84 H84    -1.6243    -1.1381     2.6075 H
85 H85    -0.8684     0.2505     3.3892 H
86 H86    -2.2358    -0.5628     4.1652 H
87 C87     0.0290     1.8744    -2.9384 C
88 H88     0.8418     2.4815    -2.5267 H
89 H89     0.2077     0.8362    -2.6458 H
90 H90     0.0814     1.9394    -4.0285 H
91 H91    -3.9381     4.2784    -3.3727 H
92 H92    -4.6666     3.6183    -1.1224 H
93 H93    -5.0788     3.2021     0.7594 H
94 H94    -5.6569     2.4085     3.0107 H
@<TRIPOS>BOND
1 2 7 1
2 2 8 1
3 2 19 1
4 3 6 1
5 3 30 1
6 3 41 1
7 4 52 1
8 4 53 Ar
9 5 62 Ar
10 5 63 1
11 6 7 1
12 6 64 1
13 6 66 1
14 6 68 1
15 7 68 1
16 7 70 1
17 7 72 1
18 8 9 Ar
19 8 17 Ar
20 9 10 1
21 9 11 Ar
22 11 12 1
23 11 13 Ar
24 13 14 1
25 13 15 Ar
26 15 16 1
27 15 17 Ar
28 17 18 1
29 19 20 Ar
30 19 28 Ar
31 20 21 1
32 20 22 Ar
33 22 23 1
34 22 24 Ar
35 24 25 1
36 24 26 Ar
37 26 27 1
38 26 28 Ar
39 28 29 1
```

40	30	31	Ar
41	30	39	Ar
42	31	32	1
43	31	33	Ar
44	33	34	1
45	33	35	Ar
46	35	36	1
47	35	37	Ar
48	37	38	1
49	37	39	Ar
50	39	40	1
51	41	42	Ar
52	41	50	Ar
53	42	43	1
54	42	44	Ar
55	44	45	1
56	44	46	Ar
57	46	47	1
58	46	48	Ar
59	48	49	1
60	48	50	Ar
61	50	51	1
62	52	57	Ar
63	52	63	Ar
64	53	54	2
65	53	83	1
66	54	55	1
67	54	56	Ar
68	56	57	2
69	56	94	1
70	57	93	1
71	58	59	2
72	58	63	Ar
73	58	92	1
74	59	60	Ar
75	59	91	1
76	60	61	1
77	60	62	2
78	62	87	1
79	64	65	1
80	64	66	1
81	64	68	1
82	64	74	1
83	64	76	1
84	66	67	1
85	66	76	1
86	66	78	1
87	68	69	1
88	68	70	1
89	68	74	1
90	70	71	1
91	70	72	1
92	70	74	1
93	70	81	1
94	72	73	1
95	72	78	1
96	72	81	1
97	74	75	1
98	74	76	1
99	74	81	1

100 76 77 1
101 76 78 1
102 76 81 1
103 78 79 1
104 78 81 1
105 81 82 1
106 83 84 1
107 83 85 1
108 83 86 1
109 87 88 1
110 87 89 1
111 87 90 1

Zn-1 S_0

@<TRIPOS>MOLECULE
Molecule Name
149 172
SMALL
NO_CHARGES

@<TRIPOS>ATOM
1 Zn1 0.5622 0.9601 -1.6496 Zn
2 Zn2 0.9208 -1.5788 0.3123 Zn
3 Zn3 -0.1304 1.2495 1.5172 Zn
4 Zn4 -2.0270 -0.5144 -0.4166 Zn
5 O5 -0.1668 0.0263 -0.0497 O
6 N6 1.9405 -0.2427 -2.5762 N
7 N7 2.7689 -1.4756 -0.6977 N
8 C8 2.4340 -0.0371 -3.8438 C
9 C9 3.6656 -0.6182 -4.0440 C
10 C10 3.9975 -1.2466 -2.8104 C
11 C11 5.0662 -2.0061 -2.3085 C
12 C12 4.9443 -2.4636 -0.9898 C
13 C13 3.8088 -2.1874 -0.2357 C
14 C14 2.8788 -0.9941 -1.9497 C
15 N15 0.3836 3.1377 0.9318 N
16 N16 1.6642 2.6630 -1.0377 N
17 C17 0.2625 4.2642 1.7126 C
18 C18 1.0751 5.2953 1.3007 C
19 C19 1.7774 4.7986 0.1664 C
20 C20 2.7321 5.2949 -0.7350 C
21 C21 3.1304 4.4306 -1.7622 C
22 C22 2.5855 3.1561 -1.8791 C
23 C23 1.2899 3.4639 -0.0229 C
24 N24 -0.9719 1.5932 -2.8431 N
25 N25 -2.9588 0.8205 -1.7303 N
26 C26 -0.7800 2.4229 -3.9236 C
27 C27 -1.9553 2.8709 -4.4772 C
28 C28 -2.9900 2.2918 -3.6923 C
29 C29 -4.3912 2.3522 -3.6683 C
30 C30 -5.0304 1.6499 -2.6390 C
31 C31 -4.2983 0.9141 -1.7157 C
32 C32 -2.3163 1.5128 -2.6914 C
33 N33 1.3431 -1.7254 2.2971 N
34 N34 1.4021 0.6083 2.8472 N
35 C35 1.8050 -2.8467 2.9475 C
36 C36 2.4620 -2.5550 4.1203 C
37 C37 2.4266 -1.1362 4.2371 C
38 C38 2.8858 -0.1811 5.1581 C

39	C39	2.5891	1.1565	4.8703	C
40	C40	1.8620	1.5019	3.7356	C
41	C41	1.7027	-0.6815	3.0872	C
42	N42	-2.0655	-2.4046	-1.1726	N
43	N43	0.0398	-3.3266	-0.4649	N
44	C44	-3.1236	-2.9369	-1.8726	C
45	C45	-2.9491	-4.2641	-2.1841	C
46	C46	-1.6785	-4.6169	-1.6507	C
47	C47	-0.8785	-5.7689	-1.6341	C
48	C48	0.3788	-5.6465	-1.0308	C
49	C49	0.7899	-4.4404	-0.4771	C
50	C50	-1.1736	-3.4170	-1.0445	C
51	N51	-1.8985	1.1444	2.5455	N
52	N52	-3.1726	-0.4973	1.3400	N
53	C53	-2.1067	1.7749	3.7502	C
54	C54	-3.2809	1.4032	4.3607	C
55	C55	-3.8780	0.4527	3.4879	C
56	C56	-5.0301	-0.3471	3.5028	C
57	C57	-5.1921	-1.2321	2.4292	C
58	C58	-4.2660	-1.2759	1.3941	C
59	C59	-2.9710	0.3332	2.3809	C
60	H60	1.8433	0.5222	-4.5613	H
61	H61	4.2380	-0.6180	-4.9609	H
62	H62	5.7547	-3.0216	-0.5316	H
63	H63	3.7256	-2.5415	0.7893	H
64	H64	-0.4398	4.2677	2.5390	H
65	H65	1.1357	6.2834	1.7345	H
66	H66	3.8933	4.7412	-2.4690	H
67	H67	2.9074	2.4904	-2.6766	H
68	H68	0.2287	2.6678	-4.2379	H
69	H69	-2.0600	3.5471	-5.3136	H
70	H70	-6.1135	1.6454	-2.5724	H
71	H71	1.6135	-3.8266	2.5241	H
72	H72	2.8846	-3.2648	4.8173	H
73	H73	2.9492	1.9457	5.5223	H
74	H74	1.6447	2.5450	3.5179	H
75	H75	-1.3651	2.4711	4.1263	H
76	H76	-3.6478	1.7427	5.3188	H
77	H77	-6.0668	-1.8723	2.3774	H
78	H78	-4.3999	-1.9672	0.5656	H
79	H79	1.7750	-4.3604	-0.0246	H
80	H80	1.0403	-6.5045	-0.9682	H
81	H81	-3.9639	-2.3026	-2.1341	H
82	H82	-3.6249	-4.8901	-2.7491	H
83	H83	-4.8066	0.3708	-0.9231	H
84	C84	6.2718	-2.3122	-3.1042	C
85	C85	6.8662	-3.5792	-3.0368	C
86	C86	6.8594	-1.3403	-3.9250	C
87	C87	8.0102	-3.8686	-3.7722	C
88	H88	6.4089	-4.3490	-2.4206	H
89	C89	8.0076	-1.6284	-4.6549	C
90	H90	6.4239	-0.3462	-3.9657	H
91	C91	8.5857	-2.8936	-4.5836	C
92	H92	8.4507	-4.8605	-3.7157	H
93	H93	8.4561	-0.8590	-5.2777	H
94	H94	9.4805	-3.1188	-5.1576	H
95	C95	-5.1734	3.1144	-4.6619	C
96	C96	-6.2864	3.8675	-4.2659	C
97	C97	-4.8324	3.0866	-6.0207	C
98	C98	-7.0336	4.5768	-5.1998	C

99 H99 -6.5486 3.9163 -3.2122 H
100 C100 -5.5847 3.7904 -6.9549 C
101 H101 -3.9861 2.4869 -6.3431 H
102 C102 -6.6860 4.5401 -6.5481 C
103 H103 -7.8867 5.1651 -4.8725 H
104 H104 -5.3121 3.7490 -8.0062 H
105 H105 -7.2703 5.0935 -7.2783 H
106 C106 -1.3155 -7.0518 -2.2198 C
107 C107 -0.4204 -7.8398 -2.9551 C
108 C108 -2.6249 -7.5165 -2.0390 C
109 C109 -0.8240 -9.0541 -3.4993 C
110 H110 0.5915 -7.4801 -3.1227 H
111 C111 -3.0253 -8.7347 -2.5772 C
112 H112 -3.3214 -6.9261 -1.4506 H
113 C113 -2.1279 -9.5066 -3.3115 C
114 H114 -0.1193 -9.6460 -4.0773 H
115 H115 -4.0418 -9.0848 -2.4183 H
116 H116 -2.4432 -10.4559 -3.7359 H
117 C117 -6.0318 -0.2824 4.5856 C
118 C118 -6.6173 -1.4528 5.0857 C
119 C119 -6.4337 0.9490 5.1201 C
120 C120 -7.5732 -1.3948 6.0938 C
121 H121 -6.2978 -2.4161 4.6965 H
122 C122 -7.3953 1.0064 6.1231 C
123 H123 -6.0040 1.8647 4.7242 H
124 C124 -7.9668 -0.1647 6.6153 C
125 H125 -8.0080 -2.3140 6.4775 H
126 H126 -7.7031 1.9708 6.5186 H
127 H127 -8.7149 -0.1190 7.4022 H
128 C128 3.6530 -0.5418 6.3671 C
129 C129 3.4078 0.1053 7.5853 C
130 C130 4.6542 -1.5210 6.3190 C
131 C131 4.1373 -0.2207 8.7234 C
132 H132 2.6195 0.8515 7.6416 H
133 C133 5.3880 -1.8416 7.4560 C
134 H134 4.8718 -2.0117 5.3748 H
135 C135 5.1308 -1.1951 8.6627 C
136 H136 3.9250 0.2838 9.6622 H
137 H137 6.1682 -2.5960 7.3979 H
138 H138 5.7017 -1.4494 9.5516 H
139 C139 3.3089 6.6496 -0.6224 C
140 C140 3.5236 7.4285 -1.7670 C
141 C141 3.6730 7.1766 0.6238 C
142 C142 4.0809 8.6987 -1.6688 C
143 H143 3.2255 7.0406 -2.7376 H
144 C144 4.2372 8.4441 0.7204 C
145 H145 3.5346 6.5727 1.5159 H
146 C146 4.4408 9.2108 -0.4245 C
147 H147 4.2294 9.2928 -2.5666 H
148 H148 4.5239 8.8325 1.6942 H
149 H149 4.8775 10.2029 -0.3474 H
@<TRIPOS>BOND
1 1 5 1
2 1 6 1
3 1 16 1
4 1 24 1
5 2 5 1
6 2 7 1
7 2 33 1
8 2 43 1

9 3 5 1
10 3 15 1
11 3 34 1
12 3 51 1
13 4 5 1
14 4 25 1
15 4 42 1
16 4 52 1
17 6 8 1
18 6 14 Ar
19 7 13 Ar
20 7 14 Ar
21 8 9 Ar
22 8 60 1
23 9 10 Ar
24 9 61 1
25 10 11 Ar
26 10 14 1
27 11 12 Ar
28 11 84 1
29 12 13 Ar
30 12 62 1
31 13 63 1
32 15 17 1
33 15 23 Ar
34 16 22 Ar
35 16 23 Ar
36 17 18 Ar
37 17 64 1
38 18 19 Ar
39 18 65 1
40 19 20 Ar
41 19 23 1
42 20 21 Ar
43 20 139 1
44 21 22 Ar
45 21 66 1
46 22 67 1
47 24 26 1
48 24 32 Ar
49 25 31 Ar
50 25 32 Ar
51 26 27 Ar
52 26 68 1
53 27 28 Ar
54 27 69 1
55 28 29 Ar
56 28 32 1
57 29 30 Ar
58 29 95 1
59 30 31 Ar
60 30 70 1
61 31 83 1
62 33 35 1
63 33 41 Ar
64 34 40 Ar
65 34 41 Ar
66 35 36 Ar
67 35 71 1
68 36 37 Ar

69 36 72 1
70 37 38 Ar
71 37 41 1
72 38 39 Ar
73 38 128 1
74 39 40 Ar
75 39 73 1
76 40 74 1
77 42 44 1
78 42 50 Ar
79 43 49 Ar
80 43 50 Ar
81 44 45 Ar
82 44 81 1
83 45 46 Ar
84 45 82 1
85 46 47 Ar
86 46 50 1
87 47 48 Ar
88 47 106 1
89 48 49 Ar
90 48 80 1
91 49 79 1
92 51 53 1
93 51 59 Ar
94 52 58 Ar
95 52 59 Ar
96 53 54 Ar
97 53 75 1
98 54 55 Ar
99 54 76 1
100 55 56 Ar
101 55 59 1
102 56 57 Ar
103 56 117 1
104 57 58 Ar
105 57 77 1
106 58 78 1
107 84 85 Ar
108 84 86 Ar
109 85 87 Ar
110 85 88 1
111 86 89 Ar
112 86 90 1
113 87 91 Ar
114 87 92 1
115 89 91 Ar
116 89 93 1
117 91 94 1
118 95 96 Ar
119 95 97 Ar
120 96 98 Ar
121 96 99 1
122 97 100 Ar
123 97 101 1
124 98 102 Ar
125 98 103 1
126 100 102 Ar
127 100 104 1
128 102 105 1

129 106 107 Ar
130 106 108 Ar
131 107 109 Ar
132 107 110 1
133 108 111 Ar
134 108 112 1
135 109 113 Ar
136 109 114 1
137 111 113 Ar
138 111 115 1
139 113 116 1
140 117 118 Ar
141 117 119 Ar
142 118 120 Ar
143 118 121 1
144 119 122 Ar
145 119 123 1
146 120 124 Ar
147 120 125 1
148 122 124 Ar
149 122 126 1
150 124 127 1
151 128 129 Ar
152 128 130 Ar
153 129 131 Ar
154 129 132 1
155 130 133 Ar
156 130 134 1
157 131 135 Ar
158 131 136 1
159 133 135 Ar
160 133 137 1
161 135 138 1
162 139 140 Ar
163 139 141 Ar
164 140 142 Ar
165 140 143 1
166 141 144 Ar
167 141 145 1
168 142 146 Ar
169 142 147 1
170 144 146 Ar
171 144 148 1
172 146 149 1

Zn-1 S_1

@<TRIPOS>MOLECULE
Molecule Name
149 172
SMALL
NO_CHARGES

@<TRIPOS>ATOM
1 Zn1 0.5541 0.7752 -1.7641 Zn
2 Zn2 1.0330 -1.3993 0.5730 Zn
3 Zn3 -0.1797 1.5330 1.3006 Zn

4	Zn4	-1.9682	-0.6171	-0.3303	Zn
5	O5	-0.1429	0.0720	-0.0504	O
6	N6	1.9655	-0.5449	-2.5217	N
7	N7	2.8378	-1.3814	-0.4558	N
8	C8	2.3280	-0.6746	-3.8007	C
9	C9	3.5748	-1.3853	-3.9066	C
10	C10	3.9961	-1.6609	-2.6135	C
11	C11	5.1209	-2.3786	-2.0270	C
12	C12	4.9899	-2.5507	-0.6042	C
13	C13	3.9419	-2.0524	0.1029	C
14	C14	2.9449	-1.1773	-1.7747	C
15	N15	0.2860	3.3128	0.3985	N
16	N16	1.5988	2.5779	-1.4683	N
17	C17	0.1196	4.5452	0.9877	C
18	C18	0.9016	5.5250	0.4209	C
19	C19	1.6316	4.8785	-0.6160	C
20	C20	2.5773	5.2584	-1.5811	C
21	C21	3.0095	4.2580	-2.4611	C
22	C22	2.5063	2.9647	-2.3775	C
23	C23	1.1903	3.5143	-0.5913	C
24	N24	-0.9667	1.0926	-3.0868	N
25	N25	-2.9341	0.4077	-1.8850	N
26	C26	-0.7938	1.7152	-4.3017	C
27	C27	-1.9771	1.9641	-4.9545	C
28	C28	-2.9967	1.4720	-4.0937	C
29	C29	-4.3990	1.4358	-4.1111	C
30	C30	-5.0243	0.9009	-2.9783	C
31	C31	-4.2764	0.4090	-1.9155	C
32	C32	-2.3078	0.9475	-2.9489	C
33	N33	1.4071	-1.1957	2.5655	N
34	N34	1.3346	1.1947	2.7464	N
35	C35	1.9025	-2.1762	3.3941	C
36	C36	2.5064	-1.6723	4.5226	C
37	C37	2.3983	-0.2569	4.4153	C
38	C38	2.7795	0.8519	5.1873	C
39	C39	2.4264	2.1115	4.6882	C
40	C40	1.7208	2.2381	3.4963	C
41	C41	1.6903	-0.0250	3.1908	C
42	N42	-1.9258	-2.6076	-0.7475	N
43	N43	0.2097	-3.2926	0.1195	N
44	C44	-2.9596	-3.3056	-1.3279	C
45	C45	-2.7329	-4.6605	-1.3760	C
46	C46	-1.4519	-4.8540	-0.7881	C
47	C47	-0.6088	-5.9498	-0.5485	C
48	C48	0.6425	-5.6628	0.0087	C
49	C49	1.0049	-4.3566	0.3155	C
50	C50	-0.9961	-3.5416	-0.4276	C
51	N51	-1.9720	1.5444	2.2930	N
52	N52	-3.1484	-0.3402	1.3786	N
53	C53	-2.2343	2.3675	3.3637	C
54	C54	-3.4058	2.0566	4.0120	C
55	C55	-3.9424	0.9407	3.3130	C
56	C56	-5.0605	0.1048	3.4515	C
57	C57	-5.1595	-0.9626	2.5500	C
58	C58	-4.2085	-1.1465	1.5538	C
59	C59	-3.0058	0.6690	2.2588	C
60	H60	1.7216	-0.2780	-4.6058	H
61	H61	4.0438	-1.6811	-4.8356	H
62	H62	5.7963	-3.0150	-0.0462	H
63	H63	3.9153	-2.1331	1.1864	H

64	H64	-0.5918	4.6573	1.7987	H
65	H65	0.9246	6.5709	0.6921	H
66	H66	3.7664	4.4797	-3.2066	H
67	H67	2.8504	2.1948	-3.0639	H
68	H68	0.2061	1.9727	-4.6339	H
69	H69	-2.0991	2.4618	-5.9059	H
70	H70	-6.1064	0.8325	-2.9337	H
71	H71	1.7745	-3.2186	3.1241	H
72	H72	2.9420	-2.2420	5.3312	H
73	H73	2.7252	3.0100	5.2186	H
74	H74	1.4602	3.2221	3.1135	H
75	H75	-1.5307	3.1510	3.6223	H
76	H76	-3.8081	2.5439	4.8887	H
77	H77	-6.0048	-1.6414	2.5990	H
78	H78	-4.2930	-1.9797	0.8603	H
79	H79	1.9871	-4.1518	0.7336	H
80	H80	1.3374	-6.4654	0.2332	H
81	H81	-3.8221	-2.7670	-1.7058	H
82	H82	-3.3812	-5.4101	-1.8069	H
83	H83	-4.7745	-0.0068	-1.0434	H
84	C84	6.2813	-2.8105	-2.7503	C
85	C85	7.1110	-3.8507	-2.2546	C
86	C86	6.6694	-2.2170	-3.9791	C
87	C87	8.2286	-4.2768	-2.9509	C
88	H88	6.8397	-4.3548	-1.3318	H
89	C89	7.7924	-2.6466	-4.6669	C
90	H90	6.1121	-1.3659	-4.3557	H
91	C91	8.5816	-3.6843	-4.1666	C
92	H92	8.8302	-5.0882	-2.5487	H
93	H93	8.0681	-2.1561	-5.5972	H
94	H94	9.4602	-4.0207	-4.7093	H
95	C95	-5.1953	1.9328	-5.2508	C
96	C96	-6.3696	2.6652	-5.0333	C
97	C97	-4.8069	1.6650	-6.5702	C
98	C98	-7.1308	3.1217	-6.1036	C
99	H99	-6.6703	2.8990	-4.0152	H
100	C100	-5.5733	2.1152	-7.6398	C
101	H101	-3.9116	1.0771	-6.7508	H
102	C102	-6.7361	2.8471	-7.4109	C
103	H103	-8.0326	3.6985	-5.9161	H
104	H104	-5.2634	1.8887	-8.6566	H
105	H105	-7.3317	3.2025	-8.2474	H
106	C106	-0.9990	-7.3395	-0.8591	C
107	C107	-0.0742	-8.2335	-1.4146	C
108	C108	-2.2937	-7.7996	-0.5843	C
109	C109	-0.4345	-9.5477	-1.6920	C
110	H110	0.9268	-7.8840	-1.6538	H
111	C111	-2.6509	-9.1160	-0.8557	C
112	H112	-3.0120	-7.1233	-0.1303	H
113	C113	-1.7239	-9.9940	-1.4125	C
114	H114	0.2929	-10.2238	-2.1333	H
115	H115	-3.6562	-9.4589	-0.6260	H
116	H116	-2.0052	-11.0213	-1.6279	H
117	C117	-6.0889	0.3158	4.4897	C
118	C118	-6.6395	-0.7722	5.1795	C
119	C119	-6.5503	1.6038	4.7926	C
120	C120	-7.6189	-0.5785	6.1475	C
121	H121	-6.2742	-1.7746	4.9714	H
122	C122	-7.5350	1.7958	5.7557	C
123	H123	-6.1484	2.4532	4.2479	H

124 C124 -8.0711 0.7064 6.4384 C
125 H125 -8.0257 -1.4336 6.6807 H
126 H126 -7.8886 2.8009 5.9703 H
127 H127 -8.8372 0.8581 7.1940 H
128 C128 3.5234 0.7235 6.4563 C
129 C129 3.2024 1.5324 7.5544 C
130 C130 4.5765 -0.1919 6.5845 C
131 C131 3.9089 1.4256 8.7472 C
132 H132 2.3735 2.2309 7.4758 H
133 C133 5.2872 -0.2930 7.7756 C
134 H134 4.8522 -0.8068 5.7326 H
135 C135 4.9548 0.5129 8.8620 C
136 H136 3.6379 2.0534 9.5919 H
137 H137 6.1082 -1.0009 7.8536 H
138 H138 5.5080 0.4299 9.7936 H
139 C139 3.1118 6.6314 -1.6778 C
140 C140 3.3198 7.2292 -2.9277 C
141 C141 3.4425 7.3567 -0.5254 C
142 C142 3.8378 8.5161 -3.0233 C
143 H143 3.0468 6.6866 -3.8291 H
144 C144 3.9671 8.6409 -0.6222 C
145 H145 3.3105 6.8946 0.4485 H
146 C146 4.1643 9.2265 -1.8706 C
147 H147 3.9817 8.9677 -4.0013 H
148 H148 4.2285 9.1847 0.2818 H
149 H149 4.5706 10.2316 -1.9448 H
@<TRIPOS>BOND
1 1 5 1
2 1 6 1
3 1 16 1
4 1 24 1
5 2 5 1
6 2 7 1
7 2 33 1
8 2 43 1
9 3 5 1
10 3 15 1
11 3 34 1
12 3 51 1
13 4 5 1
14 4 25 1
15 4 42 1
16 4 52 1
17 6 8 1
18 6 14 Ar
19 7 13 Ar
20 7 14 Ar
21 8 9 Ar
22 8 60 1
23 9 10 Ar
24 9 61 1
25 10 11 Ar
26 10 14 1
27 11 12 Ar
28 11 84 1
29 12 13 Ar
30 12 62 1
31 13 63 1
32 15 17 1
33 15 23 Ar

34 16 22 Ar
35 16 23 Ar
36 17 18 Ar
37 17 64 1
38 18 19 Ar
39 18 65 1
40 19 20 Ar
41 19 23 1
42 20 21 Ar
43 20 139 1
44 21 22 Ar
45 21 66 1
46 22 67 1
47 24 26 1
48 24 32 Ar
49 25 31 Ar
50 25 32 Ar
51 26 27 Ar
52 26 68 1
53 27 28 Ar
54 27 69 1
55 28 29 Ar
56 28 32 1
57 29 30 Ar
58 29 95 1
59 30 31 Ar
60 30 70 1
61 31 83 1
62 33 35 1
63 33 41 Ar
64 34 40 Ar
65 34 41 Ar
66 35 36 Ar
67 35 71 1
68 36 37 Ar
69 36 72 1
70 37 38 Ar
71 37 41 1
72 38 39 Ar
73 38 128 1
74 39 40 Ar
75 39 73 1
76 40 74 1
77 42 44 1
78 42 50 Ar
79 43 49 Ar
80 43 50 Ar
81 44 45 Ar
82 44 81 1
83 45 46 Ar
84 45 82 1
85 46 47 Ar
86 46 50 1
87 47 48 Ar
88 47 106 1
89 48 49 Ar
90 48 80 1
91 49 79 1
92 51 53 1
93 51 59 Ar

94 52 58 Ar
95 52 59 Ar
96 53 54 Ar
97 53 75 1
98 54 55 Ar
99 54 76 1
100 55 56 Ar
101 55 59 1
102 56 57 Ar
103 56 117 1
104 57 58 Ar
105 57 77 1
106 58 78 1
107 84 85 Ar
108 84 86 Ar
109 85 87 Ar
110 85 88 1
111 86 89 Ar
112 86 90 1
113 87 91 Ar
114 87 92 1
115 89 91 Ar
116 89 93 1
117 91 94 1
118 95 96 Ar
119 95 97 Ar
120 96 98 Ar
121 96 99 1
122 97 100 Ar
123 97 101 1
124 98 102 Ar
125 98 103 1
126 100 102 Ar
127 100 104 1
128 102 105 1
129 106 107 Ar
130 106 108 Ar
131 107 109 Ar
132 107 110 1
133 108 111 Ar
134 108 112 1
135 109 113 Ar
136 109 114 1
137 111 113 Ar
138 111 115 1
139 113 116 1
140 117 118 Ar
141 117 119 Ar
142 118 120 Ar
143 118 121 1
144 119 122 Ar
145 119 123 1
146 120 124 Ar
147 120 125 1
148 122 124 Ar
149 122 126 1
150 124 127 1
151 128 129 Ar
152 128 130 Ar
153 129 131 Ar

154 129 132 1
155 130 133 Ar
156 130 134 1
157 131 135 Ar
158 131 136 1
159 133 135 Ar
160 133 137 1
161 135 138 1
162 139 140 Ar
163 139 141 Ar
164 140 142 Ar
165 140 143 1
166 141 144 Ar
167 141 145 1
168 142 146 Ar
169 142 147 1
170 144 146 Ar
171 144 148 1
172 146 149 1

Zn-2 S_0
@<TRIPOS>MOLECULE
Molecule Name
131 148
SMALL
NO_CHARGES

@<TRIPOS>ATOM
1 Zn1 -0.4902 -0.4774 -1.8451 Zn
2 Zn2 -0.7856 1.5494 0.6460 Zn
3 Zn3 -0.0722 -1.5727 1.1865 Zn
4 Zn4 2.1106 0.3999 -0.1470 Zn
5 O5 0.1904 -0.0250 -0.0313 O
6 N6 -1.6751 1.0501 -2.5295 N
7 N7 -2.5516 1.8654 -0.4581 N
8 C8 -2.0828 1.1956 -3.8343 C
9 C9 -3.2487 1.9174 -3.9582 C
10 C10 -3.6275 2.2639 -2.6308 C
11 C11 -4.6799 2.9646 -2.0157 C
12 C12 -4.6309 3.0660 -0.6162 C
13 C13 -3.5747 2.5270 0.1064 C
14 C14 -2.6010 1.7129 -1.7942 C
15 N15 -0.6917 -3.2061 0.1273 N
16 N16 -1.7946 -2.1409 -1.7112 N
17 C17 -0.7293 -4.4899 0.6180 C
18 C18 -1.6092 -5.3014 -0.0612 C
19 C19 -2.1881 -4.4793 -1.0684 C
20 C20 -3.1404 -4.6338 -2.0908 C
21 C21 -3.3978 -3.5033 -2.8812 C
22 C22 -2.7158 -2.3115 -2.6724 C
23 C23 -1.5639 -3.1978 -0.9111 C
24 N24 1.0754 -0.9705 -3.0625 N
25 N25 3.0284 -0.6629 -1.6964 N
26 C26 0.8992 -1.4931 -4.3216 C
27 C27 2.0739 -1.8976 -4.9095 C
28 C28 3.0910 -1.6202 -3.9562 C
29 C29 4.4829 -1.7941 -3.8804 C
30 C30 5.0977 -1.4064 -2.6801 C
31 C31 4.3554 -0.8655 -1.6410 C

32	C32	2.4082	-1.0477	-2.8287	C
33	N33	-1.3471	1.2591	2.5823	N
34	N34	-1.6532	-1.1168	2.5331	N
35	C35	-1.7652	2.2340	3.4571	C
36	C36	-2.5415	1.7415	4.4808	C
37	C37	-2.6376	0.3403	4.2490	C
38	C38	-3.2621	-0.7566	4.8684	C
39	C39	-3.0686	-2.0081	4.2650	C
40	C40	-2.2717	-2.1441	3.1354	C
41	C41	-1.8613	0.1003	3.0680	C
42	N42	2.3668	2.4038	-0.4134	N
43	N43	0.3008	3.3335	0.3850	N
44	C44	3.5302	2.9775	-0.8680	C
45	C45	3.5001	4.3516	-0.8396	C
46	C46	2.2126	4.6946	-0.3431	C
47	C47	1.5179	5.8837	-0.0655	C
48	C48	0.2149	5.7424	0.4348	C
49	C49	-0.3399	4.4871	0.6359	C
50	C50	1.5550	3.4411	-0.0951	C
51	N51	1.6136	-1.8958	2.3027	N
52	N52	3.1051	-0.1440	1.6191	N
53	C53	1.6728	-2.8184	3.3198	C
54	C54	2.8176	-2.7205	4.0750	C
55	C55	3.5560	-1.6468	3.5075	C
56	C56	4.7675	-0.9915	3.7829	C
57	C57	5.0882	0.1021	2.9639	C
58	C58	4.2516	0.4863	1.9259	C
59	C59	2.7561	-1.1759	2.4106	C
60	H60	-1.4828	0.7710	-4.6318	H
61	H61	-3.7433	2.1806	-4.8820	H
62	H62	-5.4383	3.5505	-0.0780	H
63	H63	-3.5473	2.6129	1.1902	H
64	H64	-0.0850	-4.7657	1.4456	H
65	H65	-1.7887	-6.3502	0.1264	H
66	H66	-4.1557	-3.5427	-3.6560	H
67	H67	-2.9246	-1.4435	-3.2932	H
68	H68	-0.0997	-1.5647	-4.7381	H
69	H69	2.1797	-2.3557	-5.8821	H
70	H70	6.1723	-1.4959	-2.5641	H
71	H71	-1.4558	3.2615	3.3006	H
72	H72	-2.9597	2.3107	5.2984	H
73	H73	-3.5629	-2.8868	4.6649	H
74	H74	-2.1314	-3.1202	2.6773	H
75	H75	0.8522	-3.5123	3.4643	H
76	H76	3.0721	-3.3232	4.9346	H
77	H77	6.0168	0.6420	3.1140	H
78	H78	4.5078	1.3406	1.3041	H
79	H79	-1.3632	4.4004	0.9918	H
80	H80	-0.3928	6.6193	0.6290	H
81	H81	4.3408	2.3439	-1.2118	H
82	H82	4.2987	5.0161	-1.1353	H
83	H83	4.8458	-0.5737	-0.7157	H
84	C84	-4.0799	-0.6566	6.0739	C
85	C85	-4.2701	-1.6234	7.0357	C
86	S86	-5.0117	0.7576	6.4594	S
87	C87	-5.1527	-1.2259	8.0711	C
88	H88	-3.7628	-2.5817	7.0088	H
89	C89	-5.6343	0.0410	7.8892	C
90	H90	-5.4145	-1.8502	8.9182	H
91	H91	-6.3206	0.5935	8.5175	H

92 C92 5.6750 -1.3756 4.8602 C
93 C93 6.5318 -0.5578 5.5630 C
94 S94 5.8416 -3.0179 5.4007 S
95 C95 7.3083 -1.2478 6.5271 C
96 H96 6.5759 0.5146 5.4070 H
97 C97 7.0424 -2.5891 6.5492 C
98 H98 8.0288 -0.7693 7.1813 H
99 H99 7.4856 -3.3496 7.1787 H
100 C100 2.0705 7.2202 -0.2649 C
101 C101 1.7553 8.3641 0.4345 C
102 S102 3.2315 7.5815 -1.5049 S
103 C103 2.4452 9.5146 -0.0222 C
104 H104 1.0670 8.3657 1.2728 H
105 C105 3.2789 9.2409 -1.0713 C
106 H106 2.3331 10.5019 0.4119 H
107 H107 3.9186 9.9241 -1.6144 H
108 C108 5.2947 -2.3537 -4.9572 C
109 C109 6.4733 -3.0557 -4.8361 C
110 S110 4.8878 -2.1578 -6.6343 S
111 C111 7.0370 -3.4333 -6.0804 C
112 H112 6.9027 -3.3165 -3.8749 H
113 C113 6.2866 -3.0115 -7.1431 C
114 H114 7.9563 -3.9993 -6.1835 H
115 H115 6.4784 -3.1568 -8.1982 H
116 C116 -3.8549 -5.8787 -2.3585 C
117 C117 -4.3586 -6.3101 -3.5654 C
118 S118 -4.2285 -7.0299 -1.1125 S
119 C119 -5.0329 -7.5542 -3.4852 C
120 H120 -4.2195 -5.7568 -4.4878 H
121 C121 -5.0434 -8.0617 -2.2154 C
122 H122 -5.4854 -8.0550 -4.3340 H
123 H123 -5.4846 -8.9865 -1.8671 H
124 C124 -5.7910 3.5711 -2.7432 C
125 C125 -6.5394 4.6641 -2.3666 C
126 S126 -6.3829 2.9345 -4.2467 S
127 C127 -7.5732 4.9847 -3.2817 C
128 H128 -6.3294 5.2358 -1.4692 H
129 C129 -7.6095 4.1300 -4.3486 C
130 H130 -8.2568 5.8173 -3.1576 H
131 H131 -8.2898 4.1376 -5.1902 H
@<TRIPOS>BOND
1 1 5 1
2 1 6 1
3 1 24 1
4 2 5 1
5 2 33 1
6 3 5 1
7 3 15 1
8 3 51 1
9 4 5 1
10 4 42 1
11 6 8 Ar
12 6 14 Ar
13 7 13 Ar
14 7 14 Ar
15 8 9 2
16 8 60 1
17 9 10 Ar
18 9 61 1
19 10 11 Ar

20 10 14 Ar
21 11 12 Ar
22 11 124 1
23 12 13 Ar
24 12 62 1
25 13 63 1
26 15 17 Ar
27 15 23 Ar
28 16 22 Ar
29 16 23 Ar
30 17 18 2
31 17 64 1
32 18 19 Ar
33 18 65 1
34 19 20 Ar
35 19 23 Ar
36 20 21 Ar
37 20 116 1
38 21 22 Ar
39 21 66 1
40 22 67 1
41 24 26 Ar
42 24 32 Ar
43 25 31 Ar
44 25 32 Ar
45 26 27 2
46 26 68 1
47 27 28 Ar
48 27 69 1
49 28 29 Ar
50 28 32 Ar
51 29 30 Ar
52 29 108 1
53 30 31 Ar
54 30 70 1
55 31 83 1
56 33 35 Ar
57 33 41 Ar
58 34 40 Ar
59 34 41 Ar
60 35 36 2
61 35 71 1
62 36 37 Ar
63 36 72 1
64 37 38 Ar
65 37 41 Ar
66 38 39 Ar
67 38 84 1
68 39 40 Ar
69 39 73 1
70 40 74 1
71 42 44 Ar
72 42 50 Ar
73 43 49 Ar
74 43 50 Ar
75 44 45 2
76 44 81 1
77 45 46 Ar
78 45 82 1
79 46 47 Ar

80 46 50 Ar
81 47 48 Ar
82 47 100 1
83 48 49 Ar
84 48 80 1
85 49 79 1
86 51 53 Ar
87 51 59 Ar
88 52 58 Ar
89 52 59 Ar
90 53 54 2
91 53 75 1
92 54 55 Ar
93 54 76 1
94 55 56 Ar
95 55 59 Ar
96 56 57 Ar
97 56 92 1
98 57 58 Ar
99 57 77 1
100 58 78 1
101 84 85 2
102 84 86 1
103 85 87 Ar
104 85 88 1
105 86 89 1
106 87 89 2
107 87 90 1
108 89 91 1
109 92 93 2
110 92 94 1
111 93 95 Ar
112 93 96 1
113 94 97 1
114 95 97 2
115 95 98 1
116 97 99 1
117 100 101 2
118 100 102 1
119 101 103 Ar
120 101 104 1
121 102 105 1
122 103 105 2
123 103 106 1
124 105 107 1
125 108 109 2
126 108 110 1
127 109 111 Ar
128 109 112 1
129 110 113 1
130 111 113 2
131 111 114 1
132 113 115 1
133 116 117 2
134 116 118 1
135 117 119 Ar
136 117 120 1
137 118 121 1
138 119 121 2
139 119 122 1

140 121 123 1
141 124 125 2
142 124 126 1
143 125 127 Ar
144 125 128 1
145 126 129 1
146 127 129 2
147 127 130 1
148 129 131 1

Zn-2 S_1
@<TRIPOS>MOLECULE
Molecule Name
131 154
SMALL
NO_CHARGES

@<TRIPOS>ATOM
1 Zn1 -0.4856 -0.9545 -1.6180 Zn
2 Zn2 -1.0363 1.5493 0.3414 Zn
3 Zn3 0.1616 -1.2051 1.5530 Zn
4 Zn4 1.9910 0.6783 -0.3256 Zn
5 O5 0.1631 0.0206 -0.0006 O
6 N6 -1.8896 0.2004 -2.5727 N
7 N7 -2.8503 1.3406 -0.7029 N
8 C8 -2.3335 -0.0241 -3.8541 C
9 C9 -3.5975 0.4721 -4.0826 C
10 C10 -4.0062 1.0601 -2.8528 C
11 C11 -5.1441 1.7242 -2.3623 C
12 C12 -5.0908 2.1553 -1.0269 C
13 C13 -3.9543 1.9593 -0.2538 C
14 C14 -2.8953 0.8745 -1.9642 C
15 N15 -0.2006 -3.1396 0.9401 N
16 N16 -1.4949 -2.6893 -1.0191 N
17 C17 0.0387 -4.2477 1.6605 C
18 C18 -0.6838 -5.3632 1.1540 C
19 C19 -1.4128 -4.9032 0.0621 C
20 C20 -2.3415 -5.4945 -0.8915 C
21 C21 -2.8300 -4.5393 -1.8735 C
22 C22 -2.4176 -3.2498 -1.8991 C
23 C23 -1.0719 -3.5193 -0.0583 C
24 N24 1.1206 -1.4772 -2.7754 N
25 N25 3.0323 -0.5760 -1.6333 N
26 C26 0.9986 -2.3114 -3.8613 C
27 C27 2.2085 -2.6766 -4.4023 C
28 C28 3.1919 -2.0342 -3.6023 C
29 C29 4.5952 -1.9863 -3.5654 C
30 C30 5.1693 -1.2351 -2.5283 C
31 C31 4.3755 -0.5689 -1.6066 C
32 C32 2.4538 -1.3066 -2.6062 C
33 N33 -1.5084 1.6377 2.3234 N
34 N34 -1.4354 -0.6992 2.8503 N
35 C35 -2.0548 2.7211 2.9703 C
36 C36 -2.7189 2.3796 4.1258 C
37 C37 -2.5971 0.9657 4.2343 C
38 C38 -3.0236 -0.0292 5.1312 C
39 C39 -2.6463 -1.3462 4.8288 C
40 C40 -1.8658 -1.6299 3.7159 C
41 C41 -1.8212 0.5668 3.0962 C

42	N42	1.9308	2.5800	-1.0514	N
43	N43	-0.2423	3.3585	-0.3839	N
44	C44	2.9792	3.1912	-1.6971	C
45	C45	2.7307	4.5088	-1.9999	C
46	C46	1.4147	4.7668	-1.5276	C
47	C47	0.5448	5.8695	-1.5073	C
48	C48	-0.7085	5.6646	-0.9113	C
49	C49	-1.0528	4.4298	-0.3814	C
50	C50	0.9699	3.5299	-0.9467	C
51	N51	1.9034	-1.0369	2.6121	N
52	N52	3.0967	0.6997	1.4615	N
53	C53	2.1256	-1.6778	3.8076	C
54	C54	3.2630	-1.2490	4.4498	C
55	C55	3.8181	-0.2461	3.6091	C
56	C56	4.9254	0.6172	3.6559	C
57	C57	5.0566	1.5246	2.5936	C
58	C58	4.1434	1.5375	1.5488	C
59	C59	2.9284	-0.1593	2.4846	C
60	H60	-1.6881	-0.5334	-4.5613	H
61	H61	-4.1399	0.4364	-5.0163	H
62	H62	-5.9550	2.6289	-0.5740	H
63	H63	-3.9243	2.2991	0.7789	H
64	H64	0.7184	-4.2331	2.5041	H
65	H65	-0.6370	-6.3638	1.5606	H
66	H66	-3.5598	-4.8531	-2.6103	H
67	H67	-2.8159	-2.5596	-2.6382	H
68	H68	0.0135	-2.6253	-4.1886	H
69	H69	2.3618	-3.3390	-5.2418	H
70	H70	6.2473	-1.1410	-2.4554	H
71	H71	-1.9164	3.7152	2.5597	H
72	H72	-3.2029	3.0601	4.8115	H
73	H73	-2.9835	-2.1656	5.4541	H
74	H74	-1.5801	-2.6554	3.4947	H
75	H75	1.4195	-2.4227	4.1580	H
76	H76	3.6269	-1.5916	5.4075	H
77	H77	5.8976	2.2087	2.5614	H
78	H78	4.2520	2.2520	0.7365	H
79	H79	-2.0425	4.2820	0.0428	H
80	H80	-1.4440	6.4614	-0.9003	H
81	H81	3.8742	2.6216	-1.9235	H
82	H82	3.4074	5.1919	-2.4923	H
83	H83	4.8352	0.0045	-0.8056	H
84	C84	-3.8188	0.2360	6.3266	C
85	C85	-3.8311	-0.4877	7.4981	C
86	S86	-4.9549	1.5462	6.4243	S
87	C87	-4.7420	0.0119	8.4621	C
88	H88	-3.1790	-1.3378	7.6666	H
89	C89	-5.4229	1.1111	8.0169	C
90	H90	-4.8823	-0.4213	9.4463	H
91	H91	-6.1741	1.6903	8.5379	H
92	C92	5.9086	0.6237	4.7352	C
93	C93	6.6525	1.6934	5.1811	C
94	S94	6.3325	-0.8080	5.6224	S
95	C95	7.5490	1.3616	6.2275	C
96	H96	6.5320	2.6956	4.7844	H
97	C97	7.4866	0.0388	6.5688	C
98	H98	8.2076	2.0745	6.7112	H
99	H99	8.0552	-0.4852	7.3260	H
100	C100	0.8726	7.1800	-2.0611	C
101	C101	0.3990	8.4036	-1.6426	C

102 S102 1.9246 7.3843 -3.4278 S
103 C103 0.8831 9.4908 -2.4121 C
104 H104 -0.2562 8.5150 -0.7854 H
105 C105 1.7189 9.0877 -3.4167 C
106 H106 0.6273 10.5280 -2.2257 H
107 H107 2.2254 9.6988 -4.1525 H
108 C108 5.4597 -2.6724 -4.5213 C
109 C109 6.7303 -3.1556 -4.3010 C
110 S110 4.9959 -2.9470 -6.1724 S
111 C111 7.3188 -3.7430 -5.4487 C
112 H112 7.2118 -3.1140 -3.3299 H
113 C113 6.4943 -3.6986 -6.5389 C
114 H114 8.3087 -4.1856 -5.4640 H
115 H115 6.6846 -4.0680 -7.5382 H
116 C116 -2.7719 -6.8143 -0.9051 C
117 C117 -3.6765 -7.4295 -1.8250 C
118 S118 -2.2530 -8.0256 0.2717 S
119 C119 -3.9094 -8.7782 -1.5689 C
120 H120 -4.1302 -6.8899 -2.6467 H
121 C121 -3.2181 -9.2579 -0.4720 C
122 H122 -4.5667 -9.4005 -2.1671 H
123 H123 -3.2226 -10.2601 -0.0651 H
124 C124 -6.3454 1.9760 -3.1529 C
125 C125 -7.2476 3.0050 -2.9987 C
126 S126 -6.8528 0.9285 -4.4420 S
127 C127 -8.3304 2.9500 -3.9114 C
128 H128 -7.1158 3.7938 -2.2661 H
129 C129 -8.2495 1.8754 -4.7534 C
130 H130 -9.1321 3.6795 -3.9466 H
131 H131 -8.9341 1.5881 -5.5407 H
@<TRIPOS>BOND
1 1 5 1
2 1 6 1
3 1 16 1
4 1 24 1
5 2 5 1
6 2 7 1
7 2 33 1
8 2 43 1
9 3 5 1
10 3 15 1
11 3 34 1
12 3 51 1
13 4 5 1
14 4 25 1
15 4 42 1
16 4 52 1
17 6 8 1
18 6 14 Ar
19 7 13 Ar
20 7 14 Ar
21 8 9 Ar
22 8 60 1
23 9 10 Ar
24 9 61 1
25 10 11 Ar
26 10 14 1
27 11 12 Ar
28 11 124 1
29 12 13 Ar

30 12 62 1
31 13 63 1
32 15 17 1
33 15 23 Ar
34 16 22 Ar
35 16 23 Ar
36 17 18 Ar
37 17 64 1
38 18 19 Ar
39 18 65 1
40 19 20 Ar
41 19 23 1
42 20 21 Ar
43 20 116 1
44 21 22 Ar
45 21 66 1
46 22 67 1
47 24 26 1
48 24 32 Ar
49 25 31 Ar
50 25 32 Ar
51 26 27 Ar
52 26 68 1
53 27 28 Ar
54 27 69 1
55 28 29 Ar
56 28 32 1
57 29 30 Ar
58 29 108 1
59 30 31 Ar
60 30 70 1
61 31 83 1
62 33 35 1
63 33 41 Ar
64 34 40 Ar
65 34 41 Ar
66 35 36 Ar
67 35 71 1
68 36 37 Ar
69 36 72 1
70 37 38 Ar
71 37 41 1
72 38 39 Ar
73 38 84 1
74 39 40 Ar
75 39 73 1
76 40 74 1
77 42 44 1
78 42 50 Ar
79 43 49 Ar
80 43 50 Ar
81 44 45 Ar
82 44 81 1
83 45 46 Ar
84 45 82 1
85 46 47 Ar
86 46 50 1
87 47 48 Ar
88 47 100 1
89 48 49 Ar

90 48 80 1
91 49 79 1
92 51 53 1
93 51 59 Ar
94 52 58 Ar
95 52 59 Ar
96 53 54 Ar
97 53 75 1
98 54 55 Ar
99 54 76 1
100 55 56 Ar
101 55 59 1
102 56 57 Ar
103 56 92 1
104 57 58 Ar
105 57 77 1
106 58 78 1
107 84 85 Ar
108 84 86 2
109 85 87 1
110 85 88 1
111 86 89 2
112 87 89 Ar
113 87 90 1
114 89 91 1
115 92 93 Ar
116 92 94 2
117 93 95 1
118 93 96 1
119 94 97 2
120 95 97 Ar
121 95 98 1
122 97 99 1
123 100 101 Ar
124 100 102 2
125 101 103 1
126 101 104 1
127 102 105 2
128 103 105 Ar
129 103 106 1
130 105 107 1
131 108 109 Ar
132 108 110 2
133 109 111 1
134 109 112 1
135 110 113 2
136 111 113 Ar
137 111 114 1
138 113 115 1
139 116 117 Ar
140 116 118 2
141 117 119 1
142 117 120 1
143 118 121 2
144 119 121 Ar
145 119 122 1
146 121 123 1
147 124 125 Ar
148 124 126 2
149 125 127 1

150 125 128 1
151 126 129 2
152 127 129 Ar
153 127 130 1
154 129 131 1

Zn-3 S_0
@<TRIPOS>MOLECULE
Molecule Name
185 208
SMALL
NO_CHARGES

@<TRIPOS>ATOM
1 Zn1 0.2176 0.6836 1.7289 Zn
2 Zn2 0.7193 0.9448 -1.4532 Zn
3 Zn3 0.1844 -1.9776 -0.1324 Zn
4 Zn4 -2.2059 0.1902 -0.3390 Zn
5 O5 -0.2670 -0.0440 -0.0588 O
6 N6 1.2416 2.4421 1.4925 N
7 N7 2.3101 2.0485 -0.6144 N
8 C8 1.4749 3.3515 2.4979 C
9 C9 2.5664 4.1546 2.2558 C
10 C10 3.0814 3.7314 0.9979 C
11 C11 4.1435 4.0986 0.1561 C
12 C12 4.2520 3.4015 -1.0548 C
13 C13 3.3398 2.4090 -1.3963 C
14 C14 2.2042 2.6816 0.5687 C
15 N15 0.7433 -2.6459 1.7172 N
16 N16 1.5863 -0.6206 2.6812 N
17 C17 0.8827 -3.9694 2.0662 C
18 C18 1.7089 -4.1569 3.1507 C
19 C19 2.1381 -2.8549 3.5340 C
20 C20 2.9599 -2.3074 4.5322 C
21 C21 3.0674 -0.9111 4.5571 C
22 C22 2.3821 -0.1220 3.6393 C
23 C23 1.4891 -1.9615 2.6199 C
24 N24 -1.4511 0.8068 2.9034 N
25 N25 -3.2549 0.1191 1.4700 N
26 C26 -1.3940 1.0883 4.2486 C
27 C27 -2.6117 0.9783 4.8765 C
28 C28 -3.5288 0.5896 3.8619 C
29 C29 -4.8942 0.2715 3.8039 C
30 C30 -5.3866 -0.1461 2.5607 C
31 C31 -4.5579 -0.2048 1.4478 C
32 C32 -2.7472 0.4939 2.6605 C
33 N33 1.5408 -0.3415 -2.8026 N
34 N34 1.8994 -2.2273 -1.3639 N
35 C35 2.0437 -0.0073 -4.0392 C
36 C36 2.9600 -0.9181 -4.5116 C
37 C37 3.0613 -1.9180 -3.5033 C
38 C38 3.7809 -3.1107 -3.3242 C
39 C39 3.5288 -3.8205 -2.1440 C
40 C40 2.6051 -3.3579 -1.2125 C
41 C41 2.1436 -1.5148 -2.4793 C
42 N42 -2.5632 1.9528 -1.2917 N
43 N43 -0.4661 2.4277 -2.3681 N
44 C44 -3.7874 2.5796 -1.3245 C
45 C45 -3.8045 3.6831 -2.1436 C

46	C46	-2.4877	3.7869	-2.6711	C
47	C47	-1.8217	4.6484	-3.5556	C
48	C48	-0.4813	4.3482	-3.8276	C
49	C49	0.1445	3.2667	-3.2202	C
50	C50	-1.7638	2.6819	-2.1078	C
51	N51	-1.3472	-3.0349	-0.9813	N
52	N52	-2.9862	-1.3622	-1.5176	N
53	C53	-1.2555	-4.3724	-1.2881	C
54	C54	-2.3223	-4.8342	-2.0217	C
55	C55	-3.1687	-3.7076	-2.2115	C
56	C56	-4.3746	-3.4522	-2.8821	C
57	C57	-4.8249	-2.1255	-2.8754	C
58	C58	-4.1220	-1.1362	-2.1985	C
59	C59	-2.5072	-2.6206	-1.5458	C
60	H60	0.8097	3.3815	3.3540	H
61	H61	2.9281	4.9582	2.8814	H
62	H62	5.0732	3.6121	-1.7323	H
63	H63	3.4381	1.8668	-2.3341	H
64	H64	0.3465	-4.7284	1.5070	H
65	H65	1.9472	-5.0978	3.6262	H
66	H66	3.7143	-0.4275	5.2821	H
67	H67	2.4794	0.9609	3.6617	H
68	H68	-0.4428	1.3455	4.7016	H
69	H69	-2.8117	1.1239	5.9283	H
70	H70	-6.4360	-0.3968	2.4442	H
71	H71	1.6879	0.8884	-4.5363	H
72	H72	3.4660	-0.8893	-5.4662	H
73	H73	4.0771	-4.7320	-1.9286	H
74	H74	2.4272	-3.9106	-0.2930	H
75	H75	-0.3886	-4.9381	-0.9648	H
76	H76	-2.4611	-5.8372	-2.3992	H
77	H77	-5.7532	-1.8602	-3.3710	H
78	H78	-4.4809	-0.1098	-2.2002	H
79	H79	1.1981	3.0767	-3.4074	H
80	H80	0.1010	4.9900	-4.4808	H
81	H81	-4.6032	2.1897	-0.7252	H
82	H82	-4.6500	4.3250	-2.3454	H
83	H83	-4.9538	-0.5284	0.4885	H
84	C84	-5.7875	0.3607	4.9749	C
85	C85	-6.7880	-0.6278	5.1849	C
86	C86	-5.6928	1.4095	5.8692	C
87	C87	-7.6430	-0.5524	6.2535	C
88	H88	-6.8501	-1.4681	4.4988	H
89	C89	-6.5658	1.5191	6.9771	C
90	H90	-4.9517	2.1882	5.7083	H
91	C91	-7.5630	0.5183	7.1786	C
92	H92	-8.3938	-1.3240	6.4079	H
93	C93	-6.4829	2.5973	7.8946	C
94	C94	-8.4333	0.6285	8.2902	C
95	C95	-7.3419	2.6778	8.9632	C
96	H96	-5.7245	3.3608	7.7374	H
97	C97	-8.3264	1.6836	9.1637	C
98	H98	-9.1902	-0.1380	8.4409	H
99	H99	-7.2676	3.5083	9.6600	H
100	H100	-9.0000	1.7583	10.0130	H
101	C101	-5.1453	-4.5083	-3.5662	C
102	C102	-5.7790	-4.2409	-4.8107	C
103	C103	-5.2894	-5.7620	-3.0040	C
104	C104	-6.5140	-5.2048	-5.4508	C
105	H105	-5.6491	-3.2640	-5.2686	H

106	C106	-6.0491	-6.7744	-3.6356	C
107	H107	-4.8337	-5.9746	-2.0403	H
108	C108	-6.6747	-6.4955	-4.8877	C
109	H109	-6.9803	-4.9891	-6.4095	H
110	C110	-6.2105	-8.0607	-3.0608	C
111	C111	-7.4299	-7.5128	-5.5205	C
112	C112	-6.9501	-9.0267	-3.6976	C
113	H113	-5.7358	-8.2697	-2.1048	H
114	C114	-7.5651	-8.7508	-4.9400	C
115	H115	-7.9027	-7.2967	-6.4758	H
116	H116	-7.0653	-10.0091	-3.2478	H
117	H117	-8.1475	-9.5235	-5.4344	H
118	C118	-2.4709	5.8223	-4.1703	C
119	C119	-2.2146	6.1484	-5.5303	C
120	C120	-3.3116	6.6383	-3.4383	C
121	C121	-2.7937	7.2447	-6.1151	C
122	H122	-1.5707	5.4969	-6.1151	H
123	C123	-3.9160	7.7821	-4.0109	C
124	H124	-3.4944	6.4200	-2.3893	H
125	C125	-3.6572	8.0942	-5.3793	C
126	H126	-2.5994	7.4731	-7.1606	H
127	C127	-4.7714	8.6317	-3.2642	C
128	C128	-4.2684	9.2366	-5.9512	C
129	C129	-5.3484	9.7337	-3.8461	C
130	H130	-4.9633	8.3938	-2.2205	H
131	C131	-5.0956	10.0387	-5.2029	C
132	H132	-4.0705	9.4694	-6.9951	H
133	H133	-6.0030	10.3767	-3.2640	H
134	H134	-5.5584	10.9132	-5.6520	H
135	C135	5.1177	5.1494	0.5086	C
136	C136	5.5904	6.0477	-0.4873	C
137	C137	5.6097	5.2657	1.7943	C
138	C138	6.5112	7.0171	-0.1854	C
139	H139	5.1884	5.9752	-1.4942	H
140	C140	6.5678	6.2493	2.1347	C
141	H141	5.2797	4.5698	2.5613	H
142	C142	7.0300	7.1482	1.1270	C
143	H143	6.8520	7.7060	-0.9551	H
144	C144	7.0879	6.3678	3.4486	C
145	C145	7.9862	8.1344	1.4712	C
146	C146	8.0160	7.3335	3.7520	C
147	H147	6.7375	5.6790	4.2138	H
148	C148	8.4691	8.2262	2.7542	C
149	H149	8.3333	8.8188	0.7005	H
150	H150	8.4065	7.4149	4.7627	H
151	H151	9.2036	8.9861	3.0068	H
152	C152	3.6969	-3.1357	5.5062	C
153	C153	3.8021	-2.7199	6.8619	C
154	C154	4.3222	-4.3055	5.1205	C
155	C155	4.5038	-3.4616	7.7764	C
156	H156	3.2919	-1.8128	7.1745	H
157	C157	5.0626	-5.0872	6.0383	C
158	H158	4.2738	-4.6235	4.0822	H
159	C159	5.1566	-4.6611	7.3972	C
160	H160	4.5633	-3.1376	8.8130	H
161	C161	5.7206	-6.2812	5.6482	C
162	C162	5.8964	-5.4466	8.3144	C
163	C163	6.4330	-7.0208	6.5600	C
164	H164	5.6511	-6.6014	4.6111	H
165	C165	6.5211	-6.6003	7.9065	C

166 H166 5.9632 -5.1193 9.3495 H
167 H167 6.9326 -7.9342 6.2488 H
168 H168 7.0875 -7.1942 8.6188 H
169 C169 4.7686 -3.6036 -4.3036 C
170 C170 4.8515 -4.9925 -4.5969 C
171 C171 5.6466 -2.7396 -4.9288 C
172 C172 5.7776 -5.4729 -5.4862 C
173 H173 4.1474 -5.6722 -4.1248 H
174 C174 6.6202 -3.2060 -5.8430 C
175 H175 5.6145 -1.6784 -4.6953 H
176 C176 6.6895 -4.6018 -6.1330 C
177 H177 5.8193 -6.5362 -5.7110 H
178 C178 7.5350 -2.3284 -6.4783 C
179 C179 7.6627 -5.0656 -7.0512 C
180 C180 8.4697 -2.8081 -7.3628 C
181 H181 7.4831 -1.2659 -6.2522 H
182 C182 8.5337 -4.1900 -7.6531 C
183 H183 7.7102 -6.1298 -7.2711 H
184 H184 9.1650 -2.1256 -7.8438 H
185 H185 9.2775 -4.5574 -8.3549 H
@<TRIPOS>BOND
1 1 5 1
2 1 6 1
3 1 24 1
4 2 5 1
5 2 33 1
6 3 5 1
7 3 15 1
8 3 51 1
9 4 5 1
10 4 42 1
11 6 8 Ar
12 6 14 Ar
13 7 13 Ar
14 7 14 Ar
15 8 9 2
16 8 60 1
17 9 10 Ar
18 9 61 1
19 10 11 Ar
20 10 14 Ar
21 11 12 Ar
22 11 135 1
23 12 13 Ar
24 12 62 1
25 13 63 1
26 15 17 Ar
27 15 23 Ar
28 16 22 Ar
29 16 23 Ar
30 17 18 2
31 17 64 1
32 18 19 Ar
33 18 65 1
34 19 20 Ar
35 19 23 Ar
36 20 21 Ar
37 20 152 1
38 21 22 Ar
39 21 66 1

40 22 67 1
41 24 26 Ar
42 24 32 Ar
43 25 31 Ar
44 25 32 Ar
45 26 27 2
46 26 68 1
47 27 28 Ar
48 27 69 1
49 28 29 Ar
50 28 32 Ar
51 29 30 Ar
52 29 84 1
53 30 31 Ar
54 30 70 1
55 31 83 1
56 33 35 Ar
57 33 41 Ar
58 34 40 Ar
59 34 41 Ar
60 35 36 2
61 35 71 1
62 36 37 Ar
63 36 72 1
64 37 38 Ar
65 37 41 Ar
66 38 39 Ar
67 38 169 1
68 39 40 Ar
69 39 73 1
70 40 74 1
71 42 44 Ar
72 42 50 Ar
73 43 49 Ar
74 43 50 Ar
75 44 45 2
76 44 81 1
77 45 46 Ar
78 45 82 1
79 46 47 Ar
80 46 50 Ar
81 47 48 Ar
82 47 118 1
83 48 49 Ar
84 48 80 1
85 49 79 1
86 51 53 Ar
87 51 59 Ar
88 52 58 Ar
89 52 59 Ar
90 53 54 2
91 53 75 1
92 54 55 Ar
93 54 76 1
94 55 56 Ar
95 55 59 Ar
96 56 57 Ar
97 56 101 1
98 57 58 Ar
99 57 77 1

100 58 78 1
101 84 85 Ar
102 84 86 2
103 85 87 2
104 85 88 1
105 86 89 Ar
106 86 90 1
107 87 91 Ar
108 87 92 1
109 89 91 Ar
110 89 93 Ar
111 91 94 Ar
112 93 95 2
113 93 96 1
114 94 97 2
115 94 98 1
116 95 97 Ar
117 95 99 1
118 97 100 1
119 101 102 Ar
120 101 103 2
121 102 104 2
122 102 105 1
123 103 106 Ar
124 103 107 1
125 104 108 Ar
126 104 109 1
127 106 108 Ar
128 106 110 Ar
129 108 111 Ar
130 110 112 2
131 110 113 1
132 111 114 2
133 111 115 1
134 112 114 Ar
135 112 116 1
136 114 117 1
137 118 119 Ar
138 118 120 2
139 119 121 2
140 119 122 1
141 120 123 Ar
142 120 124 1
143 121 125 Ar
144 121 126 1
145 123 125 Ar
146 123 127 Ar
147 125 128 Ar
148 127 129 2
149 127 130 1
150 128 131 2
151 128 132 1
152 129 131 Ar
153 129 133 1
154 131 134 1
155 135 136 Ar
156 135 137 2
157 136 138 2
158 136 139 1
159 137 140 Ar

160 137 141 1
161 138 142 Ar
162 138 143 1
163 140 142 Ar
164 140 144 Ar
165 142 145 Ar
166 144 146 2
167 144 147 1
168 145 148 2
169 145 149 1
170 146 148 Ar
171 146 150 1
172 148 151 1
173 152 153 Ar
174 152 154 2
175 153 155 2
176 153 156 1
177 154 157 Ar
178 154 158 1
179 155 159 Ar
180 155 160 1
181 157 159 Ar
182 157 161 Ar
183 159 162 Ar
184 161 163 2
185 161 164 1
186 162 165 2
187 162 166 1
188 163 165 Ar
189 163 167 1
190 165 168 1
191 169 170 Ar
192 169 171 2
193 170 172 2
194 170 173 1
195 171 174 Ar
196 171 175 1
197 172 176 Ar
198 172 177 1
199 174 176 Ar
200 174 178 Ar
201 176 179 Ar
202 178 180 2
203 178 181 1
204 179 182 2
205 179 183 1
206 180 182 Ar
207 180 184 1
208 182 185 1

Zn-3 S_1
@<TRIPOS>MOLECULE
Molecule Name
185 207
SMALL
NO_CHARGES

@<TRIPOS>ATOM
1 Zn1 0.2836 0.5480 1.7600 Zn

2 Zn2	0.9128	0.9525	-1.3864	Zn
3 Zn3	0.0227	-1.9620	-0.2914	Zn
4 Zn4	-2.1161	0.4525	-0.4076	Zn
5 O5	-0.2253	-0.0077	-0.0890	O
6 N6	1.4900	2.2049	1.6485	N
7 N7	2.5848	1.8157	-0.4452	N
8 C8	1.7873	3.0215	2.7149	C
9 C9	2.9696	3.7084	2.5557	C
10 C10	3.4796	3.3039	1.2898	C
11 C11	4.6056	3.5959	0.5039	C
12 C12	4.6803	2.9615	-0.7438	C
13 C13	3.6765	2.1005	-1.1727	C
14 C14	2.5064	2.3872	0.7710	C
15 N15	0.4279	-2.8193	1.5472	N
16 N16	1.4417	-0.9495	2.6449	N
17 C17	0.2845	-4.1110	1.8664	C
18 C18	0.9921	-4.4234	3.0725	C
19 C19	1.6283	-3.2589	3.4809	C
20 C20	2.4418	-2.8803	4.6296	C
21 C21	2.6761	-1.4592	4.6988	C
22 C22	2.2297	-0.5965	3.7503	C
23 C23	1.2111	-2.2624	2.5432	C
24 N24	-1.4177	0.8077	2.8743	N
25 N25	-3.2342	0.3931	1.3554	N
26 C26	-1.3771	1.0100	4.2346	C
27 C27	-2.6202	0.9980	4.8212	C
28 C28	-3.5381	0.7634	3.7612	C
29 C29	-4.9259	0.5934	3.6473	C
30 C30	-5.4174	0.2969	2.3693	C
31 C31	-4.5626	0.2107	1.2781	C
32 C32	-2.7301	0.6487	2.5787	C
33 N33	1.6247	-0.3454	-2.7908	N
34 N34	1.7197	-2.3573	-1.4871	N
35 C35	2.1909	0.0090	-3.9942	C
36 C36	3.0015	-0.9713	-4.5174	C
37 C37	2.9598	-2.0428	-3.5812	C
38 C38	3.5250	-3.3241	-3.4795	C
39 C39	3.1602	-4.0802	-2.3584	C
40 C40	2.2774	-3.5750	-1.4107	C
41 C41	2.0748	-1.6019	-2.5436	C
42 N42	-2.2538	2.2864	-1.2783	N
43 N43	-0.0728	2.6072	-2.2398	N
44 C44	-3.4038	3.0409	-1.3114	C
45 C45	-3.2695	4.1934	-2.0480	C
46 C46	-1.9266	4.1923	-2.5165	C
47 C47	-1.1349	5.0389	-3.3070	C
48 C48	0.1775	4.6160	-3.5515	C
49 C49	0.6592	3.4335	-3.0046	C
50 C50	-1.3474	2.9801	-2.0092	C
51 N51	-1.5978	-2.8228	-1.1937	N
52 N52	-3.0083	-0.9450	-1.6990	N
53 C53	-1.6480	-4.1455	-1.5688	C
54 C54	-2.7308	-4.4429	-2.3615	C
55 C55	-3.4330	-3.2166	-2.5212	C
56 C56	-4.5682	-2.7870	-3.2258	C
57 C57	-4.8601	-1.4182	-3.1655	C
58 C58	-4.0782	-0.5532	-2.4099	C
59 C59	-2.6794	-2.2486	-1.7757	C
60 H60	1.1002	3.0755	3.5522	H
61 H61	3.3965	4.4265	3.2414	H

62	H62	5.5444	3.1166	-1.3819	H
63	H63	3.7478	1.6047	-2.1381	H
64	H64	-0.3101	-4.7814	1.2578	H
65	H65	0.9680	-5.3796	3.5777	H
66	H66	3.3104	-1.0573	5.4814	H
67	H67	2.4924	0.4570	3.7953	H
68	H68	-0.4197	1.1372	4.7282	H
69	H69	-2.8394	1.1090	5.8734	H
70	H70	-6.4828	0.1645	2.2103	H
71	H71	1.9559	0.9732	-4.4314	H
72	H72	3.5285	-0.9389	-5.4604	H
73	H73	3.5878	-5.0654	-2.2023	H
74	H74	2.0078	-4.1685	-0.5403	H
75	H75	-0.8634	-4.8234	-1.2506	H
76	H76	-2.9691	-5.4024	-2.7976	H
77	H77	-5.7269	-1.0210	-3.6837	H
78	H78	-4.3138	0.5076	-2.3715	H
79	H79	1.6947	3.1459	-3.1664	H
80	H80	0.8527	5.2366	-4.1317	H
81	H81	-4.2822	2.7004	-0.7736	H
82	H82	-4.0341	4.9337	-2.2345	H
83	H83	-4.9574	-0.0171	0.2912	H
84	C84	-5.8432	0.7105	4.7971	C
85	C85	-6.9373	-0.1883	4.9286	C
86	C86	-5.6788	1.7003	5.7468	C
87	C87	-7.8138	-0.0858	5.9773	C
88	H88	-7.0560	-0.9836	4.1978	H
89	C89	-6.5727	1.8380	6.8349	C
90	H90	-4.8621	2.4108	5.6469	H
91	C91	-7.6637	0.9262	6.9581	C
92	H92	-8.6374	-0.7898	6.0720	H
93	C93	-6.4201	2.8584	7.8078	C
94	C94	-8.5545	1.0632	8.0503	C
95	C95	-7.3016	2.9674	8.8553	C
96	H96	-5.5900	3.5542	7.7102	H
97	C97	-8.3787	2.0606	8.9787	C
98	H98	-9.3829	0.3644	8.1416	H
99	H99	-7.1734	3.7529	9.5951	H
100	H100	-9.0690	2.1568	9.8124	H
101	C101	-5.4236	-3.7039	-4.0034	C
102	C102	-5.9522	-3.2916	-5.2575	C
103	C103	-5.7508	-4.9576	-3.5240	C
104	C104	-6.7679	-4.1176	-5.9861	C
105	H105	-5.6767	-2.3166	-5.6501	H
106	C106	-6.5987	-5.8288	-4.2479	C
107	H107	-5.3755	-5.2777	-2.5553	H
108	C108	-7.1192	-5.4044	-5.5074	C
109	H109	-7.1532	-3.7938	-6.9503	H
110	C110	-6.9522	-7.1118	-3.7583	C
111	C111	-7.9649	-6.2791	-6.2320	C
112	C112	-7.7764	-7.9374	-4.4831	C
113	H113	-6.5575	-7.4314	-2.7965	H
114	C114	-8.2872	-7.5176	-5.7324	C
115	H115	-8.3571	-5.9522	-7.1924	H
116	H116	-8.0395	-8.9187	-4.0977	H
117	H117	-8.9382	-8.1801	-6.2964	H
118	C118	-1.6271	6.3197	-3.8496	C
119	C119	-1.2704	6.7223	-5.1658	C
120	C120	-2.4112	7.1629	-3.0863	C
121	C121	-1.6981	7.9196	-5.6784	C

122	H122	-0.6722	6.0520	-5.7773	H
123	C123	-2.8602	8.4086	-3.5839	C
124	H124	-2.6676	6.8836	-2.0675	H
125	C125	-2.5003	8.7988	-4.9087	C
126	H126	-1.4286	8.2077	-6.6919	H
127	C127	-3.6553	9.2855	-2.8032	C
128	C128	-2.9543	10.0449	-5.4053	C
129	C129	-4.0793	10.4887	-3.3116	C
130	H130	-3.9240	8.9875	-1.7924	H
131	C131	-3.7263	10.8718	-4.6256	C
132	H132	-2.6798	10.3375	-6.4162	H
133	H133	-4.6889	11.1521	-2.7042	H
134	H134	-4.0679	11.8263	-5.0167	H
135	C135	5.6778	4.5075	0.9473	C
136	C136	6.2781	5.4051	0.0221	C
137	C137	6.1386	4.4900	2.2495	C
138	C138	7.2901	6.2453	0.4075	C
139	H139	5.9030	5.4380	-0.9972	H
140	C140	7.1888	5.3372	2.6750	C
141	H141	5.7093	3.7904	2.9622	H
142	C142	7.7785	6.2378	1.7380	C
143	H143	7.7294	6.9365	-0.3082	H
144	C144	7.6780	5.3162	4.0058	C
145	C145	8.8267	7.0877	2.1675	C
146	C146	8.6976	6.1510	4.3924	C
147	H147	7.2300	4.6258	4.7167	H
148	C148	9.2770	7.0463	3.4648	C
149	H149	9.2715	7.7743	1.4507	H
150	H150	9.0635	6.1262	5.4153	H
151	H151	10.0829	7.7021	3.7828	H
152	C152	3.0226	-3.7986	5.5485	C
153	C153	3.4557	-3.3623	6.8491	C
154	C154	3.2160	-5.1617	5.2420	C
155	C155	3.9905	-4.2263	7.7562	C
156	H156	3.3018	-2.3268	7.1362	H
157	C157	3.7782	-6.0698	6.1602	C
158	H158	2.9948	-5.5209	4.2421	H
159	C159	4.1736	-5.6093	7.4545	C
160	H160	4.2799	-3.8677	8.7418	H
161	C161	3.9803	-7.4383	5.8399	C
162	C162	4.7306	-6.5190	8.3714	C
163	C163	4.5313	-8.3054	6.7560	C
164	H164	3.6889	-7.7928	4.8536	H
165	C165	4.9079	-7.8474	8.0343	C
166	H166	5.0257	-6.1585	9.3545	H
167	H167	4.6774	-9.3500	6.4940	H
168	H168	5.3406	-8.5394	8.7514	H
169	C169	4.4669	-3.8651	-4.4782	C
170	C170	4.3890	-5.2306	-4.8672	C
171	C171	5.4552	-3.0728	-5.0295	C
172	C172	5.2698	-5.7576	-5.7757	C
173	H173	3.5984	-5.8508	-4.4537	H
174	C174	6.3852	-3.5902	-5.9615	C
175	H175	5.5459	-2.0342	-4.7219	H
176	C176	6.2931	-4.9611	-6.3477	C
177	H177	5.1887	-6.8004	-6.0741	H
178	C178	7.4116	-2.7882	-6.5217	C
179	C179	7.2234	-5.4757	-7.2833	C
180	C180	8.3009	-3.3161	-7.4253	C
181	H181	7.4824	-1.7451	-6.2221	H

182 C182 8.2055 -4.6726 -7.8108 C
183 H183 7.1482 -6.5203 -7.5765 H
184 H184 9.0831 -2.6915 -7.8481 H
185 H185 8.9149 -5.0786 -8.5267 H
@<TRIPOS>BOND
1 1 5 1
2 1 6 1
3 1 24 1
4 2 5 1
5 2 33 1
6 3 5 1
7 3 51 1
8 4 5 1
9 4 42 1
10 6 8 Ar
11 6 14 Ar
12 7 13 Ar
13 7 14 Ar
14 8 9 2
15 8 60 1
16 9 10 Ar
17 9 61 1
18 10 11 Ar
19 10 14 Ar
20 11 12 Ar
21 11 135 1
22 12 13 Ar
23 12 62 1
24 13 63 1
25 15 17 Ar
26 15 23 1
27 16 22 1
28 16 23 Ar
29 17 18 Ar
30 17 64 1
31 18 19 Ar
32 18 65 1
33 19 20 1
34 19 23 Ar
35 20 21 Ar
36 20 152 Ar
37 21 22 2
38 21 66 1
39 22 67 1
40 24 26 Ar
41 24 32 Ar
42 25 31 Ar
43 25 32 Ar
44 26 27 2
45 26 68 1
46 27 28 Ar
47 27 69 1
48 28 29 Ar
49 28 32 Ar
50 29 30 Ar
51 29 84 1
52 30 31 Ar
53 30 70 1
54 31 83 1
55 33 35 Ar

56 33 41 Ar
57 34 40 Ar
58 34 41 Ar
59 35 36 2
60 35 71 1
61 36 37 Ar
62 36 72 1
63 37 38 Ar
64 37 41 Ar
65 38 39 Ar
66 38 169 1
67 39 40 Ar
68 39 73 1
69 40 74 1
70 42 44 Ar
71 42 50 Ar
72 43 49 Ar
73 43 50 Ar
74 44 45 2
75 44 81 1
76 45 46 Ar
77 45 82 1
78 46 47 Ar
79 46 50 Ar
80 47 48 Ar
81 47 118 1
82 48 49 Ar
83 48 80 1
84 49 79 1
85 51 53 Ar
86 51 59 Ar
87 52 58 Ar
88 52 59 Ar
89 53 54 2
90 53 75 1
91 54 55 Ar
92 54 76 1
93 55 56 Ar
94 55 59 Ar
95 56 57 Ar
96 56 101 1
97 57 58 Ar
98 57 77 1
99 58 78 1
100 84 85 Ar
101 84 86 2
102 85 87 2
103 85 88 1
104 86 89 Ar
105 86 90 1
106 87 91 Ar
107 87 92 1
108 89 91 Ar
109 89 93 Ar
110 91 94 Ar
111 93 95 2
112 93 96 1
113 94 97 2
114 94 98 1
115 95 97 Ar

116 95 99 1
117 97 100 1
118 101 102 Ar
119 101 103 2
120 102 104 2
121 102 105 1
122 103 106 Ar
123 103 107 1
124 104 108 Ar
125 104 109 1
126 106 108 Ar
127 106 110 Ar
128 108 111 Ar
129 110 112 2
130 110 113 1
131 111 114 2
132 111 115 1
133 112 114 Ar
134 112 116 1
135 114 117 1
136 118 119 Ar
137 118 120 2
138 119 121 2
139 119 122 1
140 120 123 Ar
141 120 124 1
142 121 125 Ar
143 121 126 1
144 123 125 Ar
145 123 127 Ar
146 125 128 Ar
147 127 129 2
148 127 130 1
149 128 131 2
150 128 132 1
151 129 131 Ar
152 129 133 1
153 131 134 1
154 135 136 Ar
155 135 137 2
156 136 138 2
157 136 139 1
158 137 140 Ar
159 137 141 1
160 138 142 Ar
161 138 143 1
162 140 142 Ar
163 140 144 Ar
164 142 145 Ar
165 144 146 2
166 144 147 1
167 145 148 2
168 145 149 1
169 146 148 Ar
170 146 150 1
171 148 151 1
172 152 153 Ar
173 152 154 Ar
174 153 155 2
175 153 156 1

176 154 157 Ar
177 154 158 1
178 155 159 Ar
179 155 160 1
180 157 159 Ar
181 157 161 Ar
182 159 162 Ar
183 161 163 2
184 161 164 1
185 162 165 2
186 162 166 1
187 163 165 Ar
188 163 167 1
189 165 168 1
190 169 170 Ar
191 169 171 2
192 170 172 2
193 170 173 1
194 171 174 Ar
195 171 175 1
196 172 176 Ar
197 172 177 1
198 174 176 Ar
199 174 178 Ar
200 176 179 Ar
201 178 180 2
202 178 181 1
203 179 182 2
204 179 183 1
205 180 182 Ar
206 180 184 1
207 182 185 1

23. References

1 (*a*) W.-M. Kwok, C. Ma and D. L. Phillips, *J. Am. Chem. Soc.*, 2006, **128**, 11894; (*b*) W.-M. Kwok, C. Ma and D. L. Phillips, *J. Am. Chem. Soc.*, 2008, **130**, 5131; (*c*) C. T. L. Chan, C. C. W. Cheng, K. Y. F. Ho and W. M. Kwok, *Phys. Chem. Chem. Phys.*, 2011, **13**, 16306.

2 W. Lu, W. M. Kwok, C. Ma, T. L. Chan, M. X. Zhu and C.-M. Che, *J. Am. Chem. Soc.*, 2011, **133**, 14120.

3 (*a*) P. Matousek, M. Towrie, C. Ma, W. M. Kwok, D. Phillips, W. T. Toner and A. W. Parker, *J. Raman Spectrosc.*, 2001, **32**, 983; (*b*) P. Matousek, M. Towrie, A. Stanley and A. W. Parker, *Appl. Spectrosc.*, 1999, **53**, 1485.

4 M. J. Frisch, G. W. Trucks, H. B. Schlegel, G. E. Scuseria, M. A. Robb, J. R. Cheeseman, G. Scalmani, V. Barone, B. Mennucci, G. A. Petersson, H. Nakatsuji, M. Caricato, X. Li, H. P. Hratchian, A. F. Izmaylov, J. Bloino, G. Zheng, J. L. Sonnenberg, M. Hada, M. Ehara, K. Toyota, R. Fukuda, J. Hasegawa, M. Ishida, T. Nakajima, Y. Honda, O. Kitao, H. Nakai, T. Vreven, J. A. Montgomery, J. E. Peralta, F. Ogliaro, M. Bearpark, J. J. Heyd, E. Brothers, K. N. Kudin, V. N. Staroverov, R. Kobayashi, J. Normand, K. Raghavachari, A. Rendell, J. C. Burant, S. S. Iyengar, J. Tomasi, M. Cossi, N. Rega, J. M. Millam, M. Klene, J. E. Knox, J. B. Cross, V. Bakken, C. Adamo, J. Jaramillo, R. Gomperts, R. E. Stratmann, O. Yazyev, A. J. Austin, R. Cammi, C. Pomelli, J. W. Ochterski, R. L. Martin, K. Morokuma, V. G. Zakrzewski, G. A. Voth, P. Salvador, J. J. Dannenberg, S. Dapprich, A. D. Daniels, O. Farkas, J. B. Foresman, J. V. Ortiz, J. Cioslowski and D. J. Fox, *Gaussian 09*, revision D.01; Gaussian, Inc., Wallingford, CT, **2013**.

5 (*a*) J. P. Perdew, K. Burke and M. Ernzerhof, *Phys. Rev. Lett.*, 1996, 3865; (*b*) J. P. Perdew, K. Burke and M. Ernzerhof, *Phys. Rev. Lett.*, 1997, **78**, 1396; (*c*) C. Adamo and V. Barone, *J. Chem. Phys.*, 1999, **110**, 6158.

6 (*a*) P. J. Hay and W. R. Wadt, *J. Chem. Phys.*, 1985, **82**, 270; (*b*) W. R. Wadt and P. J. Hay, *J. Chem. Phys.*, 1985, **82**, 284; (*c*) P. J. Hay and W. R. Wadt, *J. Chem. Phys.*, 1985, **82**, 299.

7 (*a*) O. Tapia, *J. Math. Chem.*, 1992, **10**, 139; (*b*) J. Tomasi and M. Persico, *Chem. Rev.*, 1994, **94**, 2027.

8 F. Teixidor, C. Viñas, M. Mar Abad, R. Nuñez, R. Kivekäs and R. Sillanpäa, *J. Organomet. Chem.*, 1995, **503**, 193.
